# Chromosome-level reference genome of tetraploid *Isoetes sinensis* provides insights into evolution and adaption of lycophytes

**DOI:** 10.1093/gigascience/giad079

**Published:** 2023-09-30

**Authors:** Jinteng Cui, Yunke Zhu, Hai Du, Zhenhua Liu, Siqian Shen, Tongxin Wang, Wenwen Cui, Rong Zhang, Sanjie Jiang, Yanmin Wu, Xiaofeng Gu, Hao Yu, Zhe Liang

**Affiliations:** College of Landscape Architecture, Beijing University of Agriculture, Beijing 102206, China; Biotechnology Research Institute, Chinese Academy of Agricultural Sciences, Beijing 100081, China; Glbizzia Biosciences, Beijing 102699, China; College of Agronomy and Biotechnology, Southwest University, Chongqing 400715, China; BiosmartSeek, Wuhan 430072, China; College of Landscape Architecture, Beijing University of Agriculture, Beijing 102206, China; College of Landscape Architecture, Beijing University of Agriculture, Beijing 102206, China; College of Landscape Architecture, Beijing University of Agriculture, Beijing 102206, China; Fisheries Science Institute, Beijing Academy of Agriculture and Forestry Sciences, Beijing 100068, China; BGI Genomics, Shenzhen 518083, China; Biotechnology Research Institute, Chinese Academy of Agricultural Sciences, Beijing 100081, China; Biotechnology Research Institute, Chinese Academy of Agricultural Sciences, Beijing 100081, China; Department of Biological Sciences, National University of Singapore, Singapore 117543, Singapore; Biotechnology Research Institute, Chinese Academy of Agricultural Sciences, Beijing 100081, China

**Keywords:** *Isoetes sinensis*, genome, evolution, Lycophyta, polyploid, environmental stress

## Abstract

**Background:**

The Lycophyta species are the extant taxa most similar to early vascular plants that were once abundant on Earth. However, their distribution has greatly diminished. So far, the absence of chromosome-level assembled lycophyte genomes has hindered our understanding of evolution and environmental adaption of lycophytes.

**Findings:**

We present the reference genome of the tetraploid aquatic quillwort, *Isoetes sinensis*, a lycophyte. This genome represents the first chromosome-level assembled genome of a tetraploid seed-free plant. Comparison of genomes between *I. sinensis* and *Isoetestaiwanensis* revealed conserved and different genomic features between diploid and polyploid lycophytes. Comparison of the *I. sinensis* genome with those of other species representing the evolutionary lineages of green plants revealed the inherited genetic tools for transcriptional regulation and most phytohormones in *I. sinensis*. The presence and absence of key genes related to development and stress responses provide insights into environmental adaption of lycophytes.

**Conclusions:**

The high-quality reference genome and genomic analysis presented in this study are crucial for future genetic and environmental studies of not only *I. sinensis* but also other lycophytes.

## Introduction

The vascular plants that currently dominate the land can be categorized into 2 major phyla: Euphyllophyta and Lycophyta. Euphyllophyta includes seed plants and ferns, while Lycophyta comprises spore-bearing species that exhibit the greatest similarity to the early vascular plants found in the fossil record. Lycophytes have the longest evolutionary history among all groups of vascular plants and have had major impacts on biodiversity, soil formation [1], and CO_2_ sequestration on our planet [2]. Modern lycophytes have a widespread distribution, ranging from the epiphytic habitats (e.g., *Lycopodium phlegmaria* [3]) to the aquatic habits (e.g., *Phylloglossum drummondii*). Some members of the Lycophyta can survive in a variety of extreme environments, such as deserts (e.g., *Selaginella lepidophylla* [4]), humid tropics (e.g., *Selaginella kraussiana*), and even arctic and alpine regions [3]. However, the distribution area of lycophytes has been greatly reduced when compared to seed plants. Some lycophytes, including several species in the lycopod genus *Isoetes*, are endangered [5, 6]. The genetic basis for environmental adaptability of lycophytes remains largely unknown.

**Figure 1:**
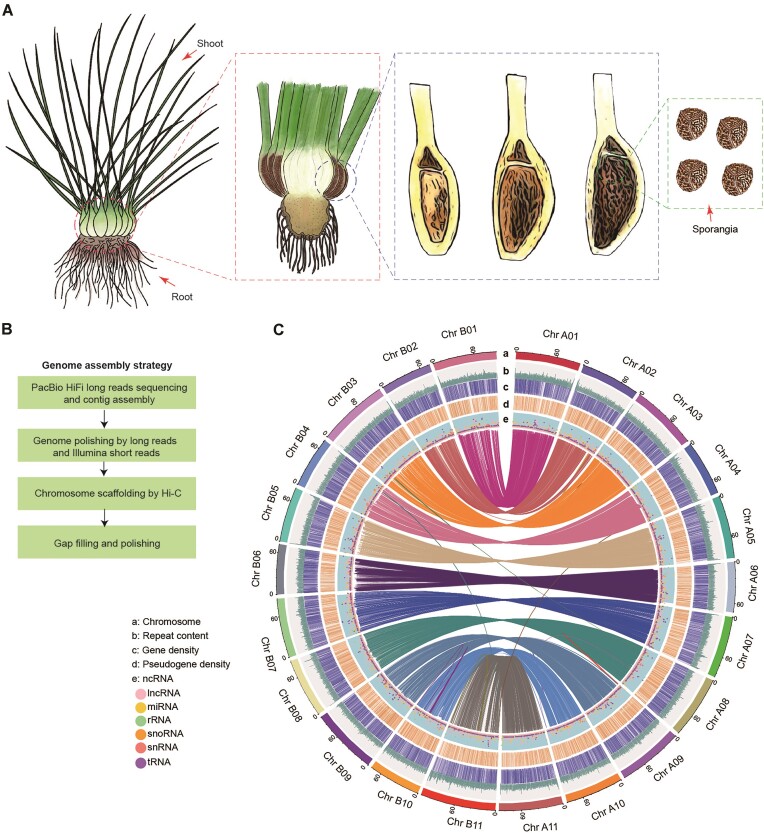
*I. sinensis* morphology and genome assembly and annotation. (A) Morphological diagram of *I. sinensis*. The main body of the plant is 15 to 30 cm high, consisting of a rhizomatous and trilobed corm, with a tuft of roots at the base and long imbricate leaves at the top. The sporangia are basal and contain megaspores and/or microspores. The tissues analyzed using RNA sequencing are indicated by arrows. (B) Diagram depicts workflow for assembly of the *I. sinensis* genome from PacBio HiFi long reads, Illumina short reads, and Hi-C data. (C) Circos plot represents the *I. sinensis* genome, including (a) 22 assembled pseudochromosomes, (b) repeat content, (c) gene density, (d) pseudogene density, and (e) ncRNAs including lncRNAs, miRNAs, rRNAs, snoRNAs, snRNAs, and tRNAs. Blocks of synteny of at least 5 gene pairs between the genomes are connected by linked lines at the center of the Circos plot. Different colors represent different pseudochromosomes or syntenic blocks. A 500-kb window size was selected to slide on the genome, and the maximum repeat content and ncRNA content on each window were 1,696 and 1,146 respectively.

Lycophytes included diploid and polyploid species in many lineages. So far, 4 genomes of diploid lycophytes, including *Selaginella moellendorffii* [7], *Selaginella tamariscina* [8], *Lycopodium clavatum* [9], and *Isoetes taiwanensis* [10], are available. However, they are scaffold assemblies, not chromosome-level assemblies. To date, the genomes of polyploid lycophytes have not yet been reported. The perennial aquatic lycophyte, *Isoetes sinensis* (NCBI:txid283158) (Fig. 1A), is a tetraploid (2n = 4x = 44) quillwort and belongs to the family Isoetaceae that diversified 45 to 60 million years ago [11]. Among extant representatives of the earliest differentiated vascular plants [12, 13], *I. sinensis* was once widely distributed but has now completely disappeared from most of their habitats except 2 restricted sites in China [14]. Like other *Isoetes, I. sinensis* possesses a Crassulacean acid metabolism (CAM) system that is crucial for the plant adaptation to a low CO_2_ environment underwater [15].

Here, we report a reference genome sequence of *I. sinensis* assembled into 22 pseudochromosomes. Our comparative analyses of its genome with *I. taiwanensis* and those of green algae and land plants allow us to better understand the evolution of lycophytes and the genetic basis of the environmental adaptability of lycophytes.

## Results and Discussion

### Assembly of a high-quality *Isoetes sinensis* genome

**Figure 2:**
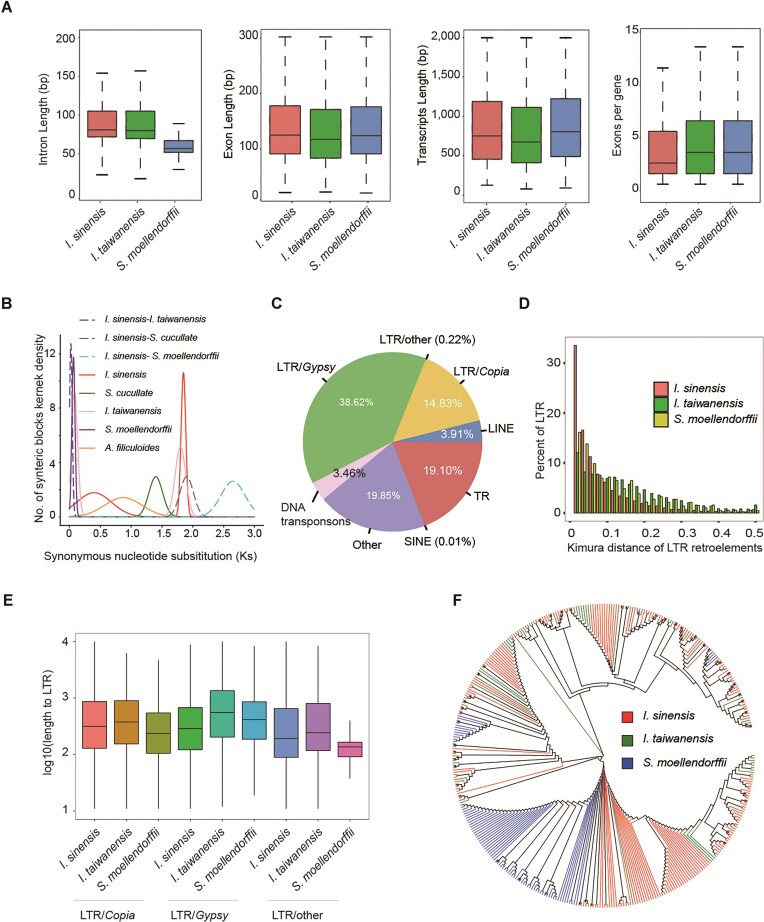
Genomic features of the *I. sinensis* genome. (A) Boxplot showing intron, exon, and transcript length comparisons among the genomes of *I. sinensis, I. taiwanensis*, and *S. moellendorffii*. Boxes indicate the first quartile, median, and third quartile with whiskers extending up to 1.5 times the interquartile distance. (B) Frequency distribution of *Ks* based on the distribution of substitution rates of paralogs in 3 lycophytes (*I. sinensis, I. taiwanensis, S. moellendorffii*) and 2 ferns (*A. filiculoides* and *S. cucullata*). The two *Ks* peaks (0.4 and 1.8) indicate 2 WGDs in *I. sinensis*. (C) Pie chart illustrating of the major classes of repetitive DNA in *I. sinensis*. LINE, long interspersed nuclear element; LTR, long terminal repeat; SINE, short interspersed transposable element; TR, tandem repeat. (D) The relative ages of LTR retroelements computed as Kimura distances suggest a long period of retroelement transposition activity. (E) Boxplot showing distributions of LTR family lengths in *I. sinensis, I. taiwanensis*, and *S. moellendorffii*. (F) Maximum likelihood phylogeny analysis of *Gypsy* retroelements showing the expansion of *Gypsy* in *I. sinensis* and *I. taiwanensis*.

Our *k*-mer analysis revealed the genome size of *I. sinensis* to be approximately 2.25 Gb with a heterozygosity value of 0.26%. We sequenced the *I. sinensis* genome by generating 176.46 Gb (79.17× coverage) Illumina short reads, 97.01 Gb (43.52× coverage) PacBio SMRT HiFi long reads, and 237.7 Gb (111.50× coverage) Hi-C data. We subsequently assembled the 2.13 Gb *I. sinensis* genome into 22 pseudochromosomes consisting of 3,741 scaffolds with N50 length of 86.66 Mb (Fig. 1B, C; Supplementary Fig. S1A, S1B; Table 1; Supplementary Tables S1 and S2). The longest chromosome is ∼109.03 Mb and the shortest is ∼70.83 Mb (Supplementary Table S3). Using a combination of Illumina and PacBio sequencing, we performed RNA sequencing (RNA-seq) of small RNAs, long noncoding RNAs (lncRNAs), and messenger RNAs (mRNAs) isolated from different tissues of *I. sinensis* to facilitate genome annotation (Supplementary Table S4). By combining homology-based alignments and *ab initio* gene models, we annotated a total of 57,303 protein-coding genes, 75% of which were supported by RNA-seq data (Table 1). In total, 52,531 coding genes (92%) were assigned to functional categories using the InterPro, NR, Swiss-Prot, and KEGG databases. BUSCO (96.5%) and CEGMA (98.39%) analyses suggest that our genome assembly exhibits a high degree of completeness (Supplementary Table S5). LTR Assembly Index (LAI) value is 9.71, which was not high but among the top LAI values reported in polyploid genomes [16]. The lengths of exons and transcripts are comparable among *I. sinensis* and its closely related species *I. taiwanensis* and *S. moellendorffii*, while *I. sinensis* has fewer exons per gene and shorter introns (Fig. 2A and Supplementary Fig. S1C). We annotated 33,515 noncoding RNA (ncRNA) genes, including 8,975 transfer RNA (tRNA), 17,453 ribosomal RNA (rRNA), 1,797 microRNA (miRNA), 1,194 small nuclear RNA (snRNA), 279 small nucleolar RNA (snoRNA), and 3,817 lncRNA genes (Fig. 1C; Table 1; Supplementary Tables S6–S12). Further, we annotated 12,886 pseudogenes containing frameshift mutations, premature stop codons, or both (Supplementary Table S13).

**Table 1:** Statistics of *I. sinensis* genome assembly and annotation

Feature	*Isoetes sinensis*
Genome size (bp)	2,131,756,688
Contig number	4,329
Maximum contig length (bp)	13,293,339
Contig N50 (bp)	2,139,932
Contig N90 (bp)	228,882
Scaffold N50 (bp)	86,663,717
Scaffold N90 (bp)	70,828,552
Gene number	57,303
Average gene length (bp)	3,031.29
Average CDS length (bp)	1,098.39
Exon number per gene	4.79
Average exon length (bp)	294.98
Intron number per gene	3.79
Average intron length (bp)	426.34

### Gene and genome evolution

**Figure 3:**
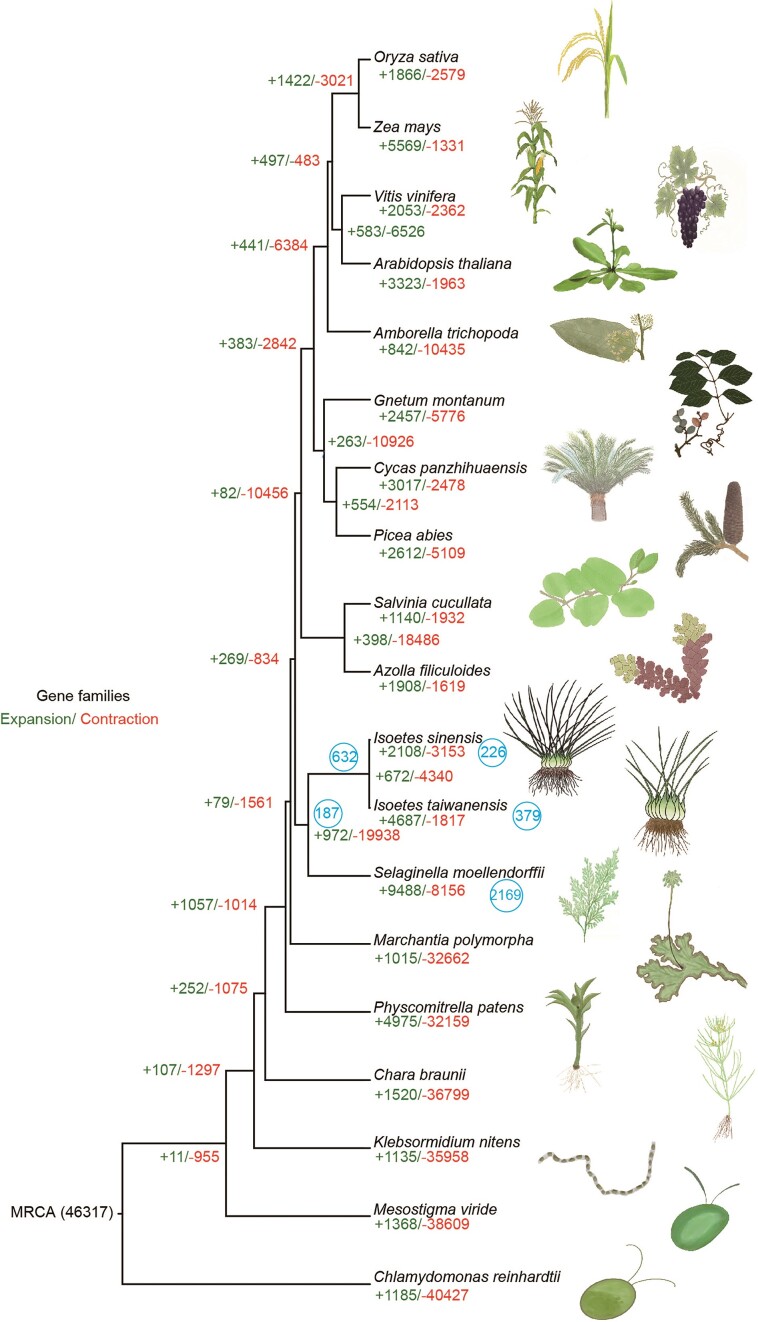
Evolution analysis of gene families in *I. sinensis* and 19 selected evolutionarily representative green algae and land plants. The phylogenetic tree was constructed from the ML method. The green numbers on the branches of the phylogenetic tree indicate the number of expanded gene families, and the red numbers refer to the number of constricted gene families. The supposed most recent common ancestor (MRCA) contains 46,317 gene families. Totals of 2,108 and 3,153 families had undergone expansion and contraction in *I. sinensis*, respectively. Only 1 subgenome of *I. sinensis* was used. The number in the blue circle indicates the retained duplicates from WGDs.

Our maximum likelihood (ML) phylogeny of 19 species of evolutionarily representative land plants and green algae indicates that *I. sinensis* and *I. taiwanensis* diverged from *S. moellendorffii* about 300 million years ago (Fig. 3). One hypothesis has suggested that the tetraploid *I. sinensis* originated from hybridization between the diploid *Isoetes yunguiensis* and *I. taiwanensis* [17]. We attempted to distinguish the *I. sinensis* genome into 2 subgenomes using genomic information from *I. taiwanensis*. However, genome-wide comparison (Supplementary Table S14) and phylogenetic analysis (Supplementary Fig. S2A) showed that the similarity between pairs of chromosomes of *I. sinensis* was greater than that between *I. sinensis* and *I. taiwanensis*, suggesting that *I. sinensis* was not directly derived from the hybridization of *I. yunguiensis* and *I. taiwanensis*. We further performed *k*-mer and SubPhaser analysis. Clustering of counts of 13-mers identified 2 groups of chromosomes. However, pairs of chromosomes, such as Chr 3 and Chr 4, were found in the same groups (Supplementary Fig. S3A). In addition, SubPhaser analysis identified 9 chromosomes in subgenome 1 and 13 chromosomes in subgenome 2 (Supplementary Fig. S3B). These results suggested that our analyses were not able to identify the 2 subgenomes of *I. sinensis*. To facilitate the subsequent analysis, we adopted an approach similar to that used for the *Artemisia argyi* genome assembly [18] and artificially divided the *I. sinensis* genome into 2 subgenomes, A and B, based on the lengths of chromosome pairs (Supplementary Table S3). Gene numbers were comparable between the 2 subgenomes, with 93.4% of subgenome A genes as homoeologs of 95.0% of subgenome B genes (Supplementary Fig. S2B). We found high collinearity between allelic chromosome pairs (i.e., A01 and B01) but weaker collinearity between other regions (Supplementary Fig. S4A and Supplementary Tables S15 and S16), indicating the stability of *I. sinensis* as a tetraploid species. Abundant synteny blocks were observed between *I. sinensis* and *I. taiwanensis* (Supplementary Fig. S4B), suggesting that collinear blocks were retained after polyploidization. The collinearity between seed-free and seed plants was little known due to lack of chromosomal genome assembly of seed-free plants. We found only 2 plausible synteny blocks between *I. sinensis* and *Arabidopsis thaliana* and *Zea mays* (Supplementary Fig. S4C), which illustrates the very limited collinearity between *I. sinensis* and seed plants.

Gene family expansions and contractions are often closely related to the adaptive evolution of species [19]. We distinguished expansion and contraction of gene families among representative plant species using homology-based methods. In total, 2,108 and 3,153 families had undergone expansion and contraction in *I. sinensis*, respectively (Fig. 3). Expanded gene families were mostly enriched for energy metabolism functions such as photosynthesis and oxidative phosphorylation, while contracted gene families were mostly enriched in lipid metabolism functions such as linoleic acid metabolism and fatty acid degradation (Supplementary Fig. S5). Notably, many more gene families that had expanded (4,687) and fewer that had contracted (1,817) were found in *I. taiwanensis* than in *I. sinensis* (Fig. 3), suggesting high genetic variation within *Isoetes*.

Diploid A and B subgenomes shared 15,280 orthologous gene families, which include 3,007 and 2,103 multicopy gene families in the A and B subgenomes, respectively. Of the orthologous single-copy gene sets in *I. taiwanensis*, 909 and 1,187 genes had been lost from the A and B subgenomes, respectively, of *I. sinensis*. These gene losses were also coincident with the smaller chromosome size of *I. sinensis* (96.8 Mb on average) relative to that of *I. taiwanensis* (150.9 Mb). Furthermore, 6,578 genes that exist as a single copy in *I. taiwanensis* still exist as a single copy (1 copy per subgenome) in each of the 2 *I. sinensis* subgenomes. To understand the effect of polyploidization on gene expression, we analyzed the gene expression bias between pairs of chromosomes in *I. sinensis* by using a similar approach reported in *Brassica juncea* [20]. On average, 5,206 gene pairs showed homoeolog expression dominance. Notably, the number of dominant genes was comparable between 11 pairs of chromosomes. The exception was found in Chr 10, where two times more dominant genes in Chr B10 than that in Chr A10 (Supplementary Fig. S6). These results suggest that polyploidization might have affected the relative expression of homoeologs and likely equally affected the 2 subgenomes except Chr 10.

### Whole-genome duplications and repeat elements

Analysis of synonymous substitutions per synonymous site (*Ks*) suggests the occurrence of 2 whole-genome duplications (WGDs) with median values of 0.4 and 1.8 in *I. sinensis*, and the strong peak ∼1.8 may represent the *K*s values of homeologs of the A and B subgenomes (Fig. 2B and Supplementary Tables S16 and S17). The 2 WGDs are consistent with a previous 1KP transcriptome study that reported 2 WGDs (ISTEɑ and ISTEβ) in *Isoetes tegetiformans* and *Isoetes echinospora* [21], but in contrast to the single WGD found in *I. taiwanensis* [10], which suggests a complex evolutionary history within *Isoetes*.

In *I. sinensis*, repetitive sequences occupy 63.15% of the genome (Supplementary Tables S18 and S19), a much higher proportion than in the genomes of *I. taiwanensis* and *S. moellendorffii* [7, 10]. These repetitive sequences were evenly distributed across the genome of *I. sinensis* (Fig. 1C). Most of the repeats in the *I. sinensis* genome (53.67%) are long terminal repeat (LTR) retrotransposons (Fig. 2C), and more than 30% of LTR insertions in the *I. sinensis* genome occurred recently (Fig. 2D). LTRs in *I. sinensis* are shorter than those in *I. taiwanensis* but longer than those in *S. moellendorffii* (Fig. 2E). We found fewer repeats in each subgenome of *I. sinensis* than that in *I. taiwanensis* but a greater number of LTR/*Copia* and *Gypsy* elements in each chromosome of *I. sinensis* than that in *I. taiwanensis* (Supplementary Table S19), which suggests that LTR copies have likely increased since the divergence of *I. sinensis* and *I. taiwanensis*. Next, we generated a phylogenetic tree to compare the evolution of the LTR retrotransposon *Gypsy* in *I. sinensis, I. taiwanensis*, and *S. moellendorffii*. In addition to transposons similar to those in *S. moellendorffii*, we found that many species-specific transposons had evolved in *I. sinensis* and *I. taiwanensis*, indicating the expansion of *Gypsy* in *Isoetes* (Fig. 2F).

### Transcriptional regulation

**Figure 4:**
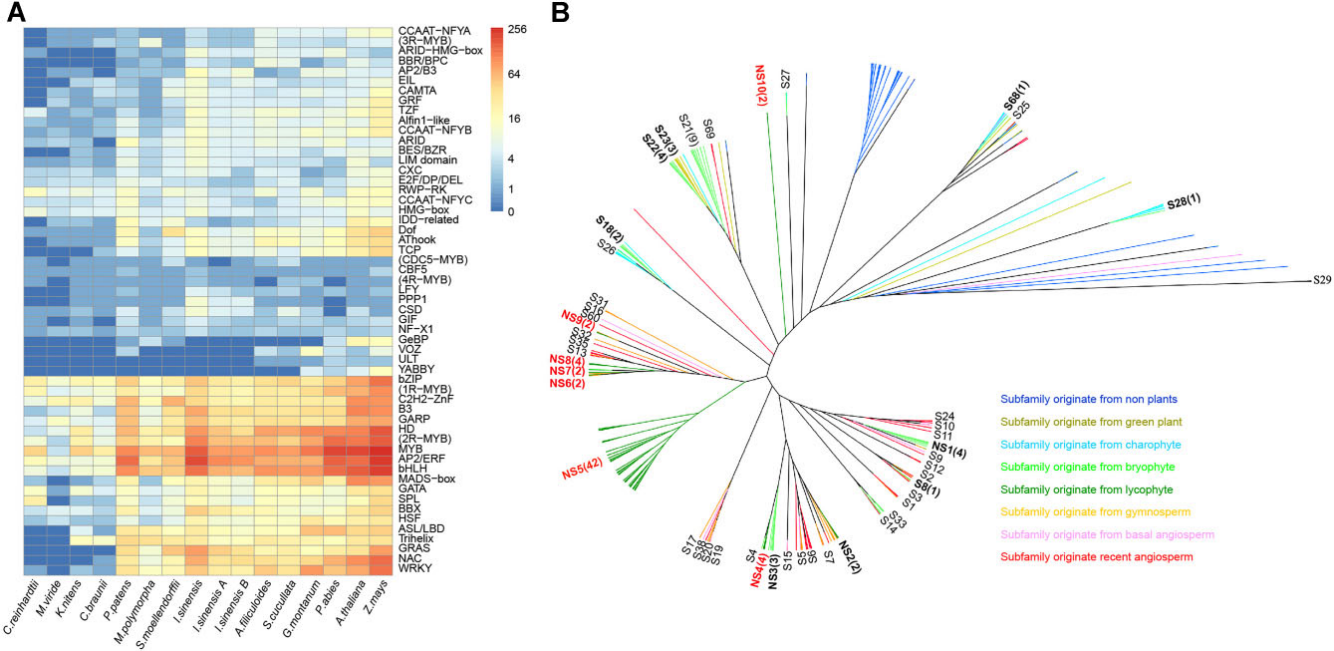
Transcription factors in *I. sinensis*. (A) Heatmap illustrating the numbers of transcription factors in *I. sinensis* compared with 13 evolutionarily representative green algae and land plants. Detailed information is shown in Supplementary Table S23. (B) Neighbor-joining phylogenetic analysis of R2R3-MYB proteins encoded by the genome of *I. sinensis*. The tree includes 90 R2R3-MYB sequences. Bootstrap replicates = 1,000. See Supplementary Dataset S1 showing the detailed tree.

We identified 1,461 sequences that encode transcription factors (TFs) belonging to 52 families in *I. sinensis* (Supplementary Tables S20 and S21). We found that 2.86% of the protein-coding genes in *I. sinensis* encode TFs, relatively fewer than in other land plants but more than in green algae [22]. Genes that encode AP2/ERF, MYB, and bHLH family members accounted for the highest proportion TF-encoding genes in *I. sinensis* (Fig. 4A). When we compared the number of TFs encoded by the diploid A and B subgenomes of *I. sinensis* and other plant genomes, we found that the number of TF-encoding genes increased likely along with organismal complexity, although we did note some exceptions [23]. For example, we found a larger number of genes encoding AP2/ERF, AP2/B3, CSD, and PPP1 in the subgenomes of *I. sinensis* than in the genomes of ferns (Fig. 4A). Interestingly, the gene encoding GeBP (GL1 enhancer binding protein) has been lost from *I. sinensis* but is present in *S. moellendorffii* and bryophytes (Fig. 4A and Supplementary Table S20). Next, we analyzed the evolution of TF families and detected many *I. sinensis–*specific subfamilies, as exemplified by the 2R-MYB family, which performs essential plant stress response functions and represents the second largest TF family in *I. sinensis*. A total of 90 2R-MYB–encoding genes were found in the genome of *I. sinensis*. Phylogenetic analysis suggests that twenty-one 2R-MYB TFs belong to 7 ancient subfamilies, including S28, S21, S22, S23, S18, S8, and S68, which have functions in stress response and development [24, 25]. Among the other nine 2R-MYB TF subfamilies, 6 of them contain only *I. sinensis* sequences, suggesting a species-specific expansion of 2R-MYB TFs within *I. sinensis* (Fig. 4B and Supplementary Dataset S1). We observed that most MYBs within group NS5 were located on a pair of chromosomes of *I. sinensis* (Supplementary Dataset S1), which may suggest their tandem duplication before polyploidization.

### Phytohormones

Although the genome sequences of *I. taiwanensis* and *S. moellendorffii* are available, little is yet known about phytohormone in the Lycophyta. To better understand phytohormone regulation in *I. sinensis*, we investigated both conserved and lost genes that related to synthesis, transport, and signal transduction of phytohormones.

The auxin biosynthesis pathway in flowering plants is conserved and includes one *TAA* (encoding tryptophan aminotransferase in *Arabidopsis*) and 5 *YUCCA* homologs encoding flavin monooxygenase-like enzymes [26]. However, only 1 *YUC* was found in *I. sinensis*. There is no TAA-encoding gene in *I. sinensis*, although its paralog *TAR* was detected (Supplementary Fig. S7 and Supplementary Datasets S2–S6). The *I. sinensis* genome possesses the auxin signal transduction components *AUX1* and a small number of *SAUR* genes that are not found in early land plants, suggesting that these genes could have evolved in the lycophytes. Interestingly, *I. sinensis* does not carry the *IAA1* and *GH* genes that are present in seed plants, suggesting a stepwise acquisition of auxin signaling during land plant evolution.

Abscisic acid (ABA) is generated under environmental stress and leads to a series of reactions that allow plants to adapt to adverse conditions [27]. Almost all the genes involved in ABA biosynthesis, except *XD* and *AAO*, are present in *I. sinensis* (Supplementary Fig. S7 and Supplementary Datasets S7–S11). The PYL receptor mediates the ABA response in cells via a complex between ABA and PYL that inhibits a PP2C (group A phosphatase 2C) to activate SnRK2, a SNF1-related protein kinase 2. While genes encoding PP2C and SnRK2 exist in *I. sinensis*, only 1 homolog encoding the PYL receptor (PYL5) was found. Genes encoding downstream TFs, such as AREB/ABFs, which are involved in desiccation tolerance, were also detected in *I. sinensis*. In addition, almost all of the genes involved in the cytokinin/ethylene-controlled signal transduction pathways exist in *I. sinensis*, except for those encoding the receptor CKR in the cytokinin signaling pathway, and 1-aminocyclopropane-1-carboxylate oxidase, which exists only in seed plants [28] (Supplementary Fig. S7 and Supplementary Datasets S12–S19). Jasmonic acid (JA) and gibberellin (GA) signaling pathways play important roles in response to biotic stress [29]. We identified almost all of the genes that constitute the JA and GA pathways in *I. sinensis* (Supplementary Fig. S7 and Supplementary Datasets S20–S24). Like other plants, *I. sinensis* contains genes that encode JA biosynthetic enzymes such as LOX, AOC, AOS, JAR1, and OPR3, as well as genes encoding COI1 receptor and MYC transcription factor orthologs. Among the few exceptions are genes encoding GA synthesis and transport functions such as *PIL* and *GA3OX* that are present in the genomes of green algae and early land plants but have been lost from the *I. sinensis* genome. Taken together, the presence of these orthologs suggests nearly intact ABA, cytokinin, ethylene, JA, and GA signaling pathways in *I. sinensis*.

On the other hand, we found a paucity of genes involved in the strigolactone (SL) and salicylic acid (SA) signaling pathways in *I. sinensis* (Supplementary Fig. S7 and Supplementary Datasets S25–S28). For example, apart from only 1 *MAX2* gene, *I. sinensis* has lost many other genes with functions in SL signaling. Furthermore, only a few components of the BR pathway (BRI1-like and DET2) can be detected in *I. sinensis* (Supplementary Datasets S29–S32). As for SA signaling, we detected genes encoding CUL3 but none encoding NPR or BOP in *I. sinensis*.

We further compared the genes involved in phytohormone between *I. sinensis* and *I. taiwanensis*. Except for a small number of genes found only in *I. sinensis*, such as *GA2OX* and *AOC3*, and the genes found only in *I. taiwanensis*, such as *BAK1, ACO4, ACS2, ACS4*, and *JAZ*, most of genes are conserved with slight copy number variation between these 2 *Isoetes* species (Supplementary Table S22). This result might suggest a conserved phytohormone regulation between *I. sinensis* and *I. taiwanensis*.

### CAM photosynthesis

CAM is a metabolic pathway that concentrates CO_2_ in plant cells to help some land plant species avoid drought and aquatic plant species avoid CO_2_ limitation [30]. This adaptation is widespread in *Isoetes*, wherein carbon accumulates as malic acid during the night and enters the Calvin cycle during the day to improve CO_2_ utilization [15]. Recently, the evolutionary path of CAM in *I. taiwanensis* has been described [10]. As does *I. taiwanensis, I. sinensis* possesses genes encoding both bacterial- and plant-type phosphoenolpyruvate carboxylase (PEPC) (Supplementary Fig. S8A, B), a key enzyme in CAM and C4 photosynthesis in various plant species*. I. sinensis* expresses the bacterial-type *PEPC* at a low level and expresses the plant-type *PEPC* at a high level in roots, shoots, and sporangia, in contrast to the higher expression of bacterial-type *PEPC* than plant-type *PEPC* during development in *I. taiwanensis* (Supplementary Fig. S8C). In addition, *I. sinensis* lacks a gene encoding phosphoenolpyruvate carboxykinase (Supplementary Fig. S7B), which participates in 1 of 2 important decarboxylation pathways in *I. taiwanensis*, suggesting differences in mechanisms of CAM across aquatic plants.

### Stomatal development

Some aquatic plant species do not develop stomata or have nonfunctional stomata occluded by wax [31]. Functional stomata are important for *Isoetes* to adapt to amphibiotic conditions. However, we found that some key genes for stomata development, such as *SPEECHLESS* (*SPCH*), *MYB88*, and *MUTE* [32, 33], are not present in the genomes of either *I. sinensis* or *I. taiwanensis* (Supplementary Fig. S9), suggesting specialized stomatal regulation in *Isoetes. I. taiwanensis* leaves have relatively fewer stomata than do those of *I. sinensis* [34]. Thus, we compared the *I. sinensis* and *I. taiwanensis* genes likely involved in stomatal development or regulation [35] and identified 45 of these genes in the *I. sinensis* genome and 39 in the *I. taiwanensis* genome, from a total 75 genes that could have been involved in these processes (Supplementary Fig. S9). The absence of some putative stomatal development genes from each genome might have contributed to the differences in stomatal number and regulation of stomatal development between *I. sinensis* and *I. taiwanensis*.

### Adaptation to environmental stresses

Land plants are often threatened by adverse abiotic environmental conditions that limit their growth and development. By comparing the genomes of *I. sinensis* and *I. taiwanensis*, as well as transcriptomes of 19 lycophytes from the 1KP project [36], we analyzed the genetic basis of lycophyte adaptation to environmental stresses.

### Cold sensing and response

**Figure 5:**
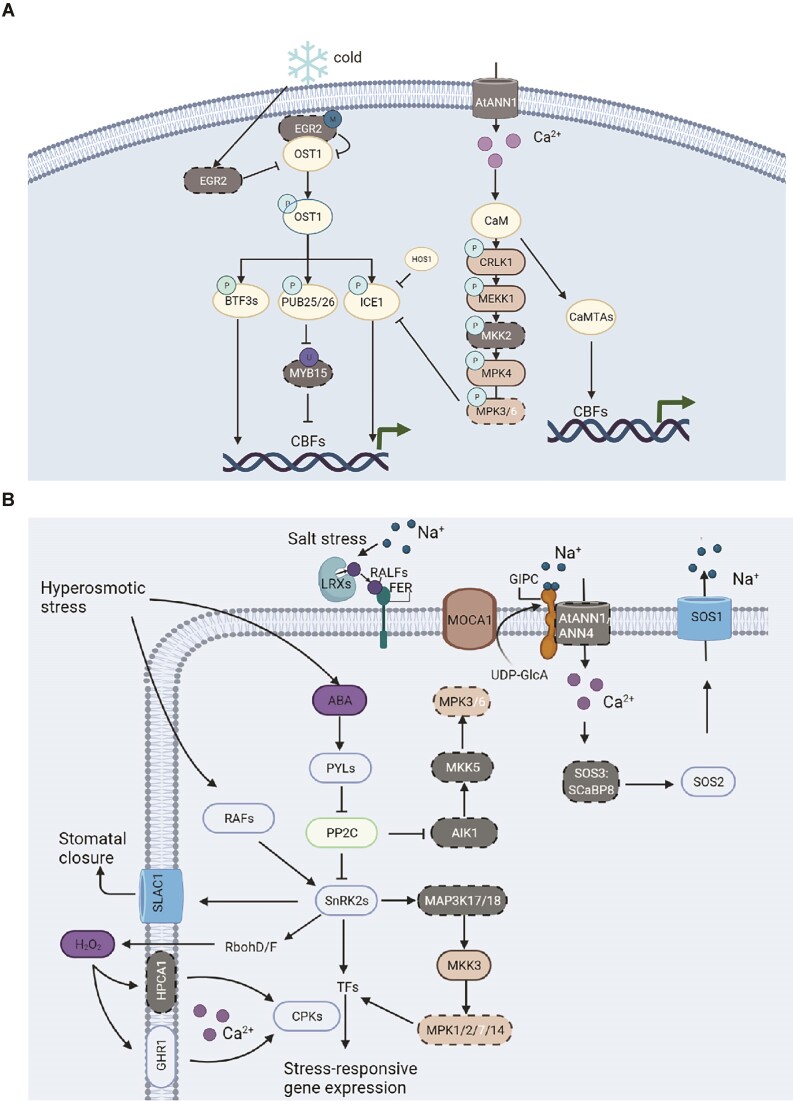
Abiotic stress responses in lycophytes. (A) Diagram showing the pathway and genes involved in cold sensing and response in plants. The key genes *EGR2, MYB15*, and *ANN1* were not detected in *I. sinensis* and most lycophytes. (B) Diagram showing the pathway and genes involved in salinity and drought stress sensing and signaling. The key genes *MKK5, AIK1, MAP3K17/K18, HPCA1, ANN1, ANN4, SOS3*, and SCaBP8 were not detected in *I. sinensis* and most lycophytes. Dotted lines and white text indicate the absence of genes.

Our comparative analysis did not detect many of the key genes responsible for cold sensing or response in lycophytes (Supplementary Fig. S10 and Supplementary Datasets S33–S45). First, as a temperature stress sensor, Ca^2+^ can induce temperature-responsive gene expression [37, 38]. Annexin 1 (ANN1) is the essential Ca^2+^ osmotic transporter that mediates cold-triggered Ca^2+^ influx and freezing resistance [37]. However, *ANN1* is absent in *I. sinensis* and most of the other lycophytes (Fig. 5A and Supplementary Fig. S9A). Second, EARLY FLOWERING 3 (ELF3), ELF4, and LUX ARRYTHMO (LUX) can form an evening complex to perceive temperature changes and regulate plant growth by directly repressing the expression of *PIF4* under cold temperatures [39]. *ELF4* was also not detected in all of the lycophytes (Fig. 5A and Supplementary Fig. S10A). Third, cold stress activates the transcription of TF-encoding genes, including those encoding C-repeat binding factors (CBFs) [40]. OST1 is a positive regulator in CBF-dependent cold signaling, while EGR2 phosphatase is a negative regulator of plant-freezing tolerance via inhibition of OST1 kinase activity, which thereby reduces the expression of CBFs during cold stress responses. In addition, the negative transcriptional regulator of CBFs, MYB15, is degraded during cold stress. We did not detect *EGR2* and *MYB15* in all of the lycophytes (Fig. 5A and Supplementary Fig. S10A). The absence of these homologs suggests a diversification between lycophytes and model plant *Arabidopsis* in the cold-sensing and response pathway.

### Drought and salinity sensing and response

Drought stress stimulates local production and accumulation of the hormone ABA in plant organs, which is an important way to improve water efficiency and drought resistance in plants [41]. ABA signaling is mediated by the ABA receptors PYR, PYL, and RCAR and by the PP2Cs and SnRK2s [42, 43] that interact with them. The genes that encode these proteins are present in lycophytes (Supplementary Fig. S11A and Supplementary Datasets S46–S66). ABA-activated SnRK2s are phosphorylated and phosphorylate the plasma membrane NADPH oxidase RbohD/F that generates O^2−^ and subsequently H_2_O_2_ [38]. Leucine-rich repeat receptor kinases HPCA1 and GHR1 then sense this extracellular H_2_O_2_ and activate Ca^2+^ signaling via Ca^2+^ channels [44, 45]. In *Arabidopsis*, H_2_O_2_‐ and ABA‐induced stomatal closure is impaired in the *hpca1* mutant [44, 45]. However, the absence of the *HPCA1* from *I. sinensis* and many other lycophytes might adversely affect the drought resistance of these species (Fig. 5B and Supplementary Fig. S11A).

Salinity is another important environmental factor inducing abiotic stress in plants and can result in hyperosmotic stress in plant cells [46]. In *Arabidopsis*, the salt overly sensitive (SOS) pathway comprises the SOS3 and SCaBP8 calcium sensors, the SOS2 protein kinase, and the SOS1 plasma membrane Na^+^/H^+^ antiporter. When an *Arabidopsis* plant experiences salt stress, SOS3 and ScaBP8 sense the calcium signal, interact with SOS2, and activate its kinase activity, which then activates the reverse transport activity of SOS1 [47, 48]. Calcium signals in this system in *Arabidopsis* are mediated by the Ca^2+^-permeable transporters AtANN1 and AtANN4 [49, 50]. The absence from the lycophytes of genes encoding the Ca^2+^ transporters ANN1 and ANN4 and those encoding the downstream sensor SOS3 and ScaBP8 might thus limit the adaptability of lycophytes to salt stress (Fig. 5B and Supplementary Fig. S11B).

On the other hand, we also observed some conserved pathways between lycophytes and angiosperms (Supplementary Figs. S11A–C) that might contribute to the adaption to drought and salinity in lycophytes.

### Cadmium stress

Water pollution and eutrophication result in heavy metal stress that critically endangers *I. sinensis* [51]. Cadmium (Cd) is a heavy metal with high toxicity to plants [52]. Uptake of cadmium occurs in root cells, mainly mediated by NRAMP5, and its root-to-shoot transport is completed by HMA2 and HMA4 [53]. HMA3 mediates an effective detoxification mechanism that limits Cd transport to shoots by accumulating Cd in vacuoles [53]. *Cadmium accumulation in leaf 1* (*CAL1*) encodes a defensin-like protein that can chelate cytosolic Cd and promotes secretion of Cd into intercellular spaces such as the cell wall apoplast and xylem to decrease the concentration of Cd in the cytosol during transport of Cd within the plant [54]. Homologs of *HMA3* and *CAL1* are not present in the *I. sinensis* and many lycophytes (Supplementary Fig. S12 and Supplementary Datasets S67–S68), which could limit the ability of lycophytes to control the transport and accumulation of Cd.

The activities of phytohormones are important for plants to adapt to heavy metal stress [55]. For example, cadmium enhances the activity of *Gretchen Hagen 3* (*GH3*), a gene present in algae and land plants that reduces the level of active indole-3-acetic acid (IAA) by esterifying it with an amino acid, resulting in increased lignin synthesis and peroxidase activity during plant defenses to heavy metal toxicity [56]. Treatment of plants with Cd resulted in the accumulation of *ETR2* and *ERF1*, which encode ethylene receptors, whereas the abundance of transcripts for brassinosteroid (BR)–related genes such as DWARF and BR6ox, decreased, suggesting that Cd-mediated BR biosynthesis feedback is inhibited when the BR contents increase [57]. BR homeostasis also requires the transcription factor BZR1 [58]. However, the homologs of all genes relevant to heavy metal response mentioned above are absent of *I. sinensis* and those of most lycophytes (Fig. 5C and Supplementary Fig. S12), which could adversely affect their ability to adapt to Cd stress.

## Conclusion

Here, we present a high-quality assembly and annotation of the *I. sinensis* genome, which represents the first sequenced tetraploid genome with chromosome-level assembly for a seed-free plant. Comparative analysis between *I. sinensis* and its close related diploid species *I. taiwanensis* revealed the features of genome and polyploidy in lycophytes. We found the differences in CAM and stomatal regulation between *I. sinensis* and *I. taiwanensis*. Comparison of the genome of *I. sinensis* with genomes representing the evolutionary lineages of green algae and land plants has revealed that *I. sinensis* possesses some common genetic tools, such as those associated with transcriptional regulation and involved in ABA, cytokinin, ethylene, JA, and GA signaling pathways. On the other hand, we have also shown that some key genes involved in important genetic pathways, including strigolactone, salicylic acid, and stress responses (cold, drought, salinity, and cadmium), have been lost or not detected in the *I. sinensis* and many lycophytes. These findings are crucial for the understanding of lycophyte development and their adaptation to adverse abiotic environmental conditions.

## Methods

### Plant materials and genome sequencing

*I. sinensis* shoot materials were harvested from Yangdongcun, Beilun District, Ningbo, Zhejiang Province of China. DNA was extracted using a modified cetyltrimethylammonium bromide procedure. DNA concentrations and purity were evaluated by NanoDrop and its quality analyzed by agarose gel electrophoresis. Paired-end libraries with a 350-bp inserts were prepared by following the Illumina protocols and then sequenced in PE150 mode on the Illumina HiSeq X Ten platform (RRID:SCR_016385). A total of 176.46 Gb paired-end reads were obtained for genome survey. The read mapping rate of the Illumina sequencing was 98.58%, covering 99.95% of the *I. sinensis* genome. For the PacBio Sequel analysis, the libraries for single-molecule real-time (SMRT) genome sequencing were prepared according to the manufacturer's protocol for the sequencing platform and then sequenced with SMRT sequencing at 43.52× coverage using 4 cells. A total of 97.01 Gb reads were obtained for the genome assembly. High-throughput chromosome conformation capture (Hi-C) sequencing libraries were produced as follows: nuclei were isolated and fixed with the cross-linking agent paraformaldehyde and then the cross-linked DNA was treated with restriction enzymes. Biotin was then added to label the ends of oligonucleotides during terminal repair. Adjacent DNA fragments were joined using nuclease ligases. Protein was digested with a protease to dissociate the protein from the DNA. Then the genomic DNA was extracted and randomly sheared into 350-bp fragments using a Covaris crusher. The library was prepared according to manufacturer's instructions (Illumina) and sequenced on a HiSeq X Ten DNA system to obtain 150-bp paired-end sequences.

### RNA-seq and full-length transcriptome sequencing

RNAs from roots, shoots, and sporangia of *I. sinensis* were extracted using a RNeasy Plus Mini Kit (Qiagen). After that, rRNA was removed from total RNA samples using the RiBO-Zero Kit (Illumina). The isolated mRNA (∼1% of total RNA) was used as template to synthesize complementary DNA (cDNA), then the cDNA was sheared into small fragments. Paired-end libraries were prepared from various tissues by following the Illumina protocols and sequenced with PE150 mode on the Illumina HiSeq X Ten platform. Pooled samples from the roots, shoots, and sporangia pooling sample were used for the PacBio Sequel analysis. The libraries for SMRT genome sequencing were prepared according to the manufacturer's protocol for the sequencing platform and then sequenced on a PacBio Sequel II with SMRT sequencing.

### Genome assembly and annotation

Before *de novo* genome assembly, Illumina short reads were used for preliminary evaluation of the genome size, heterozygosity, and repeat sequence proportions by *k*-mer analysis. After data filtering and quality control, the short reads were first assembled using SOAPdenovo (RRID:SCR_010752) software to generate contigs. These contigs were further used to construct scaffolds according to their pair-end relationships. The quality value (QV) score generated from Merqury (RRID:SCR_022964) was 46.1448, and the corresponding error rate was 2.4295e-05.

*De novo* genome assembly of the PacBio long reads from *I. sinensis* genomes was performed using Hifiasm (RRID:SCR_021069) [59]. The primary contigs were polished by aligning PacBio SMRT reads using the NextPolish software with the default parameters [60]. The consensus sequences for scaffolds were further polished based on Illumina paired-end reads using Pilon (RRID:SCR_014731). The total length of this assembly was 2,131.51 Mb, with a contig N50 up to 2,673 kb.

For the chromosome-level assembly, the clean Hi-C sequencing data were mapped to the draft genome using the Burrows–Wheeler Aligner (BWA) [61], and the repeated and unmatched data were removed by SAMtools (RRID:SCR_005227) [62]. Only unique valid paired-end reads were retained for subsequent chromosome-level assembly. Draft genome scaffolds were clustered according to interactions using the ALLHiC software (RRID:SCR_022750) [63]. Finally, about 90.10% sequences were grouped into 22 pseudochromosomes. Transcripts were aligned using Bowtie 2 (v.2.3.4.1) [64] software with the parameters (–no-mixed –no-discordant). The transcriptome was then quantified using RSEM (RRID:SCR_000262) (v.1.3.1) [65] with default parameters. After RNA-seq analysis, we found a total of 43,154 expressed genes accounting for 75.3% of the total predicted genes, which proved the high reliability of our genome annotation.

### Genome completeness assessment

Genome completeness was evaluated using BUSCO (RRID:SCR_015008) [66] and CEGMA (RRID:SCR_015055) [67] analyses. BUSCO detected 84.7% complete and 3.2% fragmented BUSCO gene models in the assembly. CEGMA results suggested that 98.39% of core eukaryotic genes have been assembled. Small fragment library reads were selected and aligned to the assembled genome using BWA software (RRID:SCR_010910). Finally, 98.58% of small fragment reads mapped to the *I. sinensis* genome. LAI was evaluated by LTR_retriever (RRID:SCR_017623) (v2.9.0) [68].

### Repeat sequence annotation

The repetitive sequences in *I. sinensis* were estimated by *de novo* strategies using RepeatModeler (RRID:SCR_015027), RepeatScout (RRID:SCR_014653), LTR_FINDER (RRID:SCR_015247) [69], MITE-Hunter (RRID:SCR_020946) [70], and PILER-DF [71]. A homology-based search for repeat sequences was carried out using RepeatMasker (RRID:SCR_012954) [72] to search Repbase (RRID:SCR_021169).

LTRs were identified using LTR_FINDER [69] and LTRharvest (RRID:SCR_018970) [73], the results of which were then integrated with LTR_retriever [68] to build an accurate, nonredundant species-specific LTR database. Subsequently, we used homology-based prediction methods to annotate, filter out false positives, and annotate comprehensive and accurate species LTR sequences, including intact LTRs, solo LTRs, and LTR-related sequences.

### LncRNA sequencing and analysis

Total RNA was extracted from each *I. sinensis* sample using the RNeasy Plus Mini Kit, and rRNA removal was performed using a RiBO-Zero Kit. Isolated RNA was used for cDNA library construction, using the dUTP method [74]. These libraries were sequenced on an Illumina HiSeq X Ten platform. The purity, concentration, and integrity of RNA were checked using the agarose gel electrophoresis, the Qubit 2.0 Fluorometer, and the Agilent 4150 TapeStation, respectively. After trimming adapters and filtering out low-quality reads, a total of 14.02 Gb clean reads were generated. The transcriptome was mapped to the reference genome using TopHat2 [75]. Transcripts greater than 200 bp in length and containing at least 2 exons were considered lncRNA candidates. Four computational approaches, including CPC [76], CNCI [77], Pfam (RRID:SCR_004726), and PhyloCSF [78], were combined to evaluate the protein-coding capability of the lncRNA candidates.

### Small RNA sequencing and analysis

Small RNA libraries for *I. sinensis* were constructed using a Small RNA Sample Pre Kit for Illumina HiSeq sequencing. Raw reads were filtered by removing 3′-adapters, primers, and low-quality sequences using Cutadapt (RRID:SCR_011841) v1.9.1. Clean reads of 18 to 30 nucleotides were screened for subsequent analysis. The clean reads were mapped to Silva (RRID:SCR_006423), GtRNAdb database (RRID:SCR_006939), Rfam (RRID:SCR_007891), and Repbase (RRID:SCR_021169) to remove rRNAs, tRNAs, snRNAs, snoRNAs, and other ncRNAs and repeats. The remaining reads were compared with reference miRNAs in the miRbase (RRID:SCR_003152) to annotate miRNAs. These reads were then mapped to the genome using Bowtie 2 (RRID:SCR_016368) [64].

### Predictions of genes and noncoding RNAs

Gene annotation was performed by combining evidence drawn from *ab initio* prediction, homology-based gene prediction, and transcript evidence from RNA-seq data for *I. sinensis*. The *ab initio* gene prediction was conducted using 2 *ab initio* gene predictors, Augustus (RRID:SCR_008417) [79] and Genscan (RRID:SCR_013362), with default parameters. Orthologous protein sequences were then aligned to the genome assembly using GeneWise (RRID:SCR_015054) [80]. In addition, the transcriptome data of the whole plant were used to predict genes using PASA [81]. Evidence Modeler [82] was used to generate a single high-confidence gene model set. Finally, 57,303 protein-coding genes were predicted for *I. sinensis* and all protein-coding genes were annotated to the public protein databases at KEGG (RRID:SCR_012773), SwissProt (RRID:SCR_021164), TrEMBL, and InterProScan v5.11–51.0 (RRID:SCR_005829), with an E-value cutoff of 1e^−5^. Pseudogenes were detected by exonerate (RRID:SCR_016088) (v.2.4) using the protein data of *Salvinia cucullata, Azolla filiculoides* [8], and *I. sinensis*.

We used 2 strategies to annotate noncoding RNAs, including *de novo* prediction and direct RNA sequencing of small RNAs and lncRNAs. rRNA fragments were identified using BLAST against rRNA sequences of reference species in the Pfam database. tRNAs were identified using tRNAscn-SE. Additionally, other types of noncoding RNA, including miRNAs and snRNAs, were identified at the Rfam database using INFERNAL (RRID:SCR_011809) [83].

### Identification of WGD

In order to search for genome-wide duplications in the *I. sinensis* genome, we used the Whole-Genome Duplication Integrated analysis tool for WGD and intragenomic collinearity detection as well as *Ks* estimation and peak fitting [84]. The WGD analyses were performed using all paralogous gene pairs.

### Gene family and phylogenomic analysis

Gene families for the 19 species were analyzed and clustered using OrthoMCL (RRID:SCR_007839) (v. 2.0.9) with default parameters [85]. The 19 species, including *A. thaliana, Vitis vinifera, Z. mays, Oryza sativa* [86], *Physcomitrella patens* [87], *Marchantia polymorpha* [26], *A. filiculoides, S. cucullata, Amborella trichopoda* [88], *Cycas panzhihuaensis* [89], *Picea abies* [90], *Gnetum montanum* [91], *S. moellendorffii* [7], *I. sinensis, I. taiwanensis* [10], *Mesostigma viride* [22], *Chlamydomonas reinhardtii* [92], *Klebsormidium nitens* [93], and *Chara braunii* [94], were used in the analysis. Gene families were clustered using OrthoMCL software with default parameters. During OrthoMCL gene family clustering, we defined single-copy gene families as genes existing as 1 copy in selected species and obtained a total of 66 single-copy gene families for further analysis. These single-copy genes were aligned using software MAFFT (RRID:SCR_011811) (v.7.490), and then ProTest (v.3.4.2) was used to find the best model of amino acid replacement in the single-copy gene alignments. Before phylogeny construction, Gblocks (RRID:SCR_015945) (v.0.91b) [95] (-b5 = h) was used to remove gap regions of the multiple sequence alignments. A phylogenetic tree was constructed using RAxML (RRID:SCR_006086) (v.8.2.12) [96] with the ML algorithm and 1,000 bootstrap replicates.

Based on a calibration of divergence times using *C. reinhardtii* and *G. montanum* from TimeTree (RRID:SCR_021162), the divergence times for the inferred species tree were calculated using r8s (RRID:SCR_021161) (v.1.81) [97]. Gene families were used to calculate the expansion or contraction of the gene families in each lineage using CAFE (RRID:SCR_005983) (v.4.2.1) with *P* < 0.05 [98]. *P* values were used to estimate the likelihood of the observed gene family sizes given average rates of gain and loss and were also used to determine expansion or contraction for individual gene families in each node.

### Phylogenetic analysis of TF, phytohormone, CAM, and stress response−related genes

To identify TF, phytohormone, CAM and stress response related genes, we performed comparative genomic analysis of the genomes of *I. sinensis* and 13 representative plants or algae (including *A. thaliana, V. vinifera, Z. mays, P. patens, M. polymorpha, A. filiculoides, S. cucullata, P. abies, G. montanum, S. moellendorffii, I. taiwanensis, M. viride*, and *C. reinhardtii*) and transcriptomes of the other 19 lycophytes from the 1KP project [36]. BLASTP search (*P* < 1e-5) was performed using well-studied proteins (mostly from *A. thaliana*) as queries to identify the homolog genes in *I. sinensis*. The redundant sequences were deleted, and subsequently, candidates were examined for the conserved domain(s) of respective gene families using SMART (RRID:SCR_005026). Amino acid sequences of our target genes were aligned using Muscle. The alignments were then manually inspected using MEGA 7. MEGA 7 was run with 1,000 bootstrap replicates to generate the neighbor-joining phylogenetic trees [99].

### Comparison of relative expression of homoeologs in the pairs of chromosomes of *I. sinensis*

We adopted the method used to analyze homoeolog expression in *Brassica juncea* [20] and focused on genes with 1:1 homoeologs between pairs of chromosomes of *I. sinensis*. DEG pairs with fold change >2 were defined as dominant gene pairs. The dominant genes were defined as the genes with higher expression in dominant gene pairs, and the lower ones within dominant gene pairs were defined as subordinate genes. The rest of the genes with 1:1 homoeologs were defined as neutral genes.

## Supplementary Material

giad079_GIGA-D-23-00116_Original_Submission

giad079_GIGA-D-23-00116_Revision_1

giad079_GIGA-D-23-00116_Revision_2

giad079_Response_to_Reviewer_Comments_Original_Submission

giad079_Response_to_Reviewer_Comments_Revision_1

giad079_Reviewer_1_Report_Original_Submission Dongya Wu -- 7/3/2023 Reviewed

giad079_Reviewer_1_Report_Revision_1 Dongya Wu -- 8/17/2023 Reviewed

giad079_Reviewer_2_Report_Original_Submission Yongzhi Yang, Ph.D. -- 7/5/2023 Reviewed

giad079_Reviewer_2_Report_Revision_1 Yongzhi Yang, Ph.D. -- 8/21/2023 Reviewed

giad079_Supplemental_Files

## Data Availability

The raw data of genome sequencing for *I. sinensis* have been deposited in the NCBI SRA with the following accession numbers: SRR17422691 (Illumina); SRR17422560, SRR17422559, SRR17422562, and SRR17422561 (Hi-C); and SRR17640823, SRR17640824, SRR17640825, and SRR17640826 (PacBio). The genome assembly and annotation have been deposited in the China National GeneBank DataBase with accession number CNA0072254. The raw data of RNA sequencing, including lncRNA sequencing, small RNA sequencing, mRNA‐seq, and full‐length transcriptome sequencing of different tissues, have been deposited in the NCBI Gene Expression Omnibus (GEO) with accession number GSE198197. All additional supporting data are available in the *GigaScience* GigaDB database [100].

## Additional Files

**Supplementary Fig. S1**. Hi-C links and length of gene and CDS.

**Supplementary Fig. S2**. High similarity between 2 subgenomes of *I. sinensis*.

**Supplementary Fig. S3**. *k*-mer and SubPhaser analyses were not able to separate the subgenomes of *I. sinensis*.

**Supplementary Fig. S4**. Collinearity analysis of the *I. sinensis* genome.

**Supplementary Fig. S5**. KEGG analysis of expansion and contraction gene families during *I. sinensis* evolution.

**Supplementary Fig. S6**. Histograms of expression of 1:1 homoeologous genes between pairs of chromosomes among *I. sinensis* sporangia, shoot, and root tissues.

**Supplementary Fig. S7**. Phytohormone biosynthesis and signaling pathways in *I. sinensis*.

**Supplementary Fig. S8**. CAM-related genes in *I. sinensis*.

**Supplementary Fig. S9**. Stomatal regulation-related genes in *I. sinensis* and *I. taiwanensis*.

**Supplementary Fig. S10**. Temperature stress–related genes in *I. sinensis* and other lycophytes.

**Supplementary Fig. S11**. Salinity and drought stress–related genes in *I. sinensis* and other lycophytes.

**Supplementary Fig. S12**. Cadmium stress–related genes in *I. sinensis* and other lycophyte species.

**Supplementary Table S1**. Statistics of the *I. sinensis* genome sequencing.

**Supplementary Table S2**. Statistics of the *I. sinensis* genome assembly.

**Supplementary Table S3**. Statistics of chromosome length of the *I. sinensis* genome.

**Supplementary Table S4**. Statistics of the *I. sinensis* RNA-seq libraries.

**Supplementary Table S5**. Genome completeness assessment based on BUSCO for the *I. sinensis* genome assembly.

**Supplementary Table S6**. Summary of annotated noncoding RNA genes in *I. sinensis*.

**Supplementary Table S7**. List of annotated tRNA genes in *I. sinensis*.

**Supplementary Table S8**. List of annotated rRNA genes in *I. sinensis*.

**Supplementary Table S9**. List of annotated snRNA genes in *I. sinensis*.

**Supplementary Table S10**. List of annotated snoRNA genes in *I. sinensis*.

**Supplementary Table S11**. List of annotated miRNA genes in *I. sinensis*.

**Supplementary Table S12**. List of annotated lncRNA genes in *I. sinensis*.

**Supplementary Table S13**. List of annotated pseudogenes in *I. sinensis*.

**Supplementary Table S14**. Genome-wide comparisons and gene coverage analyses of *I. sinensis* and *I. taiwanensis*.

**Supplementary Table S15**. Specific gene pair information for the 2 subgenomes of *I. sinensis*.

**Supplementary Table S16**. Collinearity analysis between the proposed homeologs in *I. sinensis*.

**Supplementary Table S17**. *Ks* values of blocks in *I. sinensis*.

**Supplementary Table S18**. Summary of repeat distribution in *I. sinensis* and relative species.

**Supplementary Table S19**. Summary of repeats in *I. sinensis* and relative species.

**Supplementary Table S20**. Numbers of transcription factor genes in representative land plants and green algae.

**Supplementary Table S21**. IDs and sequences of transcription factor genes in *I. sinensis*.

**Supplementary Table S22**. IDs of phytohormones genes in *I. sinensis* and *I. taiwanensis*.

**Supplementary Datasets S1–S68**. Phylogenetic relationships of proteins from *I. sinensis* and other evolutionarily representative species.

## Abbreviations

ABA: abscisic acid; BLAST: Basic Local Alignment Search Tool; BR: brassinosteroid; BUSCO: Benchmarking Universal Single-Copy Orthologs; BWA: Burrows–Wheeler Aligner; CAM: Crassulacean acid metabolism; Cd: Cadmium; cDNA: complementary DNA; CEGMA: Core Eukaryote Gene Mapping Approach; GA: gibberellin; JA: jasmonic acid; KEGG: Kyoto Encyclopedia of Genes and Genomes; LAI: LTR Assembly Index; lncRNA: long noncoding RNA; LTR: long terminal repeat; miRNA: microRNA; ML: maximum likelihood; mRNA: messenger RNA; ncRNA: noncoding RNA; PEPC: phosphoenolpyruvate carboxylase; RNA-seq: RNA sequencing; rRNA: ribosomal RNA: SA: salicylic acid; SL: strigolactone; SMRT: single-molecule real time; snoRNA: small nucleolar RNA; snRNA: small nuclear RNA; SOS: salt overly sensitive; TF: transcription factor; tRNA: transfer RNA; WGD: whole-genome duplication.
