# Supplementary material for: Chromosome-level reference genome of tetraploid Isoetes sinensis provides insights into evolution and adaption of lycophytes
Source: Gigascience. 2023 Sep 30;12:giad079. doi: 10.1093/gigascience/giad079 (PMC10541799; doi:10.1093/gigascience/giad079)
Supplement: giad079_GIGA-D-23-00116_Revision_2 [file giad079_giga-d-23-00116_revision_2.pdf]

GigaScience

Chromosome-level reference genome of tetraploid Isoetes sinensis provides insights into evolution and adaption of lycophytes

--Manuscript Draft--

Manuscript Number:	GIGA-D-23-00116R2
Full Title:	Chromosome-level reference genome of tetraploid Isoetes sinensis provides insights into evolution and adaption of lycophytes
Article Type:	Data Note
Funding Information:	The Agricultural Science and Technology Innovation Program — Prof. Zhe Liang<br>Central Public-interest Scientific Institution Basal Research Fund — Prof. Zhe Liang<br>Beijing Laboratory of Urban and Rural Eco-environment Project — Mr. Jinteng Cui
Abstract:	Background<br>The Lycophyta species are the extant taxa most similar to early vascular plants that were once abundant on Earth. However, their distribution has greatly diminished. So far, the absence of chromosome level assembled lycophyte genomes, has hindered our understanding of evolution and environmental adaption of lycophytes.<br>Findings<br>We present the reference genome of the tetraploid aquatic quillwort, Isoetes sinensis, a lycophyte. This genome represents the first chromosome-level assembled genome of a tetraploid seed-free plant. Comparison of genomes between I. sinensis and the I. taiwanensis revealed conserved and different genomic features between diploid and polyploid lycophytes. Comparison of the I. sinensis genome with those of other species representing the evolutionary lineages of green plants revealed the inherited genetic tools for transcriptional regulation and most phytohormones in I. sinensis. The presence and absence of key genes related to development and stress responses provides insights into environmental adaption of lycophytes.<br>Conclusions<br>The high-quality reference genome and genomic analysis presented in this study are crucial for future genetic and the environmental studies of not only I. sinensis but also other lycophytes.
Corresponding Author:	Zhe Liang<br>Chinese Academy of Agricultural Sciences<br>Beijing, CHINA
Corresponding Author Secondary Information:	
Corresponding Author's Institution:	Chinese Academy of Agricultural Sciences
Corresponding Author's Secondary Institution:	
First Author:	Jinteng Cui
First Author Secondary Information:	
Order of Authors:	Jinteng Cui<br>Yunke Zhu<br>Hai Du<br>Zhenhua Liu<br>Siqian Shen<br>Tongxin Wang<br>Wenwen Cui

Powered by Editorial Manager® and ProduXion Manager® from Aries Systems Corporation

	Rong Zhang
	Sanjie Jiang
	Yanmin Wu
	Xiaofeng Gu
	Hao Yu
	Zhe Liang
**Order of Authors Secondary Information:**	
**Response to Reviewers:**	Manuscript No: GIGA-D-23-00116R1<br>Manuscript Title: Chromosome-level reference genome of tetraploid Isoetes sinensis provides insights into evolution and adaption of lycophytes<br><br>Response to Reviewers<br><br>We would like to thank the Editor and two reviewers for the time committed in reviewing this revised manuscript, and for all the detailed suggestions on improving this manuscript. We have further revised the manuscript to address the reviewers' concerns as follows. All changes were highlighted in the revised manuscript.<br><br>Reviewer #1:<br>The authors have addressed most of my questions.<br>Minor concerns remain:<br>>Reviewer:<br>1. Page 6 Synteny between seed free and seed plant genomes: I do not think these two blocks could be considered as synteny blocks between I. sinensis and A. thaliana and and Z. mays (FigS4C). The genes in A. thaliana are completely out of order and three out of the four genes (evm.model.Chr1.1789, 1794, 1797) in I. sinensis are tandemly duplicated homologs to Zm00001d046136_T001 from maize.<br>>Authors:<br>We agree with the reviewer and revised the relevant description in the manuscript.<br><br>>Reviewer:<br>2.Table 1: Gap ratio means nothing, and should be removed. "Average" should be added to Gene/CDS/Exon/Intron length or number.<br>>Authors:<br>As suggested by the reviewer, we have removed gap ratio and added "average" to Gene/CDS/Exon/Intron length in Table 1.<br><br>>Reviewer:<br>3.Fig1B: Why the texts in the first box are not center aligned as the rest boxed? "Genome polishing", "chromosome scaffolding by HiC".<br>>Authors:<br>As suggested by the reviewer, we have revised the format and text in Fig. 1B.<br><br>>Reviewer:<br>4.Fig1C: the legend of circos plot should be 'a/b/c/d/e' rather than "A/B/...". Track a has no unit (Mb). Track b and e show repeat density histogram and number of ncRNA, but I did not see the scale bars.<br>>Authors:<br>As suggested by the reviewer, we have changed the "A/B/C/D/E" to "a/b/c/d/e" in the circos plot. We have also added the unit and scale information in the Figure legend of Fig. 1C.<br><br>>Reviewer:<br>5.Fig2: Copia and Gypsy should be italic.<br>>Authors:<br>As suggested by the reviewer, we have changed the format of Copia and Gypsy to italic in Fig.2.<br><br>>Reviewer:<br>6.Data availability: None of accession numbers the author provided are accessible now

Powered by Editorial Manager® and ProduXion Manager® from Aries Systems Corporation

	and genome annotation should be also deposited. Before accepting this paper, these data should be public and available. Personally I hope the genome assembly, and annotation can be also submitted to NGDC.<br>>Authors:<br>As suggested by the reviewer, we have released all the deposited data, which are available for public. We have also submitted the genome assembly and annotation to NGDC. However, we didn't include NGDC accessions in the manuscript to avoid redundance.<br><br>Reviewer #2:<br>The authors have answered all my questions and I agree to accept the article.<br>>Authors:<br>We thank the reviewer's positive comment.
**Additional Information:**	
**Question**	**Response**
Are you submitting this manuscript to a special series or article collection?	No
**Experimental design and statistics**<br><br>Full details of the experimental design and statistical methods used should be given in the Methods section, as detailed in our Minimum Standards Reporting Checklist. Information essential to interpreting the data presented should be made available in the figure legends.<br><br>Have you included all the information requested in your manuscript?	Yes
**Resources**<br><br>A description of all resources used, including antibodies, cell lines, animals and software tools, with enough information to allow them to be uniquely identified, should be included in the Methods section. Authors are strongly encouraged to cite Research Resource Identifiers (RRIDs) for antibodies, model organisms and tools, where possible.<br><br>Have you included the information requested as detailed in our Minimum Standards Reporting Checklist?	Yes
**Availability of data and materials**	Yes

Powered by Editorial Manager® and ProduXion Manager® from Aries Systems Corporation

All datasets and code on which the conclusions of the paper rely must be either included in your submission or deposited in publicly available repositories (where available and ethically appropriate), referencing such data using a unique identifier in the references and in the "Availability of Data and Materials" section of your manuscript.<br><br>Have you have met the above requirement as detailed in our Minimum Standards Reporting Checklist?	

Manuscript
Click here to access/download;Manuscript;Isoetes sinensis genome rev2-final.docx

Chromosome-level reference genome of tetraploid *Isoetes sinensis* provides insights into evolution and adaption of lycophytes

Jinteng Cui[1†], Yunke Zhu[2,3†], Hai Du[4], Zhenhua Liu[5], Siqian Shen[1], Tongxin Wang[1], Wenwen Cui[1], Rong Zhang[6], Sanjie Jiang[7], Yanmin Wu[2], Xiaofeng Gu[2], Hao Yu[8] and Zhe Liang[2*]

[1]College of Landscape Architecture, Beijing University of Agriculture, Beijing, 102206. China

[2]Biotechnology Research Institute, Chinese Academy of Agricultural Sciences, Beijing, 100081, China

[3]Glbizzia Biosciences, Beijing, 102699, China

[4]College of Agronomy and Biotechnology, Southwest University, Chongqing, 400715, China

[5]BiosmartSeek, Wuhan, 430072, China

[6]Fisheries Science Institute, Beijing Academy of Agriculture and Forestry Sciences, Beijing, 100068, China

[7]BGI Genomics, Shenzhen 518083, China

[8]Department of Biological Sciences, National University of Singapore, 117543, Singapore

*Correspondence address. Zhe Liang, Biotechnology Research Institute, Chinese Academy of Agricultural Sciences, Beijing, 100081, China; E-mail: liangzhe@caas.cn

†These authors contributed equally: Jinteng Cui, Yunke Zhu.

Hai Du [0000-0003-4274-5724]；

Xiaofeng Gu [0000-0003-1127-4261];

Hao Yu [0000-0002-9778-8855];

Zhe Liang [0000-0001-6872-6463].

Abstract

Background

The Lycophyta species are the extant taxa most similar to early vascular plants that were once abundant on Earth. However, their distribution has greatly diminished. So far, the absence of chromosome level assembled lycophyte genomes, has hindered our understanding of evolution and environmental adaption of lycophytes.

Findings

We present the reference genome of the tetraploid aquatic quillwort, *Isoetes sinensis*, a lycophyte. This genome represents the first chromosome-level assembled genome of a tetraploid seed-free plant. Comparison of genomes between *I. sinensis* and the *I. taiwanensis* revealed conserved and different genomic features between diploid and polyploid lycophytes. Comparison of the *I. sinensis* genome with those of other species representing the evolutionary lineages of green plants revealed the inherited genetic tools for transcriptional regulation and most phytohormones in *I. sinensis*. The presence and absence of key genes related to development and stress responses provides insights into environmental adaption of lycophytes.

Conclusions

The high-quality reference genome and genomic analysis presented in this study are crucial for future genetic and the environmental studies of not only *I. sinensis* but also other lycophytes.

Keywords: *Isoetes sinensis*, genome, evolution, Lycophyta, polyploid, environmental stress

Introduction

The vascular plants that currently dominate the land can be categorized into two major phyla: Euphyllophyta and Lycophyta. Euphyllophyta includes seed plants and ferns, while the Lycophyta comprises spore-bearing species that exhibit the greatest similarity to the early vascular plants found in the fossil record. Lycophytes have the longest evolutionary history of among all groups of vascular plants and have had major impacts on biodiversity, soil formation [1], and CO_2 sequestration on our planet [2]. Modern lycophytes have a widespread distribution, ranging from the epiphytic habitats (e.g., *Lycopodium phlegmaria* [3]) to the aquatic habits (e.g., *Phylloglossum drummondii*). Some members of the Lycophyta can survive in a variety of extreme environments, such as deserts (e.g., *Selaginella lepidophylla* [4]), humid tropics (e.g., *Selaginella kraussiana*) and even in arctic and alpine regions [3]. However, the distribution area of lycophytes was greatly reduced when compared to seed plants. Some lycophytes, including several species in the lycopod genus *Isoetes* are endangered [5, 6]. The genetic basis for environmental adaptability of lycophytes remains largely unknown.

Lycophytes included diploid and polyploid species in many lineages. So far, four genomes of diploid lycophytes including *Selaginella moellendorffii* [7], *Selaginella tamariscina* [8], *Lycopodium clavatum* [9] and *Isoetes taiwanensis* [10] are available. However, they are scaffold assemblies, not chromosome-level assemblies. To date, the genomes of polyploid lycophytes have not yet been reported. The perennial aquatic lycophyte, *Isoetes sinensis* (NCBI:txid283158) (Fig. 1A), is a tetraploid (2n = 4x = 44) quillwort, belongs to the family Isoetaceae that diversified in the last 45–60 million years ago [11]. Among extant representatives of the earliest differentiated vascular plants [12, 13], *I. sinensis* was once widely distributed, but has now completely disappeared from most of their habitats except two restricted sites in China [14]. Like

other *Isoetes*, *I. sinensis* possess Crassulacean Acid Metabolism (CAM) system that is crucial for the plant adaptation to low CO_2 environment underwater [15].

Here, we report a reference genome sequence of *I. sinensis* assembled into 22 pseudochromosomes. Our comparative analyses of its genome with *I. taiwanensis* and those of green algae and land plants allow us to better understand the evolution of lycophytes and the genetic basis of the environmental adaptability of lycophytes.

Results and discussion

Assembly of a high-quality *Isoetes sinensis* genome

Our *K*-mer analysis revealed the genome size of *I. sinensis* to be approximately 2.25 Gb with heterozygosity value of 0.26%. We sequenced *I. sinensis* genome by generating 176.46 Gb (79.17× coverage) Illumina short reads, 97.01 Gb (43.52× coverage) PacBio SMRT HiFi long reads, and 237.7 Gb (111.50× coverage) Hi-C data. We subsequently assembled the 2.13 Gb *I. sinensis* genome into 22 pseudochromosomes consisting of 3,741 scaffolds with N50 length of 86.66 Mb (Fig. 1B, C; Supplementary Fig. S1A, S1B; Table 1; Supplementary Tables S1, S2). The longest chromosome is ~109.03 Mb and the shortest is ~70.83 Mb (Supplementary Table S3). Using a combination of Illumina and PacBio sequencing, we performed RNA sequencing (RNA-seq) of small RNAs, long noncoding RNAs (lncRNAs), and mRNAs isolated from different tissues of *I. sinensis* to facilitate genome annotation (Supplementary Table S4). By combining homology-based alignments and *ab initio* gene models, we annotated a total of 57,303 protein-coding genes, 75% of which were supported by RNA-seq data (Table 1). 52,531 coding genes (92%) were assigned to functional categories using the InterPro, NR, Swiss-Prot, and KEGG databases. BUSCO (96.5%) and CEGMA (98.39%) analyses

suggest that our genome assembly exhibits a high degree of completeness (Supplementary Table S5). LTR Assembly Index (LAI) value is 9.71, which was not high but among the top LAI values reported in polyploid genomes [16]. The lengths of exons and transcripts are comparable among *I. sinensis* and its closely related species *I. taiwanensis* and *S. moellendorffii*, while *I. sinensis* has fewer exons per gene and shorter introns (Fig. 2A and Supplementary Fig. S1C). We annotated 33,515 noncoding RNA genes including 8,975 tRNA, 17,453 rRNA, 1,797 miRNA, 1,194 snRNA, 279 snoRNA, and 3,817 lncRNA genes (Fig. 1C; Table 1; Supplementary Tables S6-12). Further, we annotated 12,886 pseudogenes containing either frameshift mutations or premature stop codons, or both (Supplementary Table S13).

Gene and genome evolution

Our ML phylogeny of 19 species of evolutionarily representative land plants and green algae indicates that *I. sinensis* and *I. taiwanensis* diverged from *S. moellendorffii* about 300 Mya (million years ago) (Fig. 3). One hypothesis has suggested that the tetraploid *I. sinensis* originated from hybridization between the diploid *I. yunguiensis* and *I. taiwanensis* [17]. We attempted to distinguish the *I. sinensis* genome into two subgenomes using genomic information from *I. taiwanensis*. However, genome-wide comparison (Supplementary Table S14) and phylogenetic analysis (Supplementary Fig. S2A) showed that the similarity between pairs of chromosomes of *I. sinensis* was greater than that between *I. sinensis* and *I. taiwanensis*, suggesting that *I. sinensis* was not directly derived from the hybridization of *I. yunguiensis* and *I. taiwanensis.* We further performed *K*-mer and Subphaser analysis. Clustering of counts of 13-mers identified two groups of chromosomes. However, pairs of chromosomes, such as Chr

3 and Chr 4, were found in same groups (Supplementary Fig. S3A). In addition, Subphaser analysis identified 9 chromosomes in subgenome 1 and 13 chromosomes in subgenome 2 (Supplementary Fig. S3B). These results suggested that our analyses were not able to identify the two subgenomes of *I. sinensis*. To facilitate the subsequent analysis, we adopted an approach similar to that used for the *Artemisia argyi* genome assembly [18], and artificially divided *I. sinensis* genome into two subgenomes, A and B, based on the lengths of chromosome pairs (Supplementary Table S3). Gene numbers were comparable between the two subgenomes with 93.4% of subgenome A genes as homoeologs of 95.0% of subgenome B genes (Supplementary Fig. S2B). We found high collinearity between allelic chromosome pairs (*i.e*., A01 and B01) but weaker collinearity between other regions (Supplementary Fig. S4A and Supplementary Tables S15, S16), indicating the stability of *I. sinensis* as a tetraploid species. Abundant synteny blocks were observed between *I. sinensis* and *I. taiwanensis* (Supplementary Fig. S4B), suggesting that collinear blocks were retained after polyploidization. The collinearity between seed-free and seed plants was little known due to lack of chromosomal genome assembly of seed-free plants. We found only two plausible synteny block between *I. sinensis* and *A. thaliana* and *Z. mays* (Supplementary Fig. S4C), which illustrates the very limited collinearity between *I. sinensis* and seed plants.

Gene family expansions and contractions are often closely related to the adaptive evolution of species [19]. We distinguished expansion and contraction of gene families among representative plant species using homology-based methods. Totals of 2,108 and 3,153 families had undergone expansion and contraction in *I. sinensis*, respectively (Fig. 3). Expanded gene families were mostly enriched for energy metabolism functions such as photosynthesis and oxidative phosphorylation, while contracted gene families were mostly enriched in lipid metabolism functions such as linoleic acid metabolism

and fatty acid degradation (Supplementary Fig. S5). Notably, many more gene families that had expanded (4,687) and fewer that had contracted (1,817) were found in *I. taiwanensis* than in *I. sinensis* (Fig. 3), suggesting high genetic variation within *Isoetes*.

Diploid A and B subgenomes shared 15,280 orthologous gene families, which include 3,007 and 2,103 multicopy gene families in the A and B subgenome, respectively. Of the orthologous single copy gene sets in *I. taiwanensis*, 909 and 1,187 genes had been lost from the A and B subgenomes, respectively, of *I. sinensis*. These gene losses were also coincident with the smaller chromosome size of *I. sinensis* (96.8 Mb on average) relative to that of *I. taiwanensis* (150.9 Mb). Furthermore, 6,578 genes that exist as a single-copy in *I. taiwanensis* still exist as a single copy (one copy per subgenome) in each of the two *I. sinensis* subgenomes. To understand the effect of polyploidization on gene expression, we analyzed the gene expression bias between pairs of chromosomes in *I. sinensis* by using a similar approach that reported in *Brassica juncea* [20]. On average, 5,206 gene pairs showed homoeolog expression dominance. Notably, the number of dominant genes were comparable between 11 pairs of chromosomes. The exception was found in Chr10, where more than twice dominant genes in ChrB10 than that in ChrA10 (Supplementary Fig. S6). These results suggest that polyploidization might have affected the relative expression of homoeologs and likely equally affected the two subgenomes except Chr10.

WGD and repeat elements

Analysis of synonymous substitutions per synonymous site (*Ks*) suggests the occurrence of two whole-genome duplications (WGDs) with median values of 0.4 and 1.8 in *I. sinensis*, and the strong peak ~1.8 may represent the *K*s values of homeologs

of the A and B subgenomes (Fig. 2B and Supplementary Tables S16, S17). The two WGDs is consistent with a previous 1KP transcriptome study that reported two WGDs (ISTEα and ISTEβ) in *I. tegetiformans* and *I. echinospora* [21], but in contrast to the single WGD found in *I. taiwanensis* [10], which suggests a complex evolutionary history within *Isoetes.*

In *I. sinensis*, repetitive sequences occupy 63.15% of the genome (Supplementary Tables S18, S19), a much higher proportion than in the genomes of *I. taiwanensis* and *S. moellendorffii* [7, 10]. These repetitive sequences were evenly distributed across the genome of *I. sinensis* (Fig. 1C). Most of the repeats in the *I. sinensis* genome (53.67%) are long terminal repeat (LTR) retrotransposons (Fig. 2C), and more than 30% of LTR insertions in the *I. sinensis* genome occurred recently (Fig. 2D). LTRs in *I. sinensis* are shorter than those in *I. taiwanensis*, but longer than those in *S. moellendorffii* (Fig. 2E). We found that fewer repeats in each subgenome of *I. sinensis* than that in *I. taiwanensis*, however, a greater number of LTR/*Copia* and *Gypsy* elements in each chromosome of *I. sinensis* than those in *I. taiwanensis* (Supplementary Table S19), which suggests that LTR copies have likely increased since the divergence of *I. sinensis* and *I. taiwanensis*. Next, we generated a phylogenetic tree to compare the evolution of the LTR retrotransposon *Gypsy* in *I. sinensis*, *I. taiwanensis*, and *S. moellendorffii*. In addition to transposons similar to those in *S. moellendorffii*, we found that many species-specific transposons had evolved in *I. sinensis* and *I. taiwanensis*, indicating the expansion of *Gypsy* in *Isoetes* (Fig. 2F).

Transcriptional regulation

We identified 1,461 sequences that encode transcription factors (TFs) belonging to 52 families in *I. sinensis* (Supplementary Tables S20, S21). We found that 2.86% of the protein-coding genes in *I. sinensis* encode TFs, relatively fewer than in other land plants but more than in green algae [22]. Genes that encode AP2/ERF, MYB and bHLH family members accounted for the highest proportion TF-encoding genes in *I. sinensis* (Fig. 4A). When we compared the number of TFs encoded by the diploid A and B subgenomes of *I. sinensis* and other plant genomes, we found that the number of TF-encoding genes increased likely along with organismal complexity, although we did note some exceptions [23]. For example, we found a larger number of genes encoding AP2/ERF, AP2/B3, CSD, and PPP1 in the subgenomes of *I. sinensis* than in the genomes of ferns (Fig. 4A). Interestingly, the gene encoding GeBP (GL1 enhancer binding protein) has been lost from *I. sinensis*, but is present in *S. moellendorffii* and bryophytes (Fig. 4A and Supplementary Table S20). Next, we analyzed the evolution of TF families and detected many *I. sinensis*-specific subfamilies as exemplified by the 2R-MYB family, which performs essential plant stress response functions and represents the second largest TF family in *I. sinensis.* A total of 90 2R-MYB-encoding genes were found in the genome of *I. sinensis*. Phylogenetic analysis suggests that 21 2R-MYB TFs belong to seven ancient subfamilies including S28, S21, S22, S23, S18, S8, and S68, which have functions in stress response and development [24, 25]. Among the other nine 2R-MYB TF subfamilies, six of them contain only *I. sinensis* sequences, suggesting a species-specific expansion of 2R-MYB TFs within *I. sinensis* (Fig. 4B and Supplementary Dataset S1). We observed that a majority of MYBs within group NS5 were located on a pair of chromosomes of *I. sinensis* (Supplementary Dataset S1), may suggest their tandem duplication before polyploidization.

Phytohormones

Although the genome sequences of *I. taiwanensis* and *S. moellendorffii* are available, little is yet known about phytohormone in the Lycophyta. To better understand phytohormone regulation in *I. sinensis*, we investigated both conserved and lost genes that related to synthesis, transport, and signal transduction of phytohormones.

The auxin biosynthesis pathway in flowering plants is conserved and includes one *TAA* (encoding tryptophan aminotransferase in *Arabidopsis*) and five *YUCCA* homologs encoding flavin monooxygenase-like enzymes [26]. However, only one *YUC* was found in *I. sinensis*. There is no TAA-encoding gene in *I. sinensis*, although its paralog *TAR* was detected (Supplementary Fig. S7 and Supplementary Datasets S2-6). The *I. sinensis* genome possesses the auxin signal transduction components *AUX1* and a small number of *SAUR* genes that are not found in early land plants, suggesting that these genes could have evolved in the lycophytes. Interestingly, *I. sinensis* does not carry the *IAA1* and *GH* genes that are present in seed plants, suggesting a stepwise acquisition of auxin signaling during land plant evolution.

Abscisic acid (ABA) is generated under environmental stress and leads to a series of reactions that allow plants to adapt to adverse conditions [27]. Almost all the genes that involved in ABA biosynthesis except *XD* and *AAO* are present in *I. sinensis* (Supplementary Fig. S7 and Supplementary Datasets S7-11). The PYL receptor mediates the ABA response in cells via a complex between ABA and PYL that inhibits a PP2C (group A phosphatase 2C) to activate SnRK2, a SNF1-related protein kinase 2. While genes encoding PP2C and SnRK2 exist in *I. sinensis*, only one homolog encoding the PYL receptor (PYL5) was found. Genes encoding downstream TFs, such as AREB/ABFs that are involved in desiccation tolerance were also detected in *I. sinensis*. In addition, almost all of the genes involved in the cytokinin/ethylene-

controlled signal transduction pathways exist in *I. sinensis,* except for those encoding the receptor CKR in the cytokinin (CK) signaling pathway and 1-aminocyclopropane-1-carboxylate oxidase (ACO), which exists only in seed plants [28] (Supplementary Fig. S7 and Supplementary Datasets S12-19). Jasmonic acid (JA) and gibberellin (GA) signaling pathways play important roles in response to biotic stress [29]. We identified almost all of the genes that constitute the JA and GA pathways in *I. sinensis* (Supplementary Fig. S7 and Supplementary Datasets S20-24). Like other plants, *I. sinensis* contains genes that encode JA biosynthetic enzymes such as LOX, AOC, AOS, JAR1, and OPR3, and genes encoding COI1 receptor and MYC transcription factor orthologs. Among the few exceptions are genes encoding GA synthesis and transport functions such as *PIL* and *GA3OX* that are present in the genomes of green algae and early land plants, but have been lost from the *I. sinensis* genome. Taken together, the presence of these orthologs suggest nearly intact ABA, cytokinin, ethylene, JA, and GA signaling pathways in *I. sinensis*.

On the other hand, we found a paucity of genes involved in the strigolactone (SL) and salicylic acid (SA) signaling pathways in *I. sinensis* (Supplementary Fig. S7 and Supplementary Datasets S25-28). For example, apart from only one *MAX2* gene, *I. sinensis* has lost many other genes with functions in SL signaling. Furthermore, only a few components of the BR pathway (BRI1-like and DET2) can be detected in *I. sinensis* (Supplementary Datasets S29-32). As for SA signaling, we detected genes encoding CUL3, but none encoding NPR or BOP in *I. sinensis*.

We further compared the genes involved in phytohormone between *I. sinensis* and *I. taiwanensis*. Except for a small number of genes found only in *I. sinensis*, such as *GA2OX*, *AOC3*, and the genes found only in *I. taiwanensis* such as *BAK1*, *ACO4*, *ACS2*, *ACS4*, *JAZ*, most of genes are conserved with slight copy number variation between

these two *Isoetes* species (Supplementary Table S22). This result might suggest a conserved phytohormone regulation between *I. sinensis* and *I. taiwanensis.*

CAM photosynthesis

Crassulacean acid metabolism (CAM) is a metabolic pathway that concentrates CO_2 in plant cells to help some land plant species avoid drought and aquatic plant species avoid CO_2 limitation [30]. This adaptation is widespread in *Isoetes*, wherein carbon accumulates as malic acid during the night and enters the Calvin cycle during the day to improve CO_2 utilization [15]. Recently, the evolutionary path of CAM in *I. taiwanensis* has been described [10]. As does *I. taiwanensis*, *I. sinensis* possesses genes encoding both bacterial- and plant-type PHOSPHOENOLPYRUVATE CARBOXYLASE (PEPC) (Supplementary Fig. S8A, B), a key enzyme in CAM and C4 photosynthesis in various plant species. *I. sinensis* expresses the bacterial-type *PEPC* at a low-level and expresses the plant-type *PEPC* at a high level in roots, shoots, and sporangia, in contrast to the higher expression of bacterial-type *PEPC* than plant-type *PEPC* during development in *I. taiwanensis* (Supplementary Fig. S8C). In addition, *I. sinensis* lacks a gene encoding PHOSPHOENOLPYRUVATE CARBOXYKINASE (PEPCK) (Supplementary Fig. S7B), which participates in one of two important decarboxylation pathways in *I. taiwanensis*, suggesting differences in mechanisms of CAM across aquatic plants.

Stomatal development

Some aquatic plant species do not develop stomata or have nonfunctional stomata occluded by wax [31]. Functional stomata are important for *Isoetes* to adapt to

amphibiotic conditions. However, we found that some key genes for stomata development such as *SPEECHLESS* (*SPCH*), *MYB88,* and *MUTE* [32, 33], are not present both in the genomes of either *I. sinensis* or *I. taiwanensis* (Supplementary Fig. S9), suggesting specialized stomatal regulation in *Isoetes. I. taiwanensis* leaves have relatively fewer stomata than do those of *I. sinensis* [34]. Thus, we compared the *I. sinensis* and *I. taiwanensis* genes likely involved in stomatal development or regulation [35] and identified 45 of these genes in the *I. sinensis* genome and 39 in the *I. taiwanensis* genome, from a total 75 genes that could have involved in these processes (Supplementary Fig. S9). The absence of some putative stomatal development genes from each genome might have contributed to the differences in stomatal number and regulation of stomatal development between *I. sinensis* and *I. taiwanensis*.

Adaptation to environmental stresses

Land plants are often threatened by adverse abiotic environmental conditions that limit their growth and development. By comparing the genomes of *I. sinensis* and *I. taiwanensis*, and transcriptomes of other 19 lycophytes from the 1KP project [36], we analyzed the genetic basis of lycophyte adaptation to environmental stresses.

Cold sensing and response

Our comparative analysis did not detect many of the key genes responsible for cold sensing or response in lycophytes (Supplementary Fig. S10 and Supplementary Datasets S33-45). Firstly, as a temperature stress sensor, Ca^{2+} can induce temperature-responsive gene expression [37, 38]. ANNEXIN1 (ANN1) is the essential Ca^{2+} osmotic transporter that mediates cold-triggered Ca^{2+} influx and freezing resistance [37].

However, *ANN1* is absent in *I. sinensis* and most of the other lycophytes (Fig. 5A and Supplementary Fig. S9A). Secondly, EARLY FLOWERING3 (ELF3), ELF4, and LUX ARRYTHMO (LUX) can form an evening complex (EC) to perceive temperature changes and regulate plant growth by directly repressing the expression of *PIF4* under cold temperatures [39]. *ELF4* was also not detected in all of the lycophytes (Fig. 5A and Supplementary Fig. S10A). Third, cold stress activates the transcription of TF-encoding genes including those encoding CBFs [40]. OST1 is a positive regulator in CBF-dependent cold signaling, while EGR2 phosphatase is a negative regulator of plant freezing tolerance via inhibition of OST1 kinase activity, which thereby reduces the expression of CBFs during cold stress responses. In addition, the negative transcriptional regulator of CBFs, MYB15 is degraded during cold stress. We did not detect *EGR2* and *MYB15* in all of the lycophytes (Fig. 5A and Supplementary Fig. S10A). The absence of these homologs suggests a diversification between lycophytes and model plant *Arabidopsis* in cold sensing and response pathway.

Drought and salinity sensing and response

Drought stress stimulates local production and accumulation of the hormone ABA in plant organs, which is an important way to improve water efficiency and drought resistance in plants [41]. ABA signaling is mediated by the ABA receptors PYR, PYL, and RCAR, and by the PP2Cs and SnRK2s [42, 43] that interact with them. The genes that encode these proteins are present in lycophytes (Supplementary Fig. S11A and Supplementary Datasets S46-66). ABA-activated SnRK2s are phosphorylated and phosphorylate the plasma membrane NADPH oxidase RbohD/F that generates O^{2-} and subsequently H_2O_2 [38]. Leucine-rich repeat receptor kinases HPCA1 and GHR1 then

sense this extracellular H_2O_2 and activate Ca^{2+} signaling via Ca^{2+} channels [44, 45]. In *Arabidopsis*, H_2O_2□ and ABA□induced stomatal closure is impaired in the *hpca1* mutant [44, 45]. However, the absence of the *HPCA1* from *I. sinensis* and many other lycophytes might adversely affect the drought resistance of these species (Fig. 5B and Supplementary Fig. S11A).

Salinity is another important environmental factor inducing abiotic stress in plants and can result in hyperosmotic stress in plant cells [46]. In *Arabidopsis*, the salt overly sensitive (SOS) pathway comprises the SOS3 and SCaBP8 calcium sensors, the SOS2 protein kinase, and the SOS1 plasma membrane Na^+/H^+ antiporter. When an *Arabidopsis* plant experiences salt stress, SOS3 and ScaBP8 sense the calcium signal, interact with SOS2 and activate its kinase activity, which then activates the reverse transport activity of SOS1 [47, 48]. Calcium signals in this system in *Arabidopsis* are mediated by the Ca^{2+}-permeable transporters AtANN1 and AtANN4 [49, 50]. The absence from the lycophytes of genes encoding the Ca^{2+} transporters ANN1 and ANN4 and those encoding the downstream sensor SOS3 and ScaBP8 might thus limit the adaptability of lycophytes to salt stress (Fig. 5B and Supplementary Fig. S11B).

On the other hand, we also observed some conserved pathways between lycophytes and angiosperms (Supplementary Figs. S11A-C) that might contribute to the adaption to drought and salinity in lycophytes.

Cadmium (Cd) stress

Water pollution and eutrophication result in heavy metal stress that critically endangers *I. sinensis* [51]. Cadmium is a heavy metal with high toxicity to plants [52]. Uptake of cadmium occurs in root cells, mainly mediated by NRAMP5, and its root-to-shoot

transport is completed by HMA2 and HMA4 [53]. HMA3 mediates an effective detoxification mechanism that limits Cd transport to shoots by accumulating Cd in vacuoles [53]. *Cadmium Accumulation in Leaf 1* (*CAL1*) encodes a defensin-like protein that can chelate cytosolic Cd and promotes secretion of Cd into intercellular spaces such as the cell wall apoplast and xylem to decrease the concentration of Cd in the cytosol during transport of Cd within the plant [54]. Homologs of *HMA3* and *CAL1* are not present in the *I. sinensis* and many lycophytes (Supplementary Fig. S12 and Supplementary Datasets S67-68), which could limit the ability of lycophytes to control the transport and accumulation of Cd.

The activities of phytohormones are important for plants to adapt to heavy metal stress [55]. For example, cadmium enhances the activity of *Gretchen Hagen 3* (*GH3*), a gene present in algae and land plants that reduces the level of active IAA by esterifying it with an amino acid, resulting in increased lignin synthesis and peroxidase activity during plant defenses to heavy metal toxicity [56]. Treatment of plants with Cd resulted in the accumulation of *ETR2* and *ERF1,* which encode ethylene receptors, whereas the abundance of transcripts for brassinosteroid (BR)-related genes such as DWARF and BR6ox, decreased, suggesting that Cd-mediated BR biosynthesis feedback is inhibited when the BR contents increase [57]. BR homeostasis also requires the transcription factor BZR1 [58]. However, the homologs of all of the genes relevant to heavy metal response mentioned above are absent of *I. sinensis* and those of most lycophytes (Fig. 5C and Supplementary Fig. S12), which could adversely affect their ability to adapt to Cd stress.

Conclusion

Here, we present a high-quality assembly and annotation of *I. sinensis* genome, which represents the first sequenced tetraploid genome with chromosome-level assembly for a seed-free plant. Comparative analysis between *I. sinensis* and its close related diploid species *I. taiwanensis* revealed the features of genome and polyploidy in lycophytes. We found the differences in CAM and stomatal regulation between *I. sinensis* and *I. taiwanensis.* Comparison of the genome of *I. sinensis* with genomes representing the evolutionary lineages of green algae and land plants has revealed that *I. sinensis* possesses some common genetic tools, such as those associated with transcriptional regulation and involved in ABA, cytokinin, ethylene, JA, and GA signaling pathways. On the other hand, we have also shown that some key genes involved in important genetic pathways, including strigolactone, salicylic acid, and stress responses (cold, drought, salinity, and cadmium), have been lost or not detected in the *I. sinensis* and many lycophytes. These findings are crucial for the understanding of lycophyte development and their adaptation to adverse abiotic environmental conditions.

Methods

Plant materials and genome sequencing

I. sinensis shoot materials were harvested from Yangdongcun, Beilun District, Ningbo, Zhejiang Provience of China. DNA was extracted using a modified cetyltrimethylammonium bromide (CTAB) procedure. DNA concentrations and purity were evaluated by NanoDrop and its quality analyzed by agarose gel electrophoresis. Paired-end libraries with a 350-bp inserts were prepared by following the Illumina protocols and were then sequenced in PE150 mode on the Illumina HiSeq X Ten platform (RRID:SCR_016385). A total of 176.46 Gb paired-end reads were obtained

for genome survey. The read mapping rate of the Illumina sequencing was 98.58%, covering 99.95% of *I. sinensis* genome. For the PacBio Sequel analysis, the libraries for single-molecule real-time (SMRT) genome sequencing were prepared according to the manufacturer's protocol for the sequencing platform and were then sequenced with SMRT sequencing at 43.52×coverage using four cells. A total of 97.01 Gb reads were obtained for the genome assembly. High-throughput chromosome conformation capture (Hi-C) sequencing libraries were produced as follows: Nuclei were isolated and fixed with the cross-linking agent paraformaldehyde and then the cross-linked DNA was treated with restriction enzymes. Biotin was then added to label the ends of oligonucleotides during terminal repair. Adjacent DNA fragments were joined using nuclease ligases. Protein was digested with a protease to dissociate the protein from the DNA. Then the genomic DNA was extracted and randomly sheared into 350 bp fragments using a Covaris crusher. The library was prepared according to manufacturer's instructions (Illumina) and sequenced on a HiSeq X Ten DNA system to obtain 150 bp paired-end sequences.

RNA-seq and full-length transcriptome sequencing

RNAs from roots, shoots and sporangia of *I. sinensis* were extracted using a RNeasy Plus Mini Kit (Qiagen). After that, rRNA was removed from total RNA samples using the RiBO-Zero™ Kit. The isolated mRNA (~1% of total RNA) was used as template to synthesize cDNA, then the cDNA was sheared into small fragments. Paired-end libraries were prepared from various tissues by following the Illumina protocols and were sequenced with PE150 mode on the Illumina Hiseq X Ten platform. Pooled samples from roots, shoots and sporangia pooling sample were used for the PacBio Sequel analysis. The libraries for SMRT genome sequencing were prepared according

to the manufacturer's protocol for the sequencing platform and then sequenced on a PacBio Sequel II with SMRT sequencing.

Genome assembly and annotation

Before *de novo* genome assembly, Illumina short reads were used for preliminary evaluation of the genome size, heterozygosity, and repeat sequence proportions by *K*-mer analysis. After data filtering and quality control, the short reads were first assembled using SOAPdenovo (RRID:SCR_010752) software to generate contigs. These contigs were further used to construct scaffolds according to their pair-end relationships. The QV score generated from Merqury (RRID:SCR_022964) was 46.1448, and the corresponding error rate was 2.4295e-05.

De novo genome assembly of the PacBio long reads from *I. sinensis* genomes was performed using Hifiasm program (RRID:SCR_021069) [59]. The primary contigs were polished by aligning PacBio SMRT reads using the NextPolish software with the default parameters [60]. The consensus sequences for scaffolds were further polished based on Illumina paired-end reads using Pilon (RRID:SCR_014731). The total length of this assembly was 2,131.51 Mb, with a contig N50 up to 2,673 kb.

For the chromosome-level assembly, the clean Hi-C sequencing data were mapped to the draft genome using the Burrows-Wheeler Aligner (BWA) [61], and the repeated and unmatched data were removed by SAMtools (RRID:SCR_005227) [62]. Only unique valid paired-end reads were retained for subsequent chromosome-level assembly. Draft genome scaffolds were clustered according to interactions using the ALLHiC software (RRID:SCR_022750) [63]. Finally, about 90.10% sequences were grouped into 22 pseudochromosomes. Transcripts were aligned using Bowtie 2

(v.2.3.4.1) [64] software with the parameters (--no-mixed --no-discordant). The transcriptome was then quantified using RSEM (RRID:SCR_000262) (v.1.3.1) [65] with default parameters. After RNA-seq analysis, we found a total of 43,154 expressed genes accounting for 75.3% of the total predicted genes, which proved the high reliability of our genome annotation.

Genome completeness assessment

Genome completeness was evaluated using BUSCO (RRID:SCR_015008) [66] and CEGMA (RRID:SCR_015055) [67] analyses. BUSCO detected 84.7% complete and 3.2% fragmented BUSCO gene models in the assembly. CEGMA results suggested that 98.39% of core eukaryotic genes have been assembled. Small fragment library reads were selected and aligned to the assembled genome using BWA software (RRID:SCR_010910). Finally, 98.58% small fragment reads mapped to the *I. sinensis* genome. LTR Assembly Index (LAI) was evaluated by LTR_retriever (RRID:SCR_017623) (v2.9.0) [68].

Repeat sequence annotation

The repetitive sequences in *I. sinensis* was estimated by *de novo* strategies using RepeatModeler (RRID:SCR_015027), RepeatScout (RRID:SCR_014653), LTR_FINDER (RRID:SCR_015247) [69], MITE-Hunter (RRID:SCR_020946) [70], and PILER-DF [71]. A homology-based search for repeat sequences was carried out using RepeatMasker (RRID:SCR_012954) [72] to search Repbase (RRID:SCR_021169).

LTRs were identified using LTR_FINDER [69] and LTRharvest

(RRID:SCR_018970) [73], the results of which were then integrated with LTR_retriever [68] to build an accurate, non-redundant species-specific LTR database. Subsequently, we used homology-based prediction methods to annotate, filter out false positives, and annotate comprehensive and accurate species LTR sequences, including intact LTRs, solo LTRs, and LTR-related sequences.

LncRNA sequencing and analysis

Total RNA was extracted from each *I. sinensis* sample using RNeasy Plus Mini Kit, and rRNA removal was performed using a Ribo-Zero Kit. Isolated RNA was used for cDNA library construction, using the dUTP method [74]. These libraries were sequenced on an Illumina Hiseq X Ten platform. The purity, concentration, and integrity of RNA were checked using the agarose gel electrophoresis, the Qubit 2.0 Fluorometer, and the Agilent 4150 TapeStation, respectively. After trimming adapters and filtering out low-quality reads, a total of 14.02 Gb clean reads were generated. The transcriptome was mapped to the reference genome using TopHat2 [75]. Transcripts greater than 200 bp in length and containing at least two exons were considered as lncRNA candidates. Four computational approaches, including CPC [76], CNCI [77], Pfam (RRID:SCR_004726), and PhyloCSF [78] were combined to evaluate the protein-coding capability of the lncRNA candidates.

Small RNA sequencing and analysis

Small RNA libraries for *I. sinensis* were constructed using a Small RNA Sample Pre Kit for Illumina HiSeq sequencing. Raw reads were filtered by removing 3′-adapters, primers, and low-quality sequences using Cutadapt (RRID:SCR_011841) v1.9.1. Clean

reads of 18-30 nt were screened for subsequent analysis. The clean reads were mapped to Silva (RRID:SCR_006423), GtRNAdb database (RRID:SCR_006939), Rfam (RRID:SCR_007891) and Repbase (RRID:SCR_021169) to remove rRNAs, tRNAs, small nuclear RNAs (snRNAs), small nucleolar RNAs (snoRNAs), and other ncRNAs and repeats. The remaining reads were compared with reference miRNAs in the miRbase (RRID:SCR_003152) to annotate miRNAs. These reads were then mapped to the genome using Bowtie 2 (RRID:SCR_016368)[64].

Predictions of genes and noncoding RNAs

Gene annotation was performed by combining evidence drawn from ab initio prediction, homology-based gene prediction, and transcript evidence from RNA-seq data for *I. sinensis*. The *ab initio* gene prediction was conducted using two ab initio gene predictors Augustus (RRID:SCR_008417) [79] and Genscan (RRID:SCR_013362) with default parameters. Orthologous protein sequences were then aligned to the genome assembly using GeneWise (RRID:SCR_015054) [80]. In addition, the transcriptome data of the whole plant was used to predict genes using PASA [81]. Evidence Modeler [82] was used to generate a single high-confidence gene model set. Finally, 57,303 protein-coding genes were predicted for *I. sinensis* and all protein-coding genes were annotated to the public protein databases at KEGG (RRID:SCR_012773), SwissProt (RRID:SCR_021164), TrEMBL, and InterProScan v5.11-51.0 (RRID:SCR_005829), with an E-value cutoff of $1e^{-5}$. Pseudogenes were detected by exonerate (RRID:SCR_016088) (v.2.4) using the protein data of *Salvinia cucullata*, *Azolla filiculoides* [8] and *I. sinensis.*

We used two strategies to annotate noncoding RNAs, including *de novo* prediction

and direct RNA sequencing of small RNAs and lncRNAs. rRNA fragments were identified using BLAST against rRNA sequences of reference species in the Pfam database. tRNAs were identified using tRNAscn-SE. Additionally, other types of noncoding RNA, including miRNAs and snRNAs were identified at the Rfam database using INFERNAL (RRID:SCR_011809) [83].

Identification of WGD

In order to search for genome-wide duplications in the *I. sinensis* genome, we used WGDI (Whole-Genome Duplication Integrated analysis) tool for WGD and intragenomic collinearity detection as well as *Ks* estimation and peak fitting [84]. The WGD analyses were performed using all paralogous gene pairs.

Gene family and phylogenomic analysis

Gene families for the 19 species were analyzed and clustered using OrthoMCL (RRID:SCR_007839) (v. 2.0.9) with default parameters [85]. The 19 species including *A. thaliana*, *Vitis vinifera*, *Zea mays*, *Oryza sativa* [86], *Physcomitrella patens* [87], *Marchantia polymorpha* [26], *A. filiculoides*, *S. cucullata*, *Amborella trichopoda* [88], *Cycas panzhihuaensis* [89], *Picea abies* [90], *Gnetum montanum* [91], *S. moellendorffii* [7], *I. sinensis*, *I. taiwanensis* [10], *Mesostigma viride* [22], *C. reinhardtii* [92], *Klebsormidium nitens* [93], and *Chara braunii* [94] were used in the analysis. Gene families were clustered using OrthoMCL software with default parameters. During OrthoMCL gene family clustering, we defined single-copy gene families as genes existing as one copy in selected species and obtained a total of 66 single-copy gene families for further analysis. These single copy genes were aligned using software

MAFFT (RRID:SCR_011811) (v.7.490) and then ProTest (v.3.4.2) was used to find the best model of amino acid replacement in the single copy gene alignments. Before phylogeny construction, Gblocks (RRID:SCR_015945) (v.0.91b)[95] (-b5 = h) were used to remove gap regions of the multiple sequence alignments. Phylogenetic tree was constructed using RAxML (RRID:SCR_006086) (v.8.2.12) [96] with the maximum likelihood (ML) algorithm and 1,000 bootstrap replicates.

Based on a calibration of divergence times using the *C. reinhardtii* and *G. montanum* from TimeTree (RRID:SCR_021162), the divergence times for the inferred species tree were calculated using r8s (RRID:SCR_021161) (v.1.81) [97]. Gene families were used to calculate the expansion or contraction of the gene families in each lineage using CAFE (RRID:SCR_005983) (v.4.2.1) with p value < 0.05 [98]. *P* values were used to estimate the likelihood of the observed gene family sizes given average rates of gain and loss and were also used to determine expansion or contraction for individual gene families in each node.

Phylogenetic analysis of TF, phytohormone, CAM and stress response related genes

To identify TF, phytohormone, CAM and stress response related genes, we performed comparative genomic analysis of the genomes of *I. sinensis* and 13 representative plants or algae (including *A. thaliana*, *Vitis vinifera*, *Zea mays*, *P. patens*, *M. polymorpha*, *A. filiculoides*, *S. cucullata*, *P. abies*, *G. montanum*, *S. moellendorffii*, *I. taiwanensis*, *M. viride* and *C. reinhardtii*), and transcriptomes of other 19 lycophytes from the 1KP project [36]. BLASTP search (p value < 1e-5) was performed using well-studied proteins (mostly from *A. thaliana*) as queries to identify the homolog genes in *I. sinensis*. The redundant sequences were deleted, subsequently candidates were

examined for the conserved domain(s) of respective gene families using SMART (RRID:SCR_005026). Amino acid sequences of our target genes were aligned using Muscle. The alignments were then manually inspected using MEGA 7. MEGA 7 was ran with 1,000 bootstrap replicates to generate the neighbor-joining (NJ) phylogenetic trees [99].

Comparison of relative expression of homoeologs in the pairs of chromosomes of *I. sinensis*

We adopted the method used to analyze homoeologs expression in *Brassica juncea* [20] and focused on gene with 1:1 homoeologs between pairs of chromosomes of *I. sinensis*. DEG pairs with fold change > 2 were defined as dominant gene pairs. The dominant genes were defined as the genes with higher expression in dominant gene pairs, and the lower ones within dominant gene pairs were defined as subordinate genes. The rest of the genes with 1:1 homoeologs were defined as neutral genes.

Additional Files

Supplementary Fig. S1. Hi-C links and length of gene and CDS.

Supplementary Fig. S2. High similarity between two subgenomes of *I. sinensis.*

Supplementary Fig. S3. *K*-mer and Subphaser analysis were not able to separate the subgenomes of *I. sinensis*.

Supplementary Fig. S4. Collinearity analysis of *I. sinensis* genome.

Supplementary Fig. S5. KEGG analysis of expansion and contraction gene families during *I. sinensis* evolution.

Supplementary Fig. S6. Histograms of expression of 1:1 homoeologous genes between pairs of chromosomes among *I. sinensis* sporangia, shoot and root tissues.

Supplementary Fig. S7. Phytohormone biosynthesis and signaling pathways in *I. sinensis*.

Supplementary Fig. S8. CAM related genes in *I. sinensis*.

Supplementary Fig. S9. Stomatal regulation related genes in *I. sinensis* and *I. taiwanensis*.

Supplementary Fig. S10. Temperature stress related genes in *I. sinensis* and other lycophytes.

Supplementary Fig. S11. Salinity and drought stress related genes in *I. sinensis* and other lycophytes.

Supplementary Fig. S12. Cadmium stress related genes in *I. sinensis* and other lycophyte species.

Supplementary Table S1. Statistics of the *I. sinensis* genome sequencing.

Supplementary Table S2. Statistics of the *I. sinensis* genome assembly.

Supplementary Table S3. Statistics of chromosome length of the *I. sinensis* genome.

Supplementary Table S4. Statistics of the *I. sinensis* RNA-seq libraries.

Supplementary Table S5. Genome completeness assessment based on BUSCO for the *I. sinensis* genome assembly.

Supplementary Table S6. Summary of annotated non-coding RNA genes in *I. sinensis*.

Supplementary Table S7. List of annotated tRNA genes in *I. sinensis*.

Supplementary Table S8. List of annotated rRNA genes in *I. sinensis*.

Supplementary Table S9. List of annotated snRNA genes in *I. sinensis*.

Supplementary Table S10. List of annotated snoRNA genes in *I. sinensis*.

Supplementary Table S11. List of annotated miRNA genes in *I. sinensis*.

Supplementary Table S12. List of annotated lncRNA genes in *I. sinensis.*

Supplementary Table S13. List of annotated pseudogenes in *I. sinensis.*

Supplementary Table S14. Genome-wide comparisons and gene coverage analyses of *I. sinensis* and *I. taiwanensis.*

Supplementary Table S15. Specific gene pair information for the two subgenomes of *I. sinensis.*

Supplementary Table S16. Collinearity analysis between the proposed homeologs in *I. sinensis.*

Supplementary Table S17. *Ks* values of blocks in *I. sinensis.*

Supplementary Table S18. Summary of repeat distribution in *I. sinensis* and relative species.

Supplementary Table S19. Summary of repeats in *I. sinensis* and relative species.

Supplementary Table S20. Numbers of transcription factor genes in representative land plants and green algae.

Supplementary Table S21. IDs and sequences of transcription factor genes in *I. sinensis.*

Supplementary Table S22. IDs of phytohormones genes in *I. sinensis* and *I. taiwanensis.*

Supplementary Dataset S1-68. Phylogenetic relationships of proteins from *I. sinensis* and other evolutionarily representative species.

Abbreviations

ABA: abscisic acid; BUSCO: Benchmarking Universal Single-Copy Orthologs; CAM: Crassulacean acid metabolism; Cd: Cadmium; JA: jasmonic acid; LTR: long terminal repeat; NJ: neighbor-joining; SA: salicylic acid; SMRT: single-molecule real-time; TF:

transcription factor; WGD: whole-genome duplication.

Data Availability

The raw data of genome sequencing for *I. sinensis*, have been deposited in the NCBI SRA with the accession numbers: SRR17422691 (Illumina); SRR17422560, SRR17422559, SRR17422562, SRR17422561 (Hi-C); and SRR17640823, SRR17640824, SRR17640825, SRR17640826 (PacBio). The genome assembly and annotion has been deposited in the China National GeneBank DataBase with the accession number: CNA0072254. The raw data of RNA sequencing, including LncRNA sequencing, small RNA sequencing, mRNA□seq and full□length transcriptome sequencing of different tissues, have been deposited in the NCBI Gene Expression Omnibus (GEO) with the accession number: GSE198197. All additional supporting data are available in the *GigaScience* GigaDB database [100].

Authors' Contributions

J.C., Y.Z. and Z.L. conceived and designed the study. J.C., S.S., T.W., W.C. and R.Z. performed the experiments. J.C., Y.Z., H.D., Z.L., S.J., Y.W., X.G., H.Y., and Z.L. analyzed data. Y.Z. and Z.L. wrote the paper.

Competing Interests

All authors declare that they have no conflict of interest.

Finding

This work was supported by The Agricultural Science and Technology Innovation Program (ASTIP) (to Z.L.), Central Public-interest Scientific Institution Basal

Research Fund (to Z.L.), and Beijing Laboratory of Urban and Rural Eco-environment Project (to J.C.).

References

1. Kenrick P and Strullu-Derrien C. The origin and early evolution of roots. *Plant Physiol* 2014;**166**(2):570-80.
2. Beerling DJ and Berner RA. Feedbacks and the coevolution of plants and atmospheric CO2. *Proc Natl Acad Sci USA* 2005;**102**(5):1302-5.
3. Sporne KR. The Morphology of Pteridophytes. The structure of ferns and allied plants. 1962;**139**(3558):899.
4. Pampurova S and Van Dijck P. The desiccation tolerant secrets of *Selaginella lepidophylla*: what we have learned so far? *Plant Physio Biochem* 2014;**80**:285-90.
5. Taylor WCH, R.J. Habitat, evolution, and speciation in *Isoetes*. *Annals Missouri Bot Garden* 1992;**79**(3):613-22.
6. Kang M, Ye Q and Huang H. Genetic consequence of restricted habitat and population decline in endangered *Isoetes sinensis* (Isoetaceae). *Ann Bot* 2005;**96**(7):1265-74.
7. Banks JA, Nishiyama T, Hasebe M, *et al.* The *Selaginella* genome identifies genetic changes associated with the evolution of vascular plants. *Science* 2011;**332**(6032):960-3.
8. Xu Z, Xin T, Bartels D, *et al*. Genome analysis of the ancient tracheophyte *Selaginella tamariscina* reveals evolutionary features relevant to the acquisition of desiccation tolerance. *Mol Plant* 2018;**11**(7):983-94.
9. Yu J, Tang J, Wei R, *et al*. The first homosporous lycophyte genome revealed the association between the recent dynamic accumulation of LTR-RTs and genome size variation. *Plant Mol Biol* 2023;**112**(6):325-340.
10. Wickell D, Kuo L, Yang H, *et al*. Underwater CAM photosynthesis elucidated by Isoetes genome. *Nat Commun* 2021;**12**(1):6348.
11. Wood D, Besnard G, Beerling DJ, *et al*. Phylogenomics indicates the "living fossil" *Isoetes* diversified in the Cenozoic. *Plos One* 2020;**15:**6.
12. Liu X, Gituru WR and Wang Q. Distribution of basic diploid and polyploid species of *Isoetes* in East Asia. *J Biogeography* 2004;**31**(8):1239-50.

13. Pigg K. Isoetalean lycopsid evolution: from the Devonian to the present. *Am Fern J* 2001;**91**:99-114.
14. Chen Y, Kong D, Huang C, *et al*. Microsatellite analysis reveals the genetic structure and gene flow of the aquatic quillwort *Isoetes sinensis*, a critically endangered species in China. *Aquatic Bot* 2012;**96**:52-7.
15. Keeley J. Distribution of diurnal acid metabolism in the genus *Isoetes*. *Am Bot* 1982;**69**:254-7.
16. Ou S, Chen J and Jiang N. Assessing genome assembly quality using the LTR Assembly Index (LAI). *Nucleic Acids Res* 2018;**21**:e126.
17. Dai X, Li X, Huang Y *et al*. The speciation and adaptation of the polyploids: a case study of the Chinese *Isoetes L.* diploid-polyploid complex. *BMC Evol Biol* 2020;**20**(1):118.
18. Miao Y, Luo D, Zhao T, *et al*. Genome sequencing reveals chromosome fusion and extensive expansion of genes related to secondary metabolism in *Artemisia argyi*. *Plant Biotechnol J* 2022;**20**(10):1902-15.
19. Wan T, Liu Z, Leitch IJ, *et al*. The *Welwitschia* genome reveals a unique biology underpinning extreme longevity in deserts. *Nat Commun* 2021;**12**(1):4247.
20. Yang J, Liu D, Wang X, *et al*. The genome sequence of allopolyploid *Brassica juncea* and analysis of differential homoeolog gene expression influencing selection. *Nat Genet* 2016;**48**(10):1225-32.
21. Li Z and Barker M. Inferring putative ancient whole-genome duplications in the 1000 Plants (1KP) initiative: access to gene family phylogenies and age distributions. *GigaScience* 2020;**9**:giaa004.
22. Liang Z, Geng Y, Ji C, *et al*. *Mesostigma viride* genome and transcriptome provide insights into the origin and evolution of Streptophyta. *Adv Sci* 2020;**7**(1):1901850.
23. Catarino B, Hetherington AJ, Emms DM, *et al*. The stepwise increase in the number of transcription factor families in the precambrian predated the diversification of plants on land. *Mol Biol Evol* 2016;**33**(11):2815-9.
24. Jung C, Seo JS, Han SW, *et al*. Overexpression of *AtMYB44* enhances stomatal closure to confer abiotic stress tolerance in transgenic *Arabidopsis*. *Plant Physiol* 2008;**146**(2):623-35.
25. Du H, Liang Z, Zhao S, *et al*. The evolutionary history of R2R3-MYB proteins across 50 eukaryotes: new insights into subfamily classification and expansion.

Sci Rep 2015;**5**(1):11037.

26. Bowman JL, Kohchi T, Yamato KT, *et al*. Insights into land plant evolution garnered from the *Marchantia polymorpha* genome. *Cell* 2017;**171**(2):287-304.
27. Kim B, Brownlee SN, Grant JS *et al*. Gene expression characteristics in response to abscisic acid under shade. *Plant Mol Biol Rep* 2021;**40**:43–67.
28. Li F, Brouwer P, Carretero-Paulet L, *et al*. Fern genomes elucidate land plant evolution and cyanobacterial symbioses. *Nat Plants* 2018;**4**(7):460-72.
29. Li H, Chen S, Song A, *et al*. RNA-Seq derived identification of differential transcription in the chrysanthemum leaf following inoculation with *Alternaria tenuissima*. *BMC Genomics* 2014;**15**(1):9.
30. Keeley JE. CAM photosynthesis in submerged aquatic plants. *Bot Rev* 1998;**64**(2):121-75.
31. Pallardy SG. Transpiration and plant water balance. In: Pallardy SG ed. Physiology of Woody Plants (Third Edition). San Diego: Academic Press, 2008; 325-66.
32. Sugano SS, Shimada T, Imai Y, *et al*. Stomagen positively regulates stomatal density in *Arabidopsis*. *Nature* 2010;**463**(7278):241-4.
33. MacAlister CA, Ohashi-Ito K and Bergmann DC. Transcription factor control of asymmetric cell divisions that establish the stomatal lineage. *Nature* 2007;**445**(7127):537-40.
34. Liu JH, Fan LI, Xing JJ, *et al*. Comparative observation on the characteristics of leaves of four species in Chinese Isoetaceae. *Bullet Bot Res* 2013;**33**(5):528-13.
35. Cai S, Chen G, Wang Y, *et al*. Evolutionary conservation of ABA signaling for stomatal closure. *Plant Physiol* 2017;**174**(2):732-47.
36. Leebens-Mack JH, Barker MS, Carpenter EJ, *et al*. One thousand plant transcriptomes and the phylogenomics of green plants. *Nature* 2019;**574**(7780):679-85.
37. Liu Q, Ding Y, Shi Y, *et al*.The calcium transporter ANNEXIN1 mediates cold-induced calcium signaling and freezing tolerance in plants. *EMBO J* 2021;**40**(2):e104559.
38. Zhu JK. Abiotic stress signaling and responses in plants. *Cell* 2016;**167**(2):313-24.
39. Ezer D, Jung J, Lan H, *et al*. The evening complex coordinates environmental

and endogenous signals in *Arabidopsis*. *Nat Plants* 2017;**3**(7):17087.

40. Chinnusamy V, Zhu J and Zhu J. Cold stress regulation of gene expression in plants. *Trends Plant Sci* 2007;**12**(10):444-51.

41. Gupta A, Rico-Medina A and Caño-Delgado A. The physiology of plant responses to drought. *Science* 2020;**368**:266-9.

42. Cutler SR, Rodriguez PL, Finkelstein RR *et al*. Abscisic acid: emergence of a core signaling network. *Annu Rev Plant Biol* 2010;**61**:651-79.

43. Hauser F, Waadt R and Schroeder JI. Evolution of abscisic acid synthesis and signaling mechanisms. *Curr Biol* 2011;**21**(9):R346-55.

44. Wu F, Chi Y, Jiang Z, *et al*. Hydrogen peroxide sensor HPCA1 is an LRR receptor kinase in Arabidopsis. *Nature* 2020;**578**(7796):577-81.

45. Hua D, Wang C, He J, *et al*. A plasma membrane receptor kinase, GHR1, mediates abscisic acid- and hydrogen peroxide-regulated stomatal movement in *Arabidopsis*. *Plant Cell* 2012;**24**(6):2546-61.

46. Yang Y and Guo Y. Elucidating the molecular mechanisms mediating plant salt-stress responses. *New Phytol* 2018;**217**(2):523-39.

47. Quan R, Lin H, Mendoza I, *et al*. SCABP8/CBL10, a putative calcium sensor, interacts with the protein kinase SOS2 to protect *Arabidopsis* shoots from salt stress. Plant Cell 2007;**19**(4):1415-31.

48. Shi H, Ishitani M, Kim C *et al*. The *Arabidopsis thaliana* salt tolerance gene SOS1 encodes a putative Na+/H+ antiporter. *Proc Natl Acad Sci USA* 2000;**97**(12):6896-901.

49. Ma L, Ye J, Yang Y, *et al*. The SOS2-SCaBP8 complex generates and fine-tunes an AtANN4-dependent calcium signature under salt stress. *Dev Cell* 2019;**48**(5):697-709.e5.

50. Laohavisit A, Richards SL, Shabala L, *et al*. Salinity-induced calcium signaling and root adaptation in *Arabidopsis* require the calcium regulatory protein annexin1. *Plant Physiol* 2013;**163**(1):253-62.

51. Liu X, Wang J and Wang Q-F. Current status and conservation strategies for Isoetes in China: a case study for the conservation of threatened aquatic plants. *Oryx* 2005;**39**(3):335-8.

52. Tiwari S and Lata C. Heavy metal stress, signaling, and tolerance due to plant-associated microbes: an overview. *Front Plant Sci* 2018;**9**:452.

53. Nocito F, Lancilli C, Dendena B, *et al*. Cadmium retention in rice roots is

influenced by cadmium availability, chelation and translocation. Plant Cell Environ 2011;**34**(6):994-1008.

54. Luo J-S, Huang J, Zeng D-L, *et al*. A defensin-like protein drives cadmium efflux and allocation in rice. *Nat Commun* 2018;**9**(1):645.
55. Sytar O, Kumari P, Yadav S, *et al*. Phytohormone Priming: regulator for heavy metal stress in plants. *J Plant Growth Reg* 2019;**38**(2):739-52.
56. Elobeid M, Göbel C, Feussner I *et al*. Cadmium interferes with auxin physiology and lignification in poplar. *J Exp Bot* 2012;**63**(3):1413-21.
57. Schellingen K, Van Der Straeten D, Vandenbussche F, *et al*. Cadmium-induced ethylene production and responses in *Arabidopsis thaliana* rely on *ACS2* and *ACS6* gene expression. *BMC Plant Biol* 2014;**14**(1):214.
58. Villiers F, Jourdain A, Bastien O, *et al*. Evidence for functional interaction between brassinosteroids and cadmium response in *Arabidopsis thaliana*. *J Exp Bot* 2011;**63**(3):1185-200.
59. Cheng H, Concepcion GT, Feng X, *et al*. Haplotype-resolved de novo assembly using phased assembly graphs with hifiasm. *Nat Methods* 2021;**18**(2):170-5.
60. Hu J, Fan J, Sun Z and Liu S. NextPolish: a fast and efficient genome polishing tool for long-read assembly. *Bioinformatics* 2019;**36**(7):2253–5.
61. Li H and Durbin R. Fast and accurate short read alignment with Burrows-Wheeler transform. *Bioinformatics* 2009;**25**(14):1754-60.
62. Danecek P, Bonfield JK, Liddle J, Marshall J, Ohan V, Pollard MO, et al. Twelve years of SAMtools and BCFtools. Gigascience. 2021,10(2):giab008. doi: 10.1093/gigascience/giab008.
63. Zhang X, Zhang S, Zhao Q, *et al*. Assembly of allele-aware, chromosomal-scale autopolyploid genomes based on Hi-C data. *Nat Plants* 2019;**5**(5):833-45.
64. Langmead B and Salzberg SL. Fast gapped-read alignment with Bowtie 2. *Nat Methods* 2012;**9**(4):357-9.
65. Li B and Dewey CN. RSEM: accurate transcript quantification from RNA-Seq data with or without a reference genome. *BMC Bioinformatics* 2011;**12**(1):323.
66. Simão F, Waterhouse RM, Panagiotis I, *et al*. BUSCO: assessing genome assembly and annotation completeness with single-copy orthologs. *Bioinformatics* 2015;**31**(19):3210-2.
67. Parra G, Bradnam K and Korf I. CEGMA: a pipeline to accurately annotate core genes in eukaryotic genomes. *Bioinformatics* 2007;**23**(9):1061-7.

68. Ou S and Jiang N. LTR_retriever: a highly accurate and sensitive program for identification of long terminal repeat retrotransposons. *Plant Physiol* 2018;**176**(2):1410-22.
69. Xu Z and Wang H. LTR_FINDER: an efficient tool for the prediction of full-length LTR retrotransposons. *Nucleic Acids Res* 2007;**35**:W265-8.
70. Han Y and Wessler SR. MITE-Hunter: a program for discovering miniature inverted-repeat transposable elements from genomic sequences. *Nucleic Acids Res* 2010;**38**(22):e199.
71. Edgar RC and Myers EW. PILER: identification and classification of genomic repeats. *Bioinformatics* 2005;**21**(1):i152-8.
72. Tarailo-Graovac M and Chen N. Using RepeatMasker to identify repetitive elements in genomic sequences. *Curr Protoc Bioinformatics* 2009;**4**:10.
73. Ellinghaus D, Kurtz S and Willhoeft U. LTRharvest, an efficient and flexible software for de novo detection of LTR retrotransposons. *BMC Bioinformation* 2008;**9**(1):18.
74. Borodina T, Adjaye J and Sultan M. A strand-specific library preparation protocol for RNA sequencing. *Methods Enzymol* 2011;**500**:79-98.
75. Kim D, Pertea G, Trapnell C, *et al*. TopHat2: accurate alignment of transcriptomes in the presence of insertions, deletions and gene fusions. *Genome Biol* 2013;**14**(4):R36.
76. Kong L, Fau ZY, Ye Z, *et al*. CPC: assess the protein-coding potential of transcripts using sequence features and support vector machine. *Nucleic Acids Res* 2007;**35**:W345-9.
77. Sun L, Luo H, Bu D, *et al*. Utilizing sequence intrinsic composition to classify protein-coding and long non-coding transcripts *Nucleic Acids Res* 2013;**43**:e166.
78. Lin MF, Jungreis I and Kellis M. PhyloCSF: a comparative genomics method to distinguish protein coding and non-coding regions. *Bioinformatics* 2011;**27**(13):i275-i82.
79. Stanke M, Steinkamp R, Waack S *et al*. AUGUSTUS: a web server for gene finding in eukaryotes. *Nucleic Acids Res* 2004;**32**:W309-12.
80. Birney E, Clamp M and Durbin R. GeneWise and genomewise. *Genome Res* 2004;**14**(5):988-95.
81. Haas BJ, Delcher AL, Mount SM, *et al*. Improving the Arabidopsis genome

annotation using maximal transcript alignment assemblies. *Nucleic Acids Res* 2003;**31**(19):5654-66.

82. Haas BJ, Salzberg SL, Zhu W, *et al*. Automated eukaryotic gene structure annotation using EVidenceModeler and the program to assemble spliced alignments. *Genome Biol* 2008;**9**(1):R7.

83. Nawrocki, Eric P, Eddy *et al*. Infernal 1.1: 100-fold faster RNA homology searches. *Bioinformatics* 2013;**29**:2933–5.

84. Sun P, Jiao B, Yang Y, *et al*. WGDI: A user-friendly toolkit for evolutionary analyses of whole-genome duplications and ancestral karyotypes. Mol Plant. 2022;15(12):1841-1851. doi: 10.1016/j.molp.2022.10.018.

85. Li Li, Christian J. Stoeckert Jr. *et al*. OrthoMCL: identification of ortholog groups for eukaryotic genomes. *Genome Res* 2003;**13**(9):2178-89.

86. Zhang Q, Liang Z, Cui X, *et al*. N^6-Methyladenine DNA methylation in *Japonica* and *Indica* rice genomes and its association with gene expression, plant development, and stress responses. *Mol Plant* 2018;**11**(12):1492-508.

87. Rensing SA, Lang D, Zimmer AD, *et al*. The *Physcomitrella* genome reveals evolutionary insights into the conquest of land by plants. *Science* 2008;**319**(5859):64-9.

88. Amborella Genome P. The *Amborella* genome and the evolution of flowering plants. *Science* 2013;**342**(6165):1241089.

89. Liu Y, Wang S, Li L, *et al*. The Cycas genome and the early evolution of seed plants. *Nat Plants* 2022;**8**(4):389-401.

90. Nystedt B, Street NR, Wetterbom A, *et al*. The Norway spruce genome sequence and conifer genome evolution. *Nature* 2013;**497**(7451):579-84.

91. Wan T, Liu Z-M, Li L-F, *et al*. A genome for gnetophytes and early evolution of seed plants. *Nat Plants* 2018;**4**(2):82-9.

92. Witman GB, Terry A, Salamov A, *et al*. The *Chlamydomonas* genome reveals the evolution of key animal and plant functions. *Science* 2007;**318**(5848):245-50.

93. Hori K, Maruyama F, Fujisawa T, *et al*. *Klebsormidium flaccidum* genome reveals primary factors for plant terrestrial adaptation. *Nat Commun* 2014;**5**:3978.

94. Nishiyama T, Sakayama H, de Vries J, *et al*. The *Chara* genome: secondary complexity and implications for plant terrestrialization. *Cell* 2018;**174**(2):448-

64 e24.

95. Talavera G and Castresana J. Improvement of phylogenies after removing divergent and ambiguously aligned blocks from protein sequence alignments. *Systematic Biol* 2007;**56**(4):564-77.

96. Alexandros S. RAxML version 8: a tool for phylogenetic analysis and post-analysis of large phylogenies. *Bioinformatics* 2014;**30**(9):1312-3.

97. Sanderson MJ. r8s: Inferring absolute rates of molecular evolution and divergence times in the absence of a molecular clock. *Bioinformatics* 2003;**19**(2):301-2.

98. Bie TD, Cristianini N, Demuth JP, *et al*. CAFE: Computational Analysis of gene Family Evolution. *Bioinformatics* 2006;**22**(10):1269–71.

99. Caspermeyer J. MEGA software celebrates silver anniversary. *Mol Biol Evol* 2018;**35**(6):1558-60.

100. Cui J, Zhu Y, Du H, *et al*. Supporting data for "Chromosome-level reference genome of tetraploid Isoetes sinensis provides insights into evolution and adaption of lycophytes" GigaScience Database. 2023. http://dx.doi.org/10.5524/102447.

Table 1: Statistics of *I. sinensis* genome assembly and annotation.

**Feature**	***Isoetes sinensis***
Genome size (bp)	2,131,756,688
Contig number	4,329
Maximum contig length (bp)	13,293,339
Contig N50 (bp)	2,139,932
Contig N90 (bp)	228,882
Scaffold N50 (bp)	86,663,717
Scaffold N90 (bp)	70,828,552
Gene number	57,303
Average gene length (bp)	3,031.29
Average CDS length (bp)	1,098.39
Exon number per gene	4.79
Average exon length (bp)	294.98
Intron number per gene	3.79
Average intron length (bp)	426.34

Figure legends

Figure 1: *I. sinensis* morphology and genome assembly and annotation. (A) Morphological diagram of *I. sinensis*. The main body of the plant is 15–30 cm high, consisting of a rhizomatous and trilobed corm, with a tuft of roots at the base and long imbricate leaves at the top. The sporangia are basal and contain megaspores and/or microspores. The tissues analyzed using RNA-seq are indicated by arrows. (B) Diagram depicts workflow for assembly of the *I. sinensis* genome from PacBio HiFi long reads, Illumina short reads, and Hi-C data. (C) Circos plot represents the *I. sinensis* genome including a) 22 assembled pseudochromosomes. Unit, Mb, b) repeat content, c) gene density, d) pseudogene density, and e) ncRNAs including lncRNAs, miRNAs, rRNAs, snoRNAs, snRNAs, and tRNAs. Blocks of synteny of at least five gene pairs between the genomes are connected by linked lines at the center of the Circos plot. Different colors represent different pseudochromosomes or syntenic blocks. 500-Kb window size was selected to slide on the genome, the maximum repeat content and ncRNA content on each window were 1,696 and 1,146 respectively.

Figure 2: Genomic features of *I. sinensis* genome. (A) Boxplot showing intron, exon, and transcript length comparisons among the genomes of *I. sinensis, I. taiwanensis*, and *S. moellendorffii*. Boxes indicate the 1st quartile, median and 3rd quartile with whiskers extending up to 1.5 times the interquartile distance. (B) Frequency distribution of *Ks* based on the distribution of substitution rates of paralogs in three lycophytes (*I. sinensis, I. taiwanensis*, *S. moellendorffii*) and two ferns (*A. filiculoides* and *S. cucullata*). The two *Ks* peaks (0.4 and 1.8) indicate two WGDs in *I. sinensis.* (C) Pie chart illustrating of the major classes of repetitive DNA in *I. sinensis*. LINE, long interspersed nuclear element; LTR, long terminal repeat; SINE, short interspersed

transposable element; TR, tandem repeat. (D) The relative ages LTR retroelements computed as Kimura distances suggest a long period of retroelement transposition activity. (E) Boxplot showing distributions of LTR family lengths in *I. sinensis, I. taiwanensis* and *S. moellendorffii*. (F) Maximum likelihood phylogeny analysis of *Gypsy* retroelements showing the expansion of *Gypsy* in *I. sinensis* and *I. taiwanensis.*

Figure 3: Evolution analysis of gene families in *I. sinensis* and 19 selected evolutionarily representative green algae and land plants. The phylogenetic tree was constructed from Maximum Likelihood (ML) method. The green numbers on the branches of the phylogenetic tree indicate the number of expanded gene families, and the red numbers refer to the number of constricted gene families. The supposed most recent common ancestor (MRCA) contains 46,317 gene families. Totals of 2,108 and 3,153 families had undergone expansion and contraction in *I. sinensis*, respectively. Only one subgenome of *I. sinensis* was used. The number in blue circle indicate the retained duplicates from WGDs.

Figure 4: Transcription factors in *I. sinensis.* (A) Heat map illustrating the numbers of transcription factors in *I. sinensis* compared with 13 evolutionarily representative green algae and land plants. Detailed information is shown in Supplementary Table S23. (B) Neighbor-joining (NJ) phylogenetic analysis of R2R3-MYB proteins encoded by the genome of *I. sinensis*. The tree includes 90 R2R3-MYB sequences. Bootstrap replicates =1,000. See Supplementary Dataset S1 showing the detailed tree.

Figure 5: Abiotic stress responses in lycophytes. (A) Diagram showing the pathway and genes involved in cold sensing and response in plant. The key genes *EGR2*, *MYB15*

and *ANN1* were not detected in *I. sinensis* and most lycophytes. (B) Diagram showing the pathway and genes involved salinity and drought stress sensing and signaling. The key genes *MKK5*, *AIK1*, *MAP3K17/K18*, *HPCA1, ANN1*, *ANN4*, *SOS3* and SCaBP8 were not detected in *I. sinensis* and most lycophytes. Dotted lines and white text indicate the absence of genes.

Figure 1

Click here to access/download;Figure;Fig1.jpg

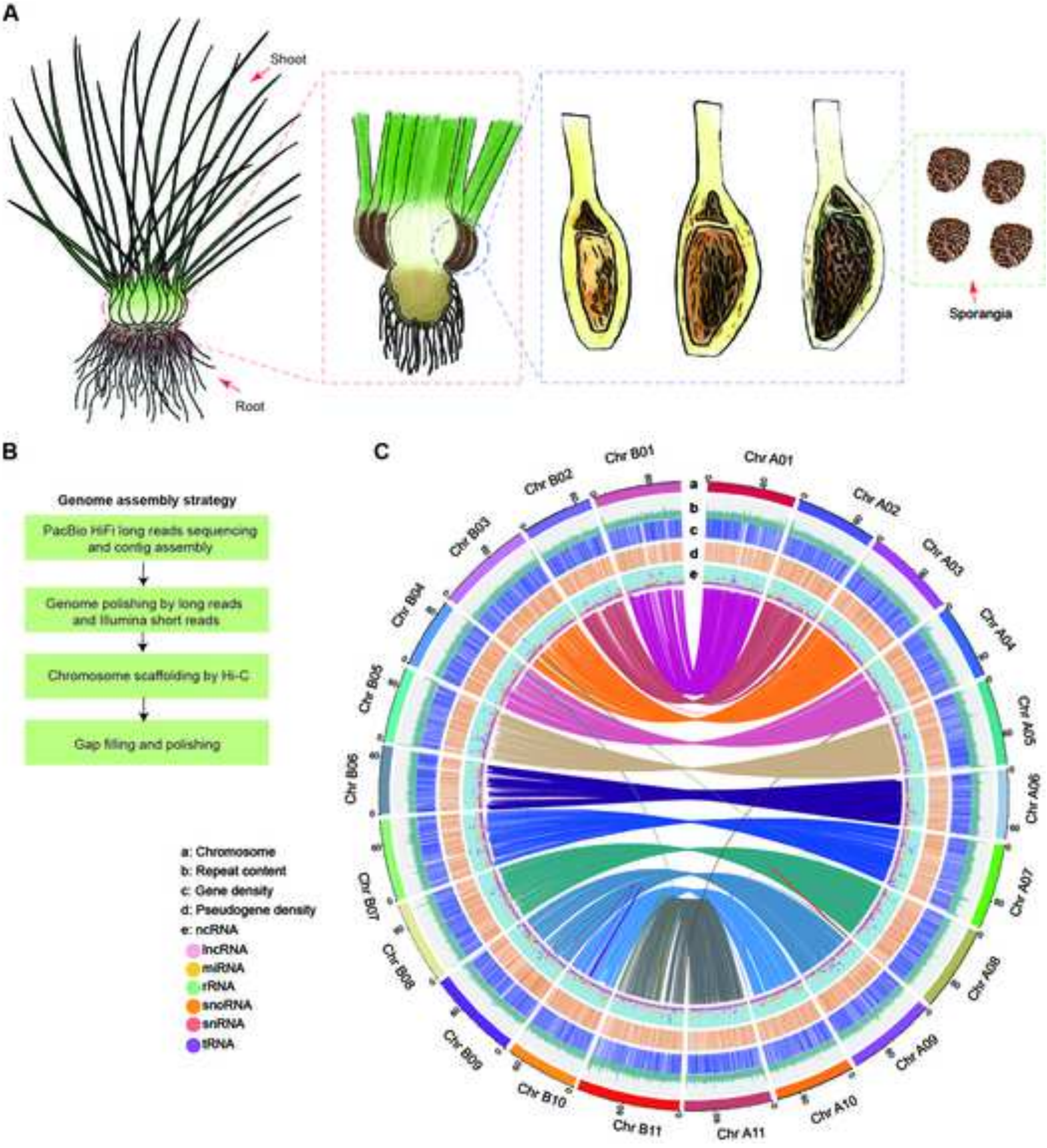

 Click here to access/download;Figure;Fig2.jpg

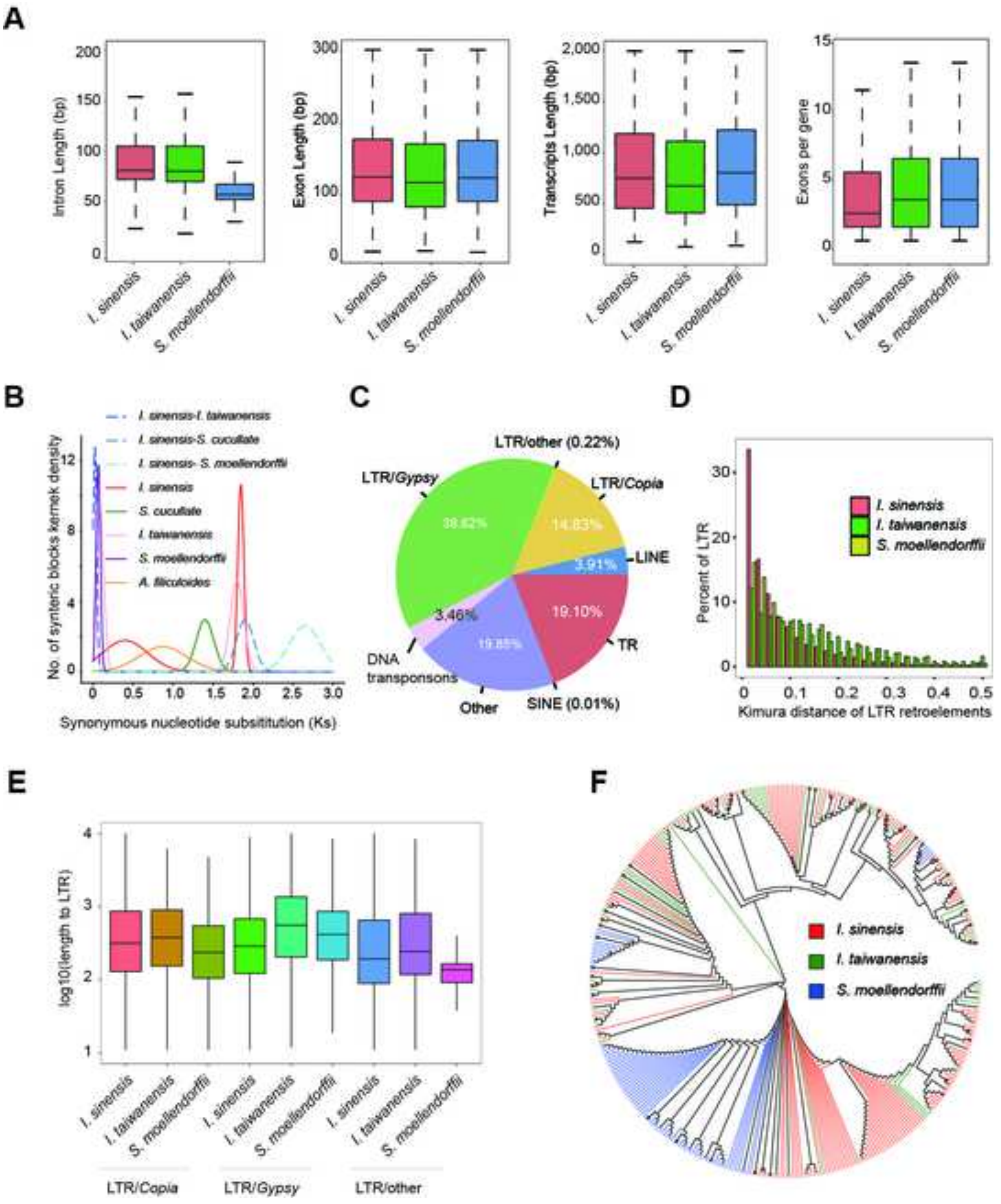

Figure 3 Click here to access/download;Figure;Fig3.jpg

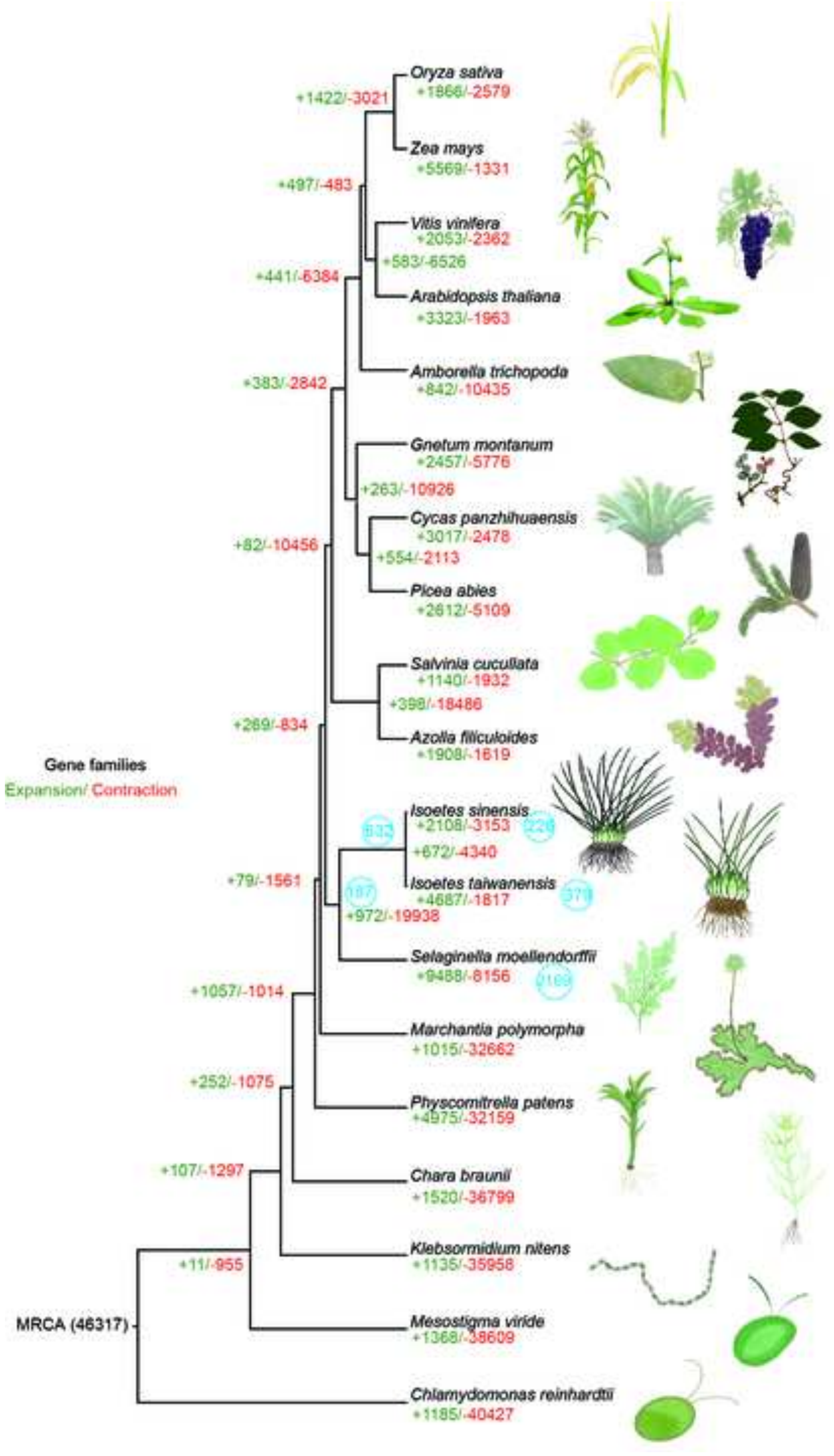

Figure 4

Click here to access/download;Figure;Fig4.jpg

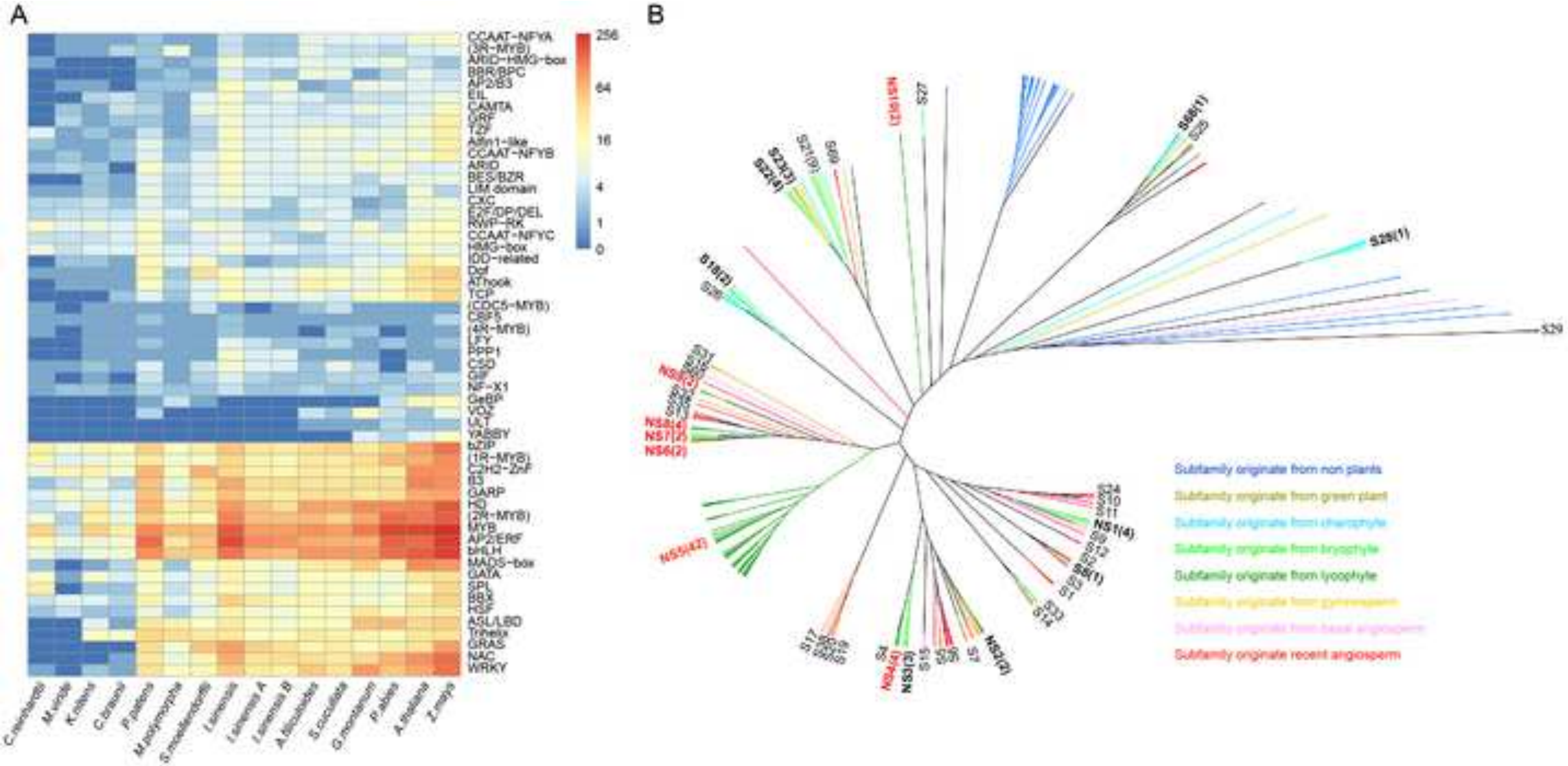

Figure 5

Click here to access/download;Figure;Fig5.jpg

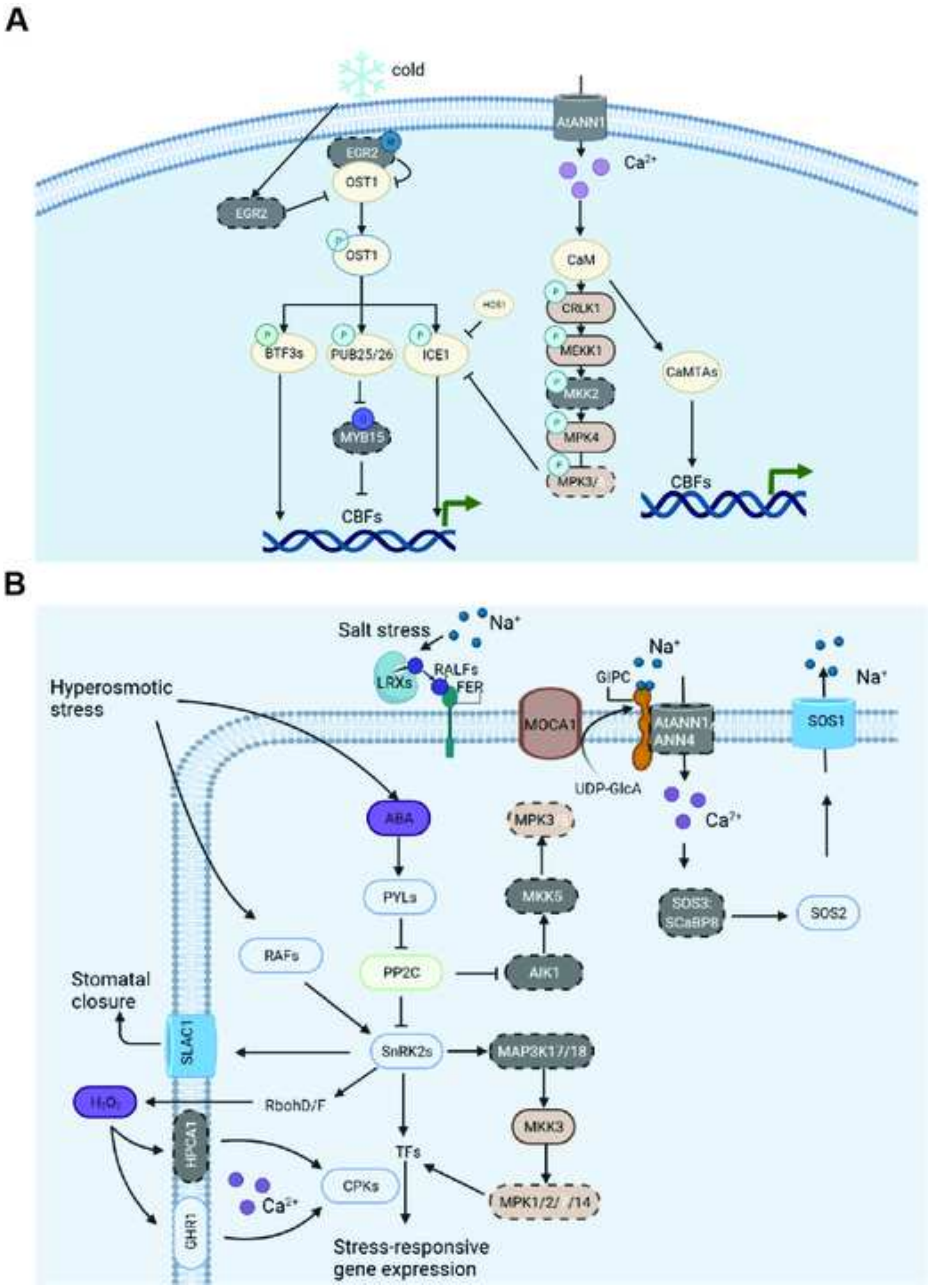

Supplementary figure

Click here to access/download
Supplementary Material
Supplementary Figure rev 2.docx

Click here to access/download
Supplementary Material
Supplementary Dataset.docx

Personal Cover Click here to access/download;Personal Cover;Cover Letter.docx

Manuscript No: GIGA-D-23-00116R1
Manuscript Title: Chromosome-level reference genome of tetraploid *Isoetes sinensis* provides insights into evolution and adaption of lycophytes

Dear Hongfang,

We would like to thank you and two reviewers for the time committed in reviewing our manuscript "Chromosome-level reference genome of tetraploid *Isoetes sinensis* provides insights into evolution and adaption of lycophytes", and for all the detailed suggestions on improving this manuscript. We have further revised the manuscript to fully address the reviewers' minor concerns, and all the sequencing data were released for public. Our point-by-point responses to the referees are listed in the section of "Response to Reviewers". We have highlighted the changes in yellow in the revised manuscript. With these revisions, we hope that the manuscript is now acceptable for publication.

Thanks for your consideration.

Sincerely yours,

Zhe Liang

Biotechnology Research Institute

Chinese Academy of Agricultural Sciences

Beijing, 100081, China

Email: liangzhe@caas.cn
