# Supplementary material for: Chromosome-level reference genome of tetraploid Isoetes sinensis provides insights into evolution and adaption of lycophytes
Source: Gigascience. 2023 Sep 30;12:giad079. doi: 10.1093/gigascience/giad079 (PMC10541799; doi:10.1093/gigascience/giad079)
Supplement: giad079_Supplemental_Files [file giad079_supplemental_files.zip › Supplementary Dataset.docx]

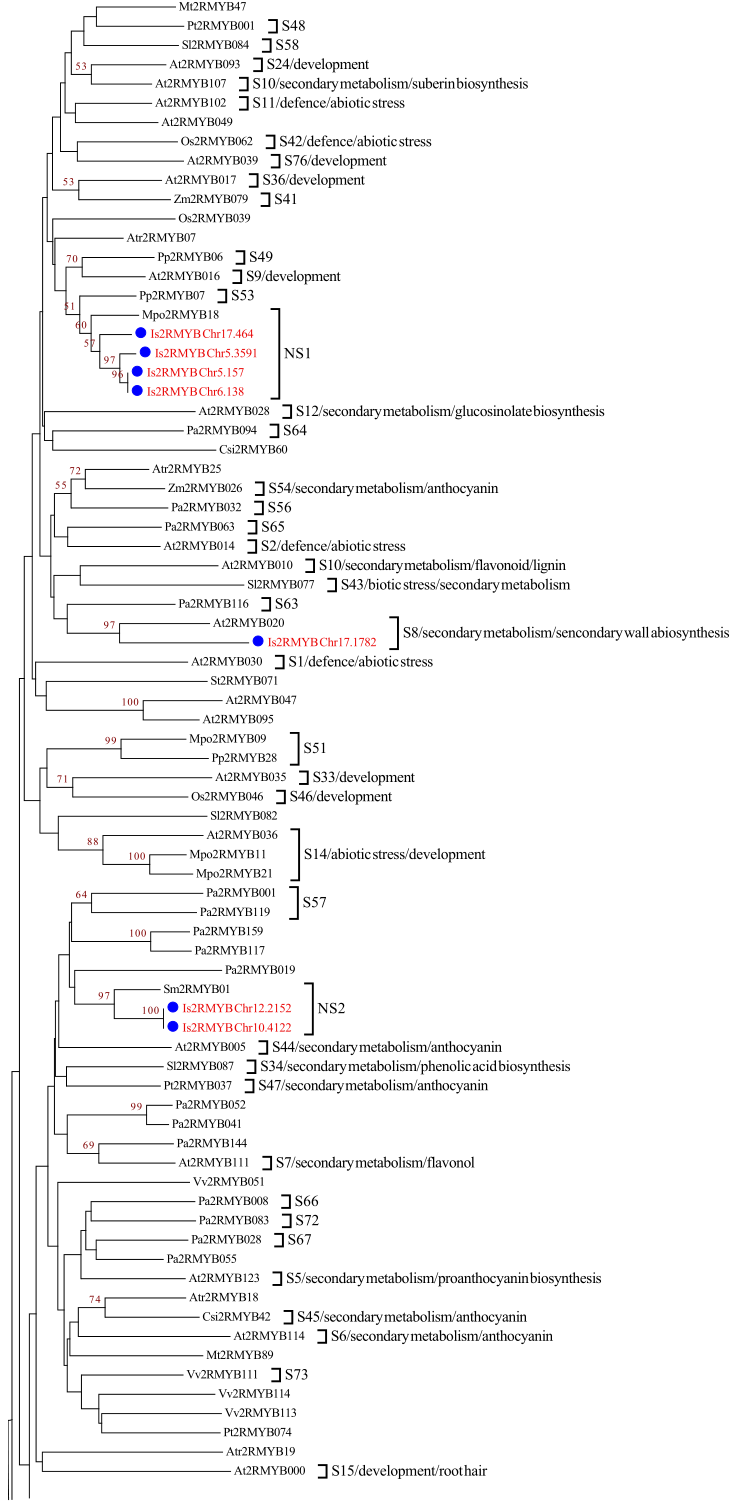

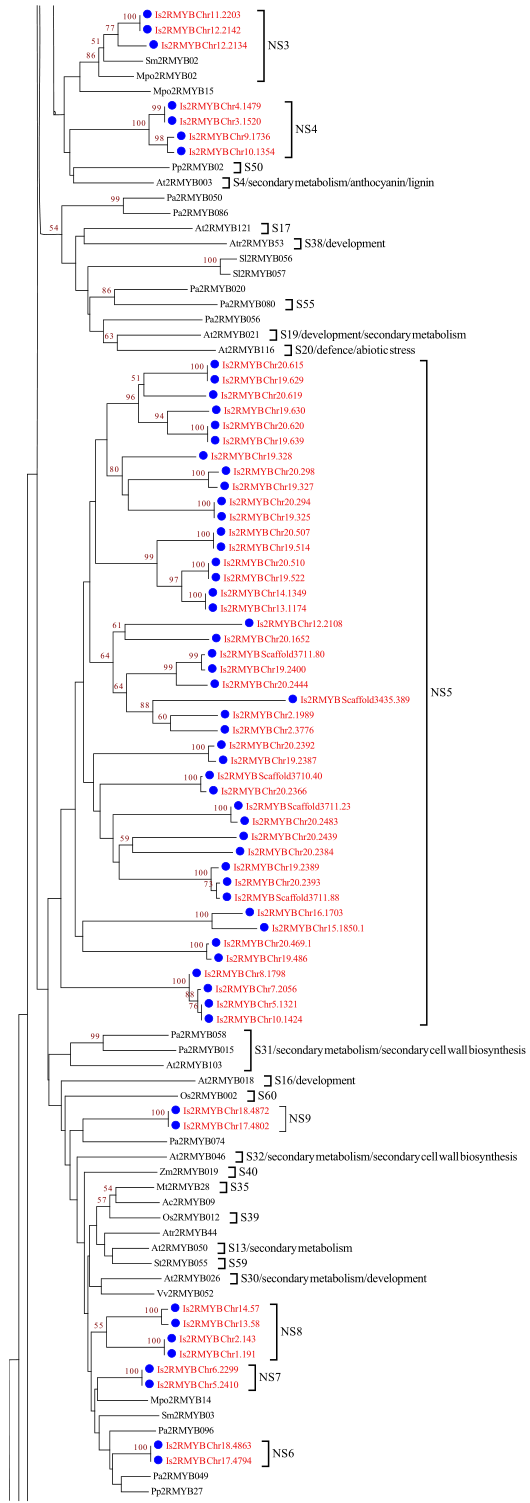


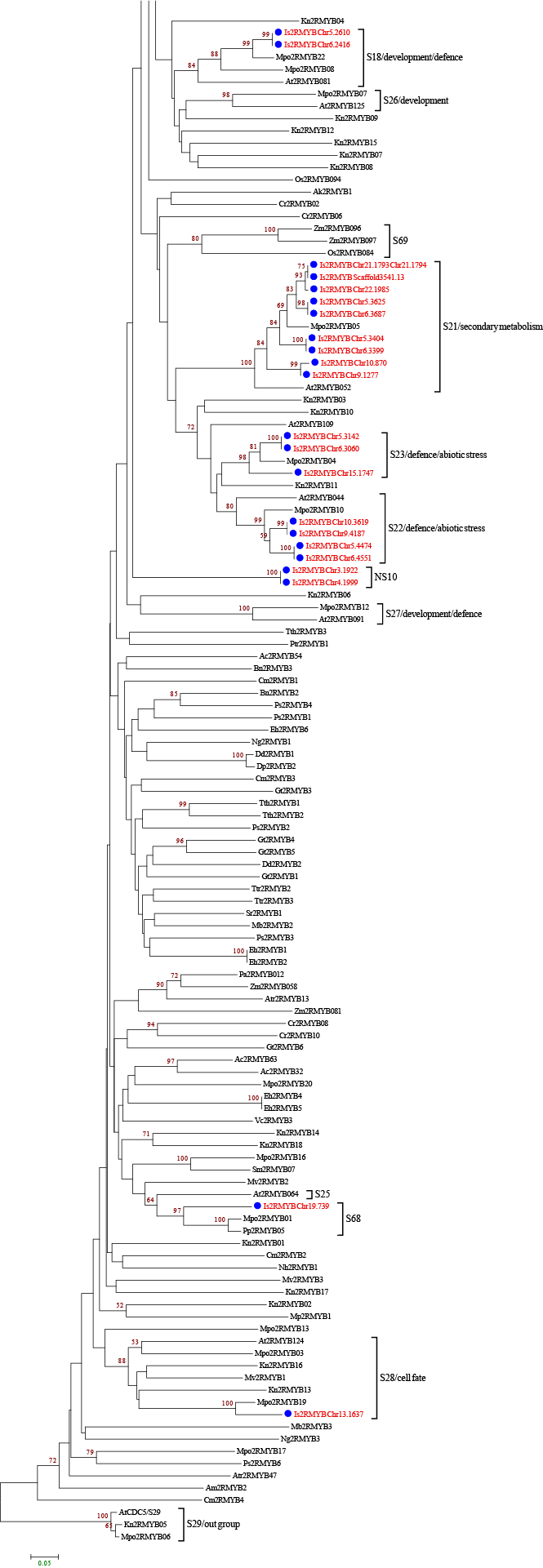


**Dataset S1. Phylogenetic relationships of 2R-MYB TFs from *I. sinensis* and other evolutionarily representative species.** Numbers on the major branches indicate bootstrap values (> 50%) in 1,000 replicates. In *I. sinensis*, some 2R-MYB genes belonged to seven ancient subfamilies, including: S28, S21, S22, S23, S18, S8 and S68. While most 2R-MYB genes did not belong to any subfamily of higher plants and were species or genus specific.


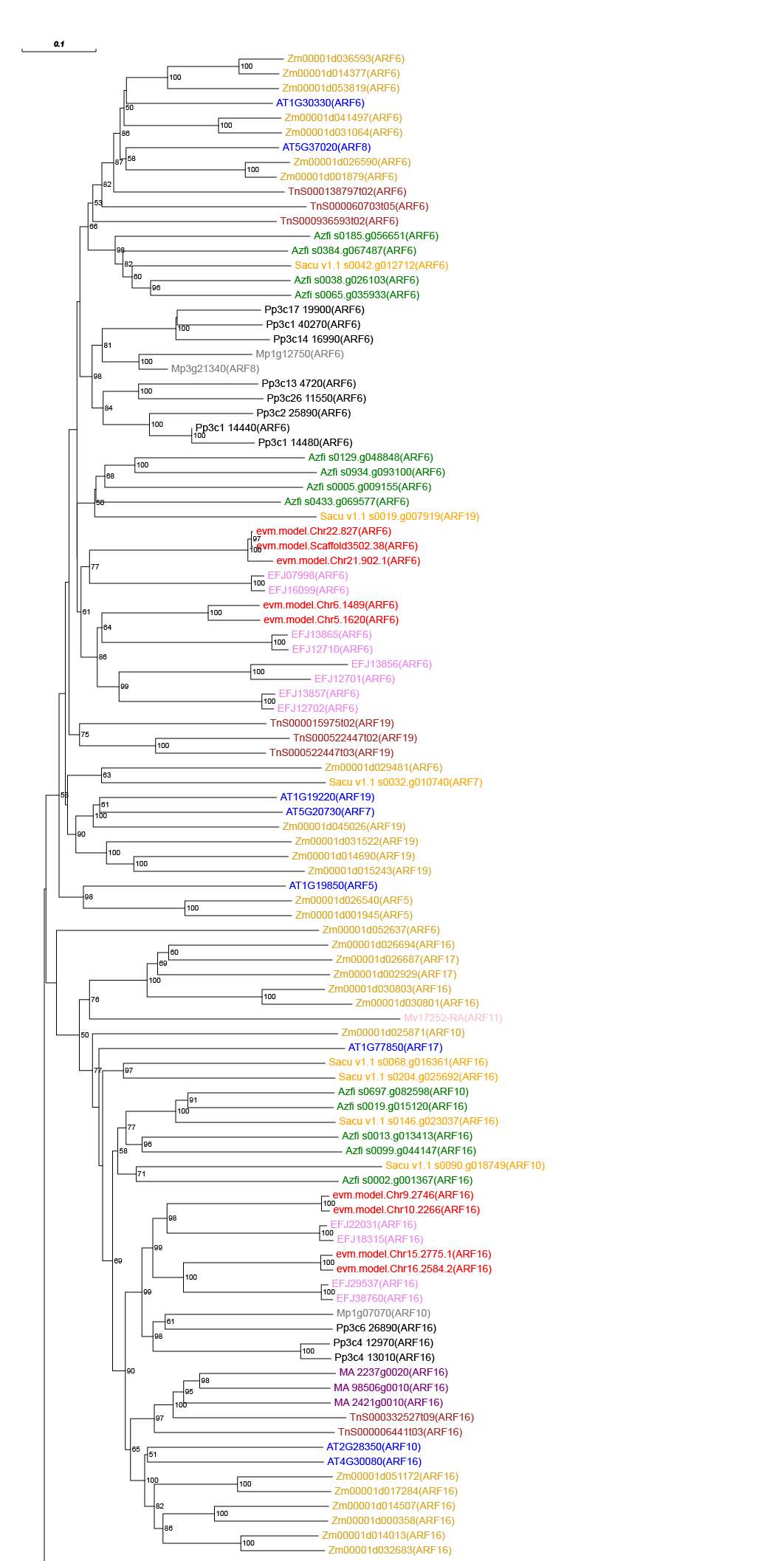

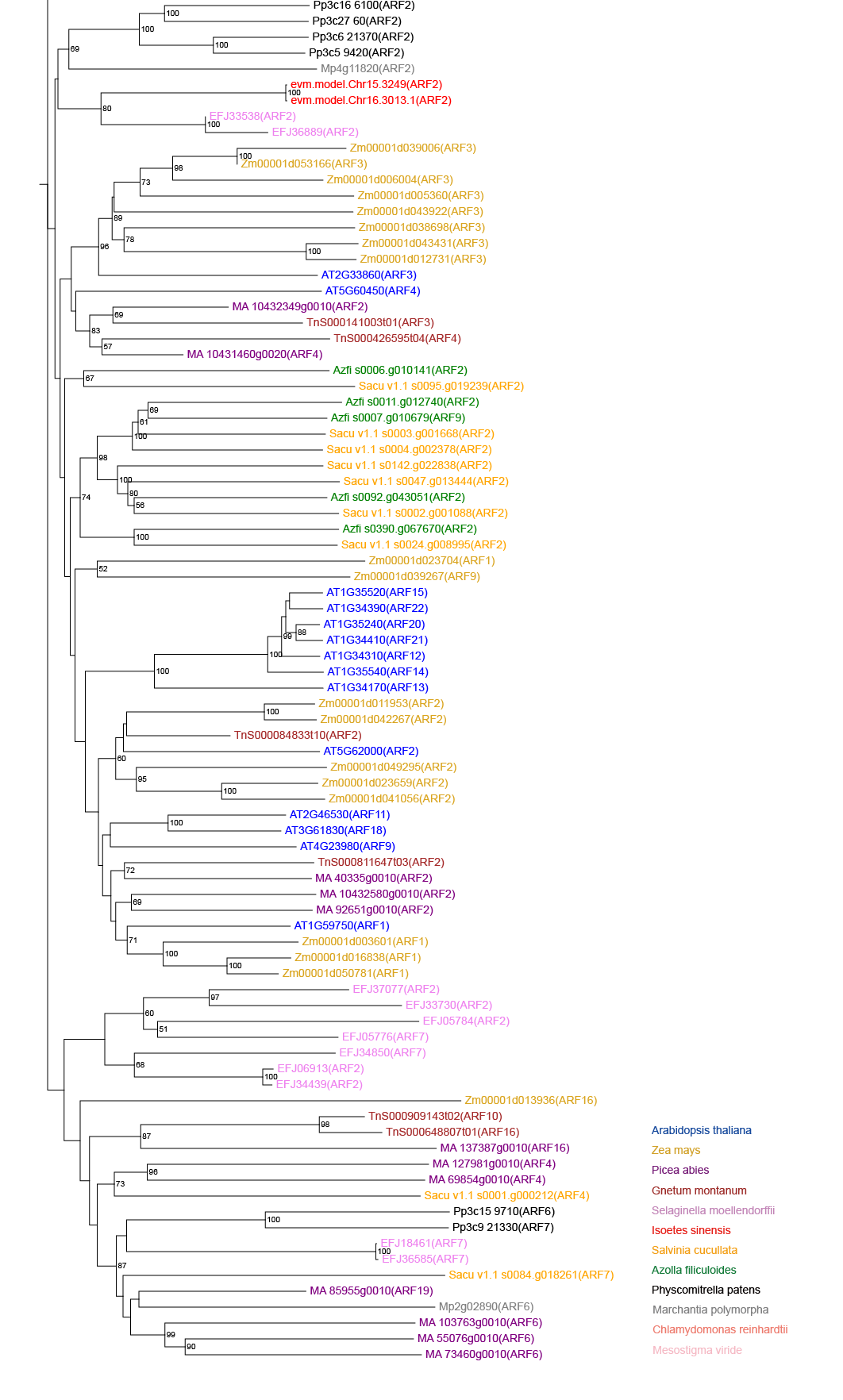


**Dataset S2. Phylogenetic relationships of ARF proteins from *I. sinensis* and other evolutionarily representative species.** Numbers on the major branches indicate bootstrap values (> 50%) in 1,000 replicates.


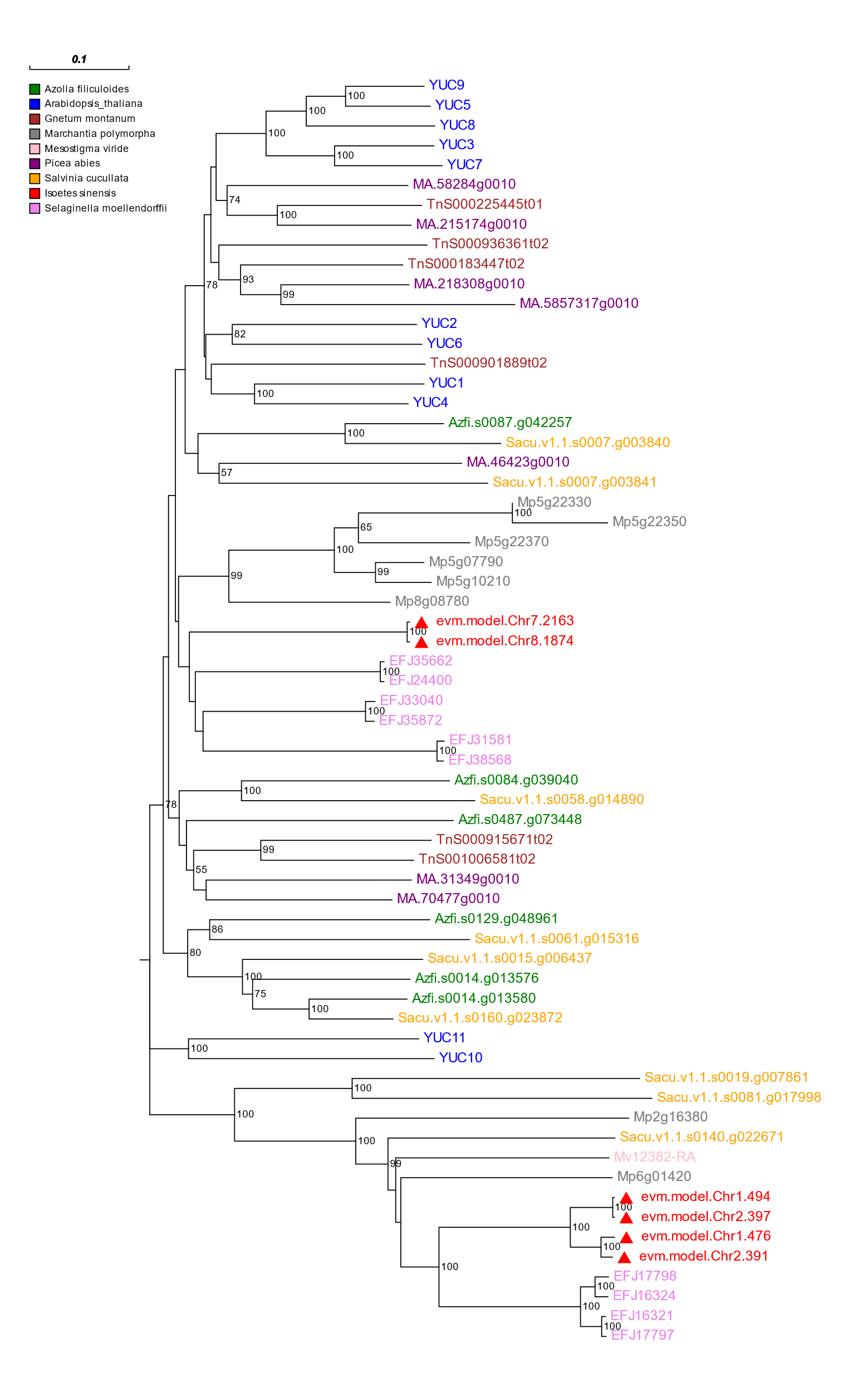


**Dataset S3. Phylogenetic relationships of YUC proteins from *I. sinensis* and other evolutionarily representative species.** Numbers on the major branches indicate bootstrap values (> 50%) in 1,000 replicates.


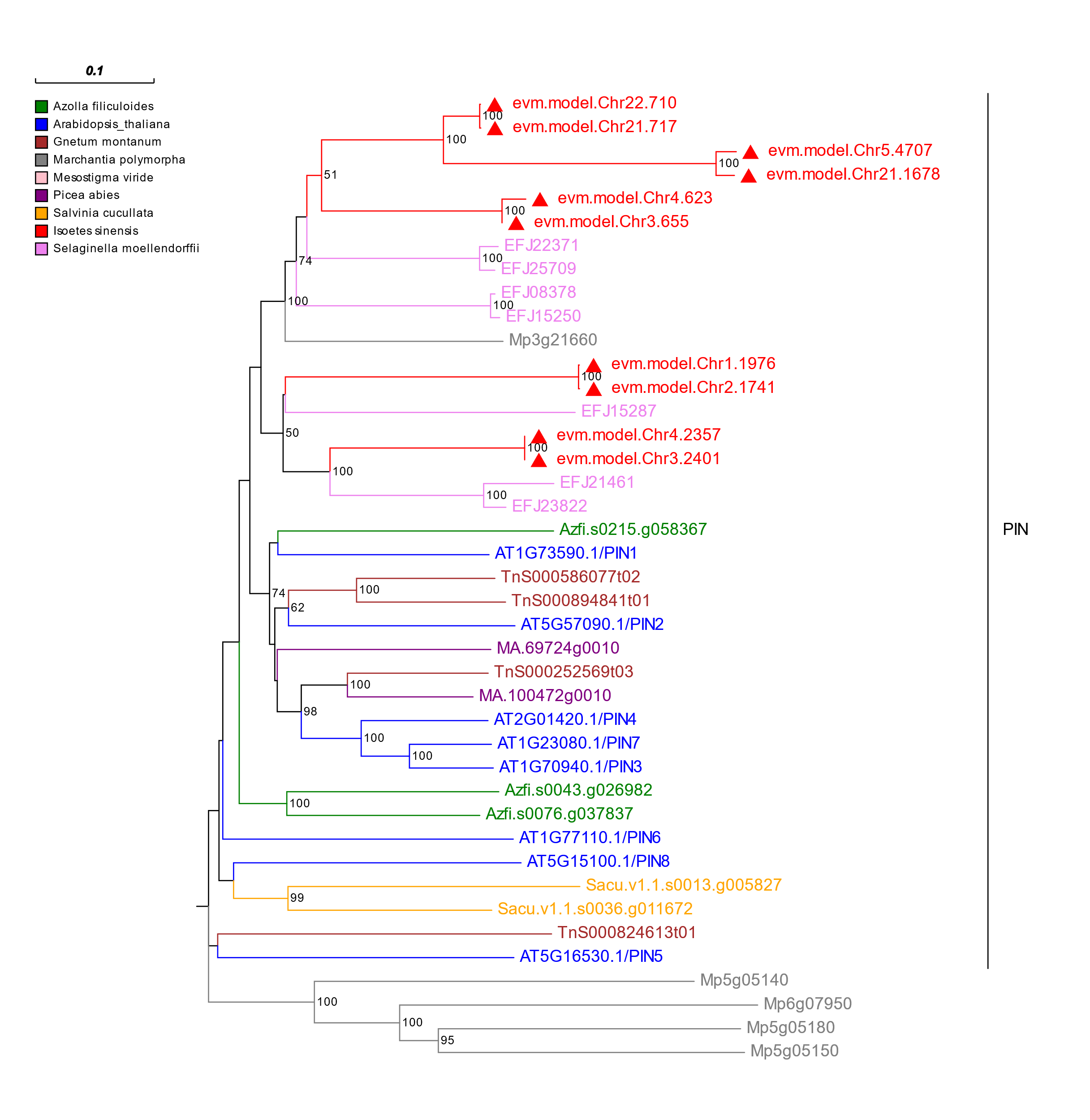


**Dataset S4. Phylogenetic relationships of PIN proteins from *I. sinensis* and other evolutionarily representative species.** Numbers on the major branches indicate bootstrap values (> 50%) in 1,000 replicates.


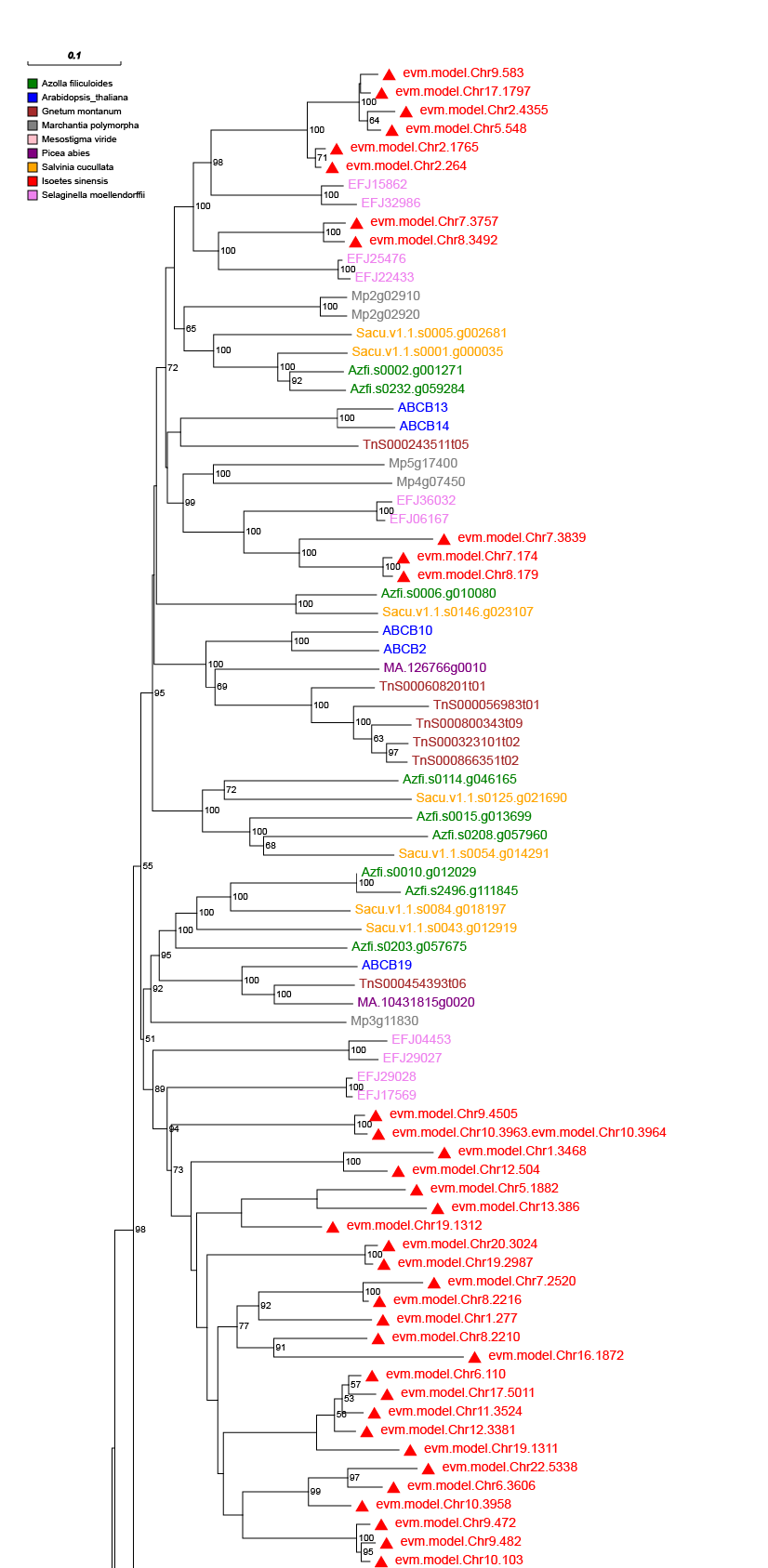

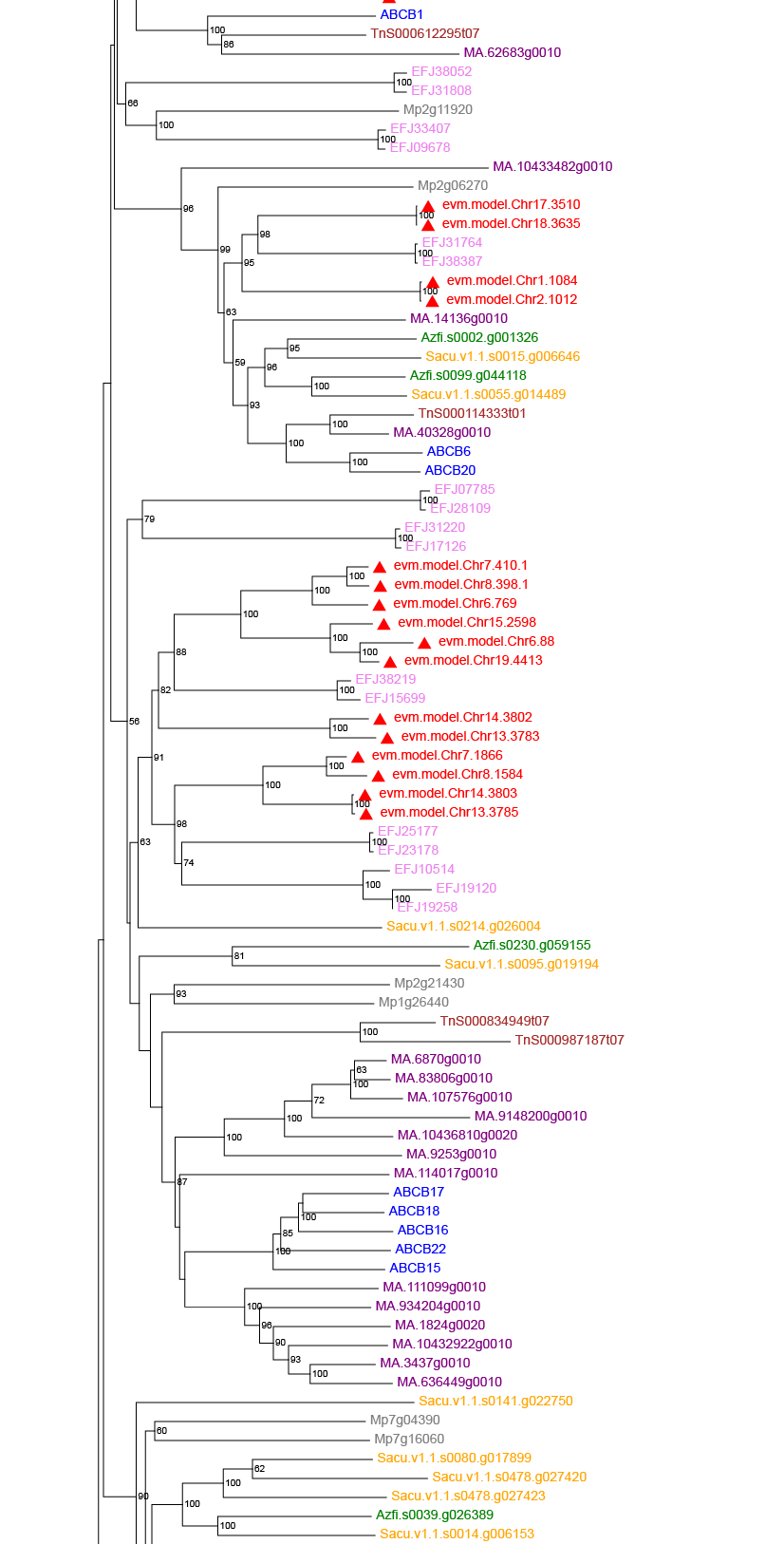

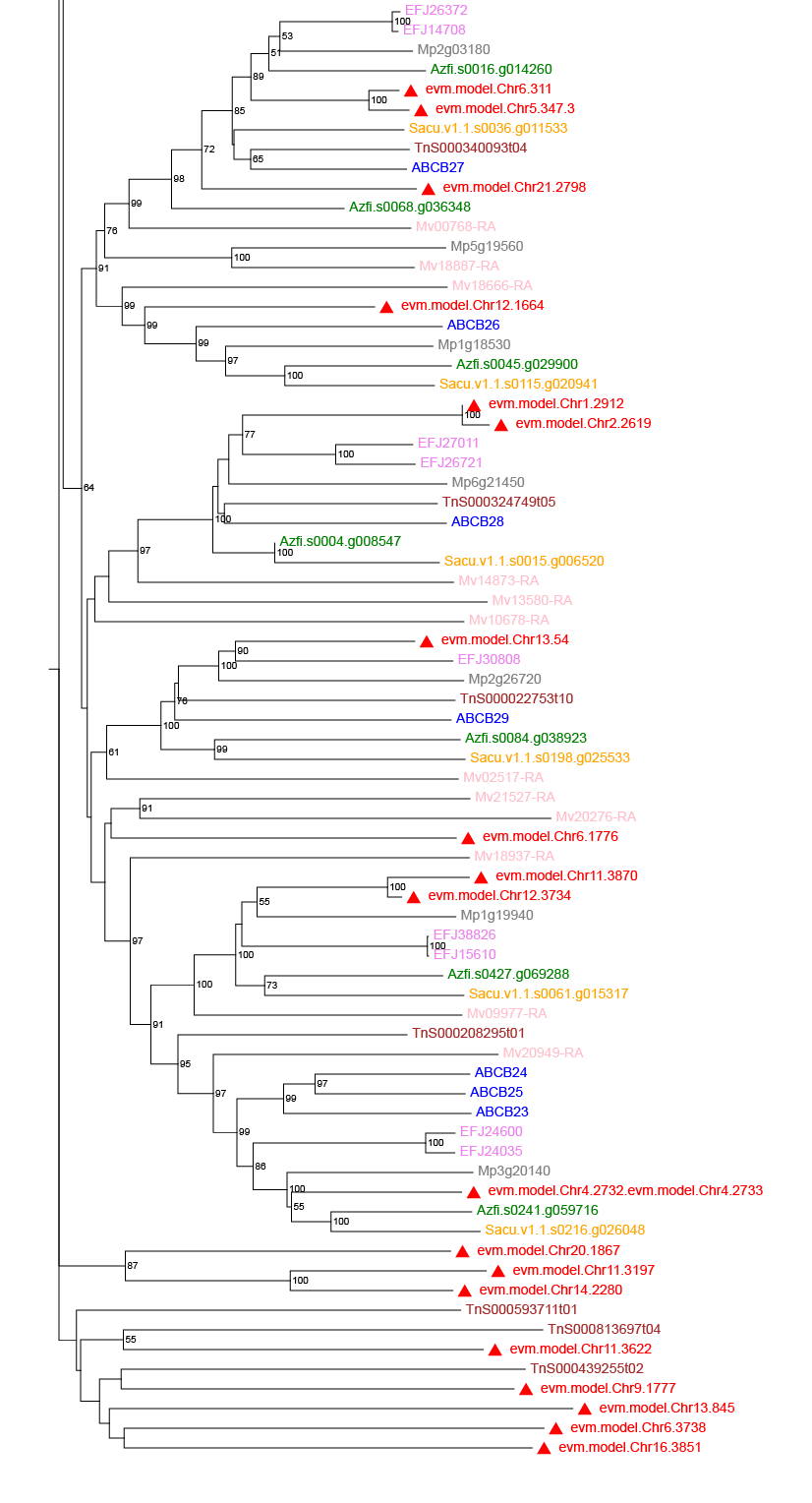

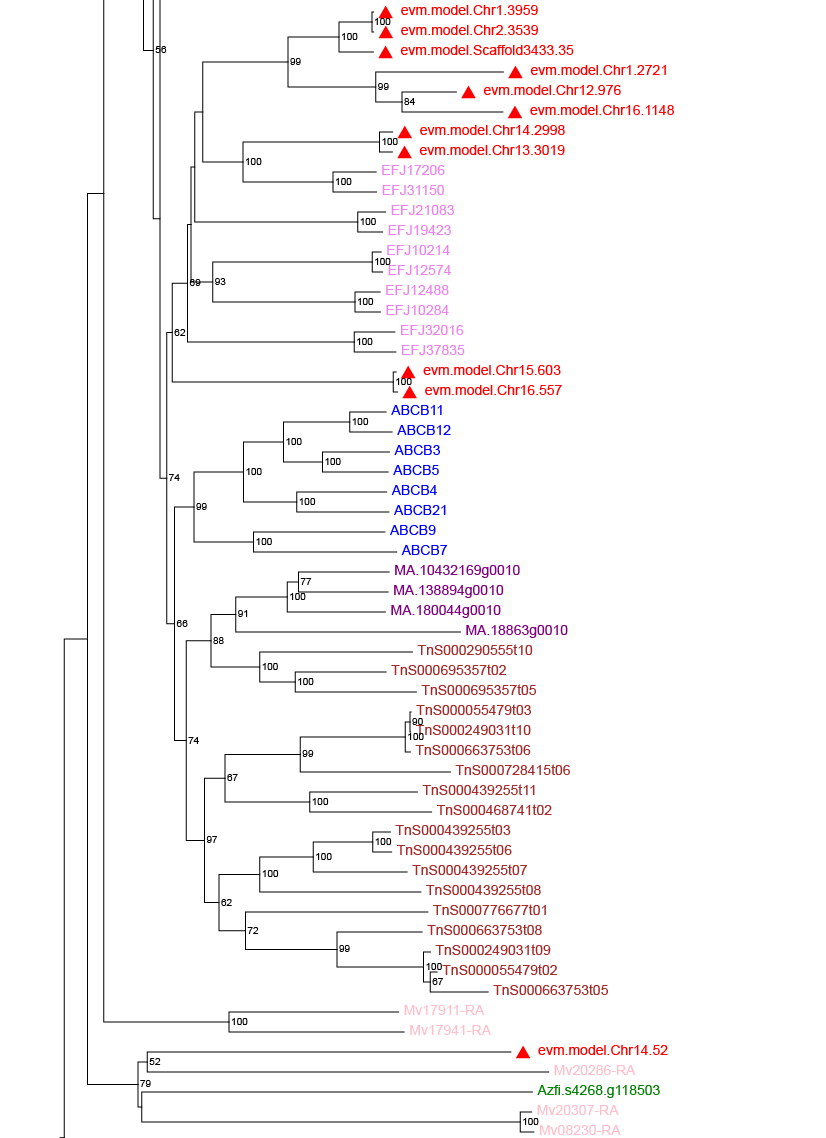


**Dataset S5. Phylogenetic relationships of ABCB proteins from *I. sinensis* and other evolutionarily representative species.** Numbers on the major branches indicate bootstrap values (> 50%) in 1,000 replicates.


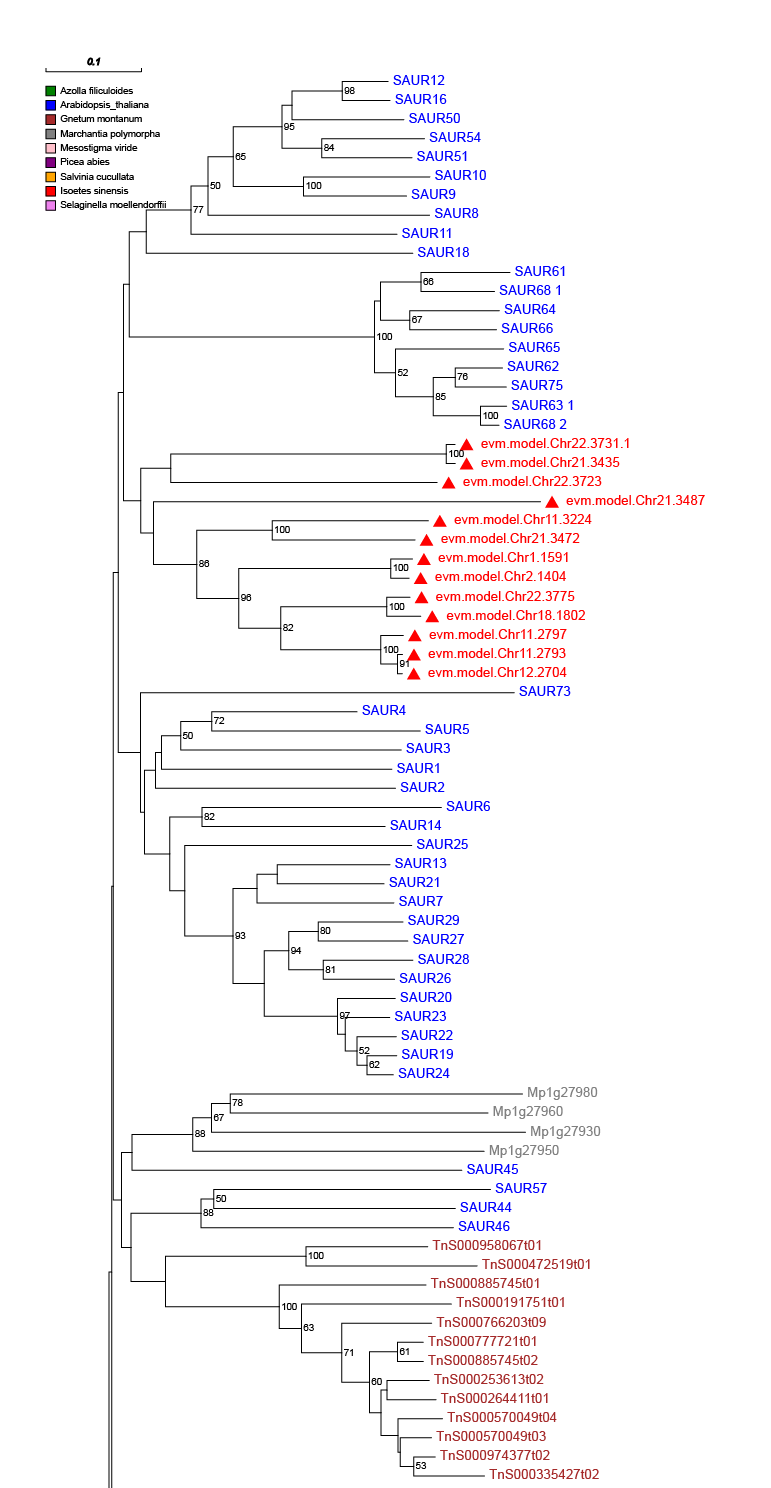

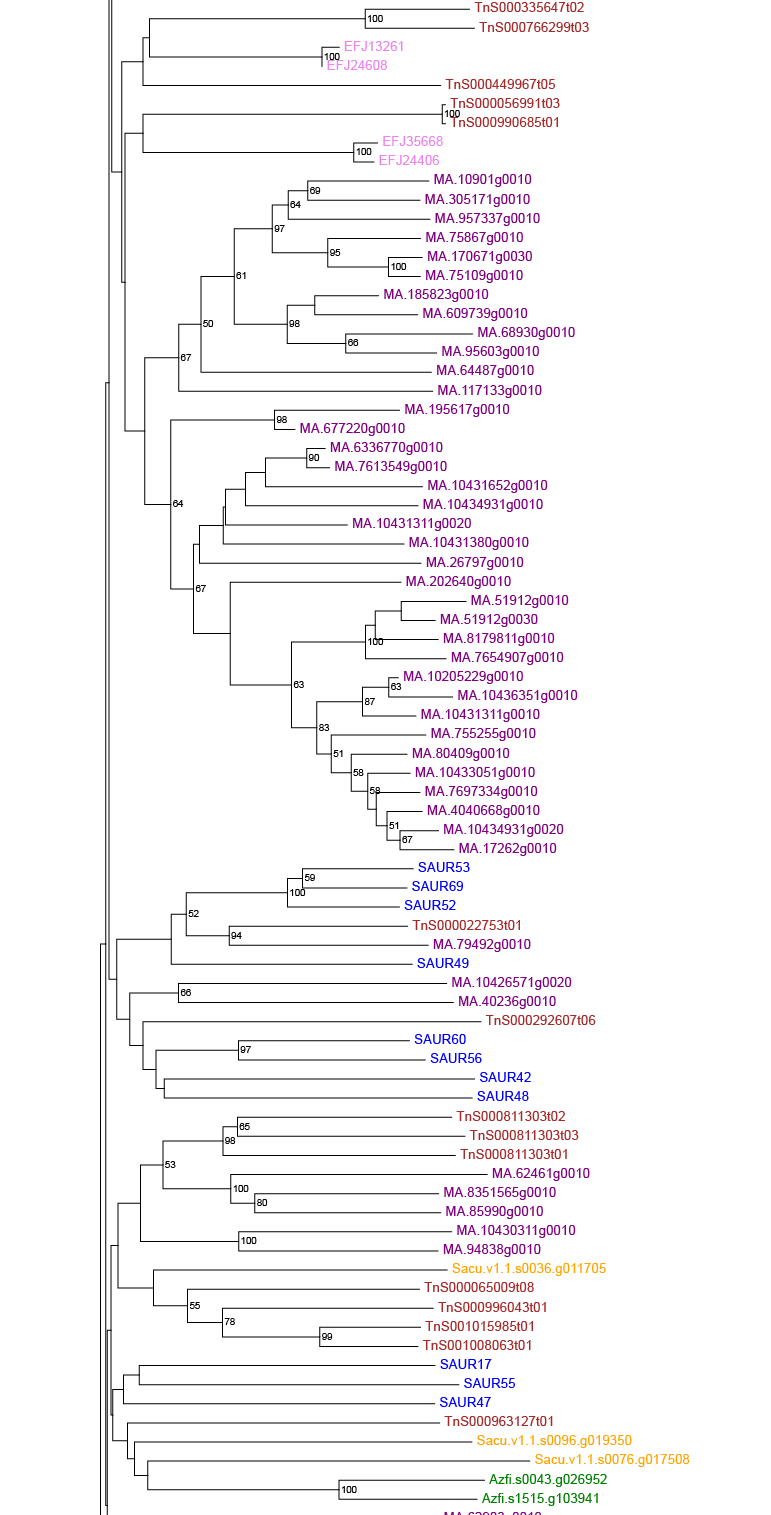

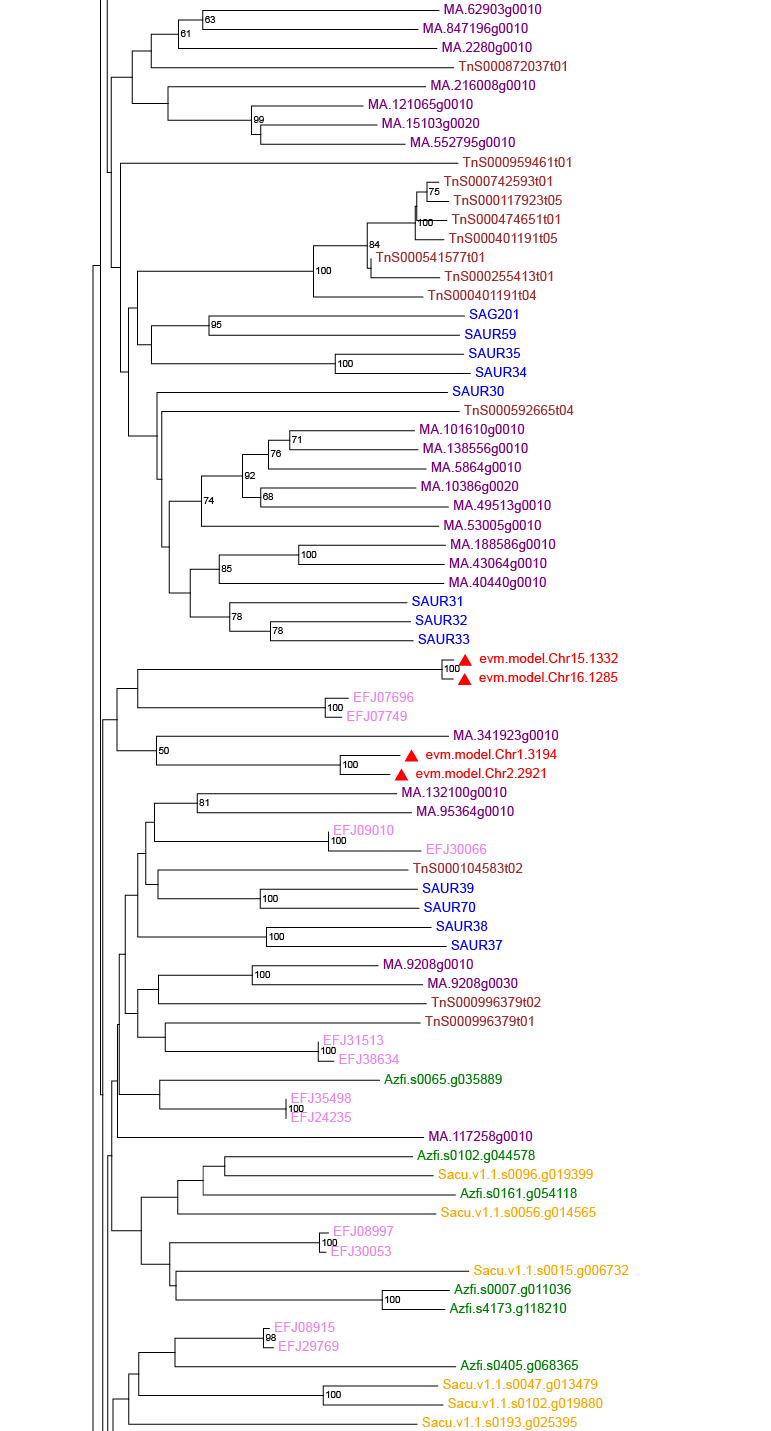

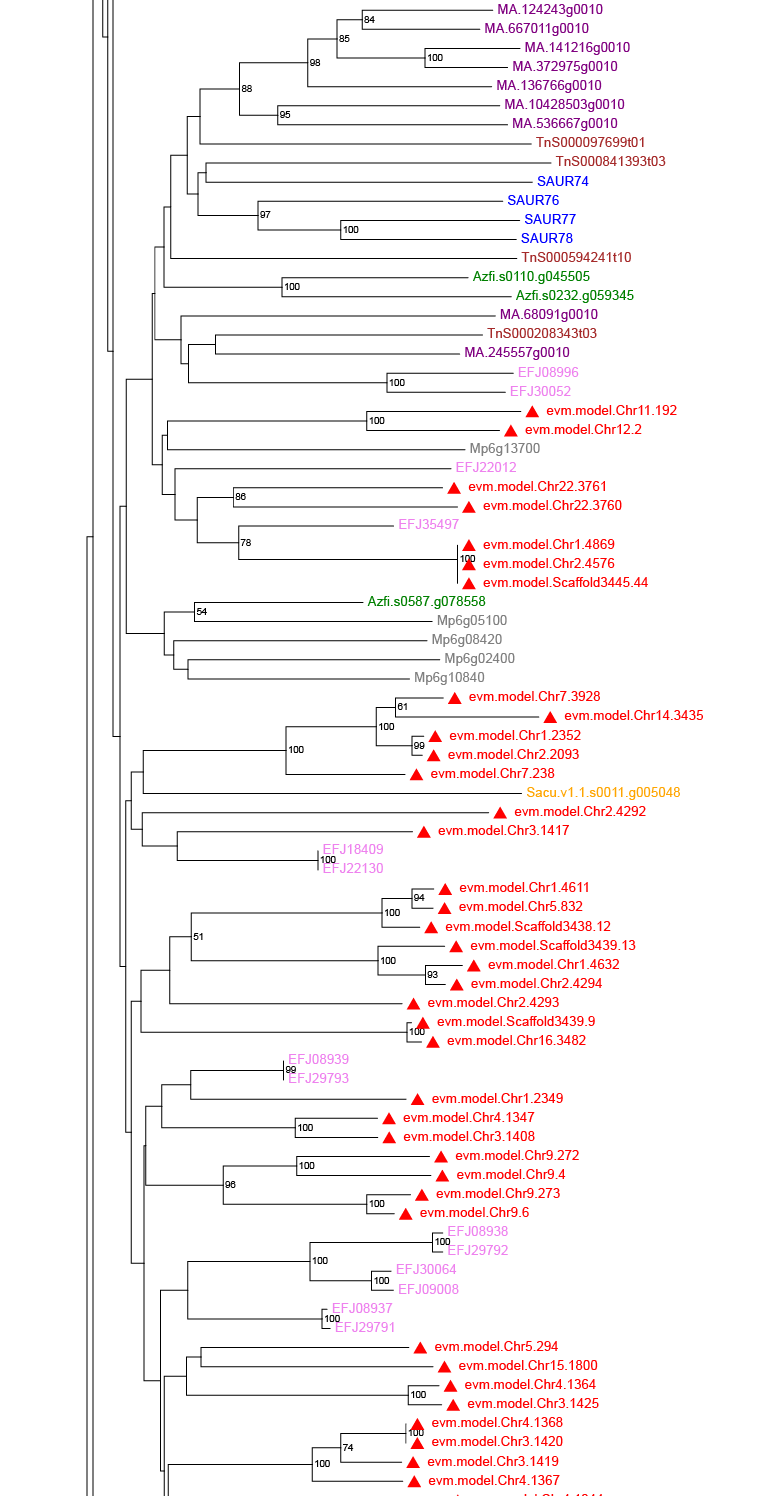

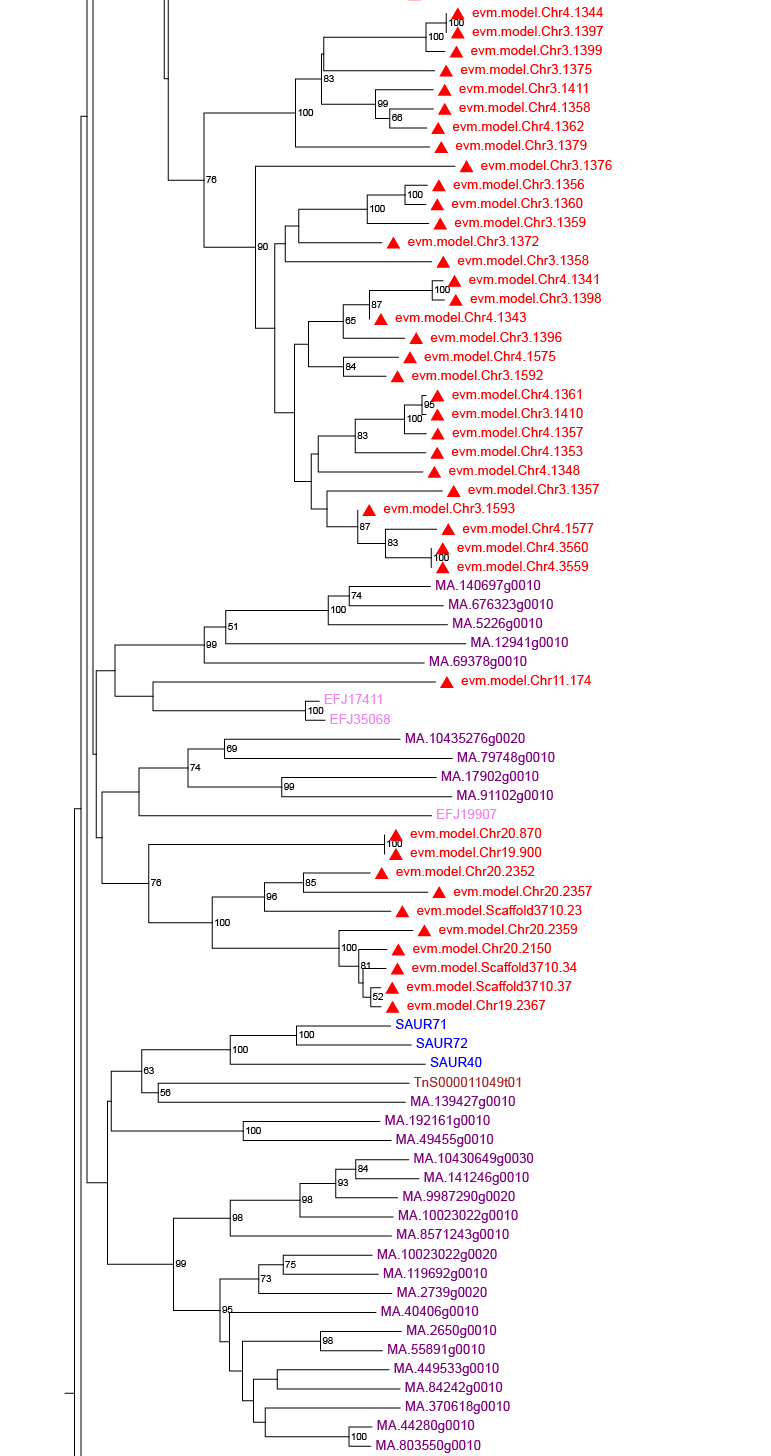

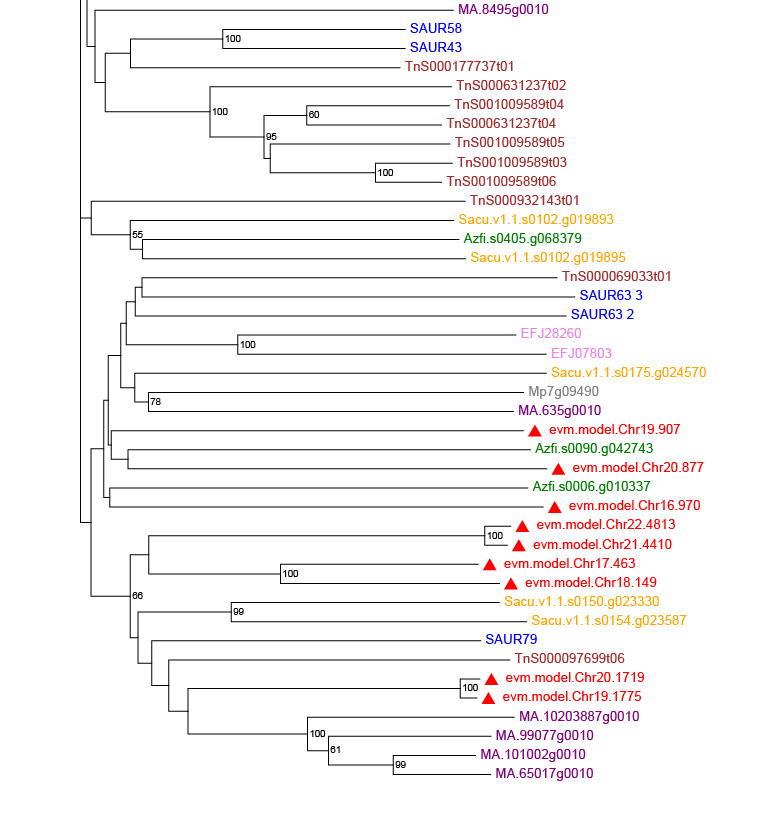


**Dataset S6. Phylogenetic relationships of SAUR proteins from *I. sinensis* and other evolutionarily representative species.** Numbers on the major branches indicate bootstrap values (> 50%) in 1,000 replicates.


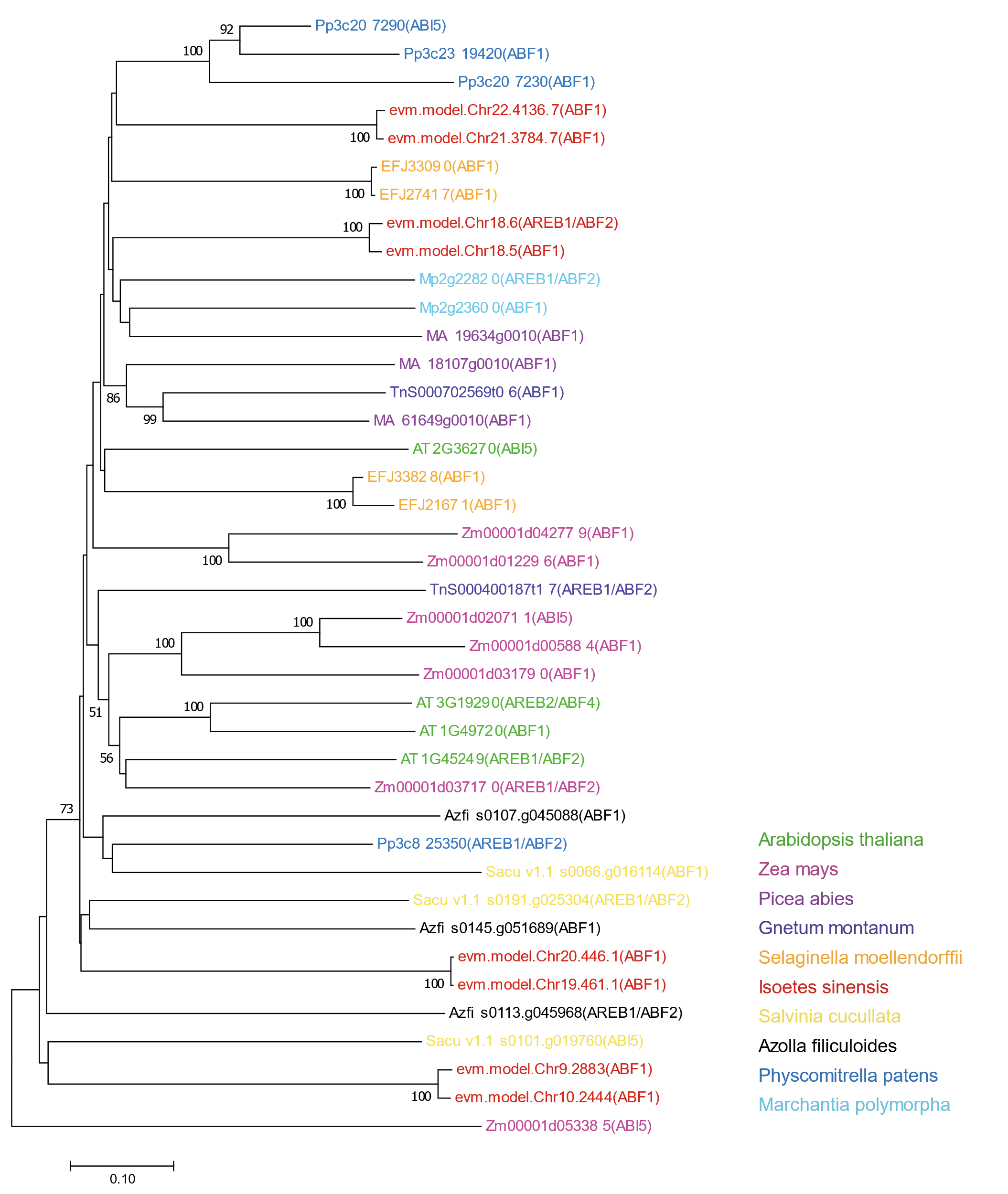


**Dataset S7. Phylogenetic relationships of ABF and AREB proteins from *I. sinensis* and other evolutionarily representative species.** Numbers on the major branches indicate bootstrap values (> 50%) in 1,000 replicates.


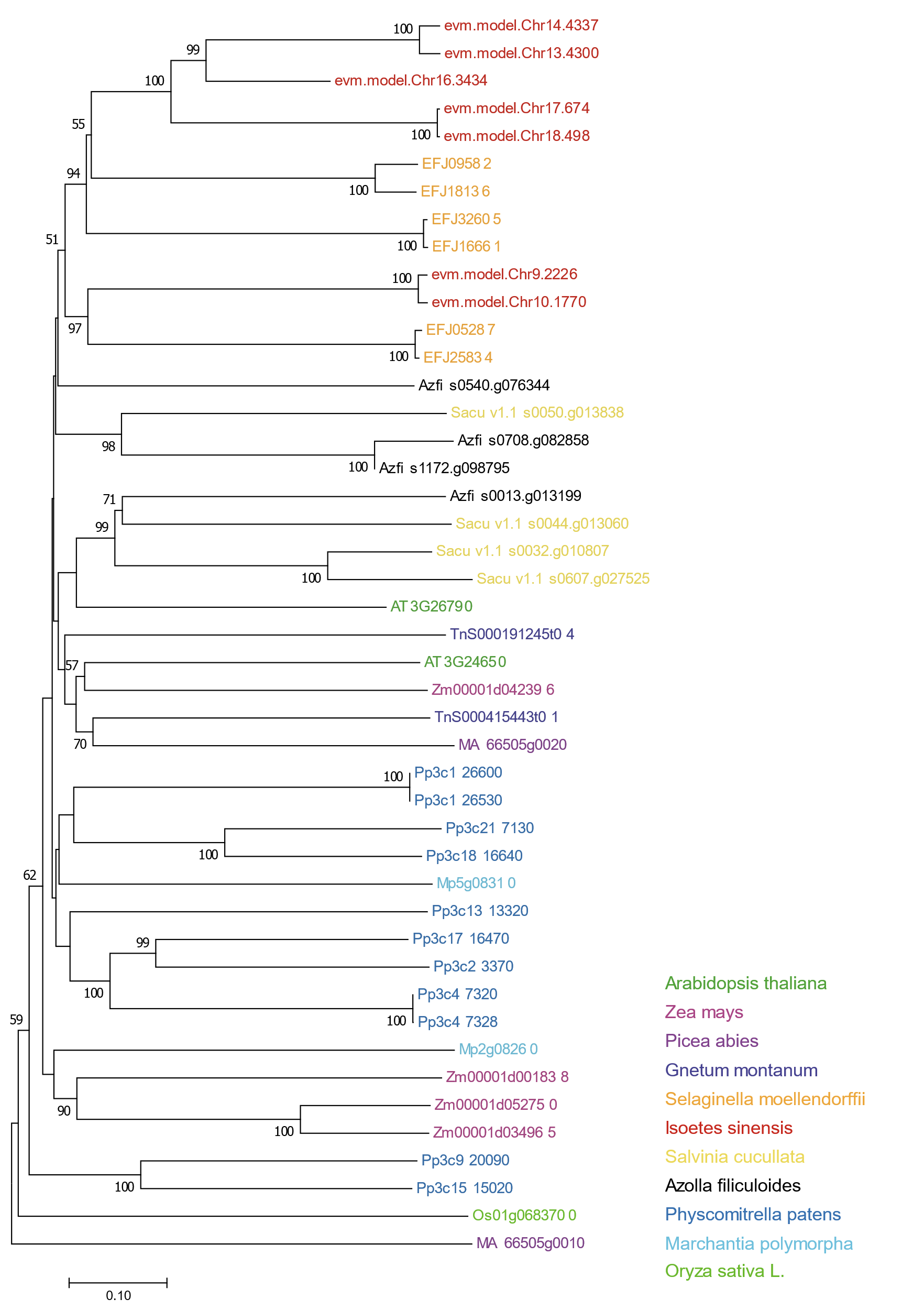


**Dataset S8. Phylogenetic relationships of ABI3 proteins from *I. sinensis* and other evolutionarily representative species.** Numbers on the major branches indicate bootstrap values (> 50%) in 1,000 replicates.


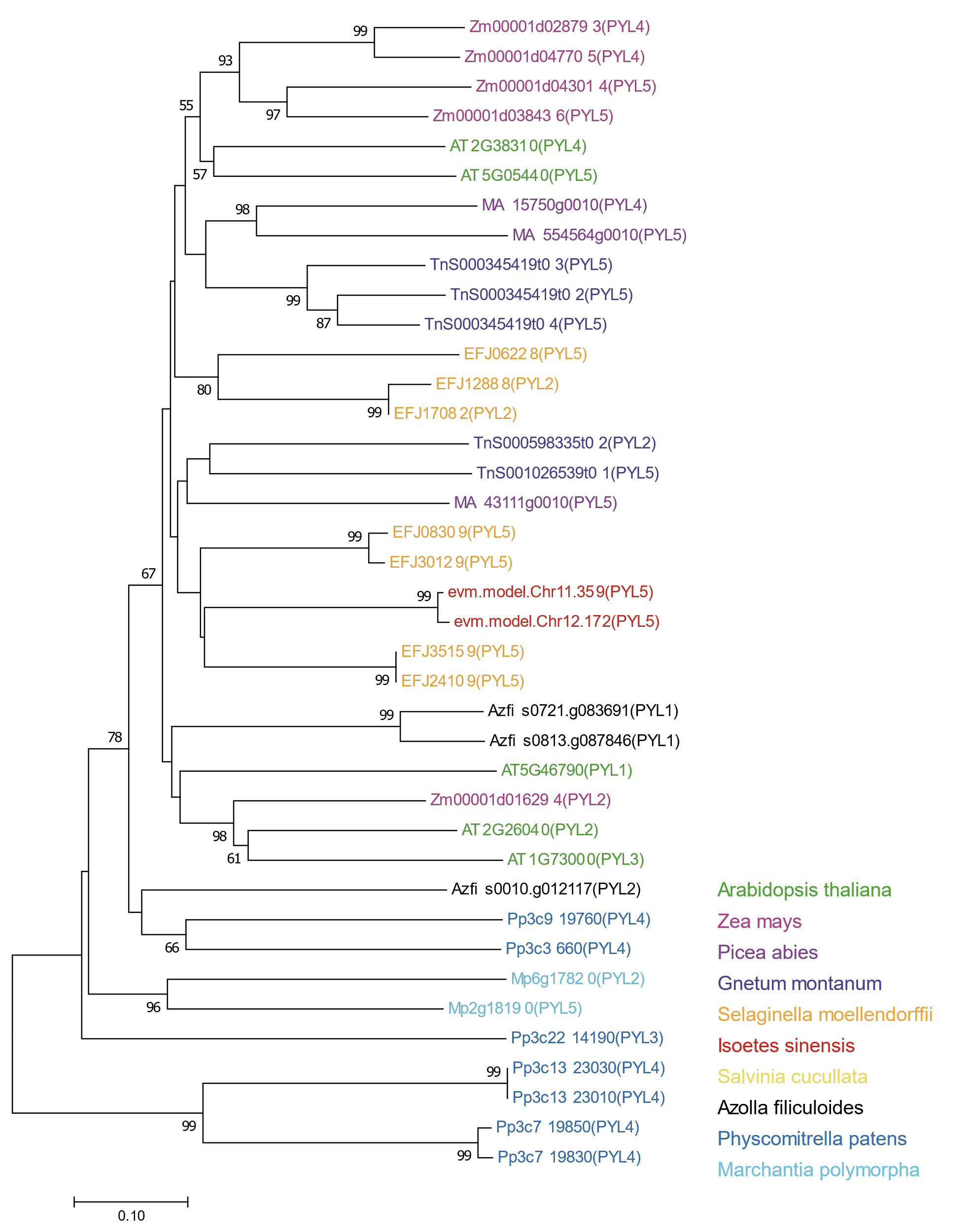


**Dataset S9. Phylogenetic relationships of PYL proteins from *I. sinensis* and other evolutionarily representative species.** Numbers on the major branches indicate bootstrap values (> 50%) in 1,000 replicates.


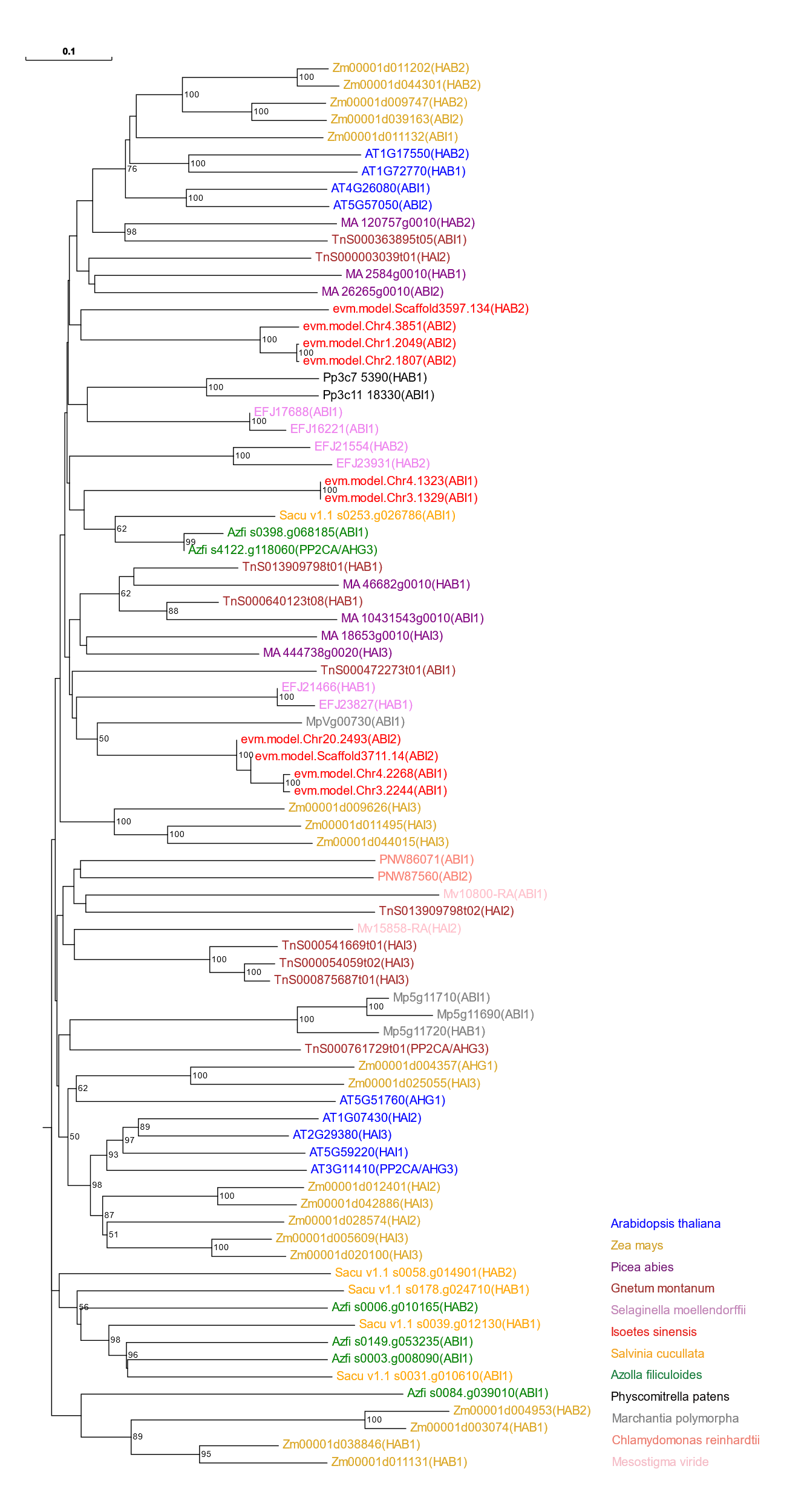


**Dataset S10. Phylogenetic relationships of PP2C proteins from *I. sinensis* and other evolutionarily representative species.** Numbers on the major branches indicate bootstrap values (> 50%) in 1,000 replicates.


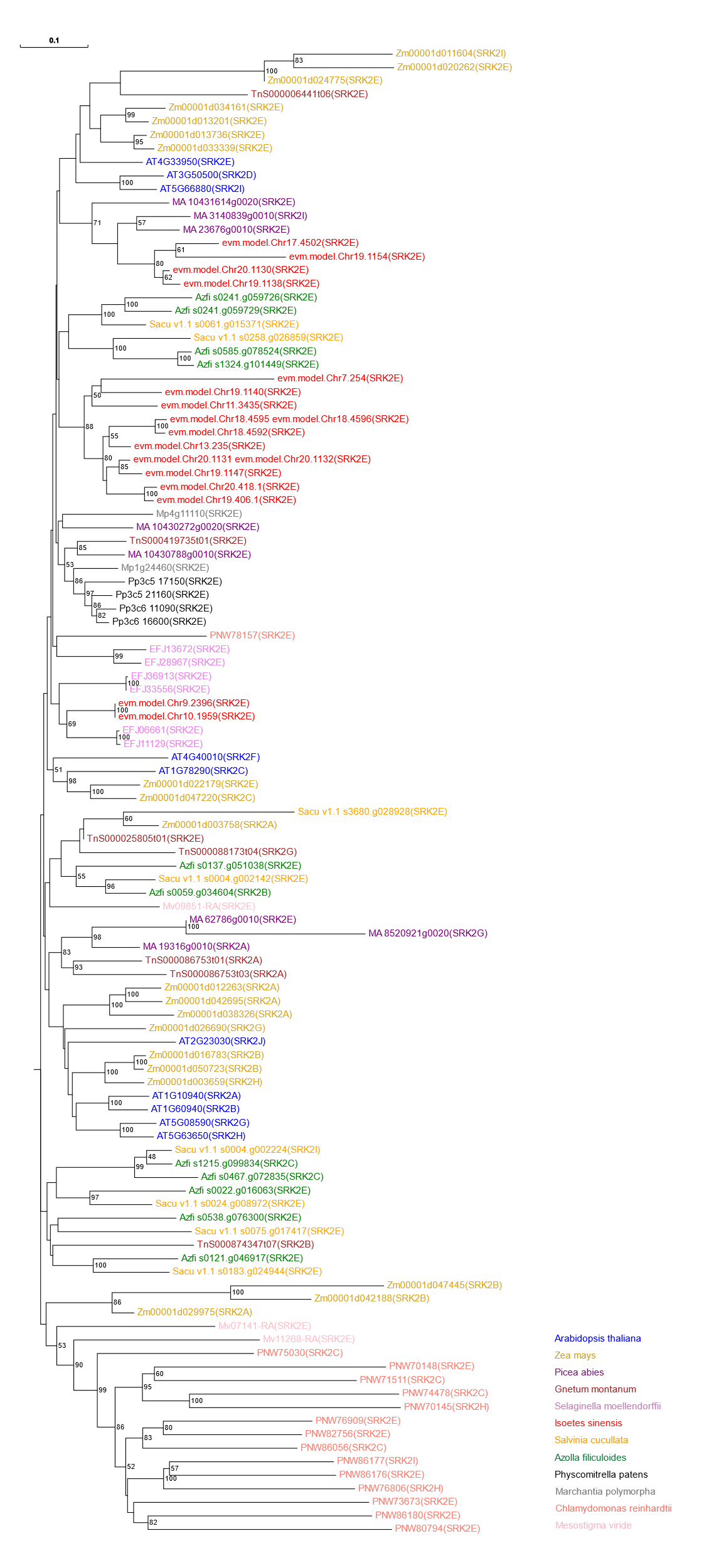


**Dataset S11. Phylogenetic relationships of SnRK proteins from *I. sinensis* and other evolutionarily representative species.** Numbers on the major branches indicate bootstrap values (> 50%) in 1,000 replicates.


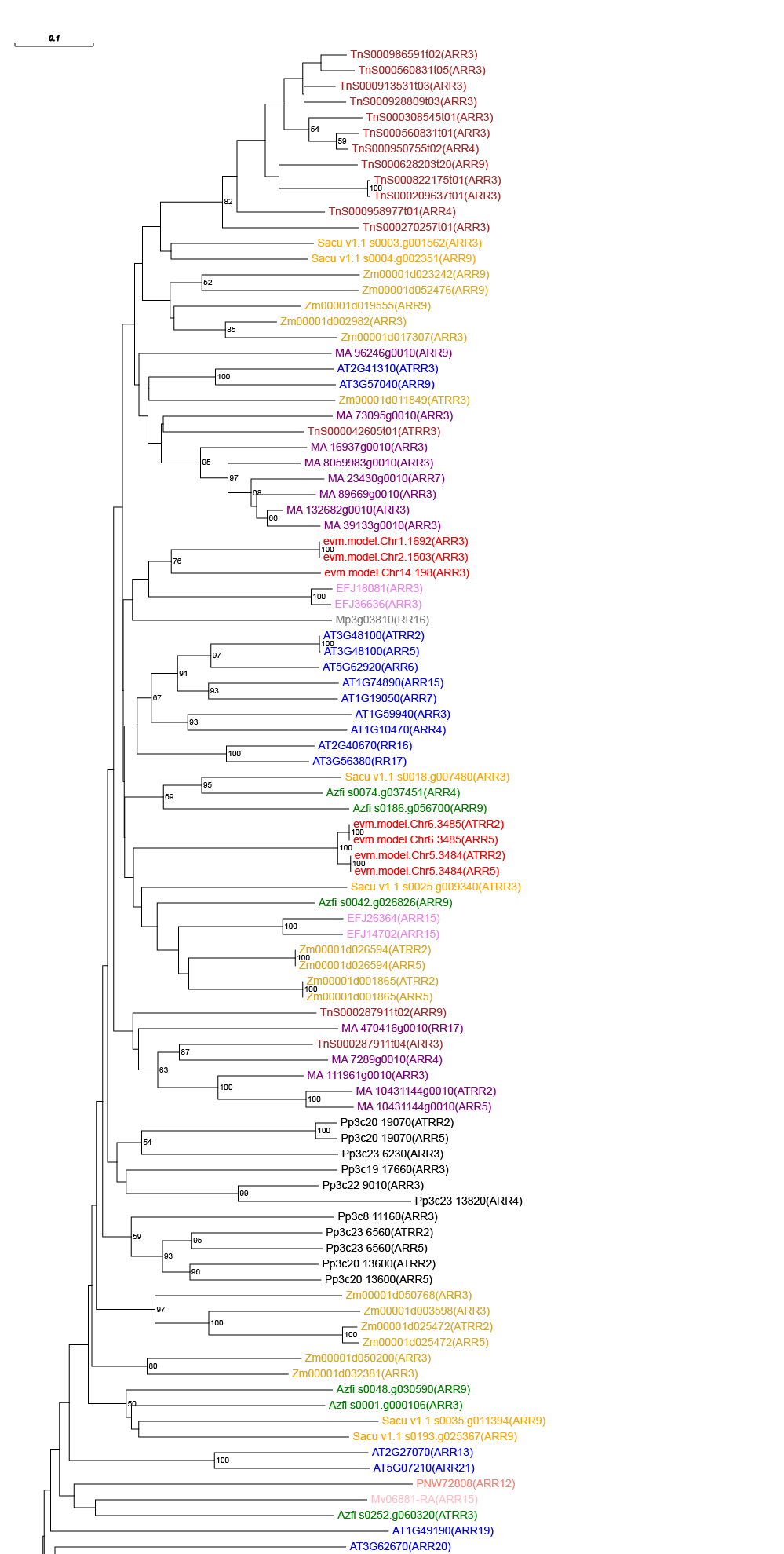


**
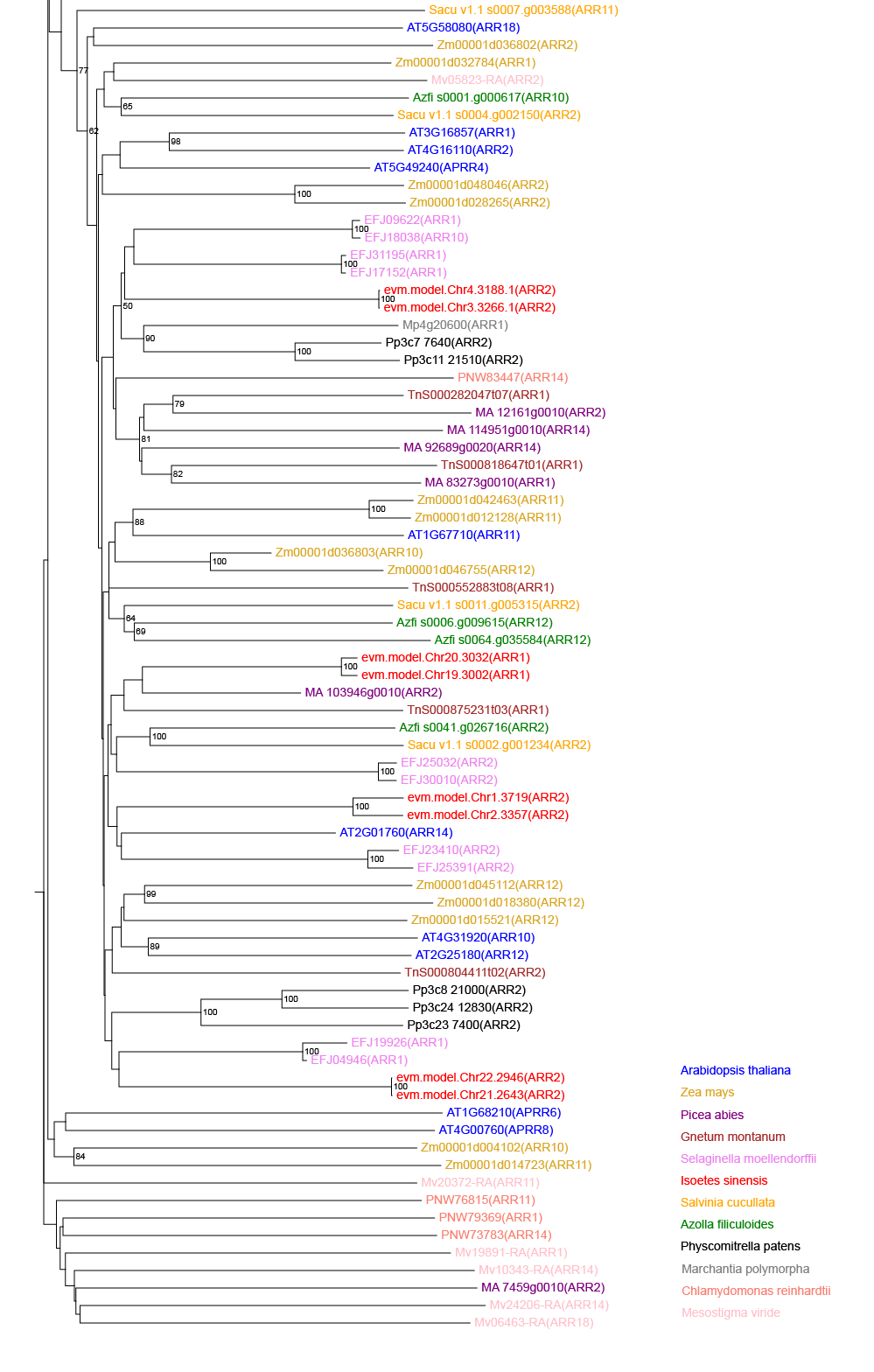
**

**Dataset S12.** **Phylogenetic relationships of ARR proteins from *I. sinensis* and other evolutionarily representative species.** Numbers on the major branches indicate bootstrap values (> 50%) in 1,000 replicates.


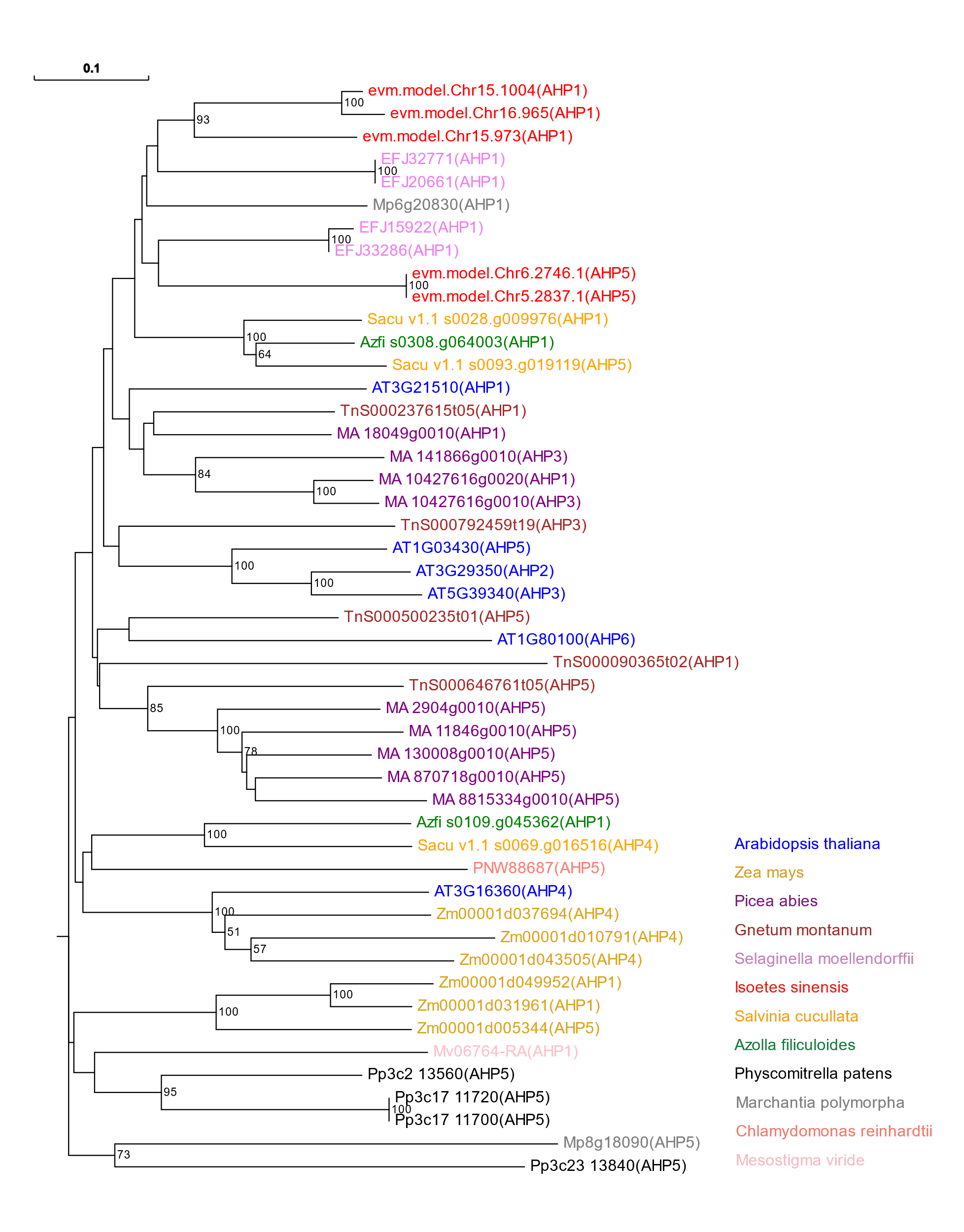


**Dataset S13.** **Phylogenetic relationships of AHP proteins from *I. sinensis* and other evolutionarily representative species.** Numbers on the major branches indicate bootstrap values (> 50%) in 1,000 replicates.


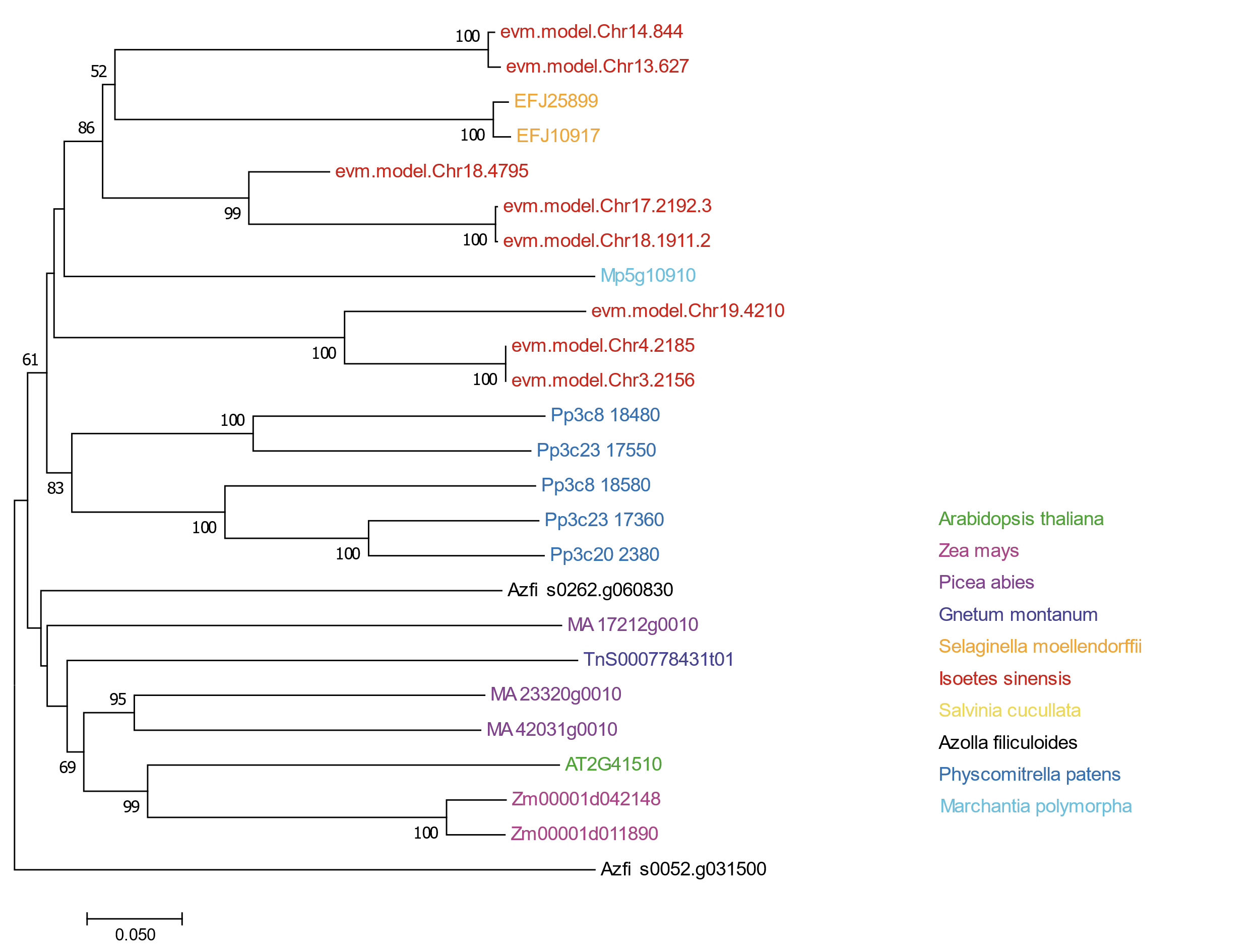


**Dataset S14.** **Phylogenetic relationships of CKX1 proteins from *I. sinensis* and other evolutionarily representative species.** Numbers on the major branches indicate bootstrap values (> 50%) in 1,000 replicates.


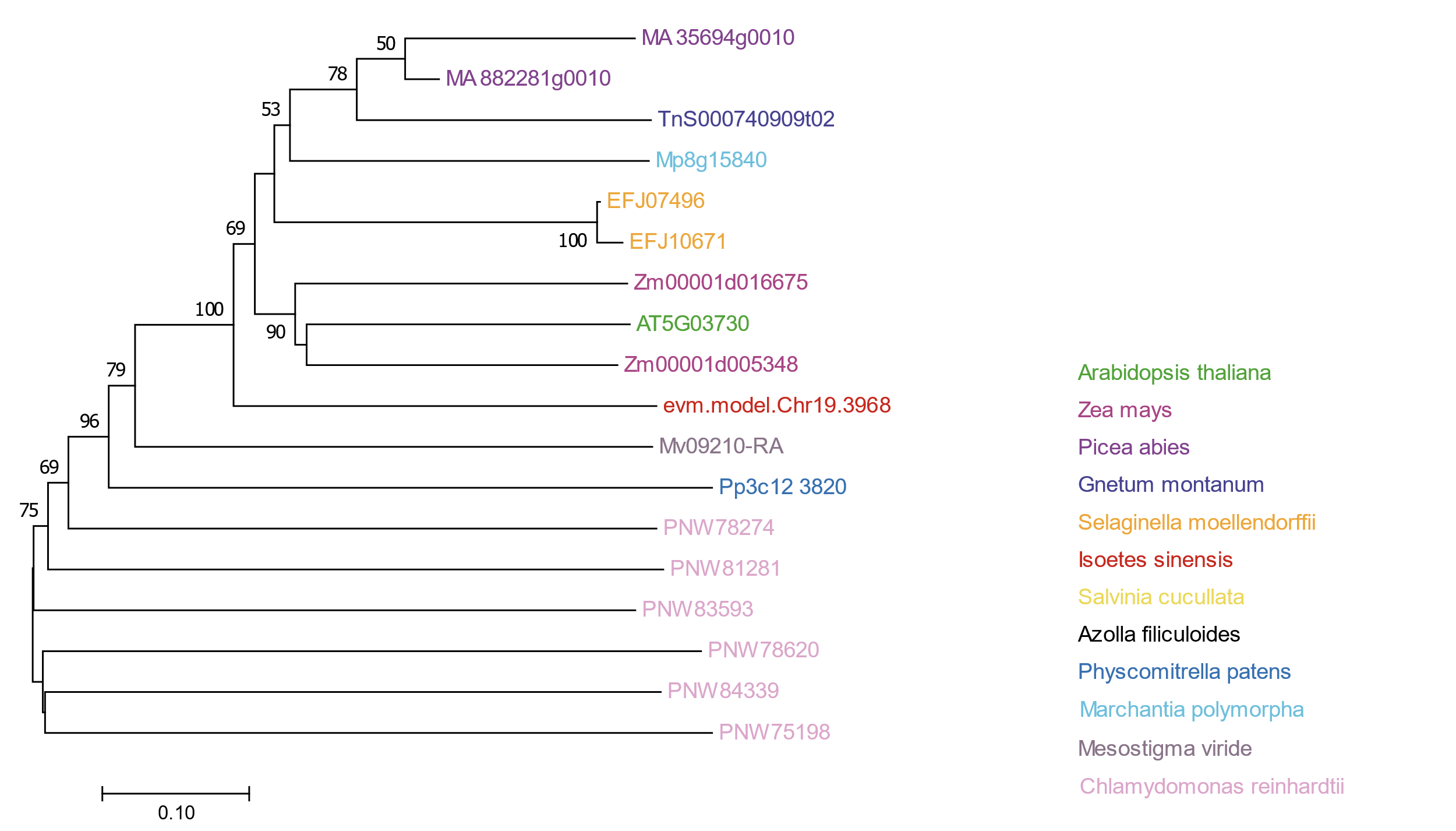


**Dataset S15. Phylogenetic relationships of CTR1 proteins from *I. sinensis* and other evolutionarily representative species.** Numbers on the major branches indicate bootstrap values (> 50%) in 1,000 replicates.


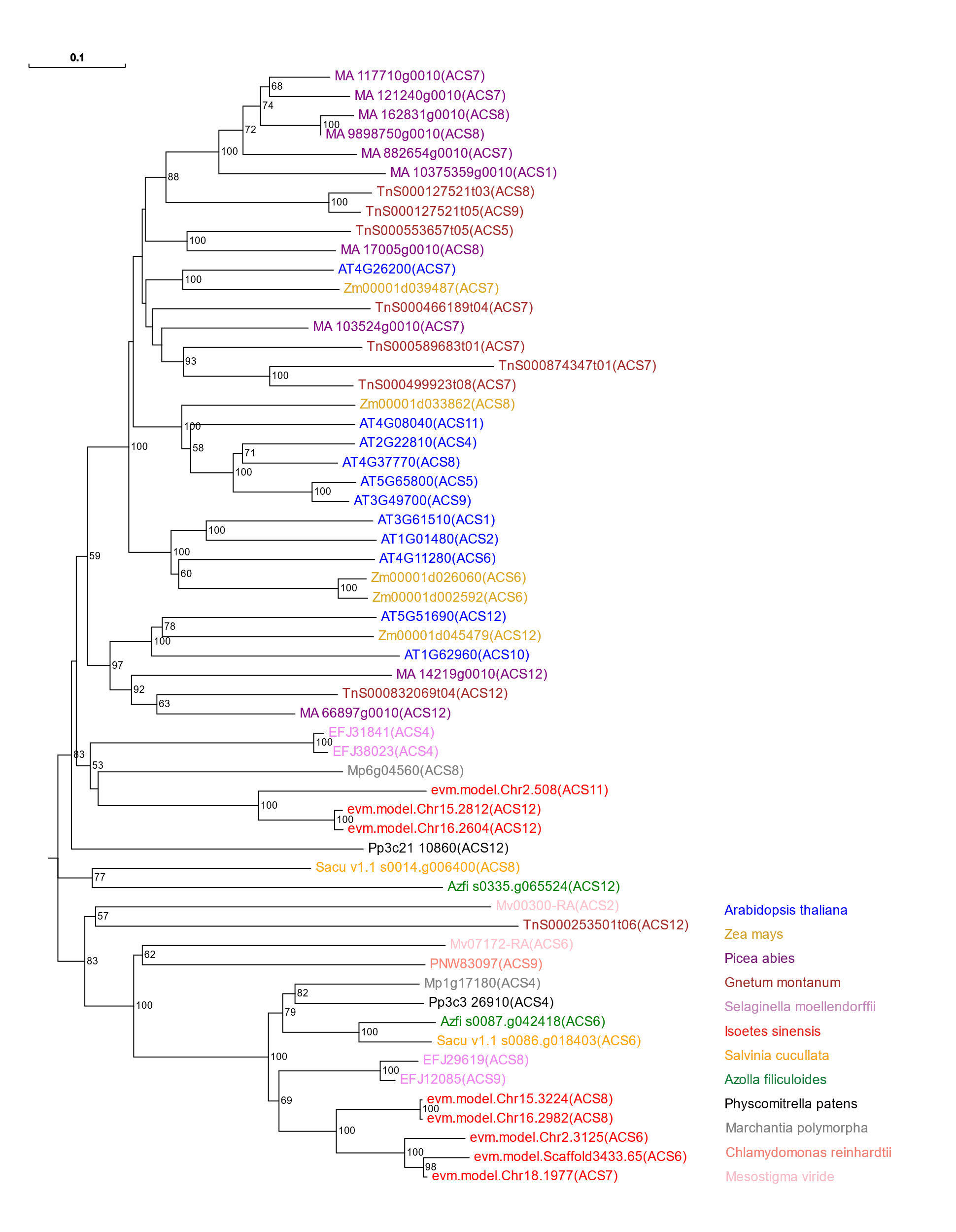


**Dataset S16. Phylogenetic relationships of ACS proteins from *I. sinensis* and other evolutionarily representative species.** Numbers on the major branches indicate bootstrap values (> 50%) in 1,000 replicates.


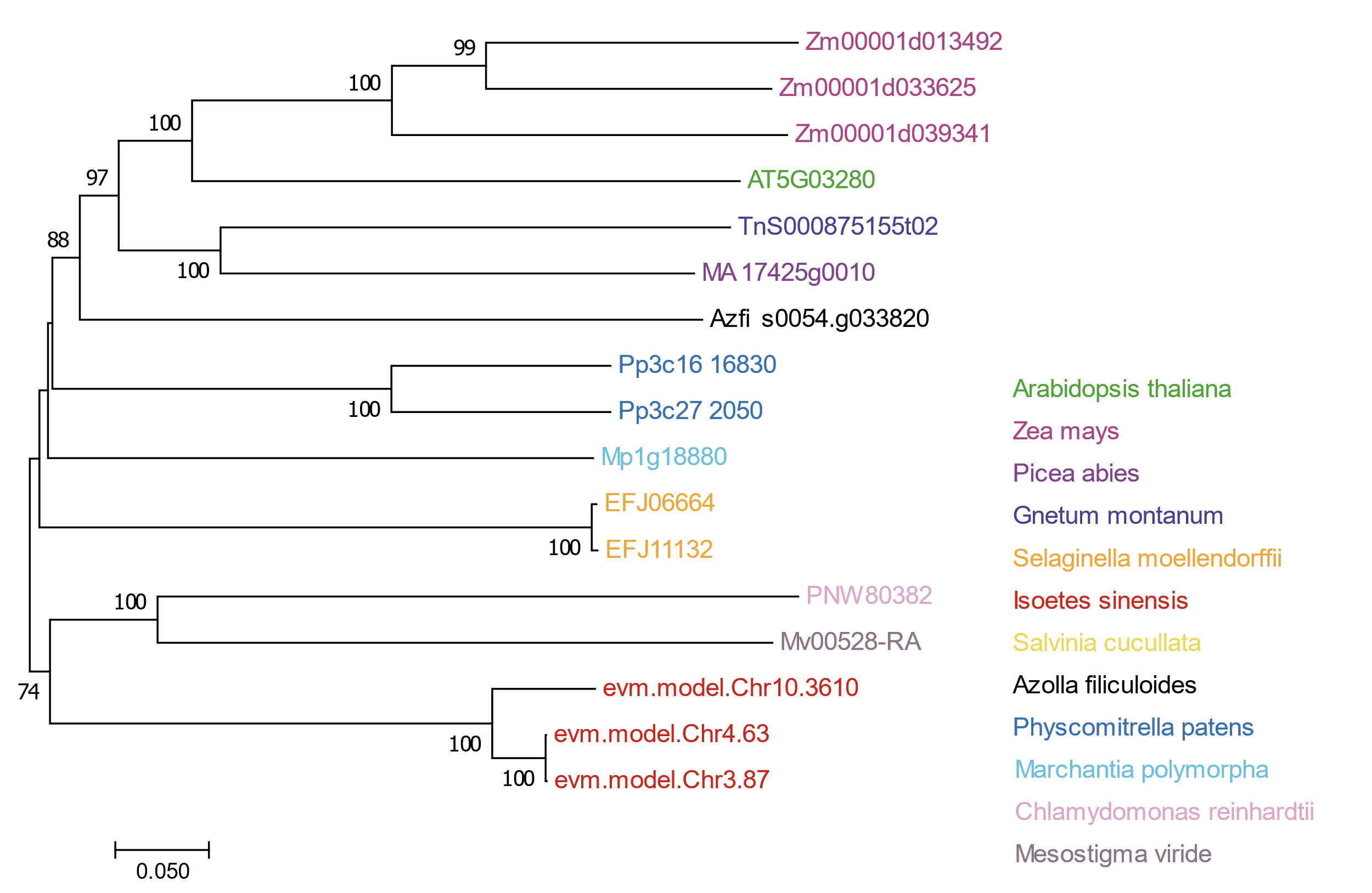


**Dataset S17. Phylogenetic relationships of EIN2 proteins from *I. sinensis* and other evolutionarily representative species.** Numbers on the major branches indicate bootstrap values (> 50%) in 1,000 replicates.


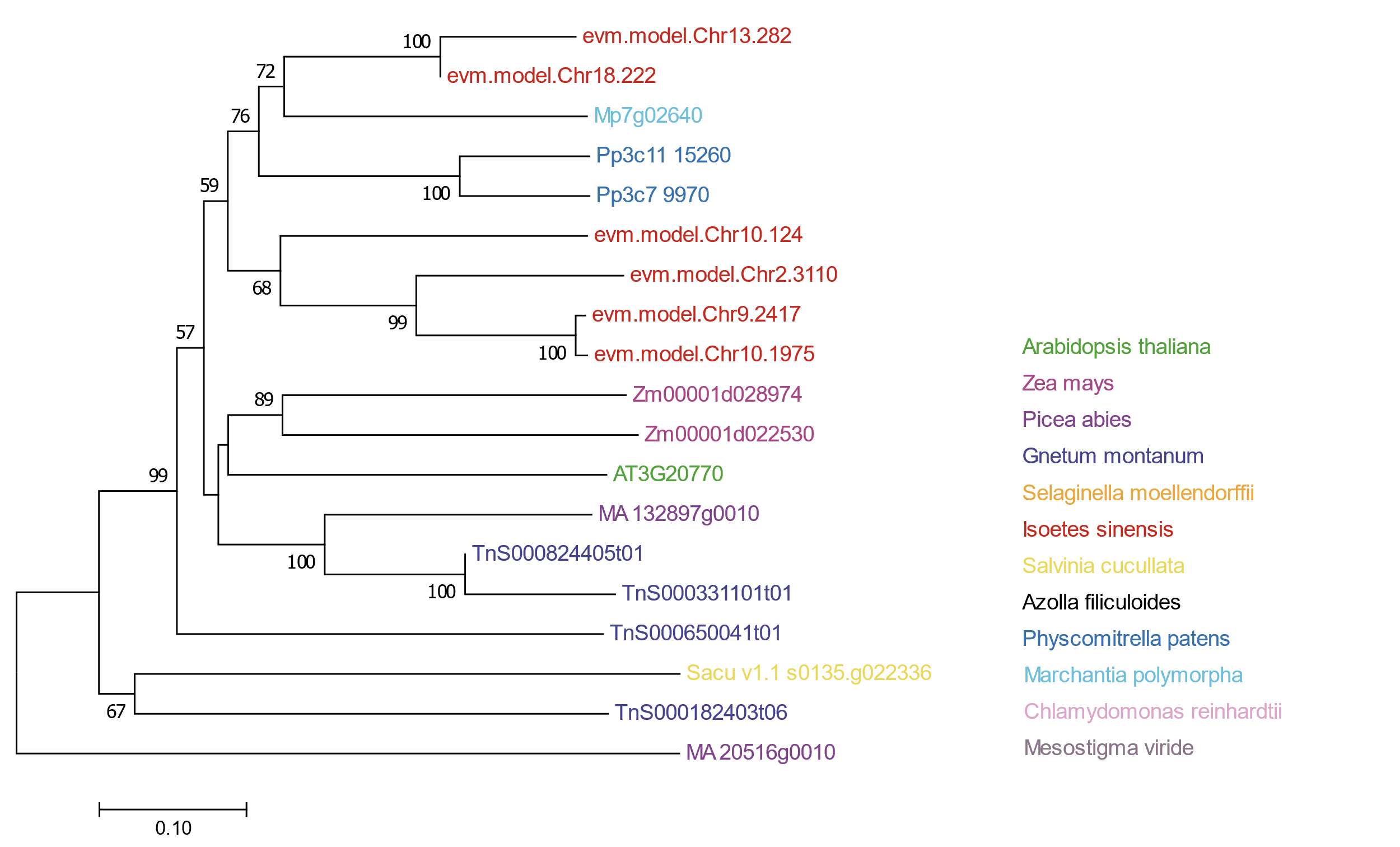


**Dataset S18. Phylogenetic relationships of EIN3 proteins from *I. sinensis* and other evolutionarily representative species.** Numbers on the major branches indicate bootstrap values (> 50%) in 1,000 replicates.


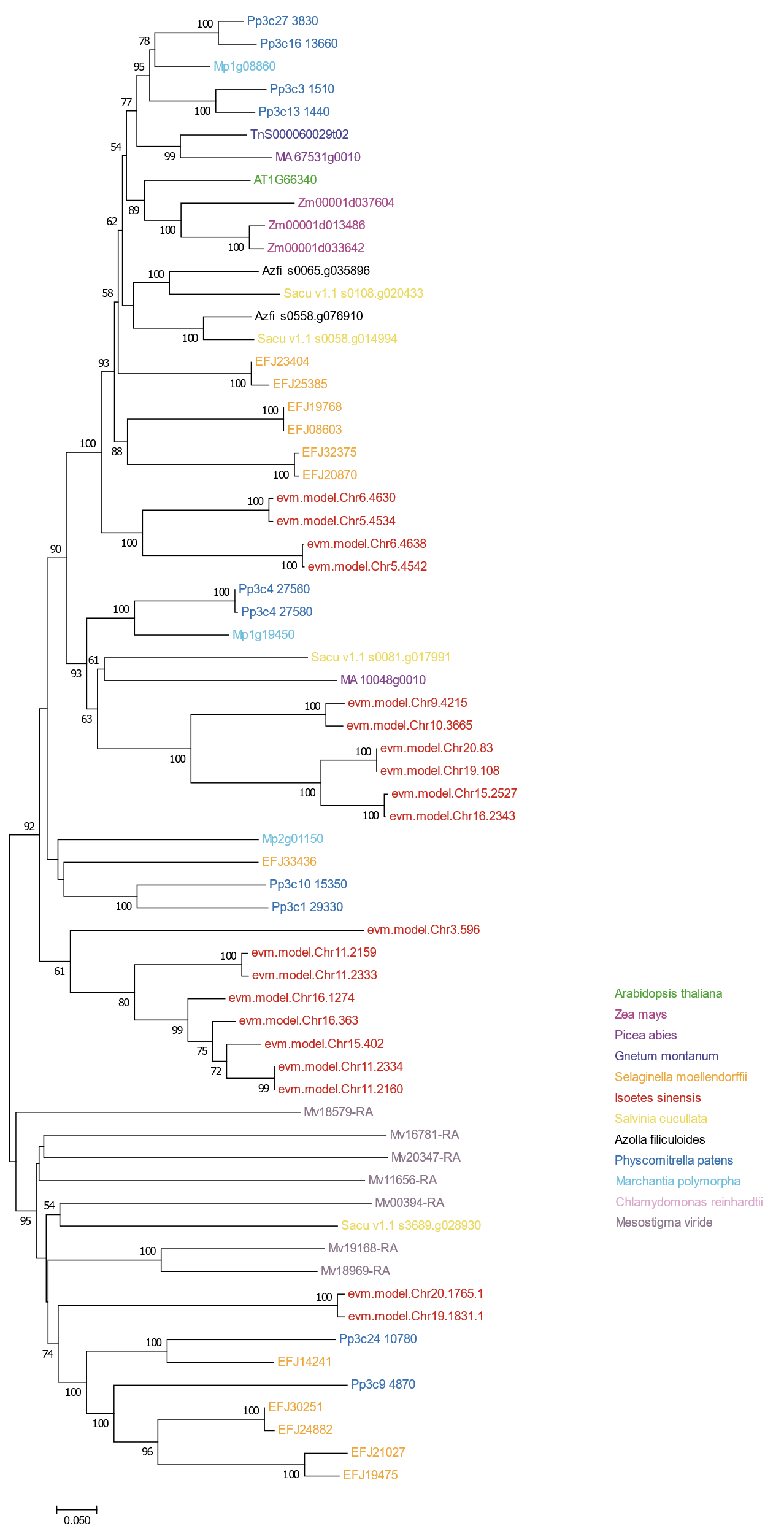


**Dataset S19. Phylogenetic relationships of ETR1 proteins from *I. sinensis* and other evolutionarily representative species.** Numbers on the major branches indicate bootstrap values (> 50%) in 1,000 replicates.


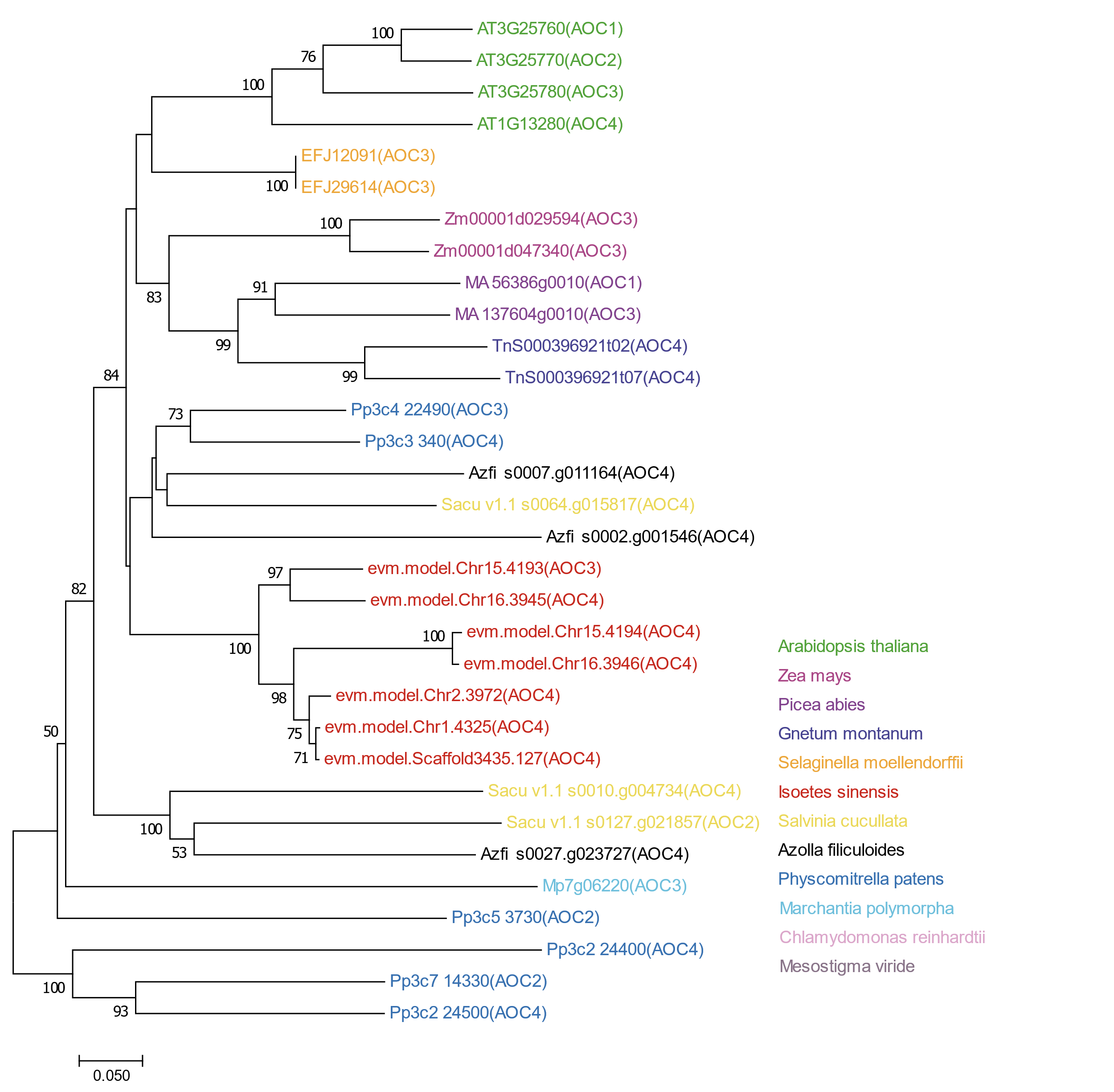


**Dataset S20. Phylogenetic relationships of AOC proteins from *I. sinensis* and other evolutionarily representative species.** Numbers on the major branches indicate bootstrap values (> 50%) in 1,000 replicates.


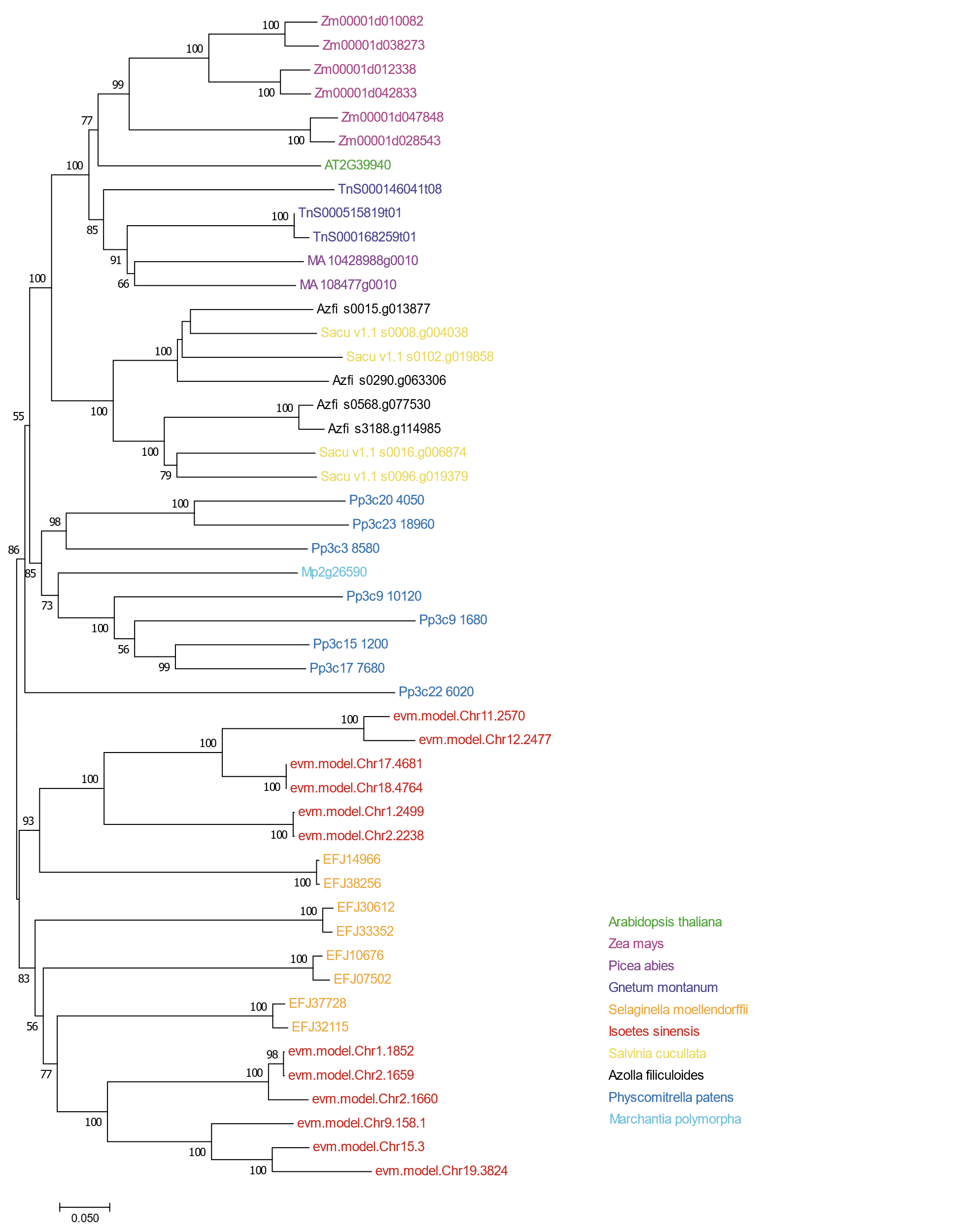


**Dataset S21. Phylogenetic relationships of COL1 proteins from *I. sinensis* and other evolutionarily representative species.** Numbers on the major branches indicate bootstrap values (> 50%) in 1,000 replicates.


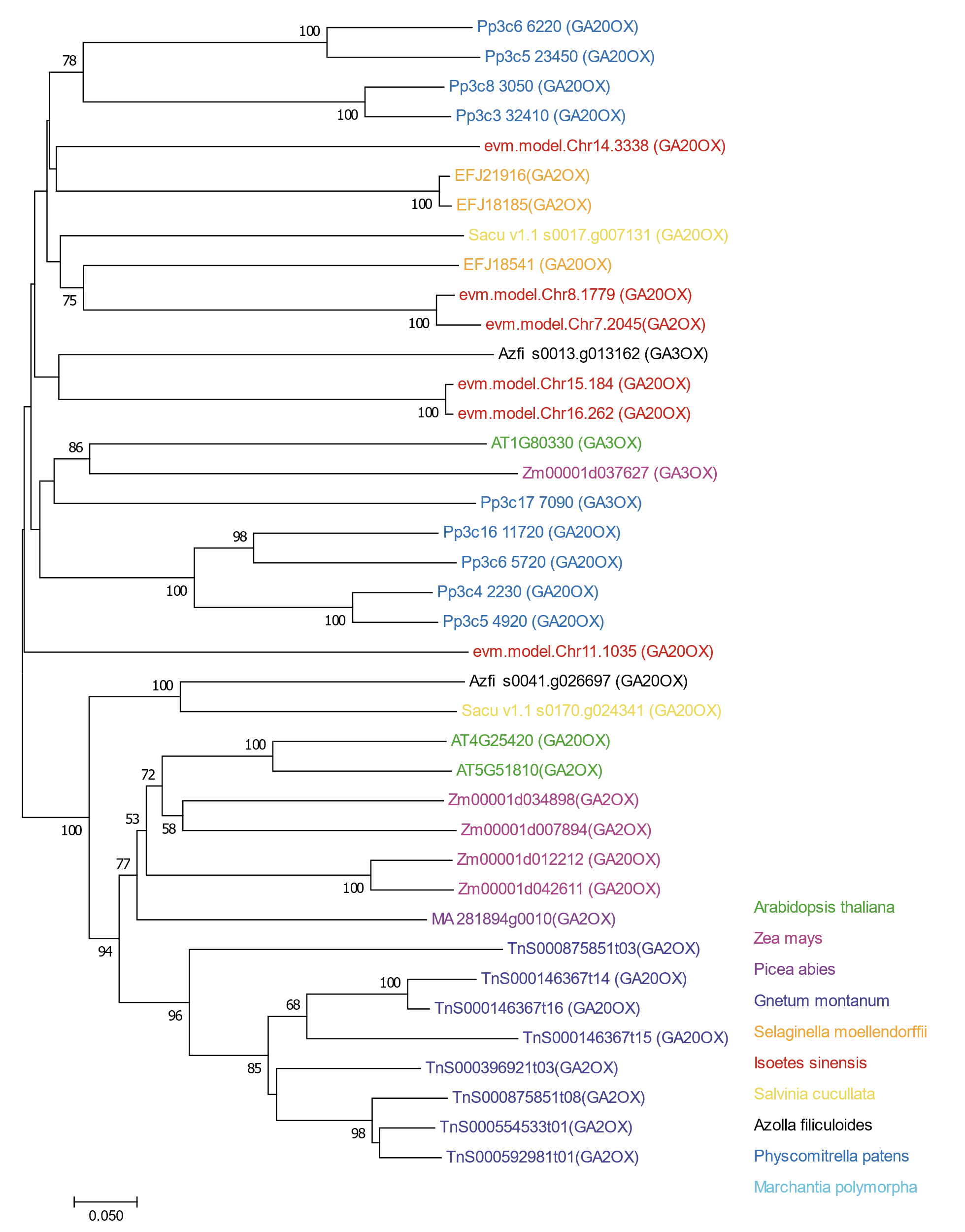


**Dataset S22. Phylogenetic relationships of gibberellin related proteins from *I. sinensis* and other evolutionarily representative species.** Numbers on the major branches indicate bootstrap values (> 50%) in 1,000 replicates.


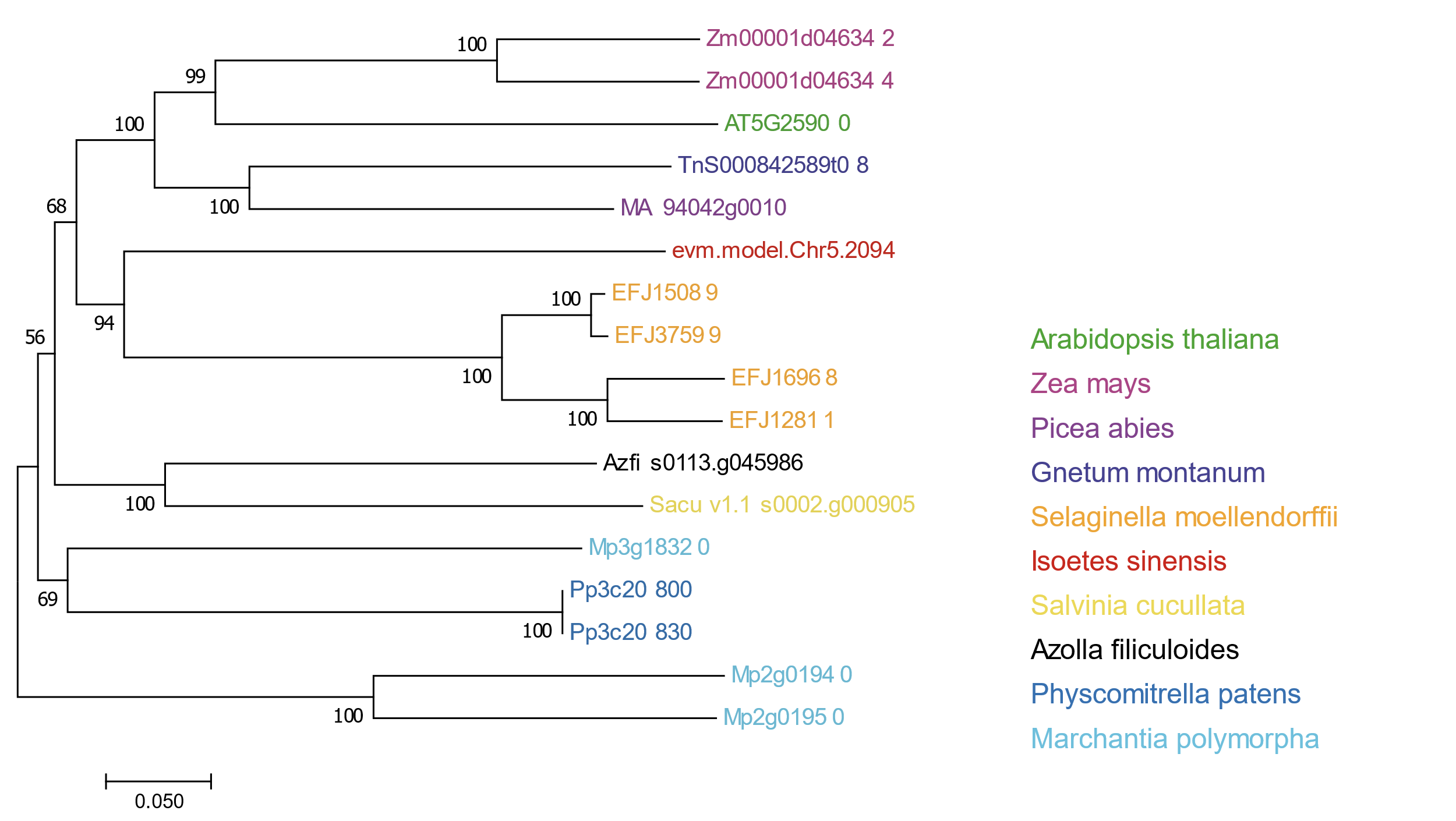


**Dataset S23. Phylogenetic relationships of KO proteins from *I. sinensis* and other evolutionarily representative species.** Numbers on the major branches indicate bootstrap values (> 50%) in 1,000 replicates.


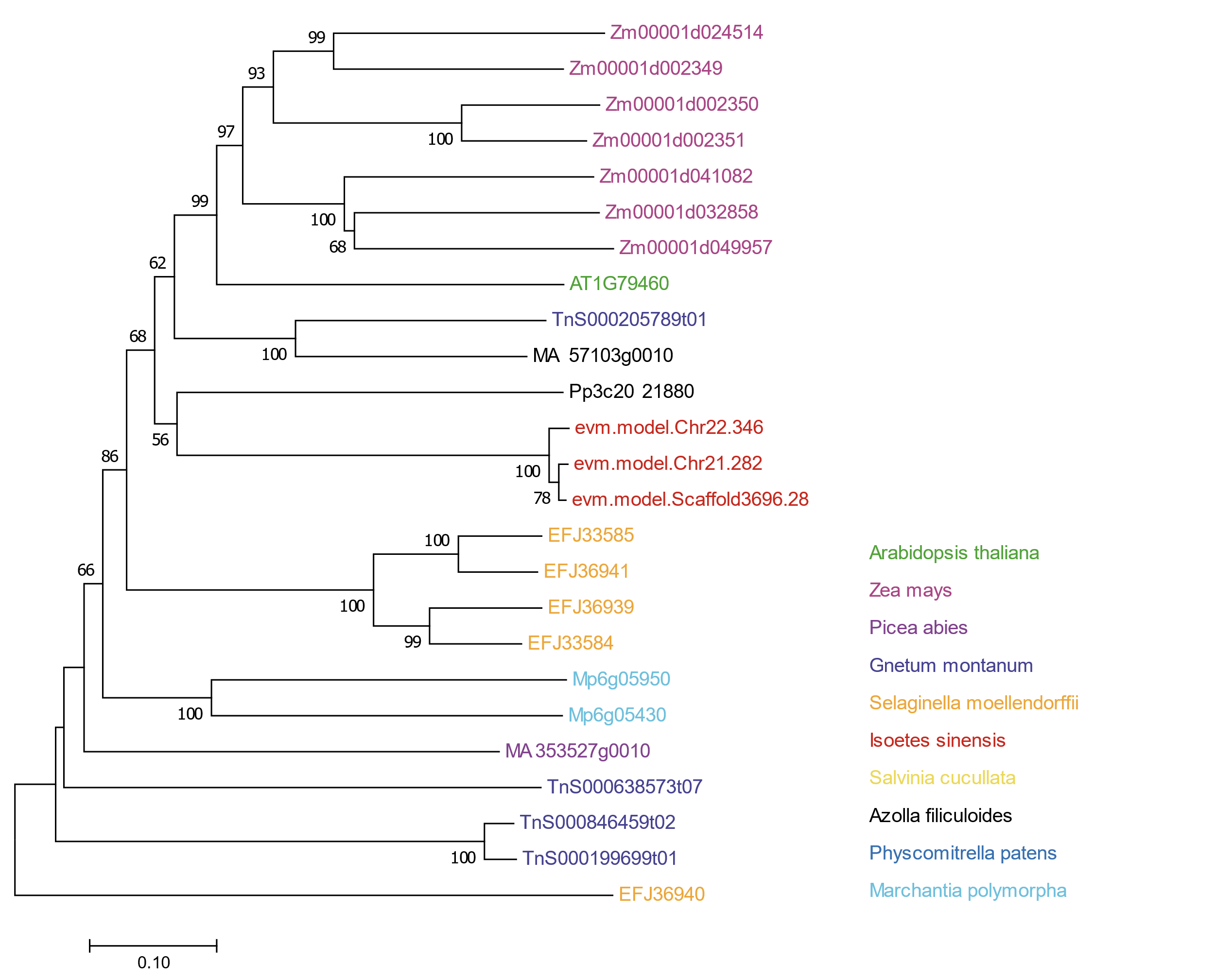


**Dataset S24. Phylogenetic relationships of KS proteins from *I. sinensis* and other evolutionarily representative species.** Numbers on the major branches indicate bootstrap values (> 50%) in 1,000 replicates.


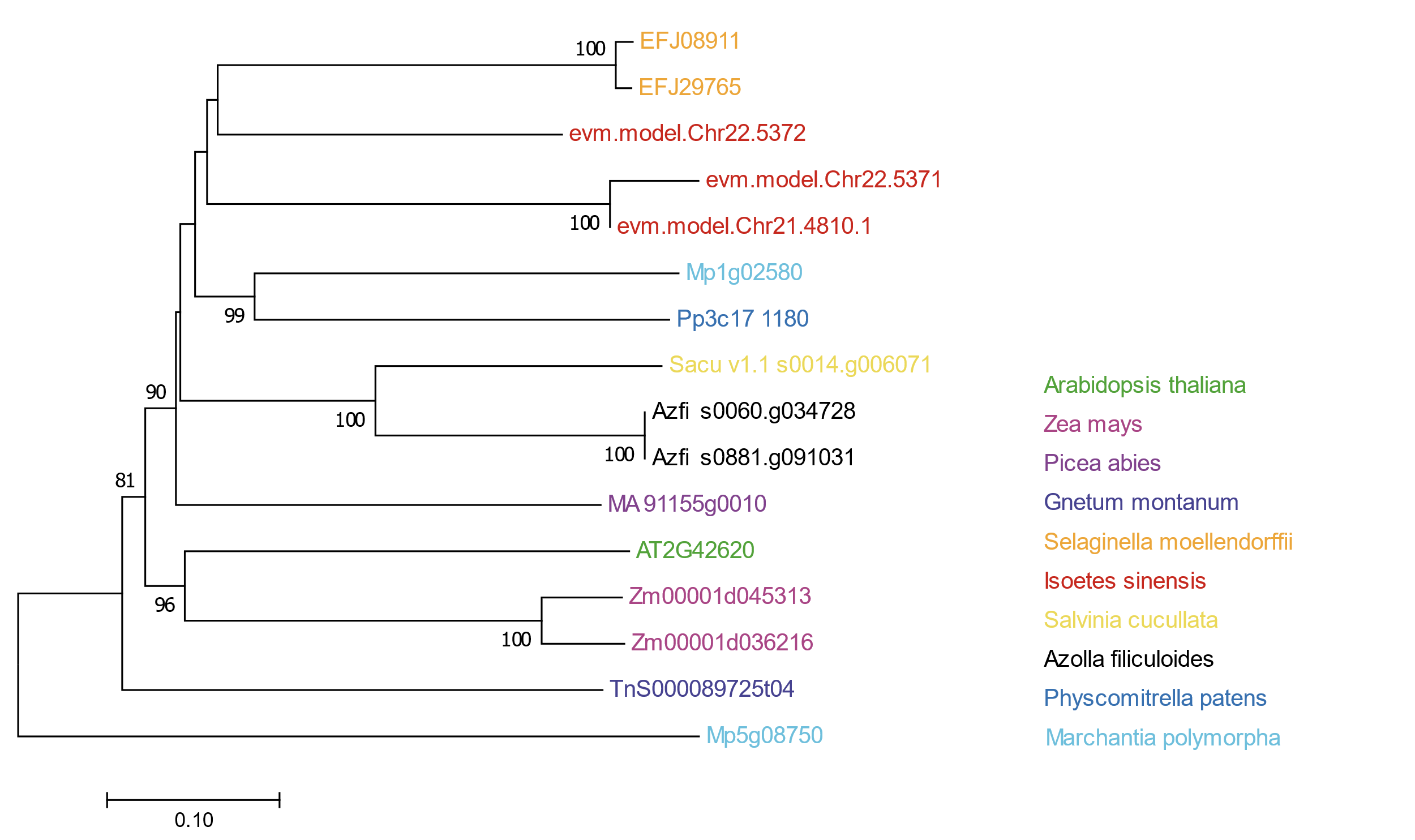


**Dataset S25. Phylogenetic relationships of D3/MAX2 proteins from *I. sinensis* and other evolutionarily representative species.** Numbers on the major branches indicate bootstrap values (> 50%) in 1,000 replicates.


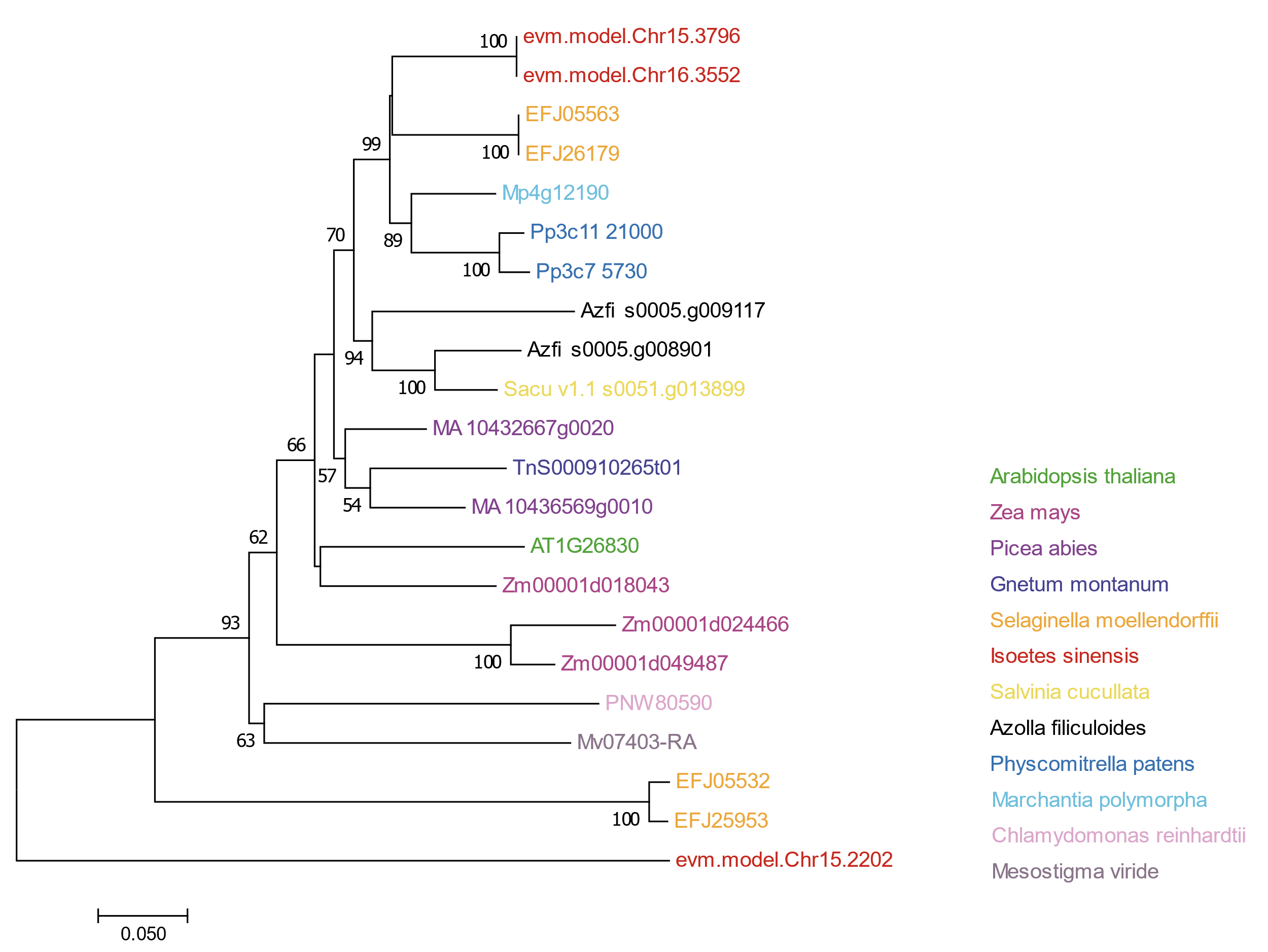


**Dataset S26. Phylogenetic relationships of CUL3 proteins from *I. sinensis* and other evolutionarily representative species.** Numbers on the major branches indicate bootstrap values (> 50%) in 1,000 replicates.


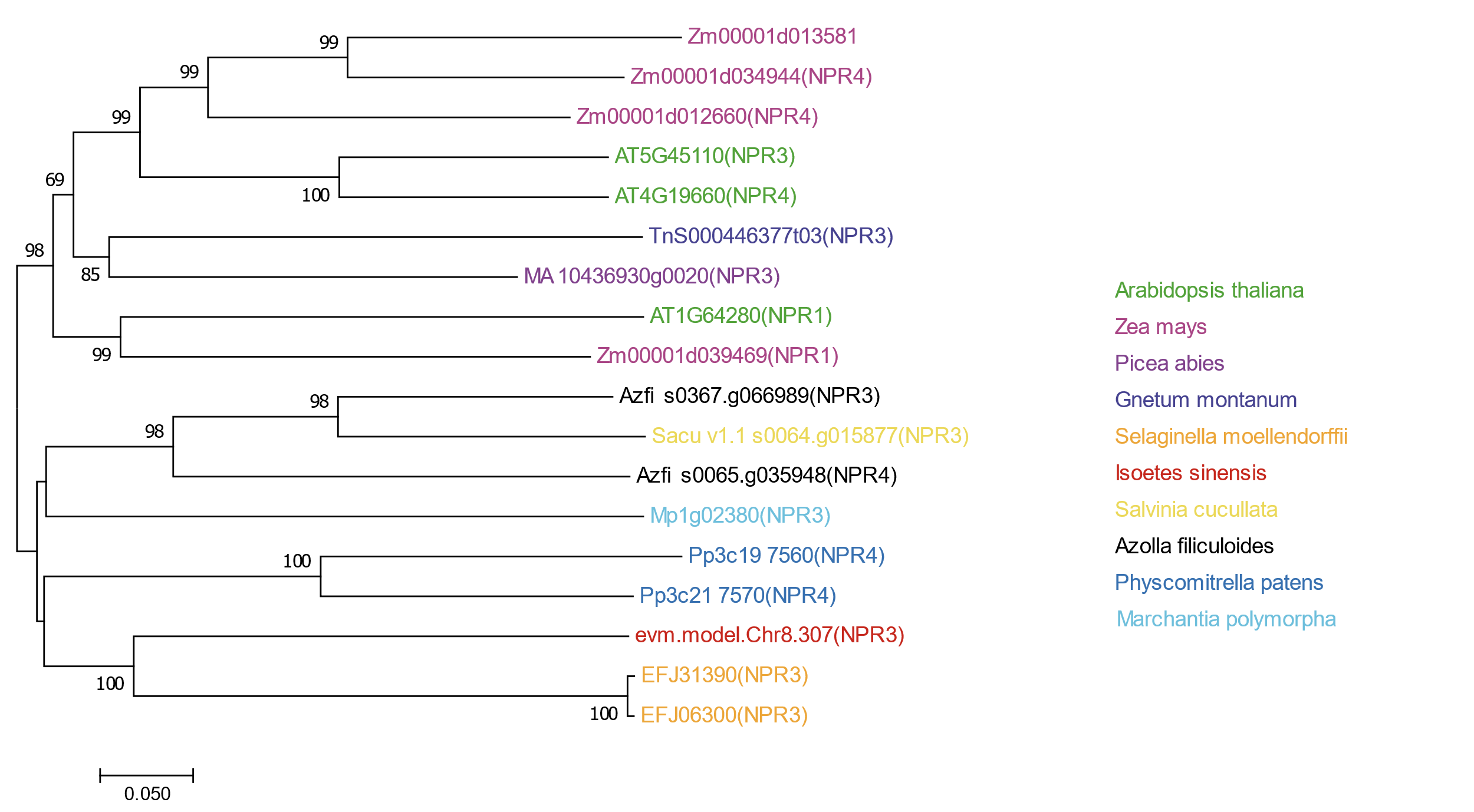


**Dataset S27. Phylogenetic relationships of NPR proteins from *I. sinensis* and other evolutionarily representative species.** Numbers on the major branches indicate bootstrap values (> 50%) in 1,000 replicates.


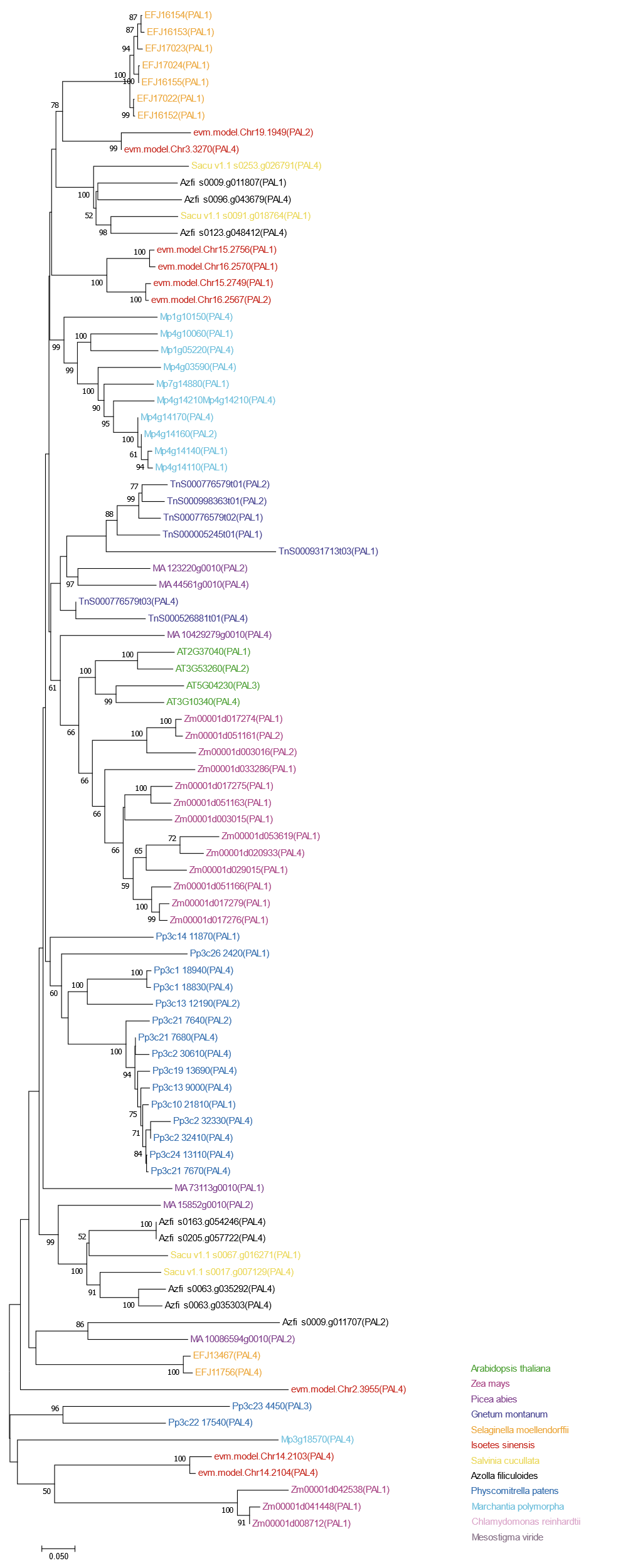


**Dataset S28. Phylogenetic relationships of PAL proteins from *I. sinensis* and other evolutionarily representative species.** Numbers on the major branches indicate bootstrap values (> 50%) in 1,000 replicates.


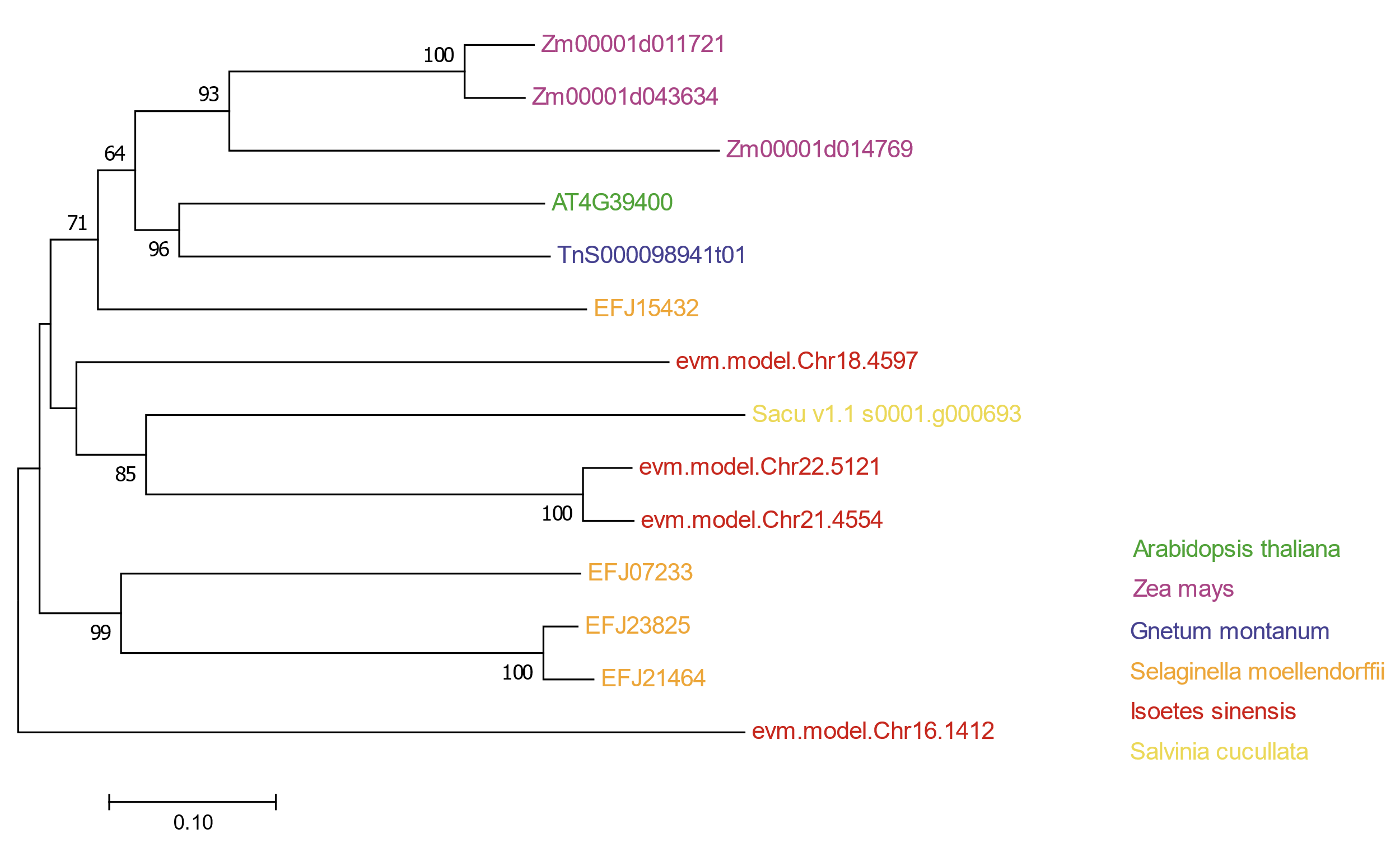


**Dataset S29.** **Phylogenetic relationships of BPI1 proteins from *I. sinensis* and other evolutionarily representative species.** Numbers on the major branches indicate bootstrap values (> 50%) in 1,000 replicates.


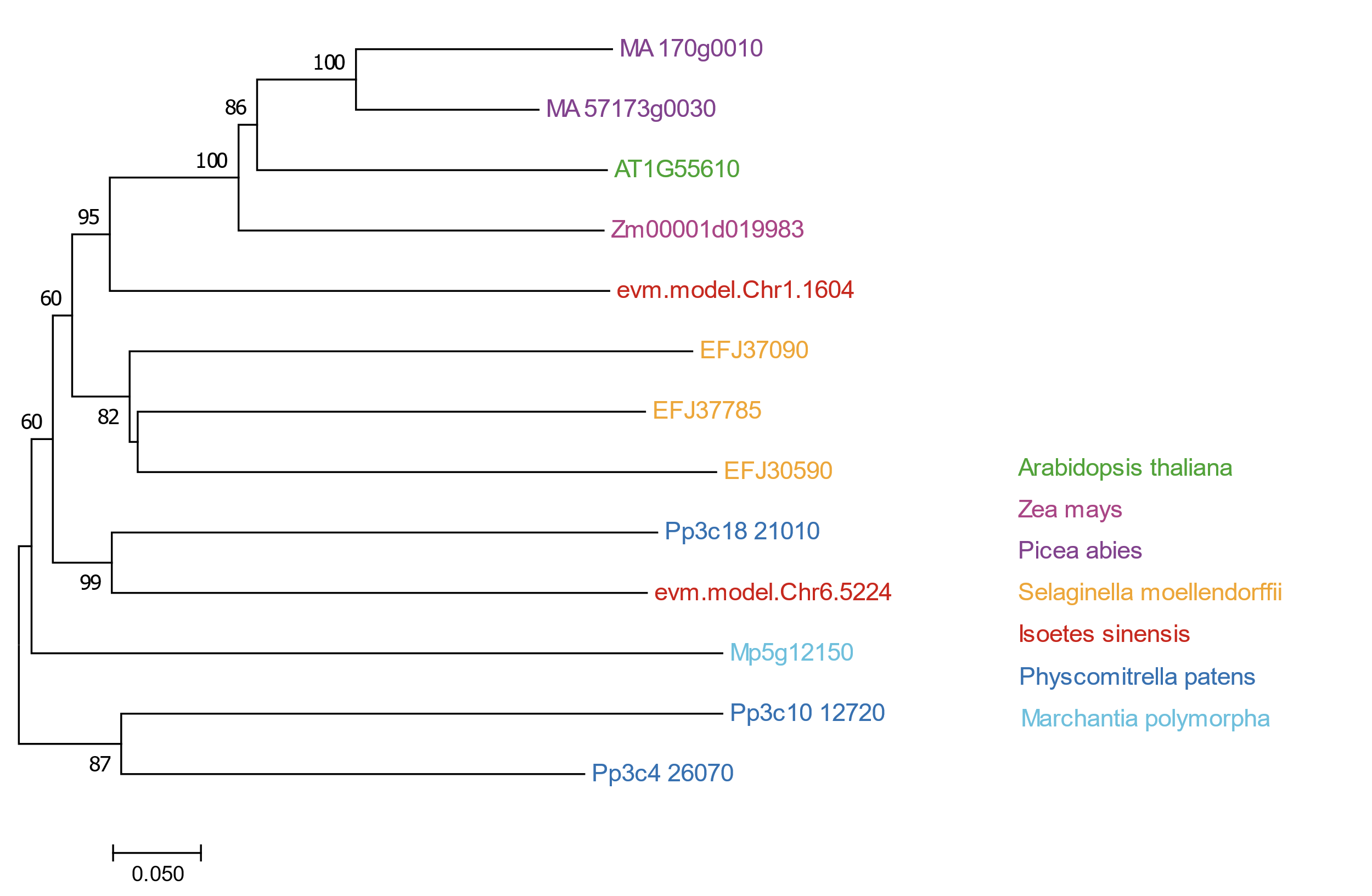


**Dataset S30. Phylogenetic relationships of BRI1-like proteins from *I. sinensis* and other evolutionarily representative species.** Numbers on the major branches indicate bootstrap values (> 50%) in 1,000 replicates.


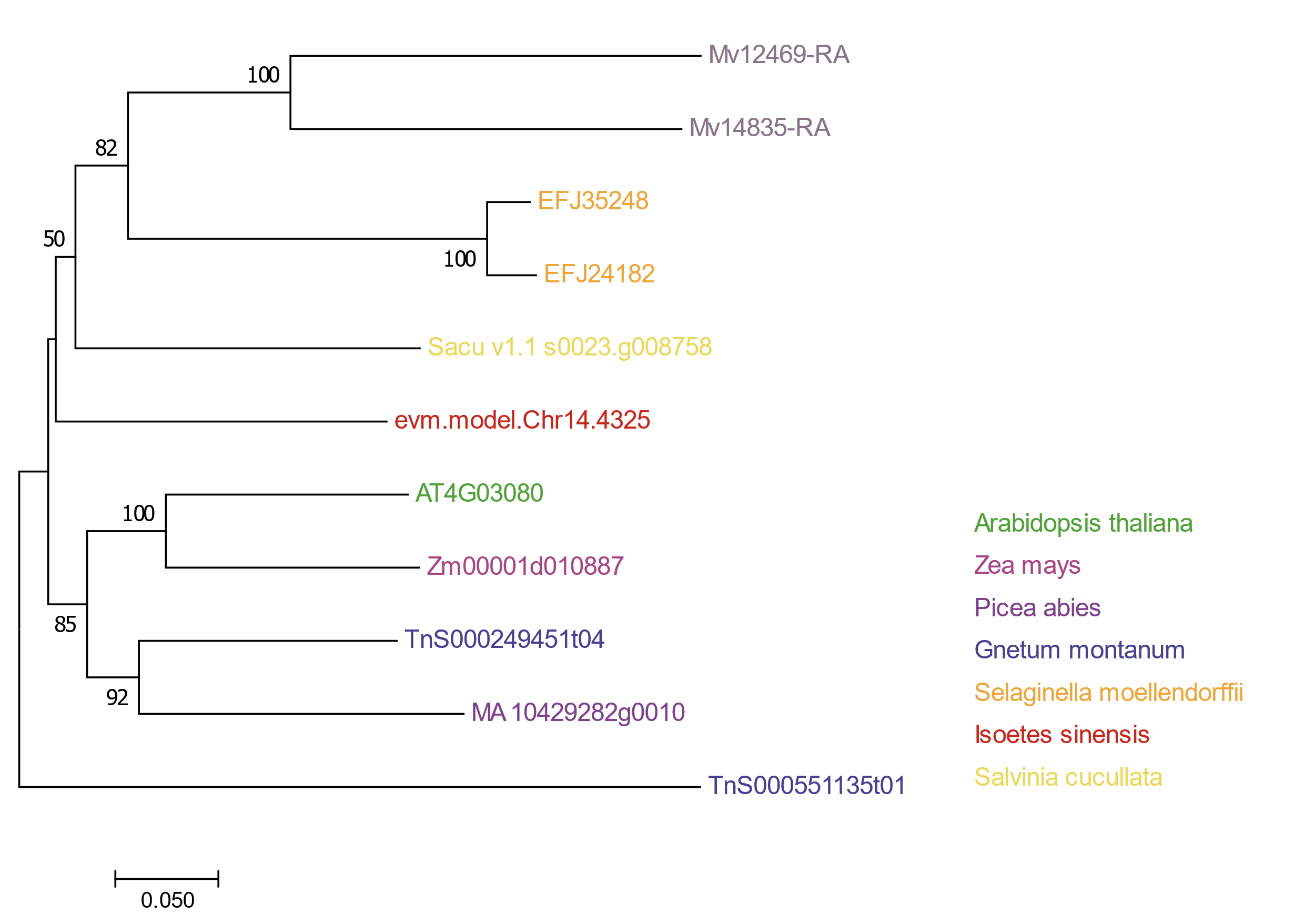


**Dataset S31.** **Phylogenetic relationships of BSU proteins from *I. sinensis* and other evolutionarily representative species.** Numbers on the major branches indicate bootstrap values (> 50%) in 1,000 replicates.


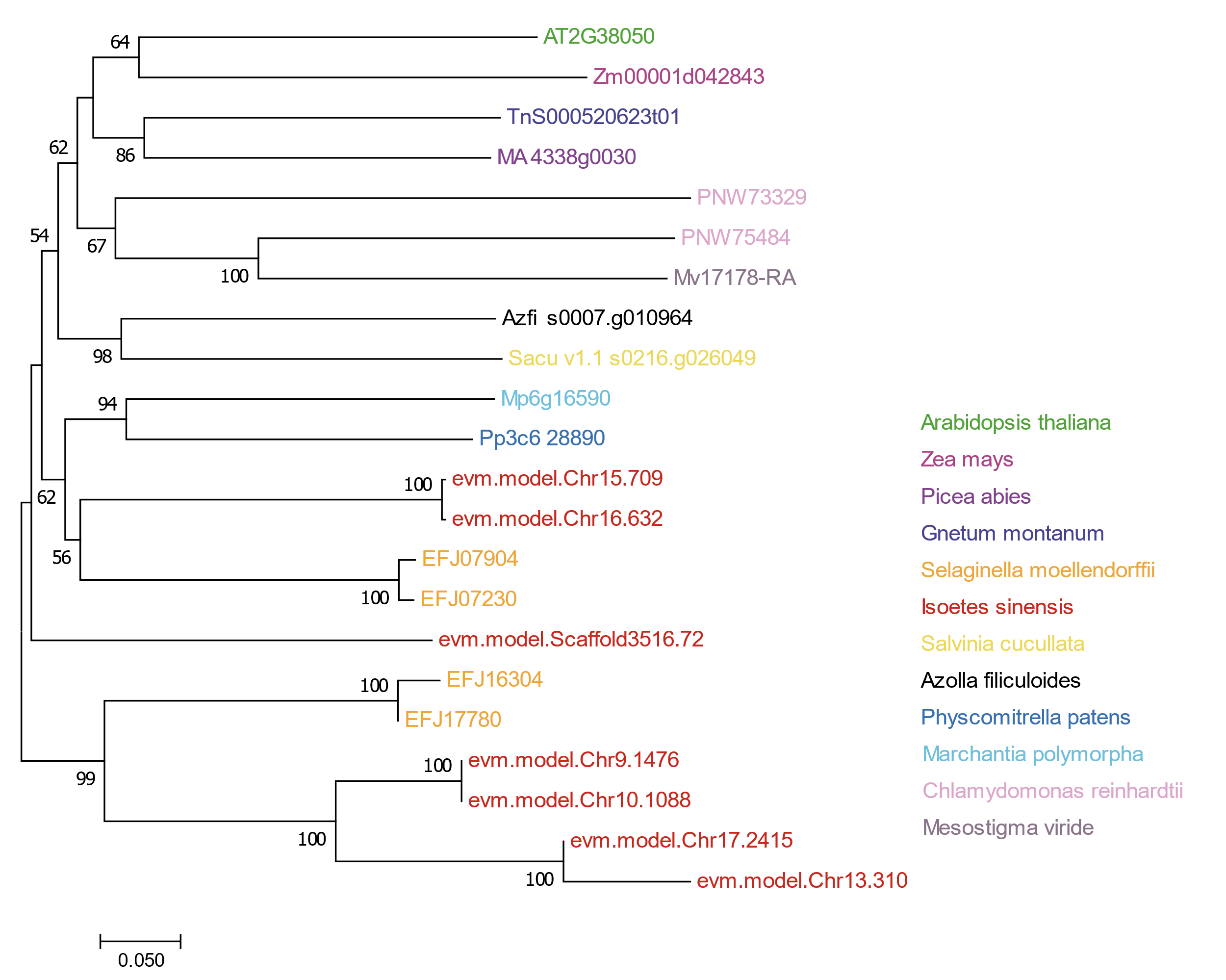


**Dataset S32.** **Phylogenetic relationships of DET2 proteins from *I. sinensis* and other evolutionarily representative species.** Numbers on the major branches indicate bootstrap values (> 50%) in 1,000 replicates.


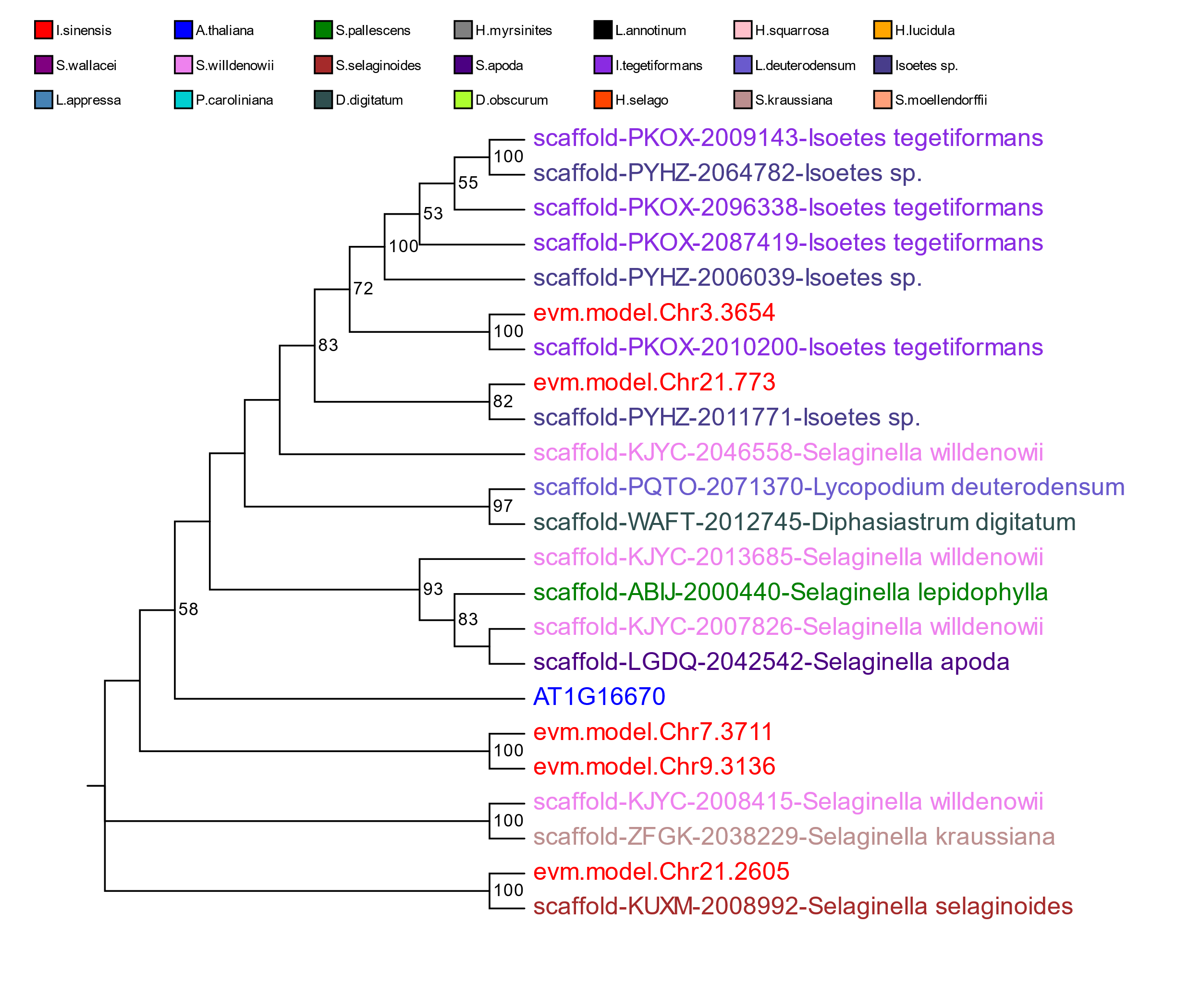


**Dataset S33. Phylogenetic relationships of CRPK1 proteins from *I. sinensis* and other lycophyte species.** Numbers on the major branches indicate bootstrap values (> 50%) in 1,000 replicates.


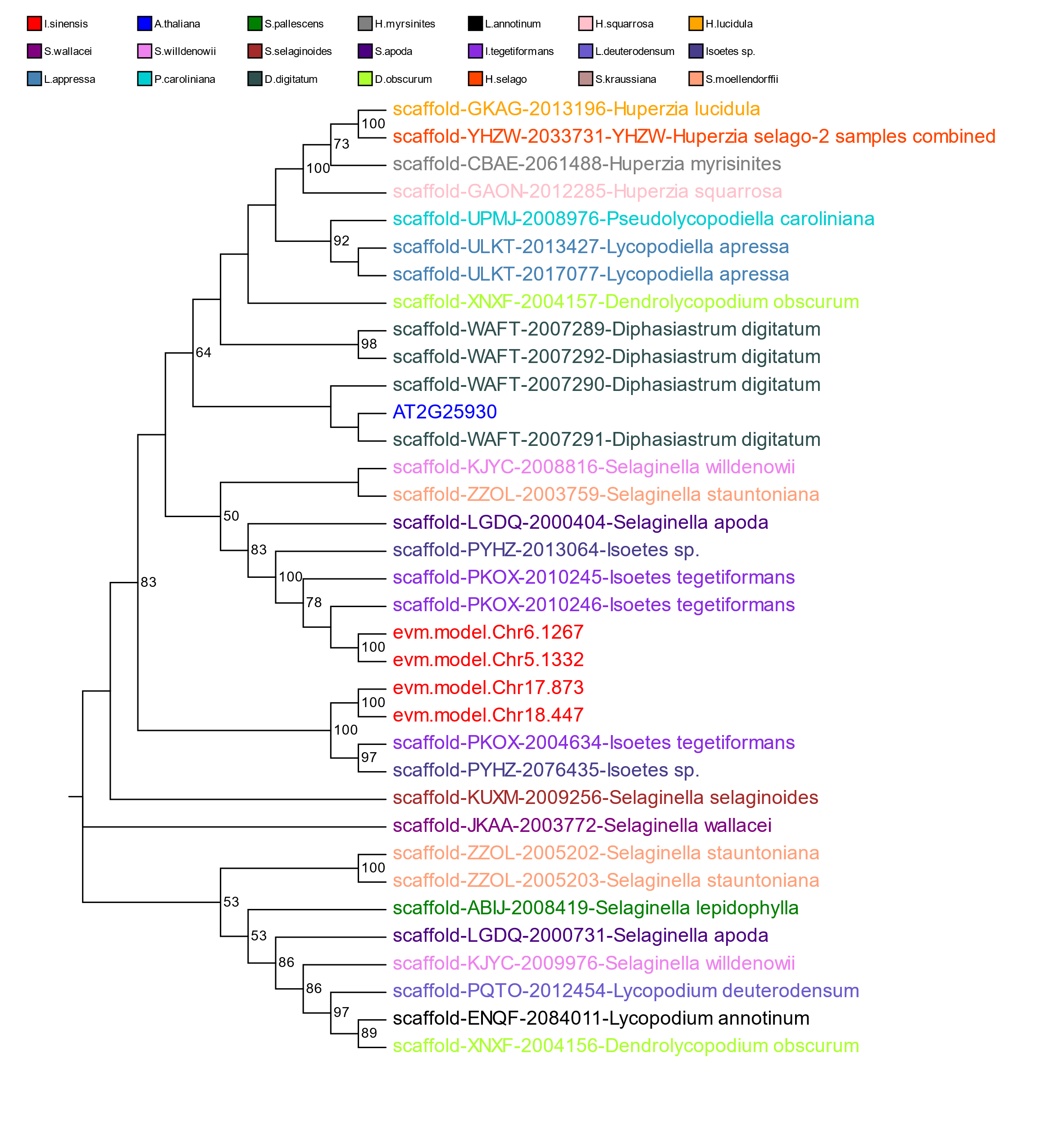


**Dataset S34. Phylogenetic relationships of ELF3 proteins from *I. sinensis* and other lycophyte species.** Numbers on the major branches indicate bootstrap values (> 50%) in 1,000 replicates.


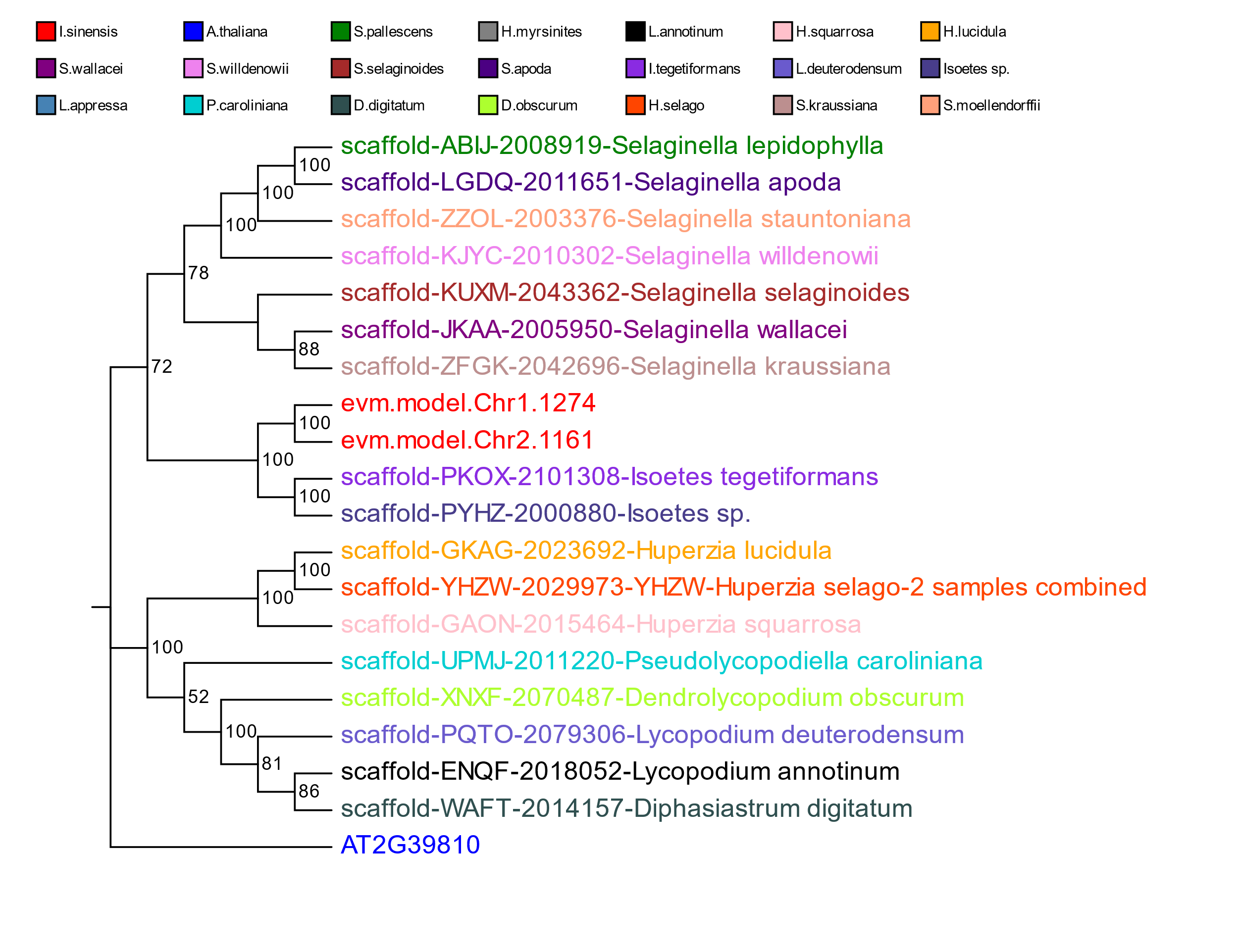


**Dataset S35. Phylogenetic relationships of HOS1 proteins from *I. sinensis* and other lycophyte species.** Numbers on the major branches indicate bootstrap values (> 50%) in 1,000 replicates.


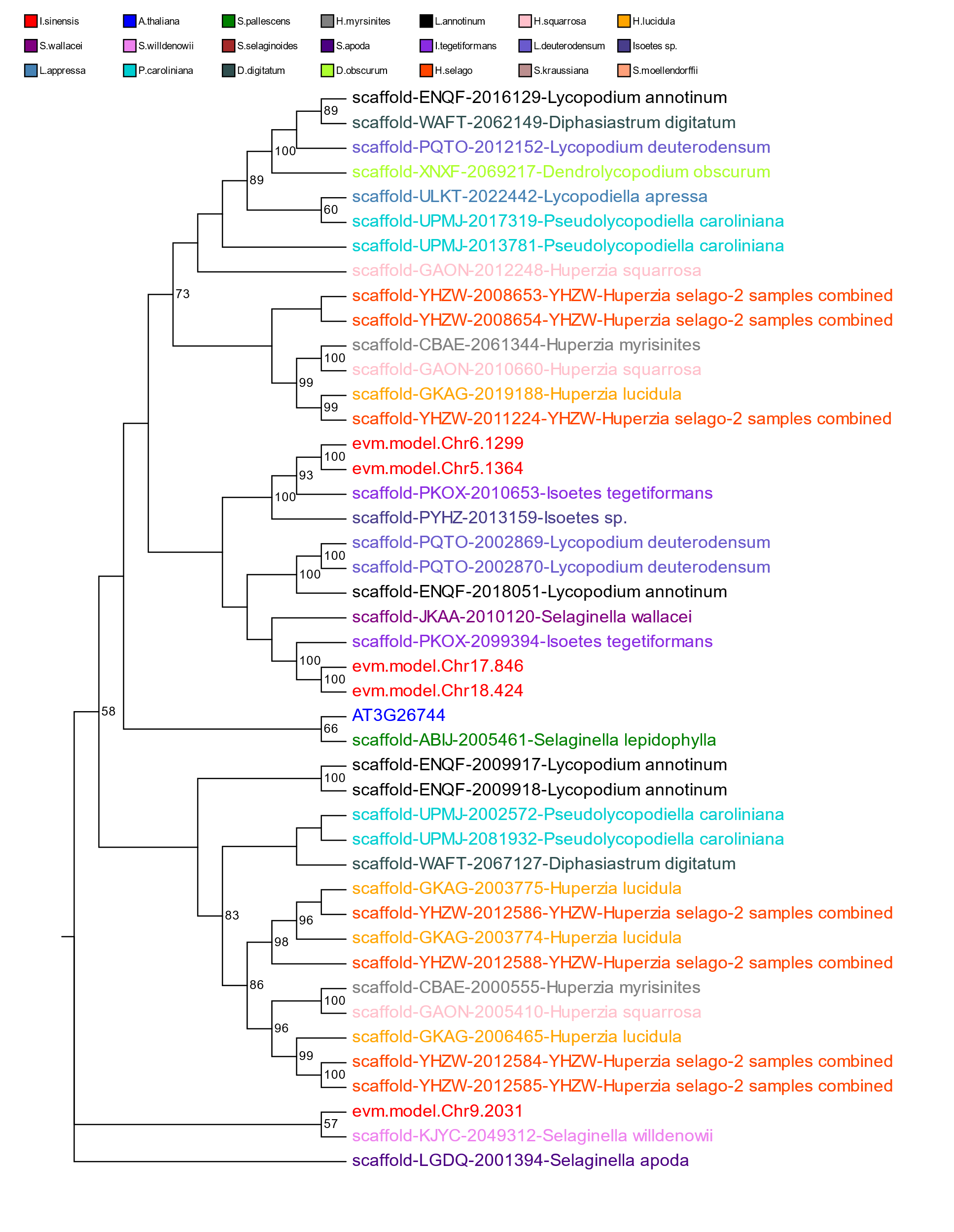


**Dataset S36. Phylogenetic relationships of ICE1 proteins from *I. sinensis* and other lycophyte species.** Numbers on the major branches indicate bootstrap values (> 50%) in 1,000 replicates.


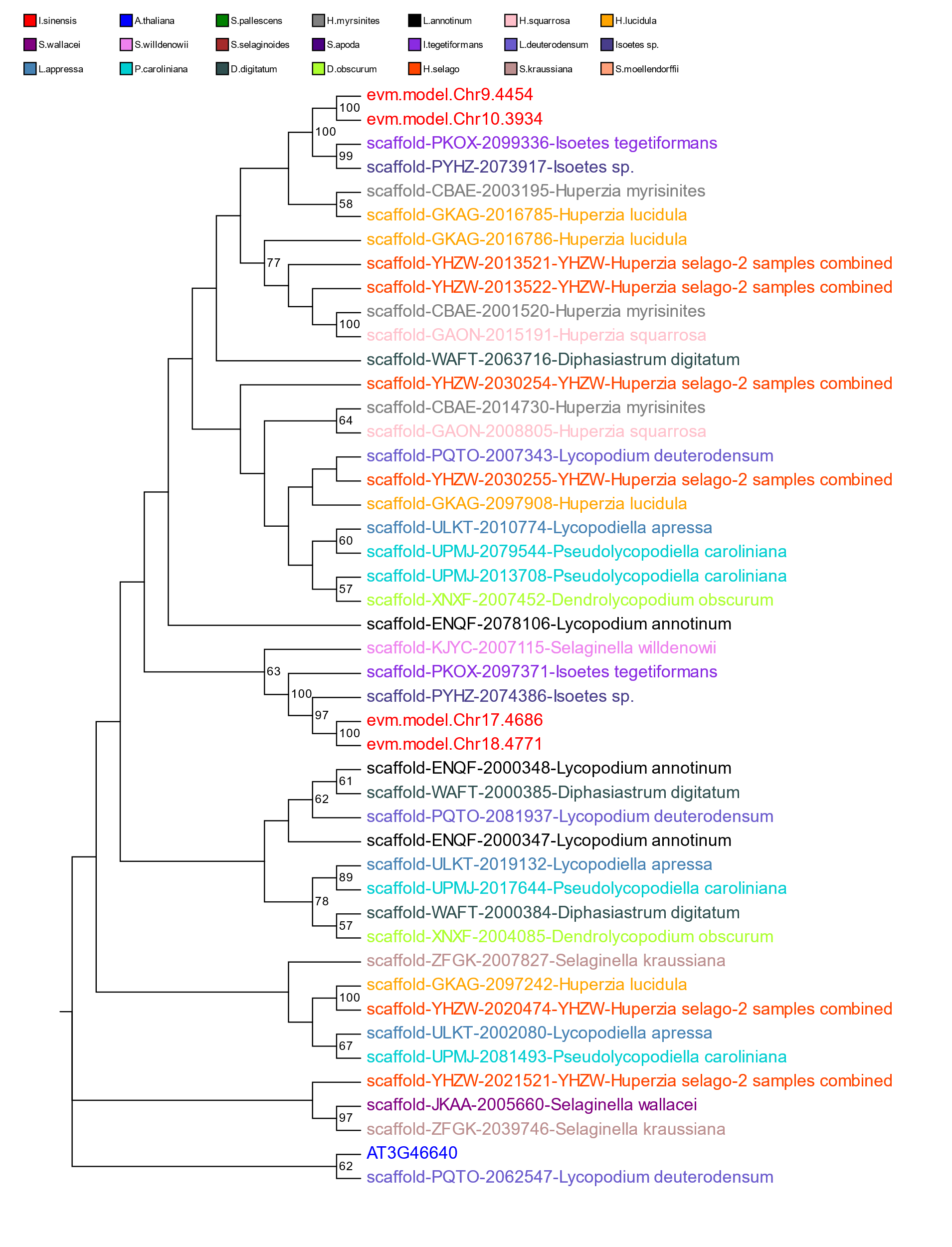


**Dataset S37. Phylogenetic relationships of LUX proteins from *I. sinensis* and other lycophyte species.** Numbers on the major branches indicate bootstrap values (> 50%) in 1,000 replicates.


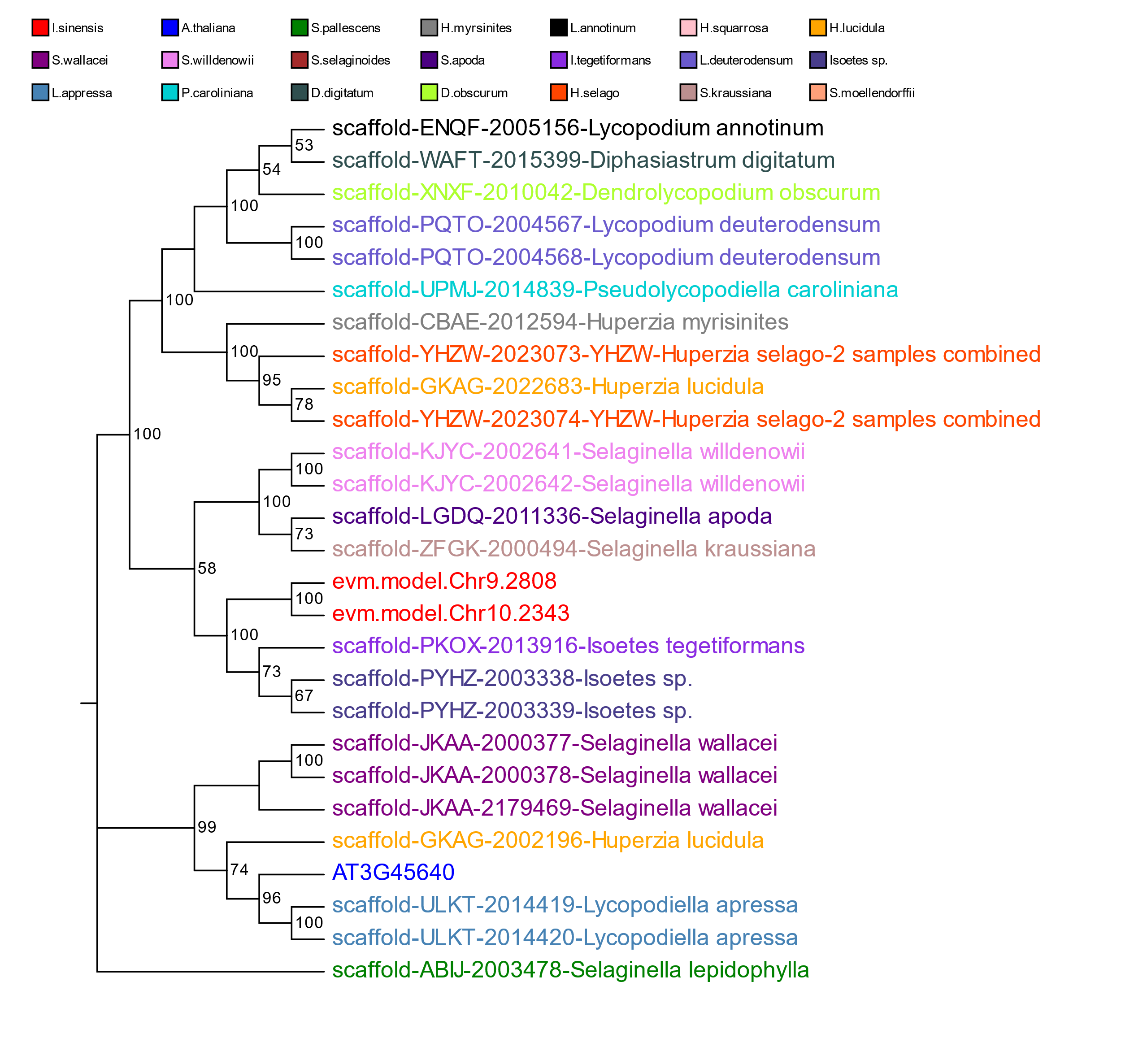


**Dataset S38. Phylogenetic relationships of MPK3 proteins from *I. sinensis* and other lycophyte species.** Numbers on the major branches indicate bootstrap values (> 50%) in 1,000 replicates.


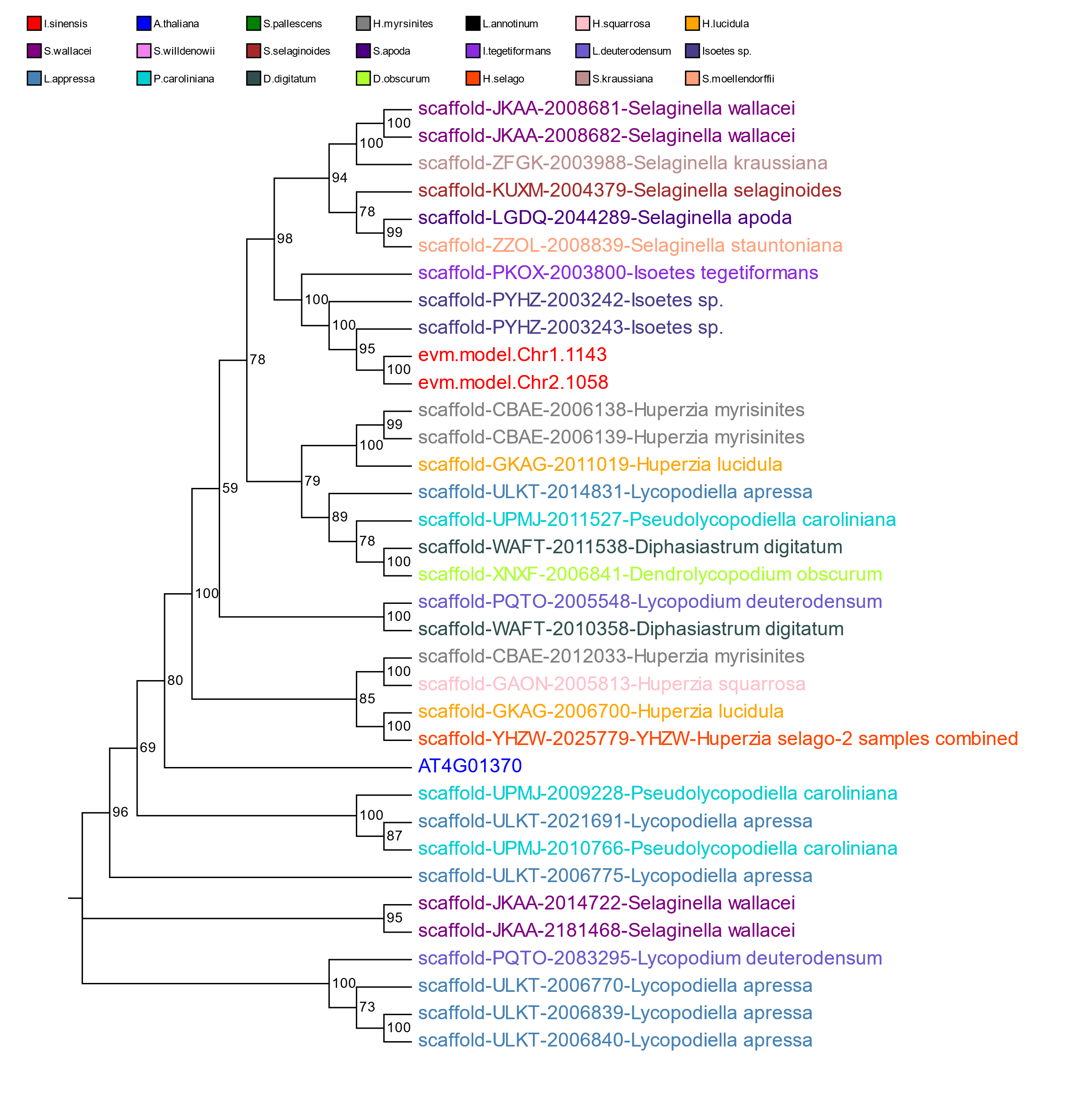


**Dataset S39.** **Phylogenetic relationships of MPK4 proteins from *I. sinensis* and other lycophyte species.** Numbers on the major branches indicate bootstrap values (> 50%) in 1,000 replicates.


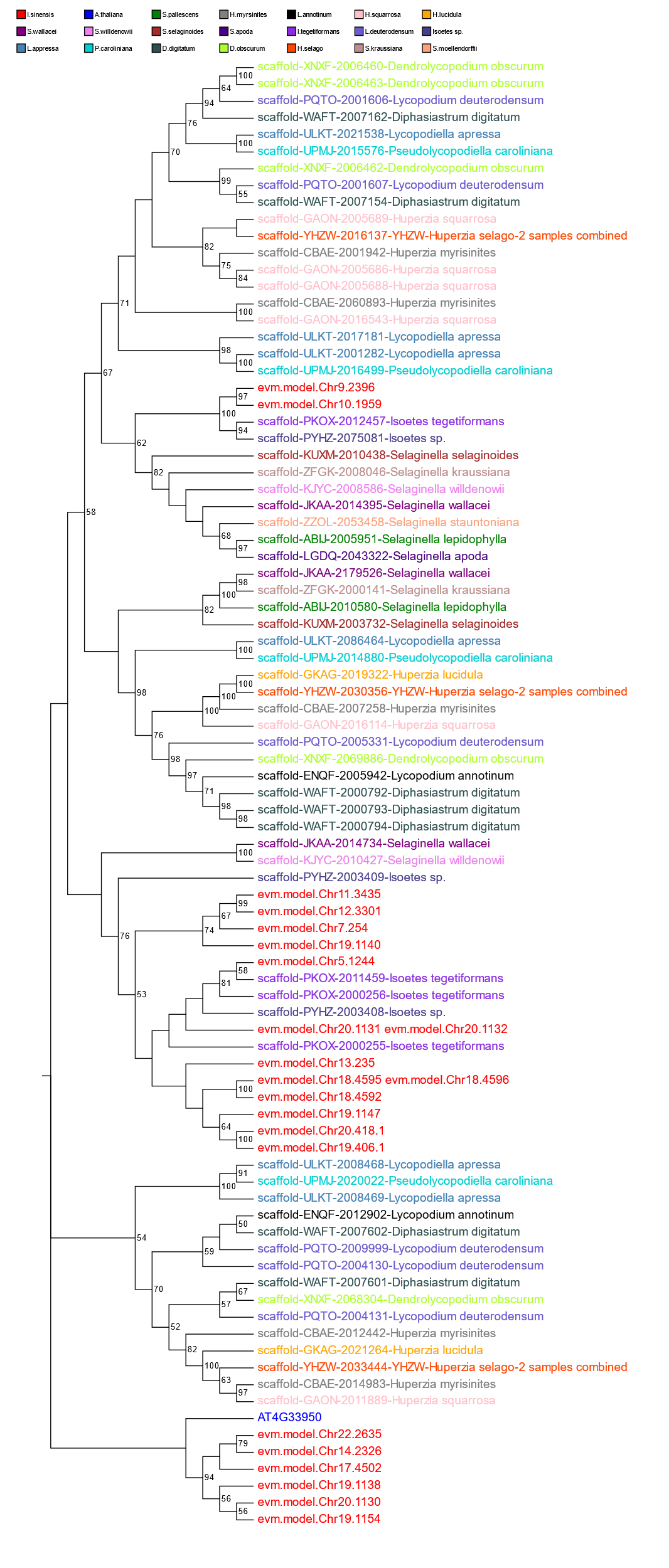


**Dataset S40.** **Phylogenetic relationships of OST1 proteins from *I. sinensis* and other lycophyte species.** Numbers on the major branches indicate bootstrap values (> 50%) in 1,000 replicates.


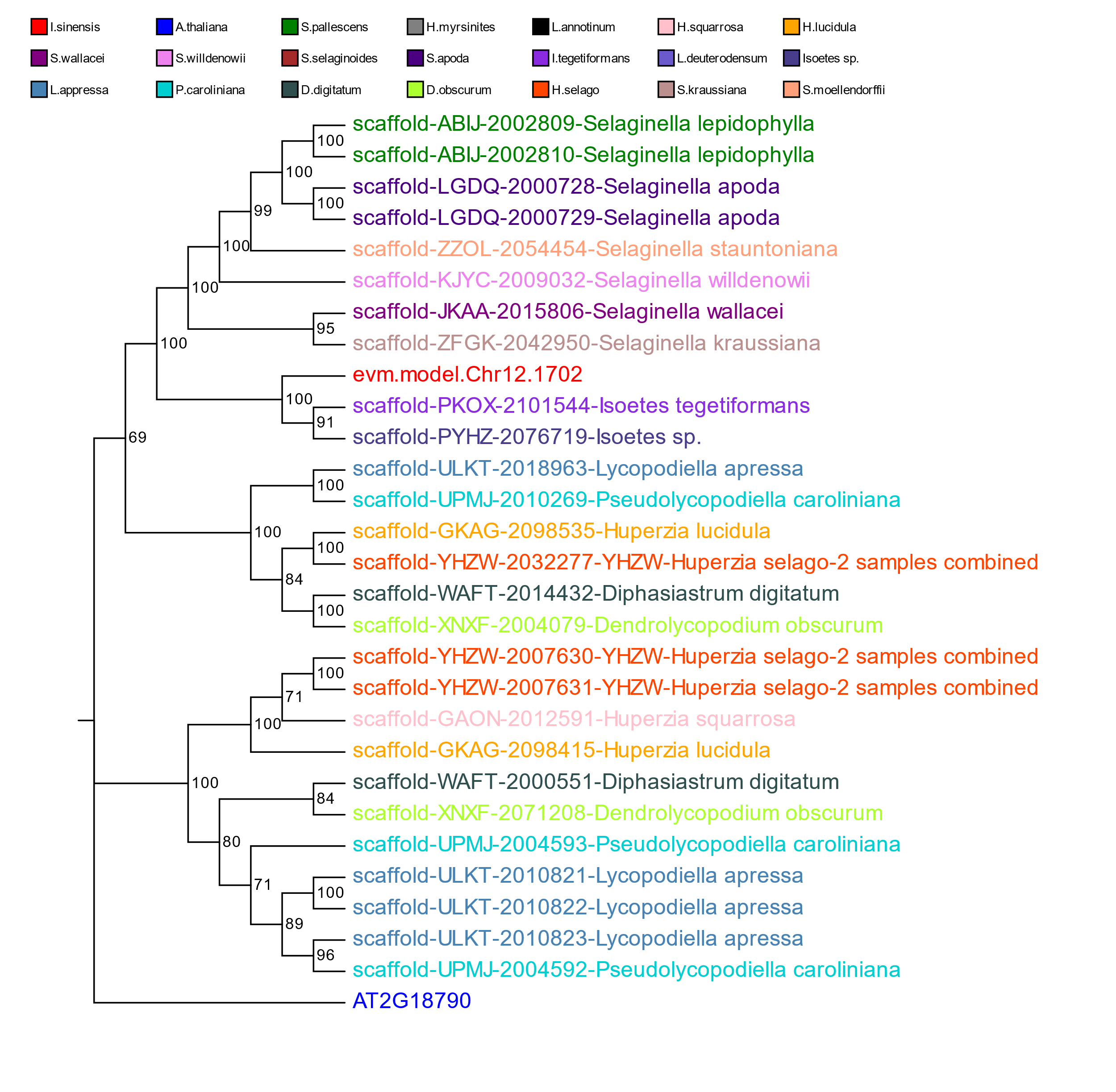


**Dataset S41. Phylogenetic relationships of phyB proteins from *I. sinensis* and other lycophyte species.** Numbers on the major branches indicate bootstrap values (> 50%) in 1,000 replicates.


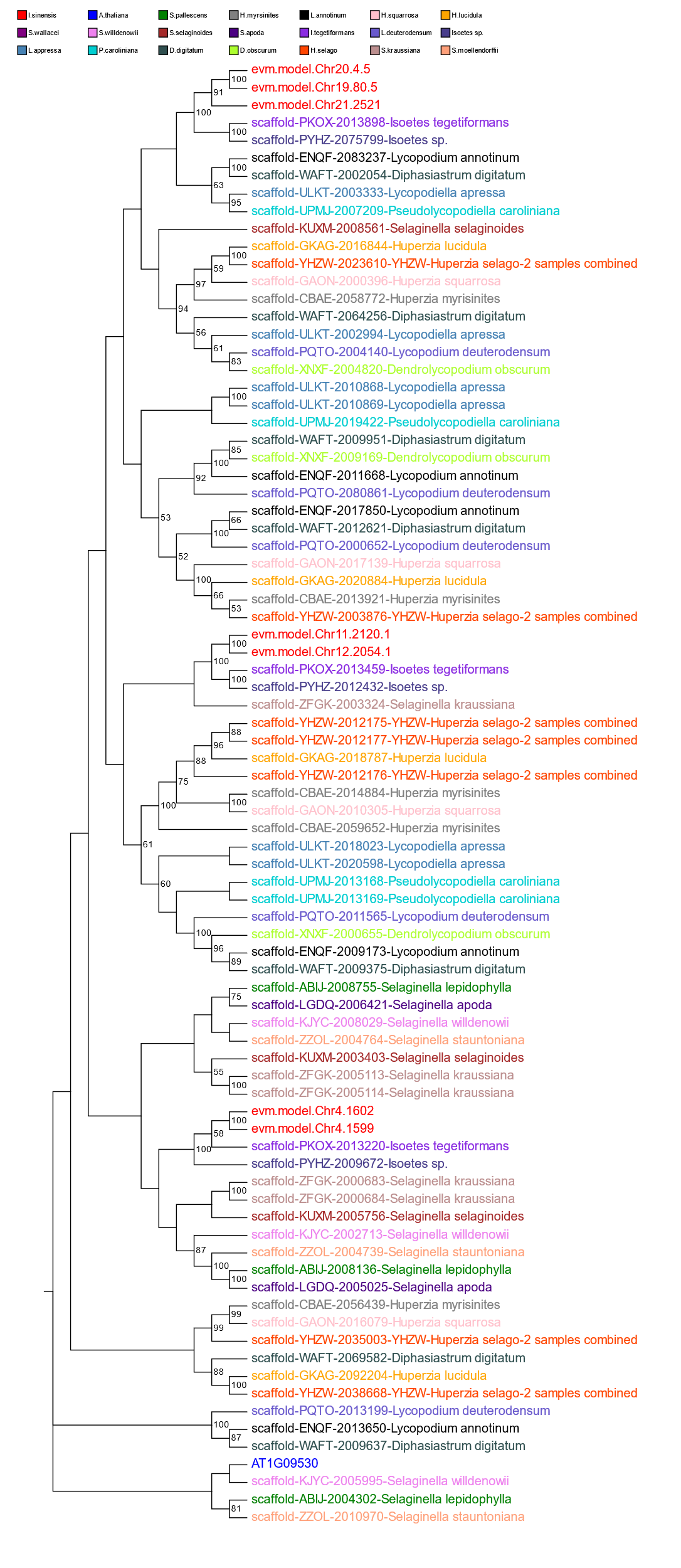


**Dataset S42. Phylogenetic relationships of PIF3 proteins from *I. sinensis* and other lycophyte species.** Numbers on the major branches indicate bootstrap values (> 50%) in 1,000 replicates.


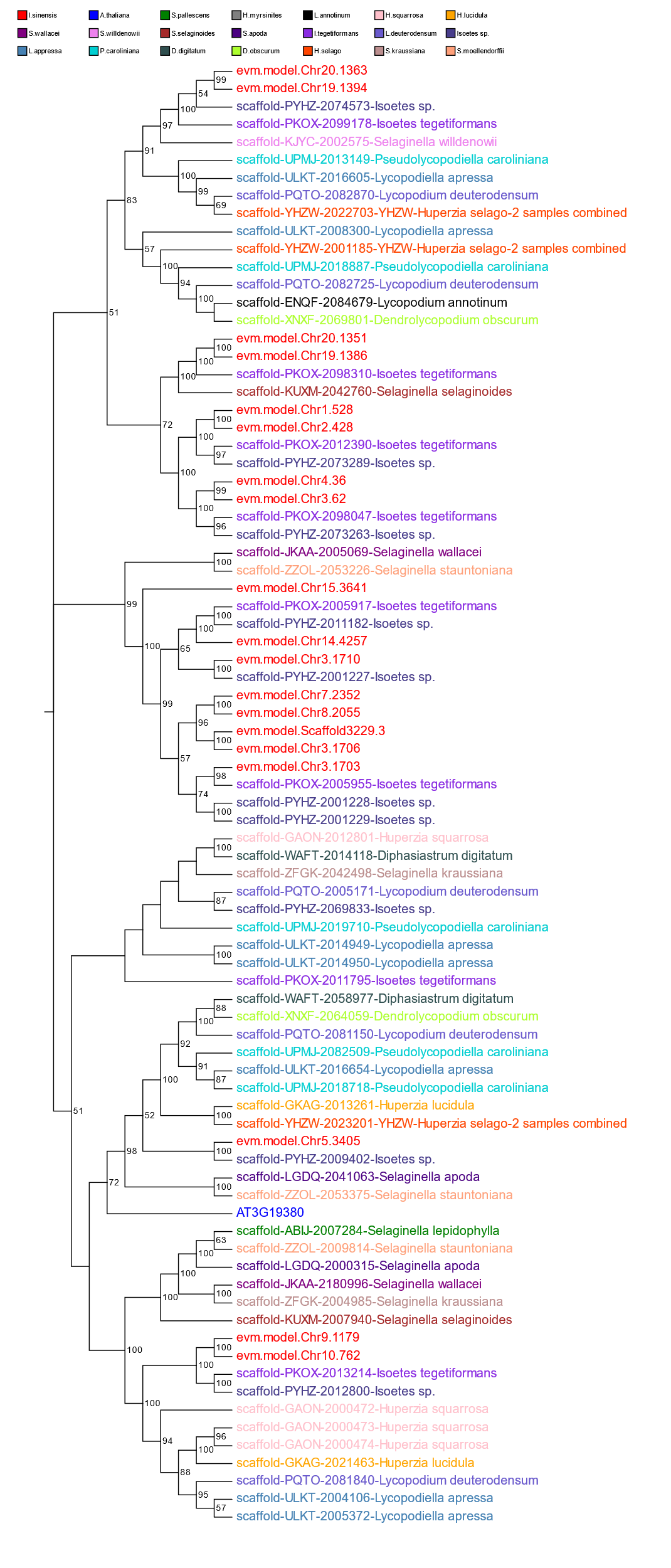


**Dataset S43. Phylogenetic relationships of PUB25 proteins from *I. sinensis* and other lycophyte species.** Numbers on the major branches indicate bootstrap values (> 50%) in 1,000 replicates.


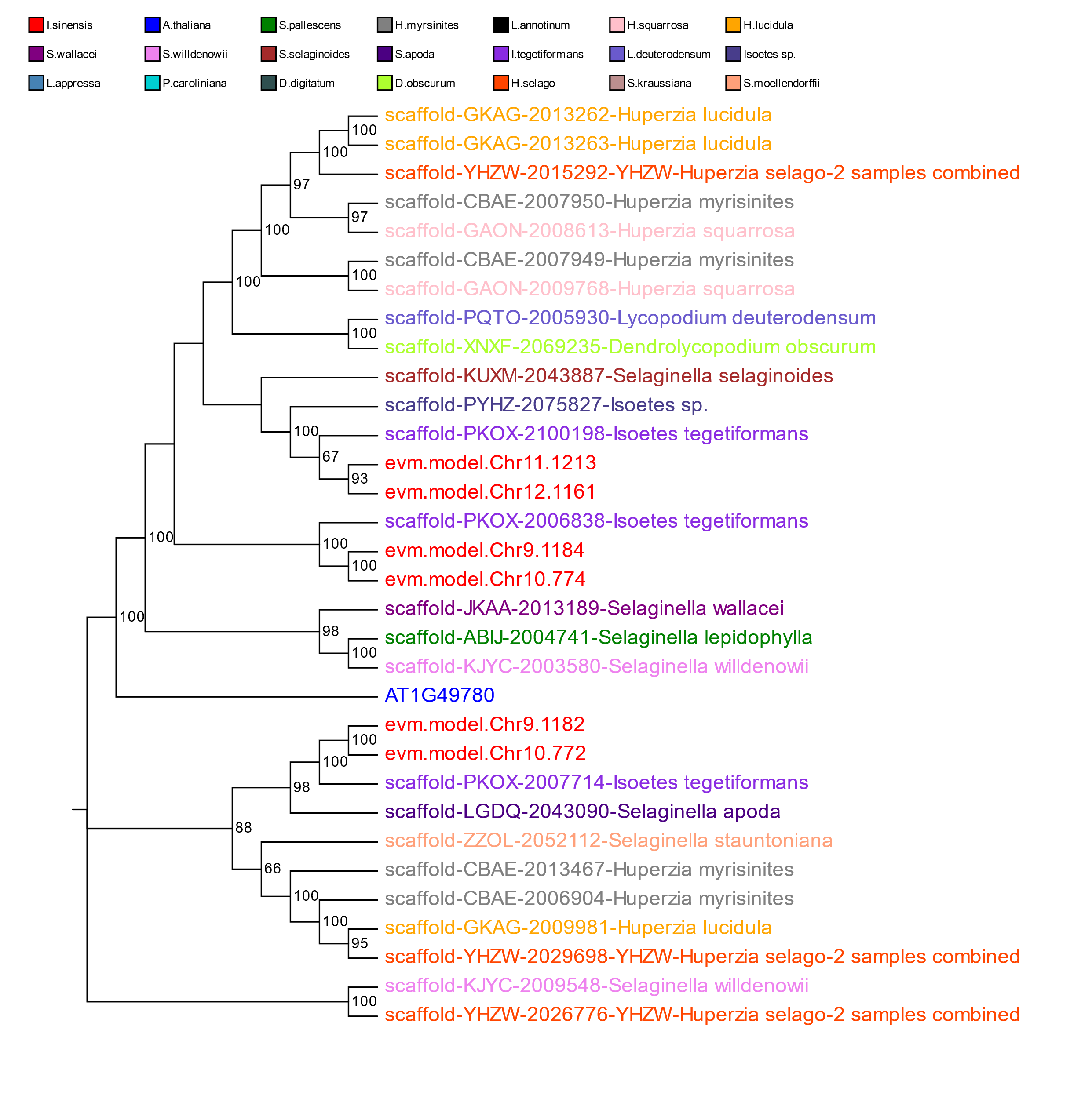


**Dataset S44. Phylogenetic relationships of PUB26 proteins from *I. sinensis* and other lycophyte species.** Numbers on the major branches indicate bootstrap values (> 50%) in 1,000 replicates.


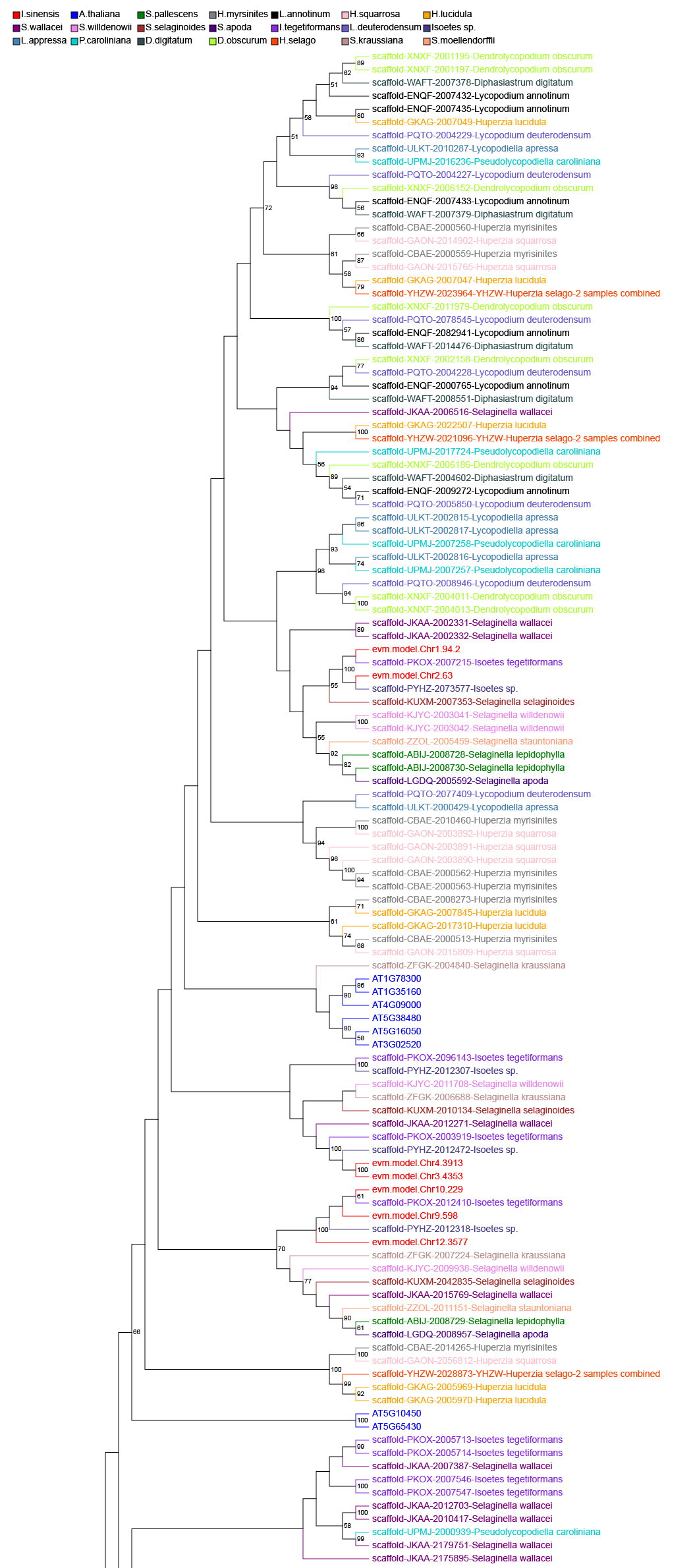


**
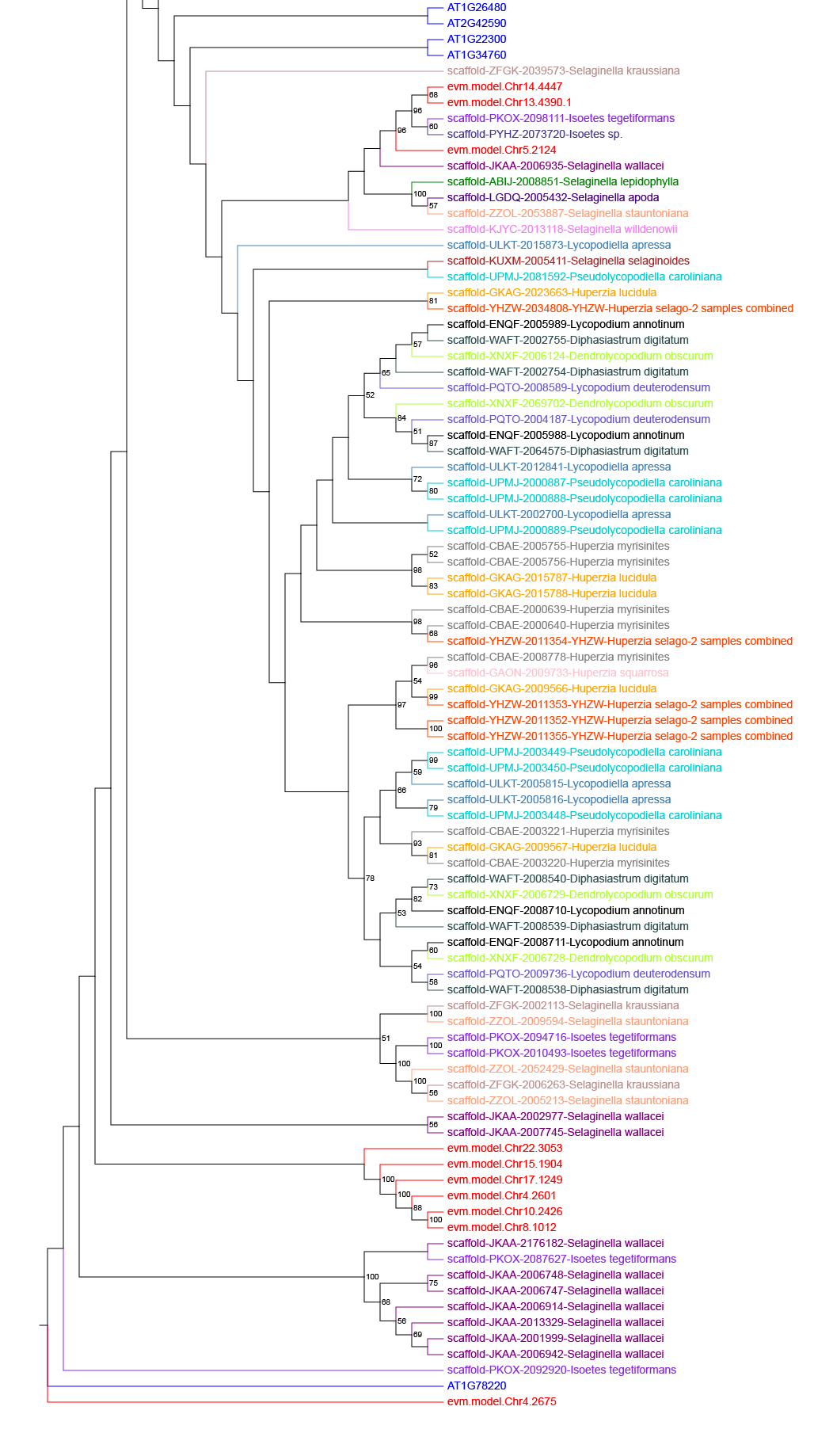
**

**Dataset S45. Phylogenetic relationships of GRF proteins from *I. sinensis* and other lycophyte species.** Numbers on the major branches indicate bootstrap values (> 50%) in 1,000 replicates.


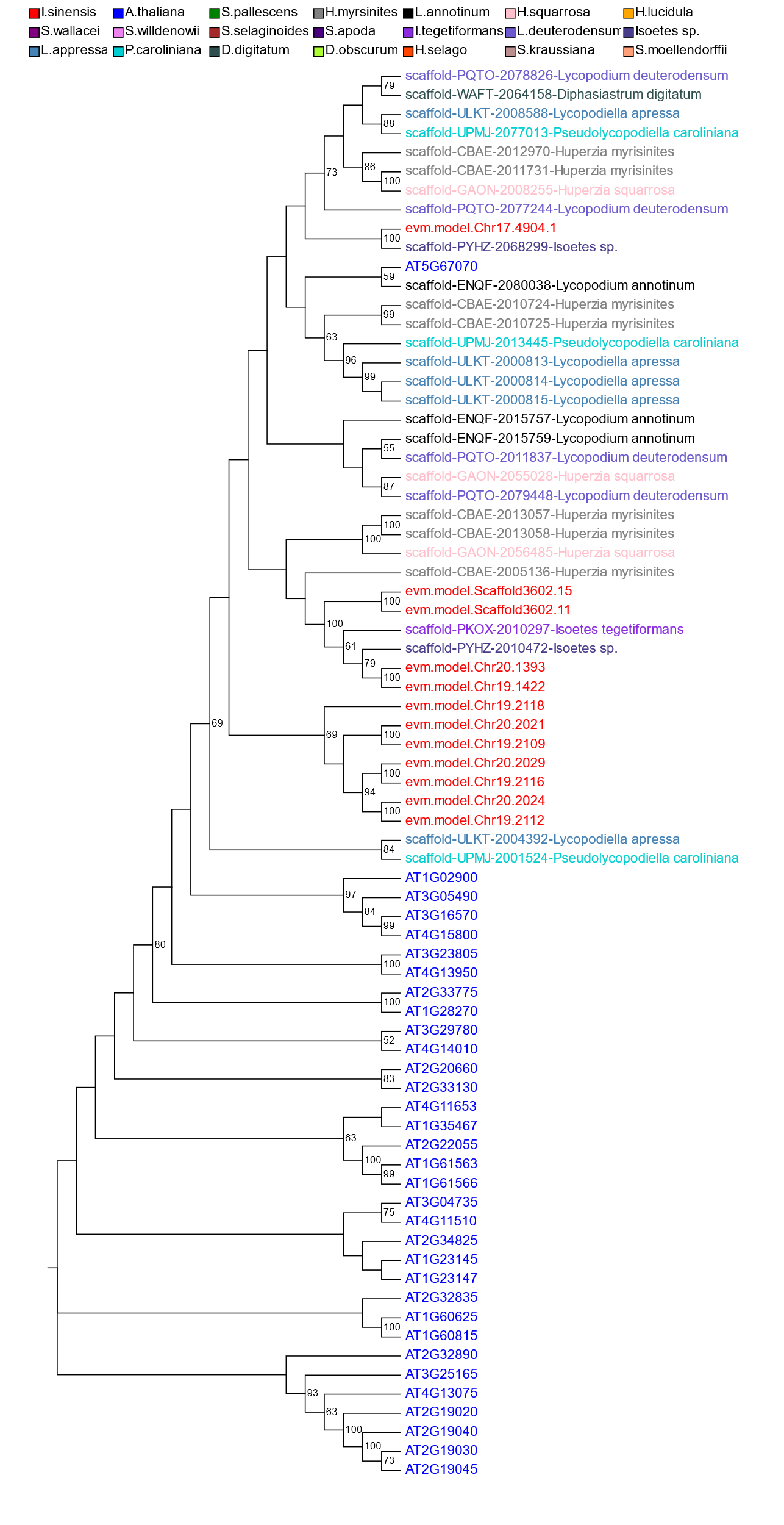


**Dataset S46. Phylogenetic relationships of RALF proteins from *I. sinensis* and other lycophyte species.** Numbers on the major branches indicate bootstrap values (> 50%) in 1,000 replicates.


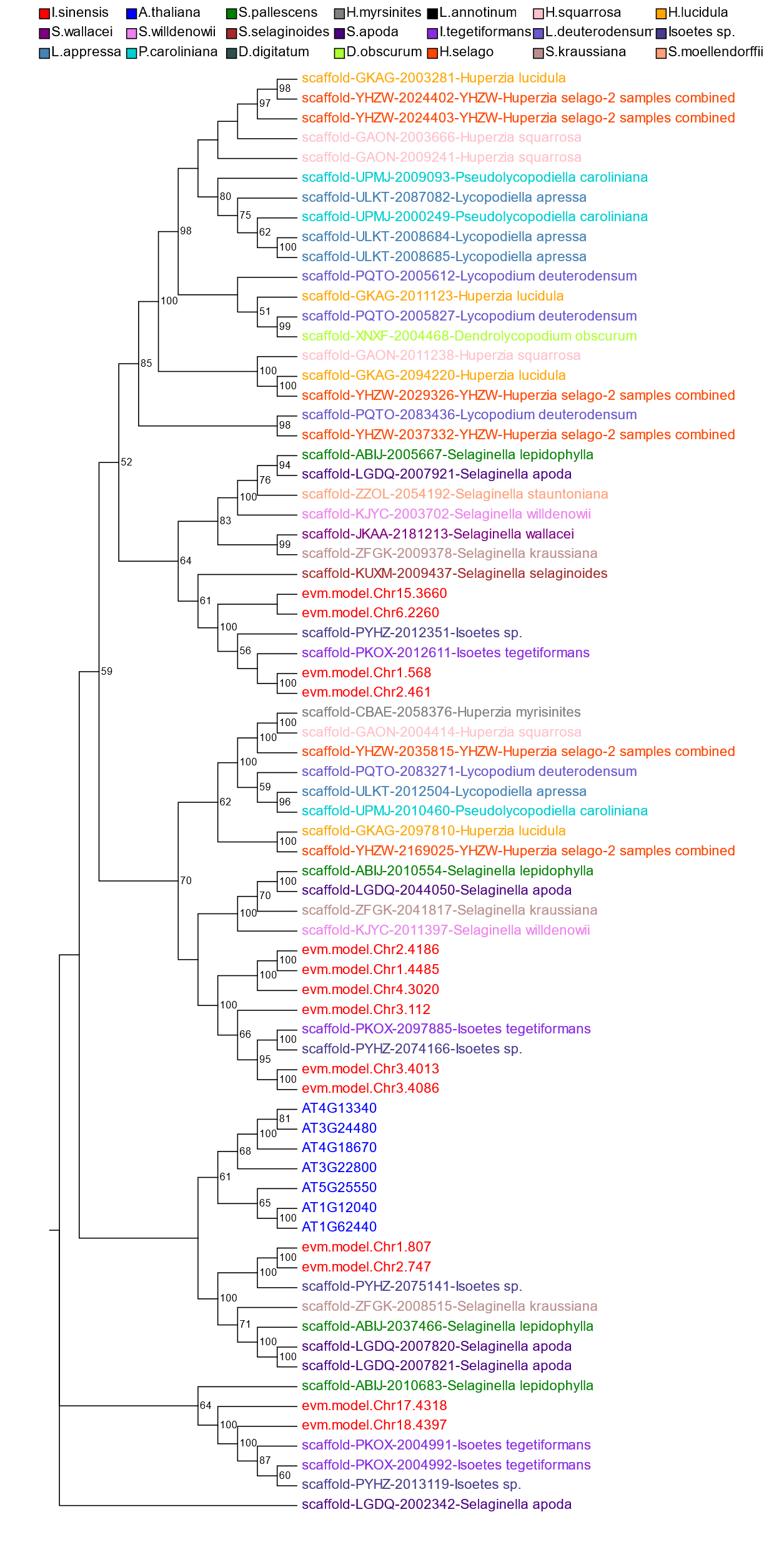


**Dataset S47. Phylogenetic relationships of LRX proteins from *I. sinensis* and other lycophyte species.** Numbers on the major branches indicate bootstrap values (> 50%) in 1,000 replicates.


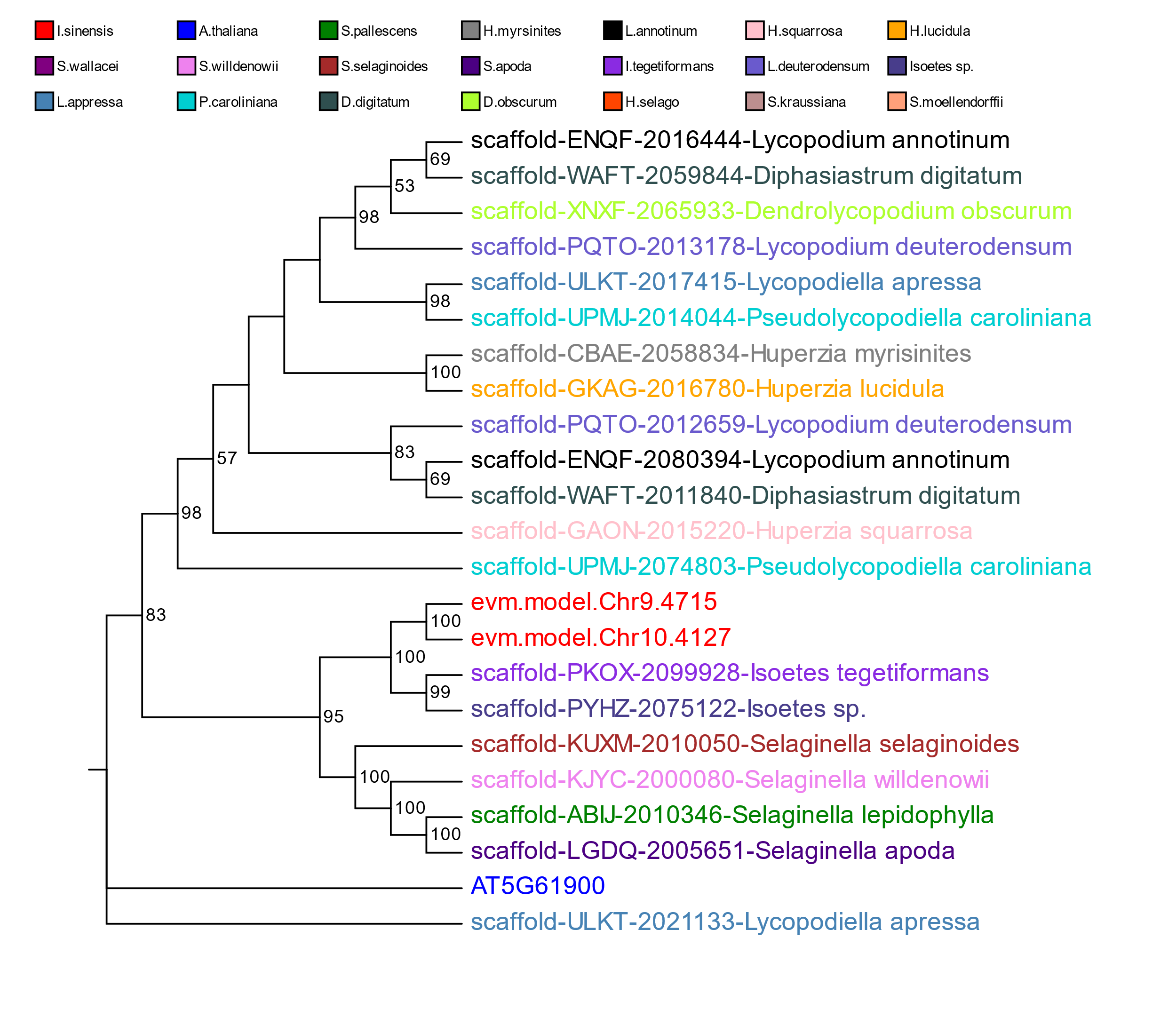


**Dataset S48. Phylogenetic relationships of BON1 proteins from *I. sinensis* and other lycophyte species.** Numbers on the major branches indicate bootstrap values (> 50%) in 1,000 replicates.


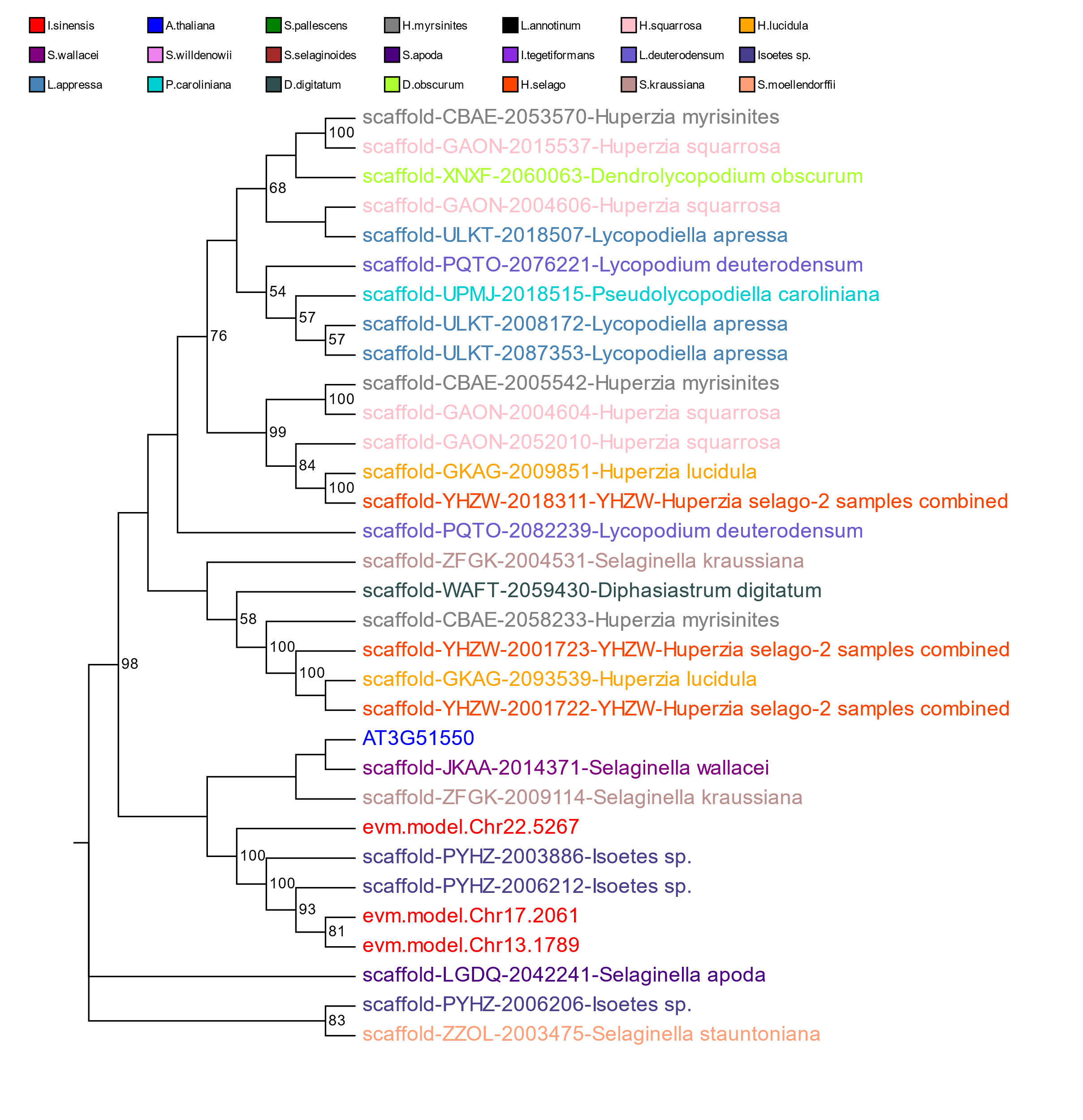


**Dataset S49. Phylogenetic relationships of FER proteins from *I. sinensis* and other lycophyte species.** Numbers on the major branches indicate bootstrap values (> 50%) in 1,000 replicates.


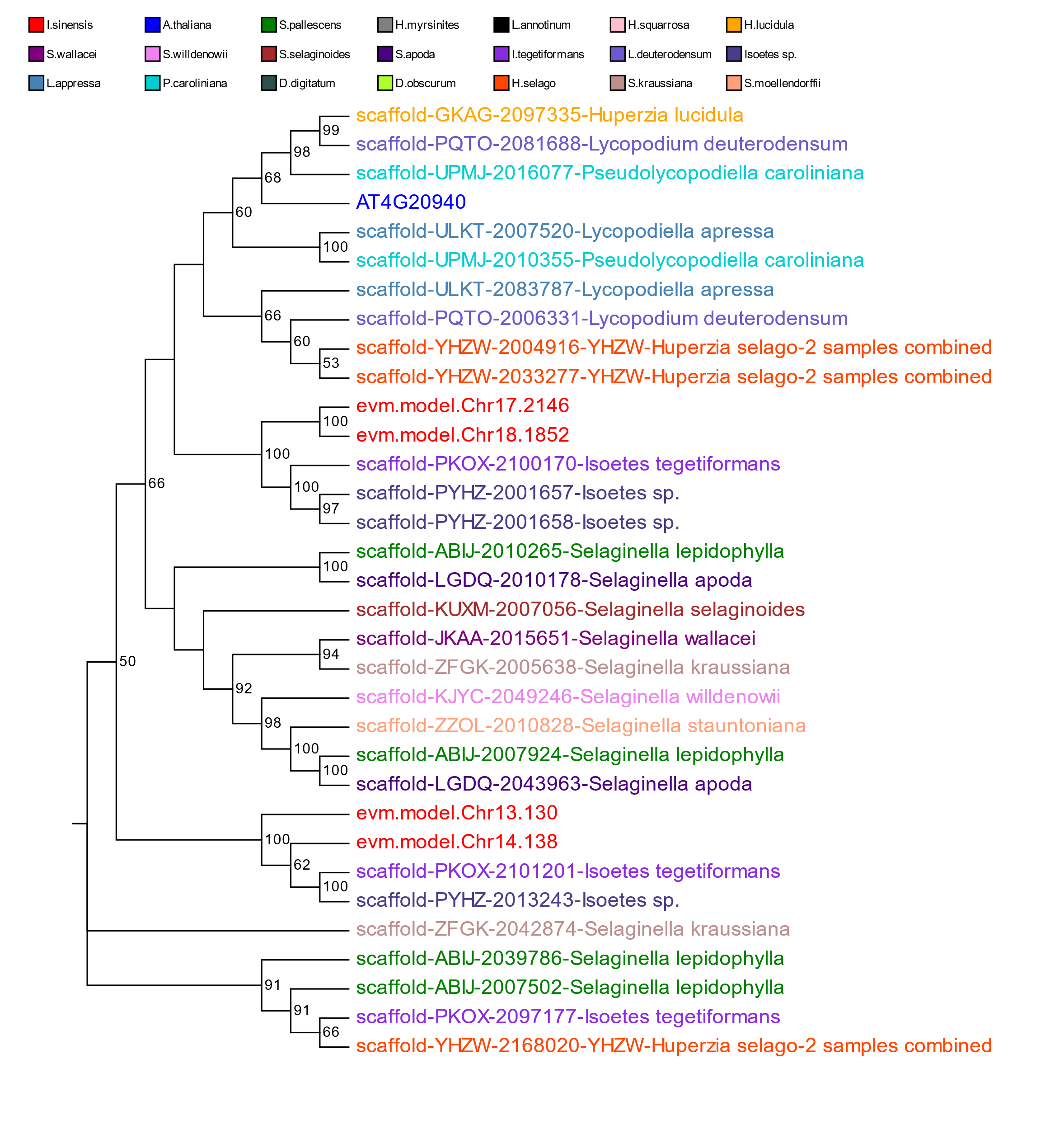


**Dataset S50. Phylogenetic relationships of GHR1 proteins from *I. sinensis* and other lycophyte species.** Numbers on the major branches indicate bootstrap values (> 50%) in 1,000 replicates.


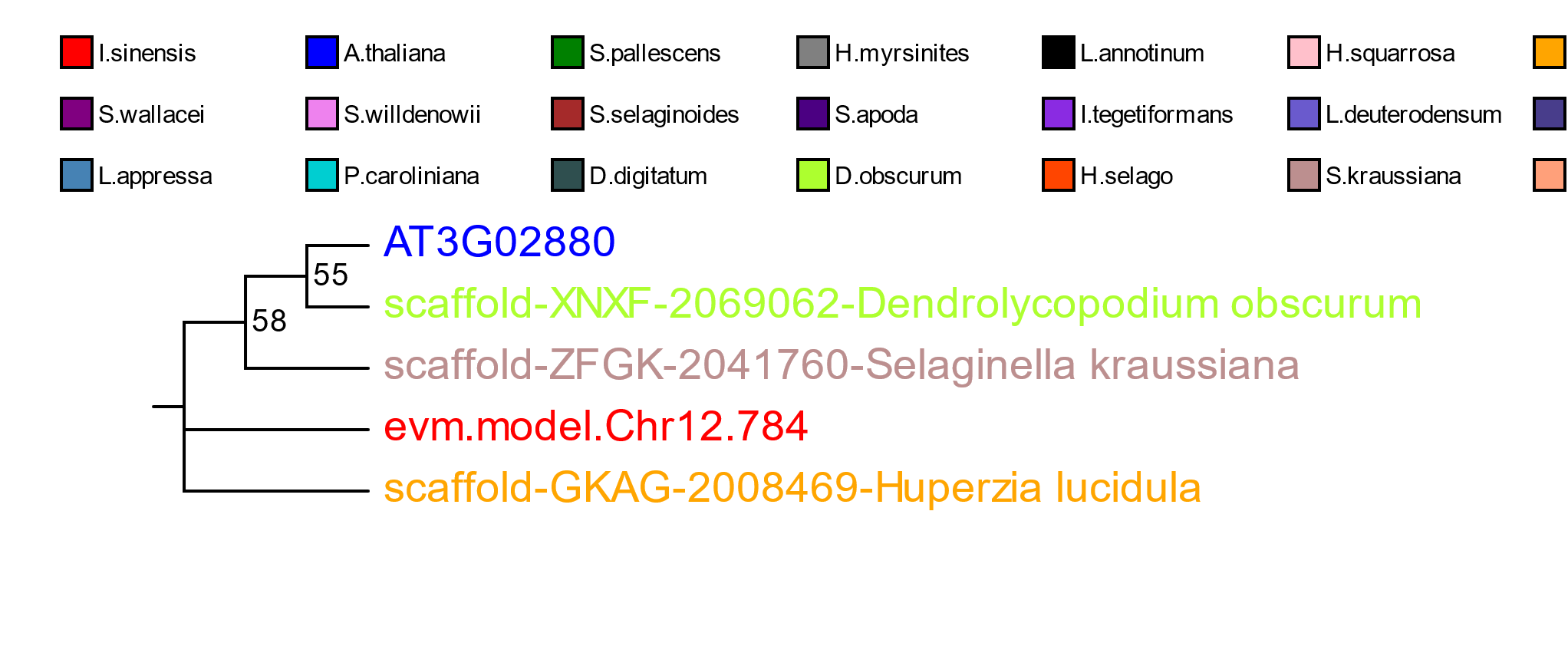


**Dataset S51. Phylogenetic relationships of KIN7 proteins from *I. sinensis* and other lycophyte species.** Numbers on the major branches indicate bootstrap values (> 50%) in 1,000 replicates.


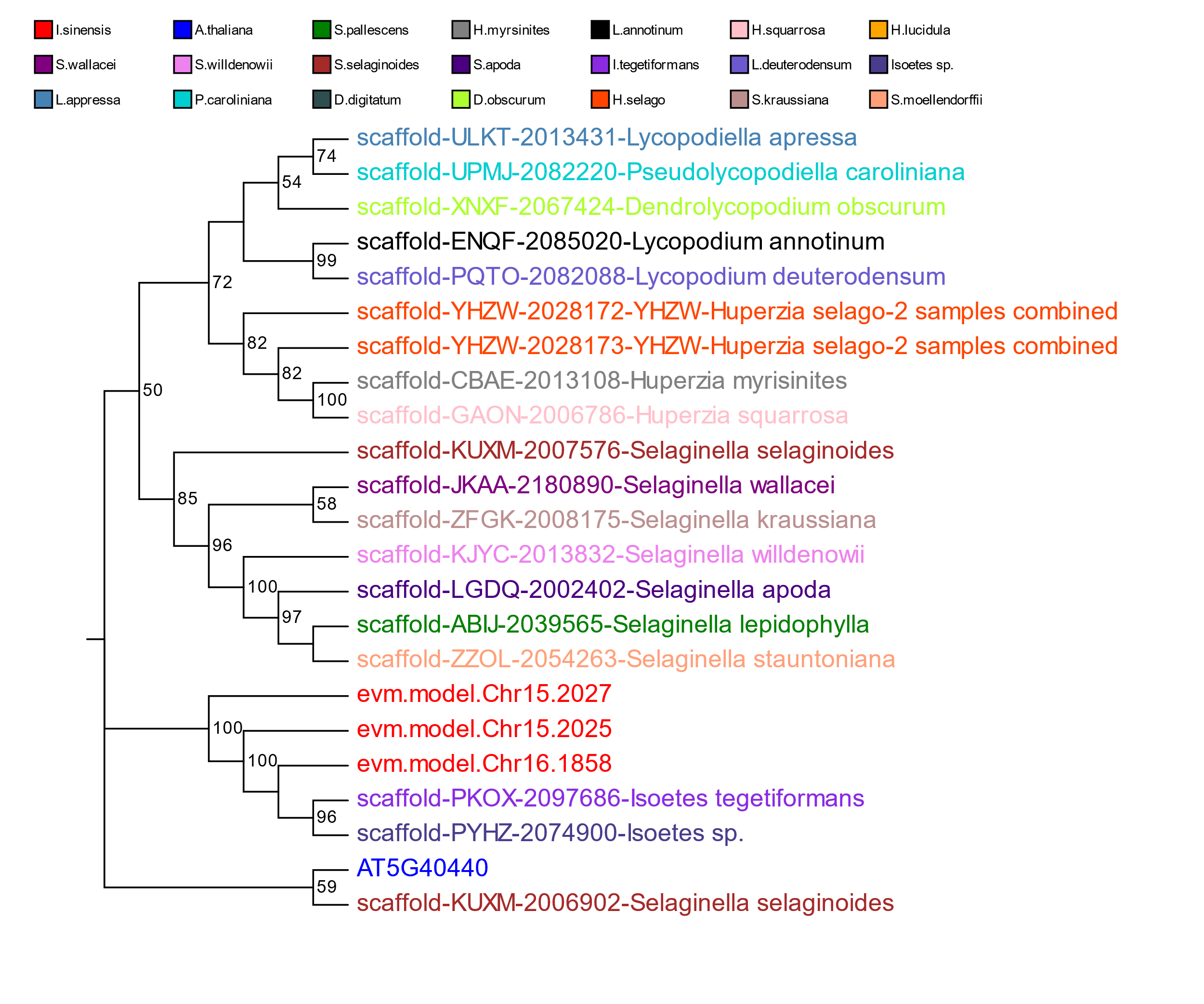


**Dataset S52. Phylogenetic relationships of MKK3 proteins from *I. sinensis* and other lycophyte species.** Numbers on the major branches indicate bootstrap values (> 50%) in 1,000 replicates.


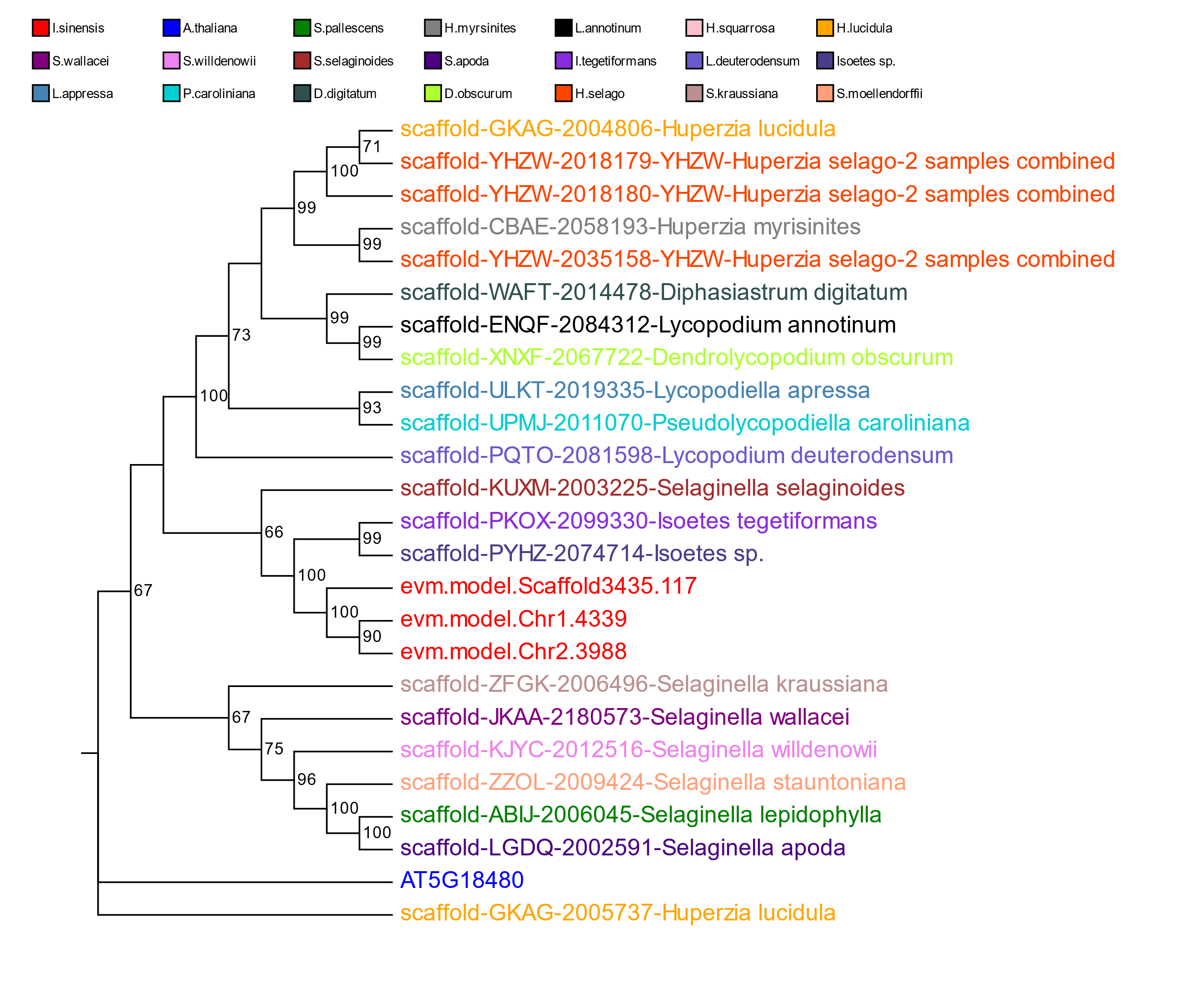


**Dataset S53. Phylogenetic relationships of MOCA1 proteins from *I. sinensis* and other lycophyte species.** Numbers on the major branches indicate bootstrap values (> 50%) in 1,000 replicates.


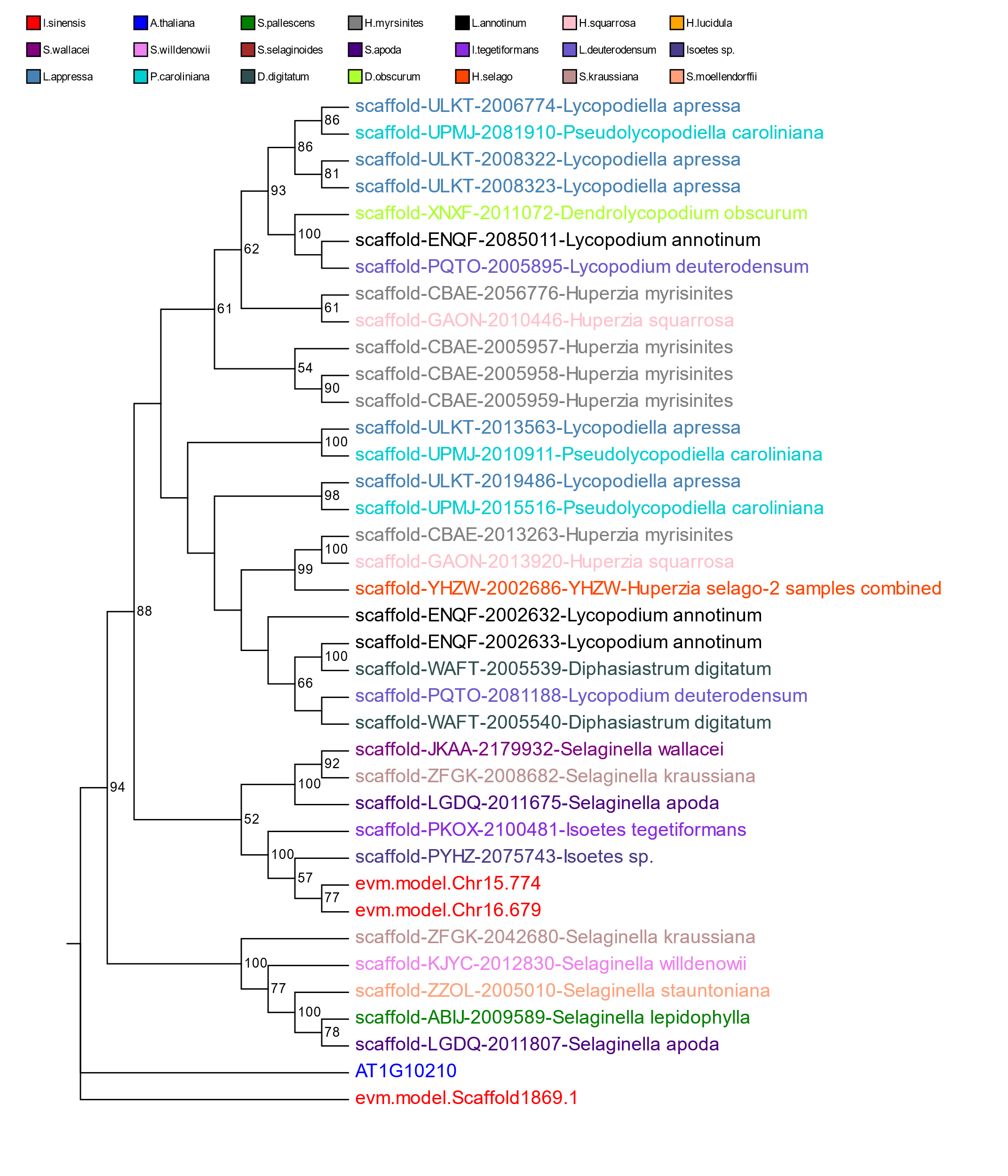


**Dataset S54. Phylogenetic relationships of MPK1 proteins from *I. sinensis* and other lycophyte species.** Numbers on the major branches indicate bootstrap values (> 50%) in 1,000 replicates.


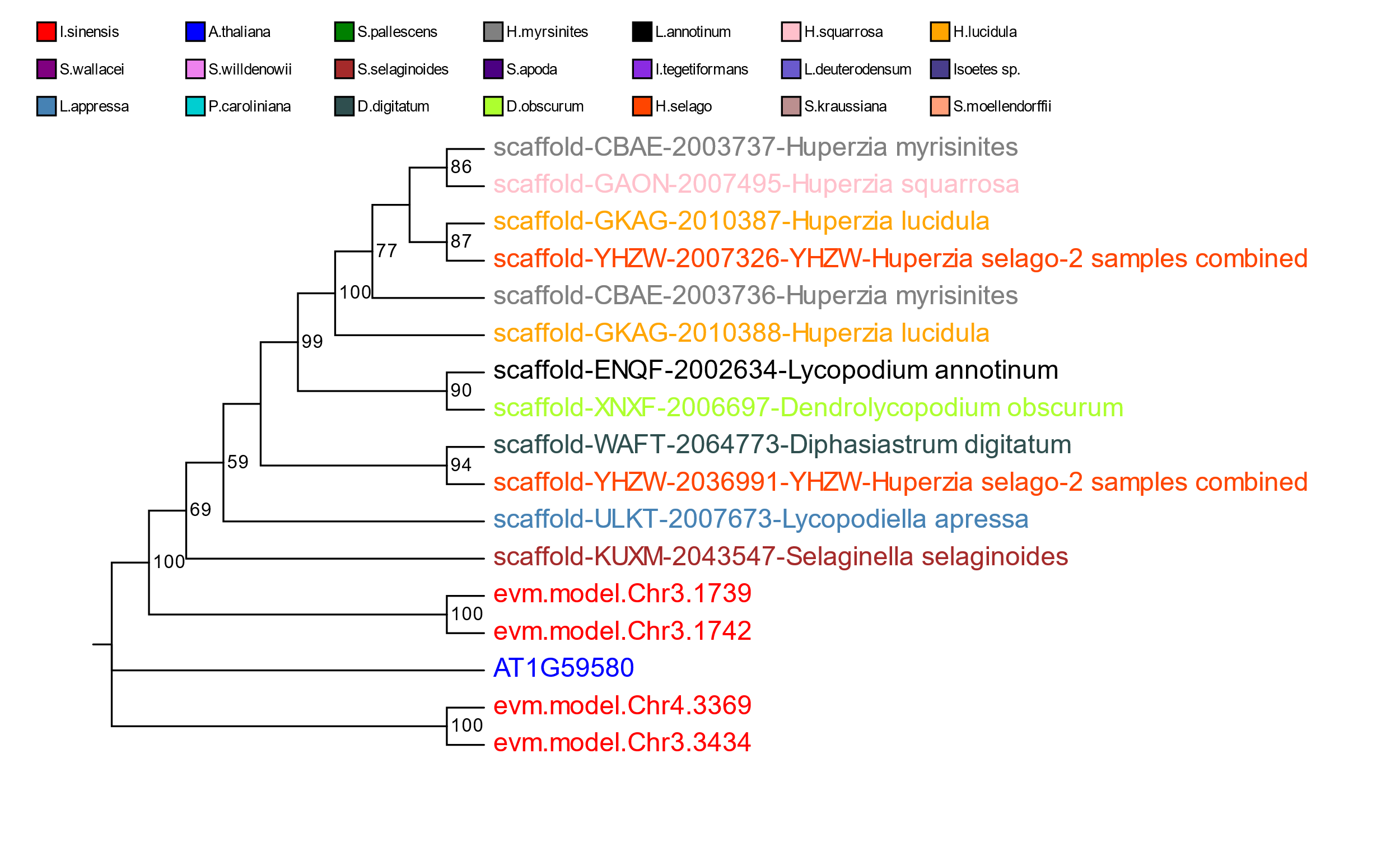


**Dataset S55.** **Phylogenetic relationships of MPK2 proteins from *I. sinensis* and other lycophyte species.** Numbers on the major branches indicate bootstrap values (> 50%) in 1,000 replicates.


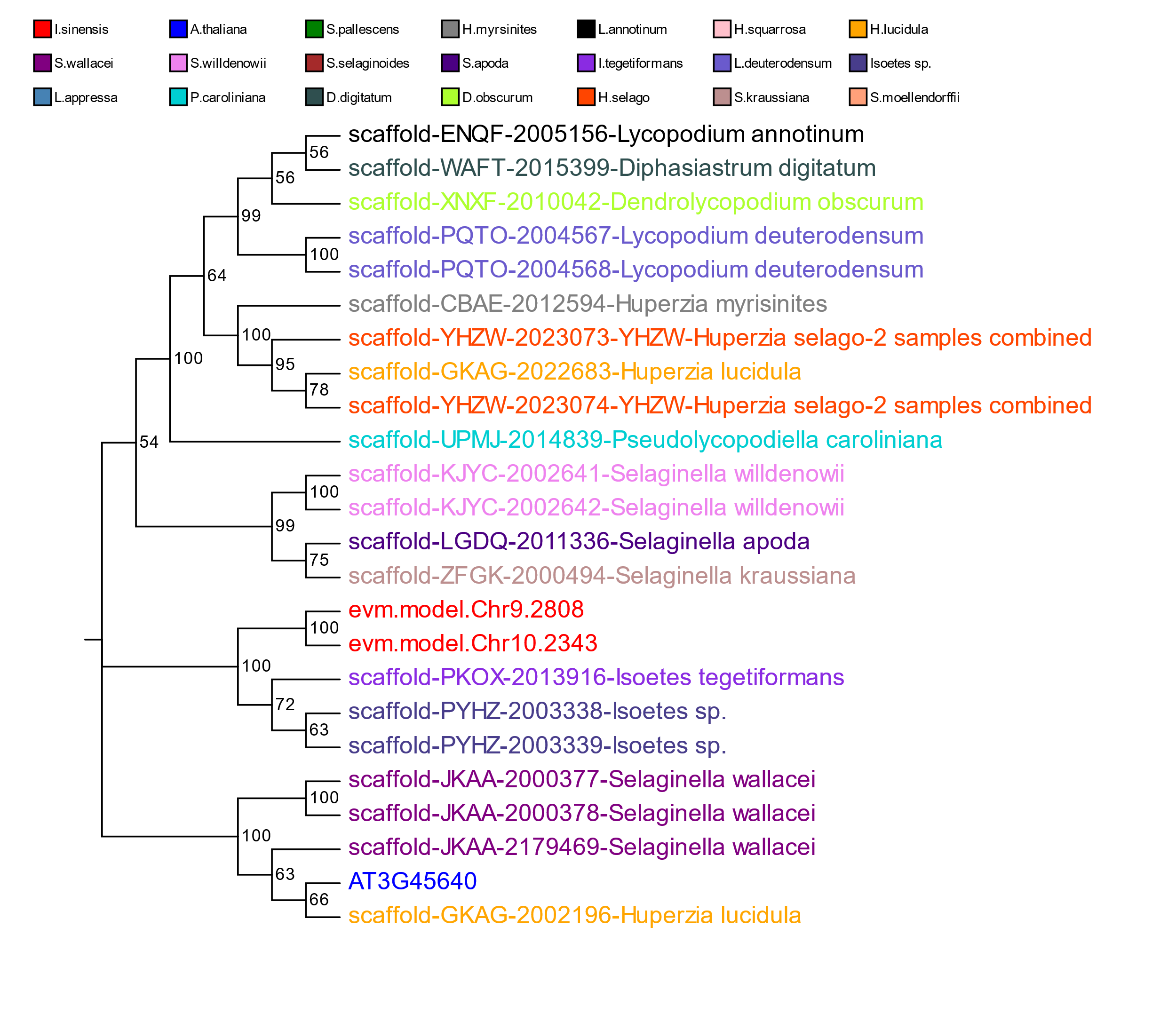


**Dataset S56. Phylogenetic relationships of MPK3 proteins from *I. sinensis* and other lycophyte species.** Numbers on the major branches indicate bootstrap values (> 50%) in 1,000 replicates.


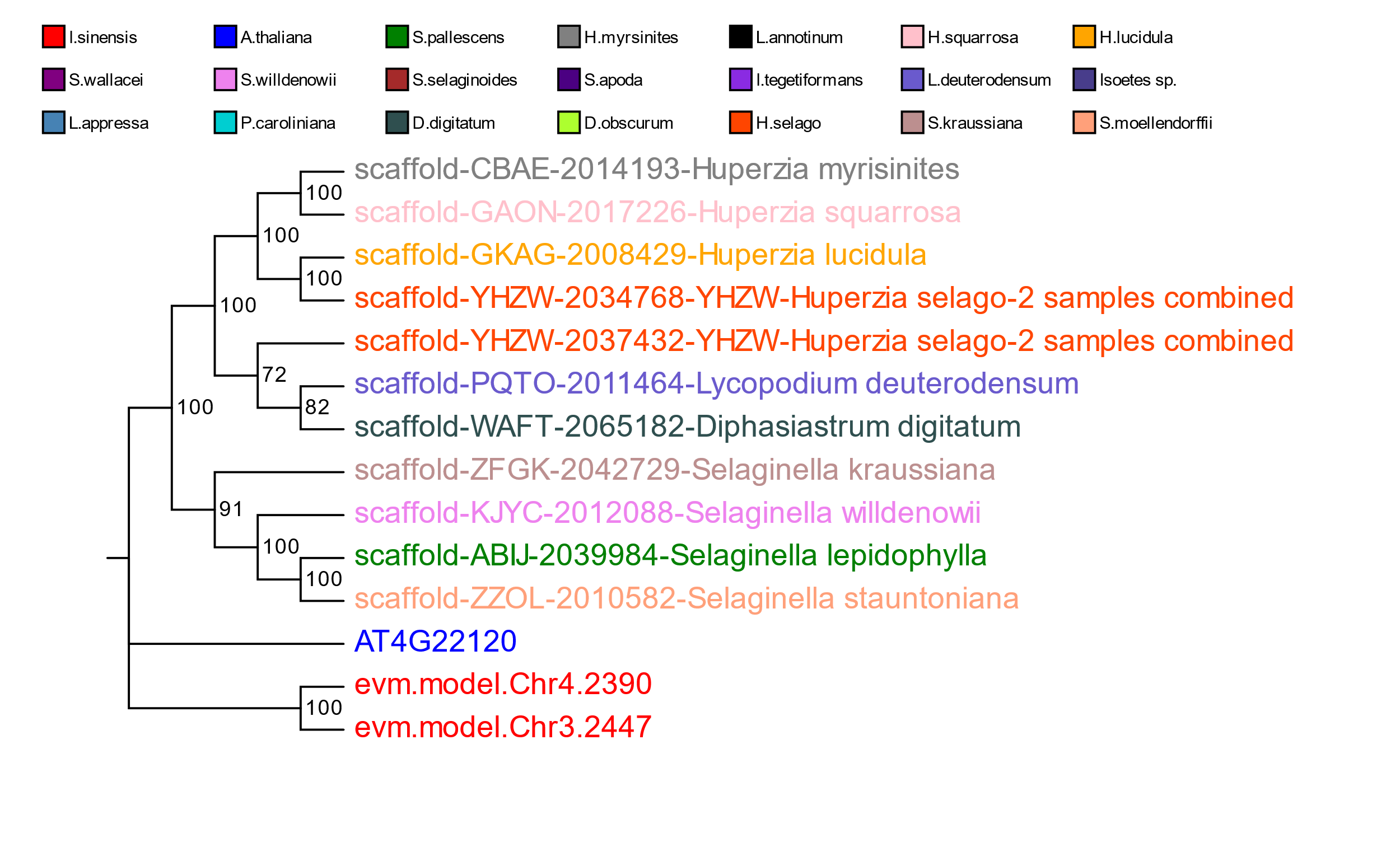


**Dataset S57. Phylogenetic relationships of OSCA1.2 proteins from *I. sinensis* and other lycophyte species.** Numbers on the major branches indicate bootstrap values (> 50%) in 1,000 replicates.


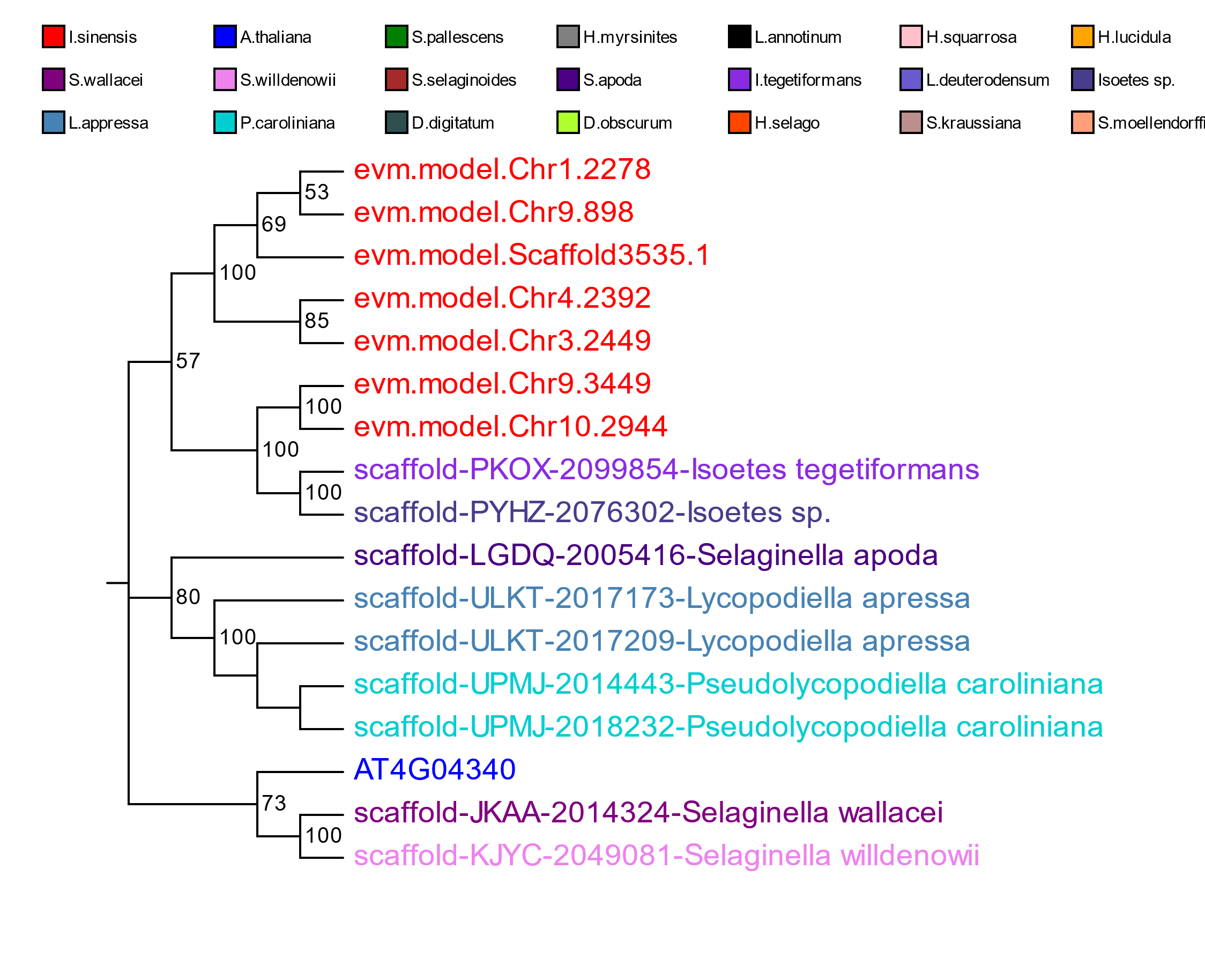


**Dataset S58. Phylogenetic relationships of OSCA2 proteins from *I. sinensis* and other lycophyte species.** Numbers on the major branches indicate bootstrap values (> 50%) in 1,000 replicates.


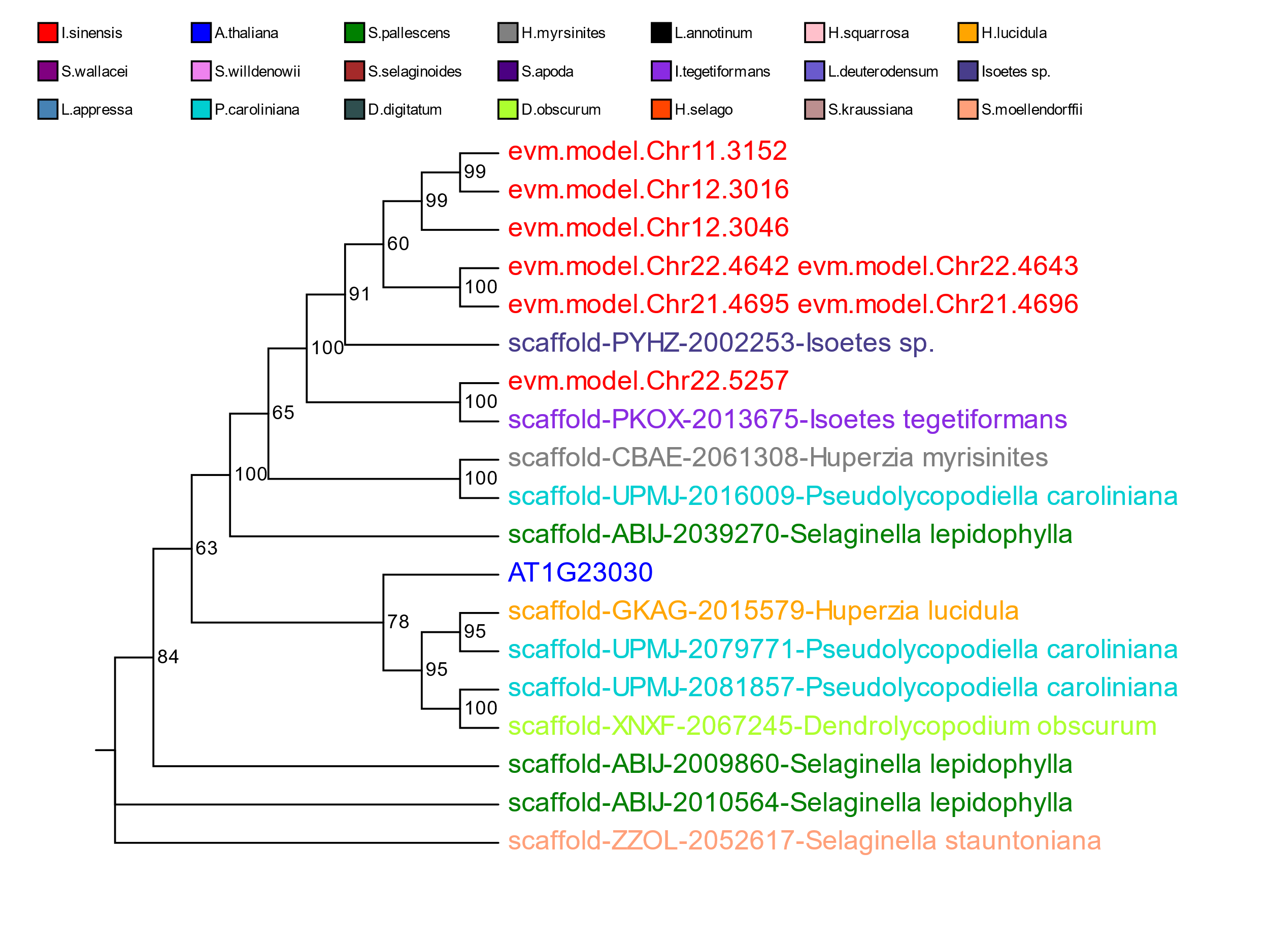


**Dataset S59. Phylogenetic relationships of PUB11 proteins from *I. sinensis* and other lycophyte species.** Numbers on the major branches indicate bootstrap values (> 50%) in 1,000 replicates.


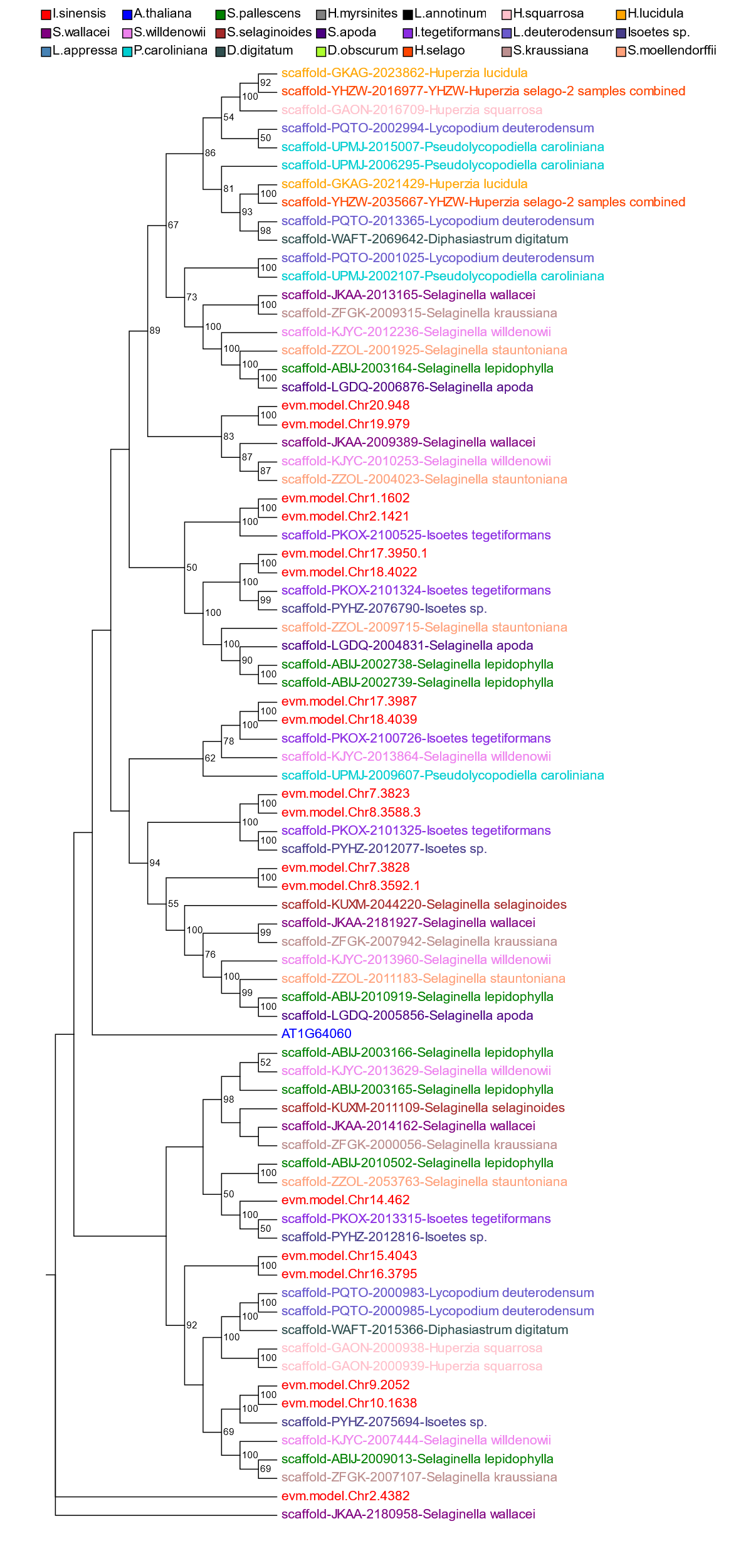


**Dataset S60. Phylogenetic relationships of RbohF proteins from *I. sinensis* and other lycophyte species.** Numbers on the major branches indicate bootstrap values (> 50%) in 1,000 replicates.


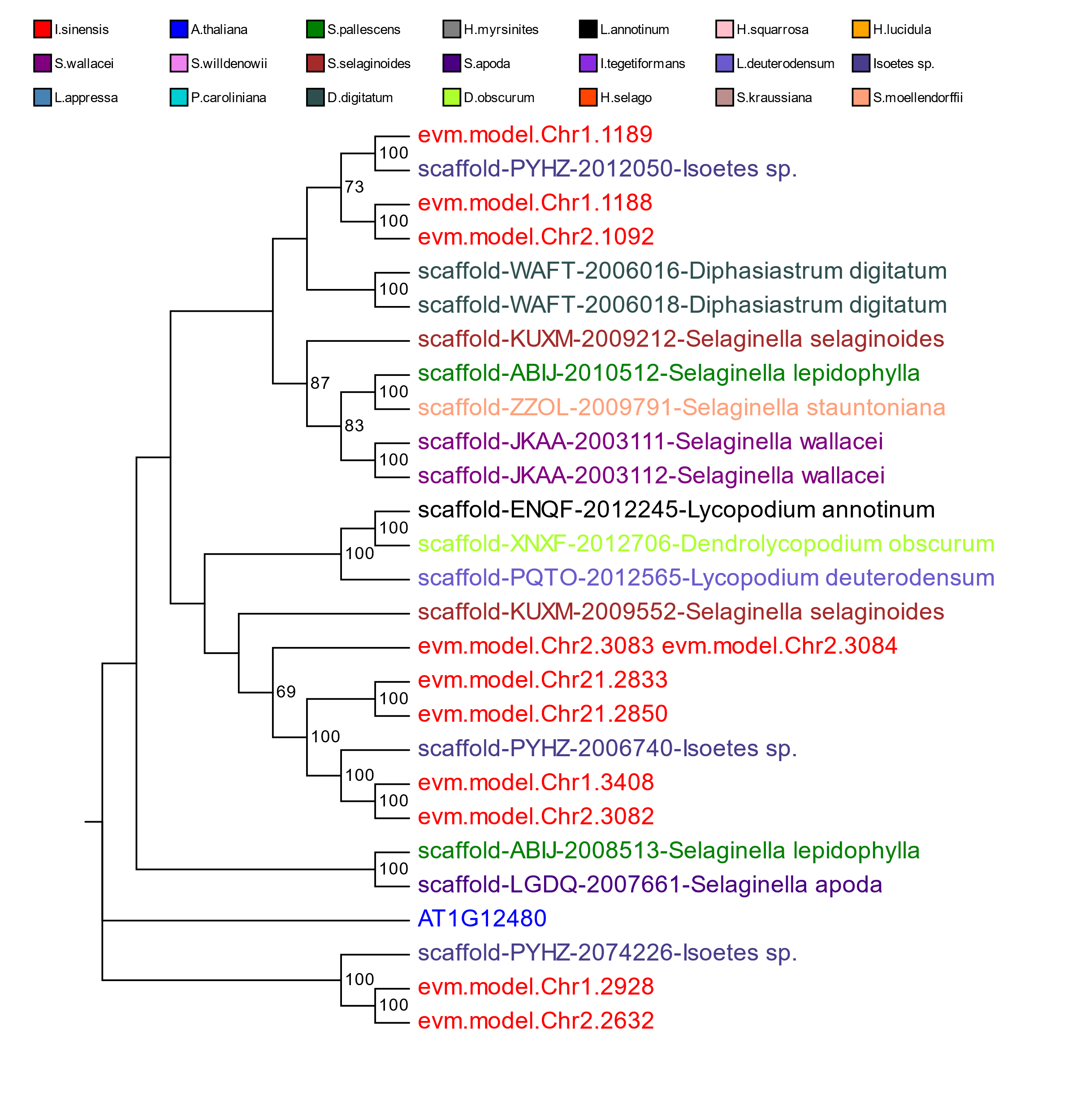


**Dataset S61. Phylogenetic relationships of SLAC1 proteins from *I. sinensis* and other lycophyte species.** Numbers on the major branches indicate bootstrap values (> 50%) in 1,000 replicates.


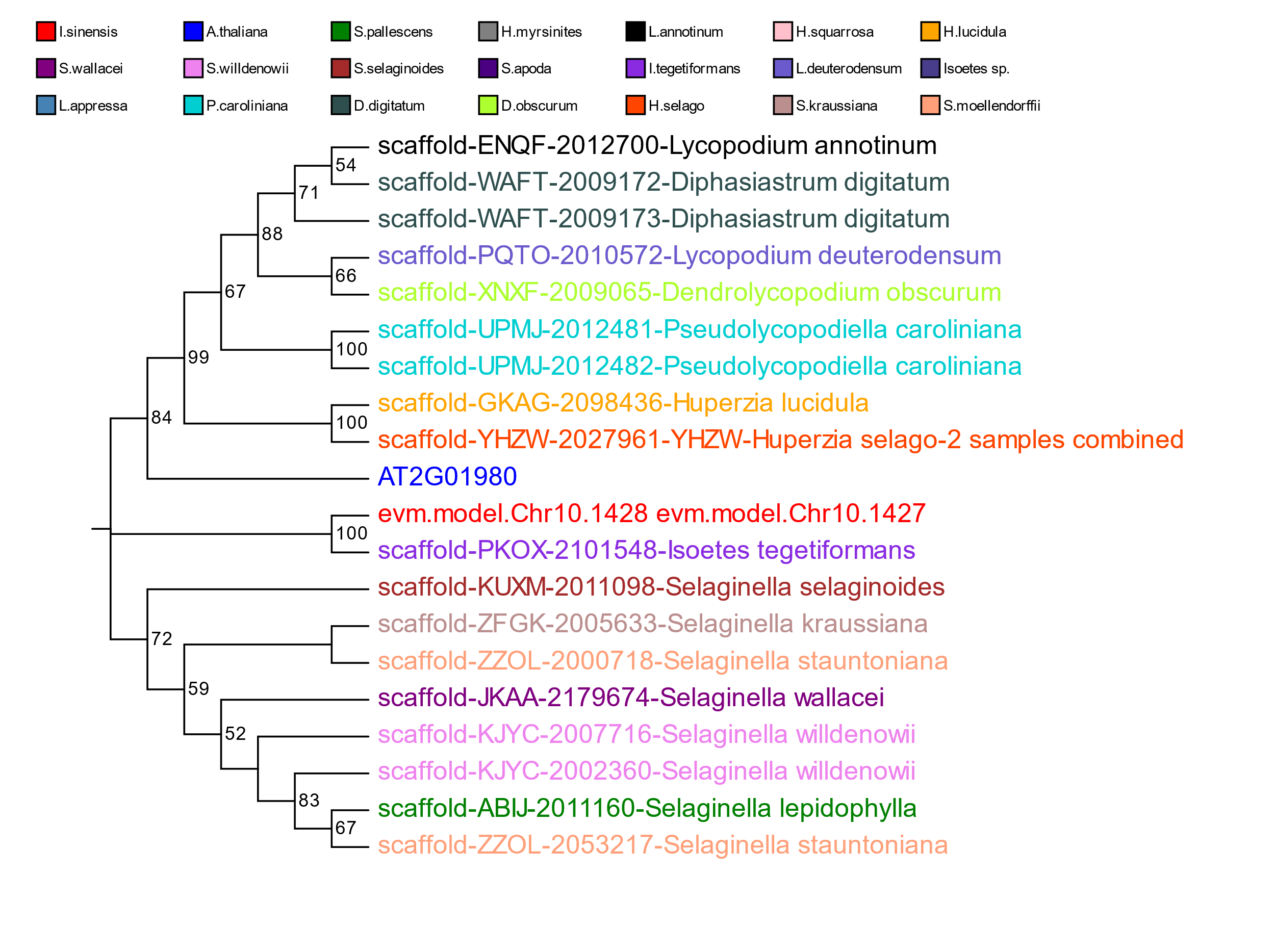


**Dataset S62. Phylogenetic relationships of SOS1 proteins from *I. sinensis* and other lycophyte species.** Numbers on the major branches indicate bootstrap values (> 50%) in 1,000 replicates.


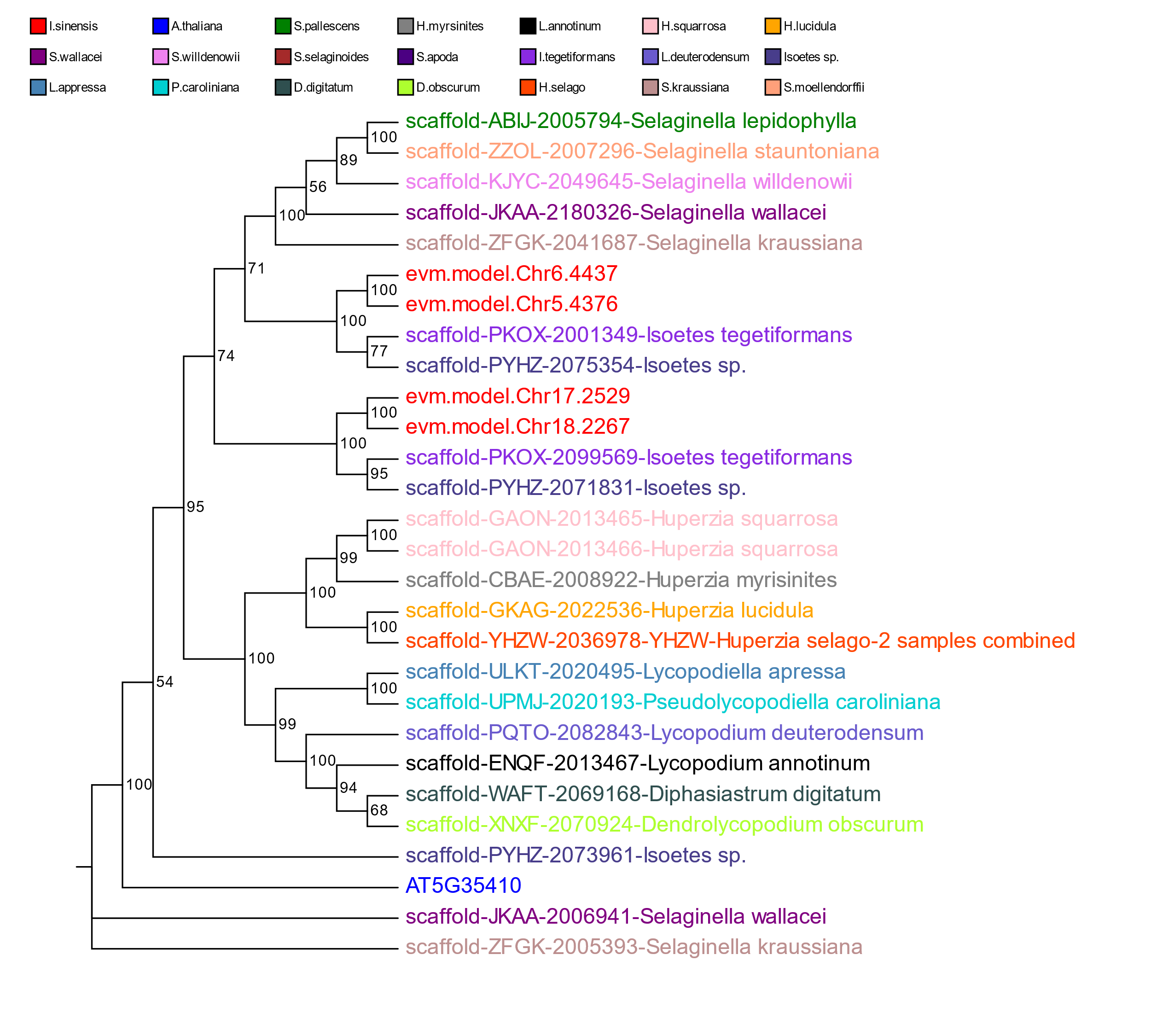


**Dataset S63.** **Phylogenetic relationships of SOS2 proteins from *I. sinensis* and other lycophyte species.** Numbers on the major branches indicate bootstrap values (> 50%) in 1,000 replicates.


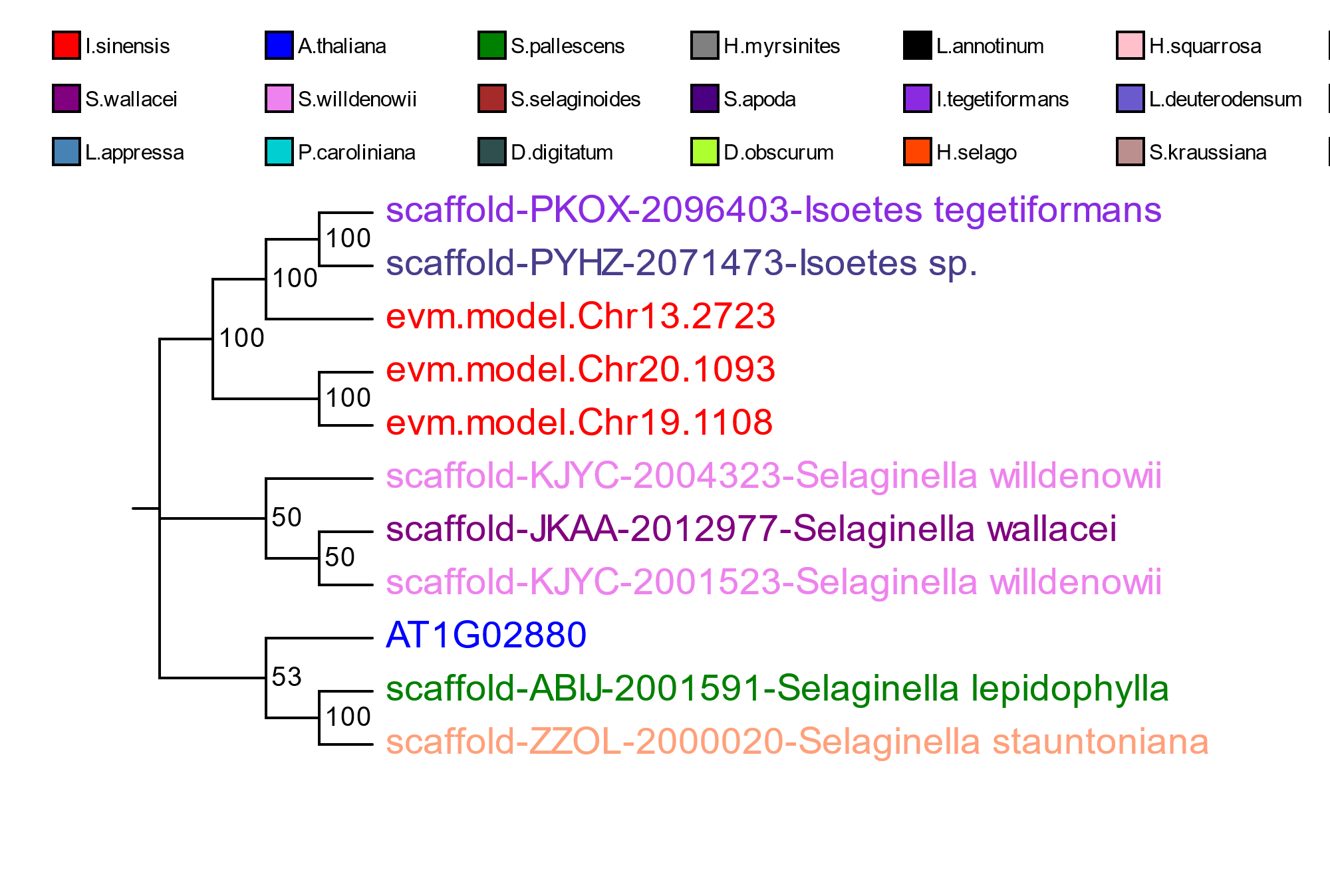


**Dataset S64.** **Phylogenetic relationships of TPK1 proteins from *I. sinensis* and other lycophyte species.** Numbers on the major branches indicate bootstrap values (> 50%) in 1,000 replicates.


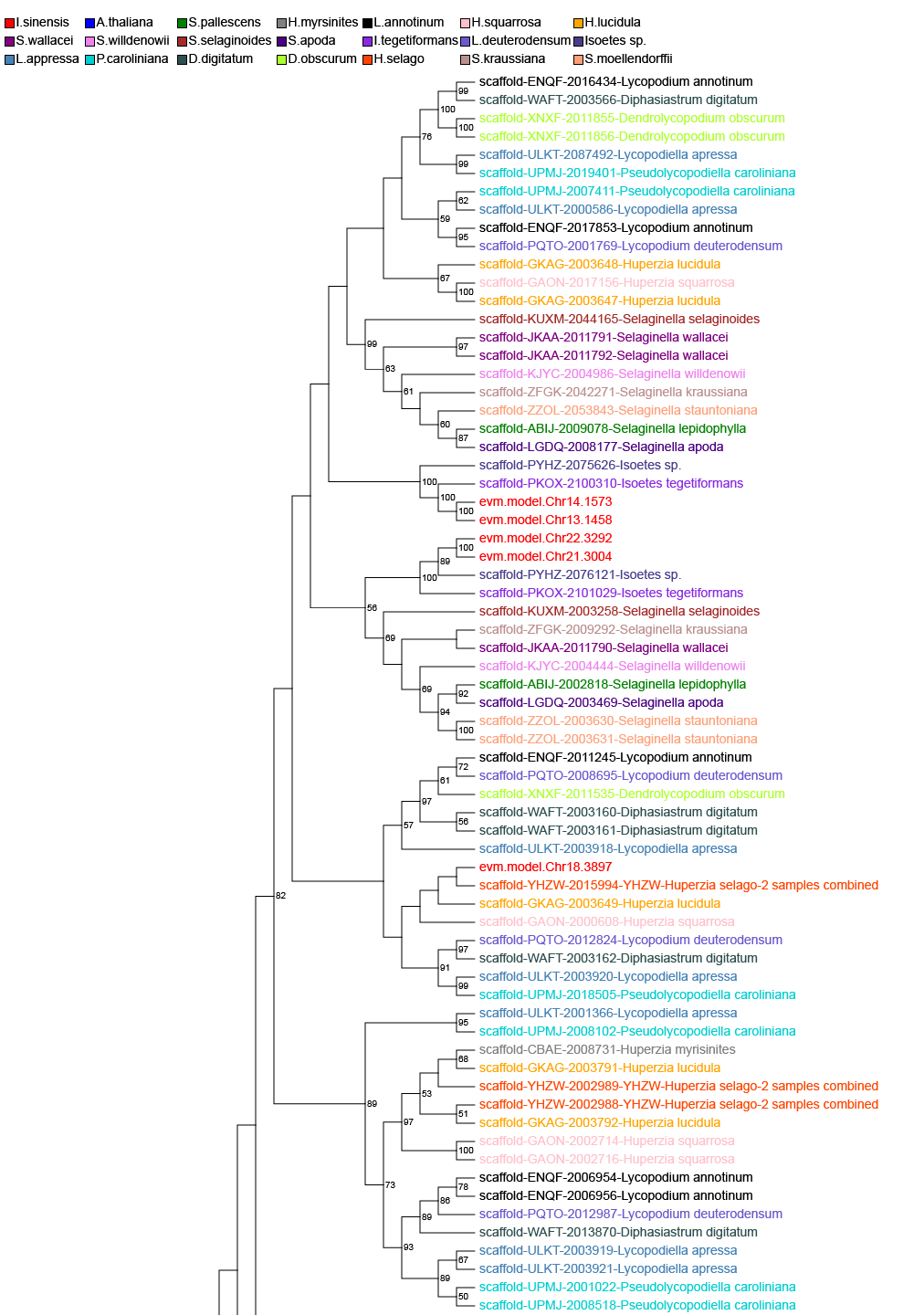

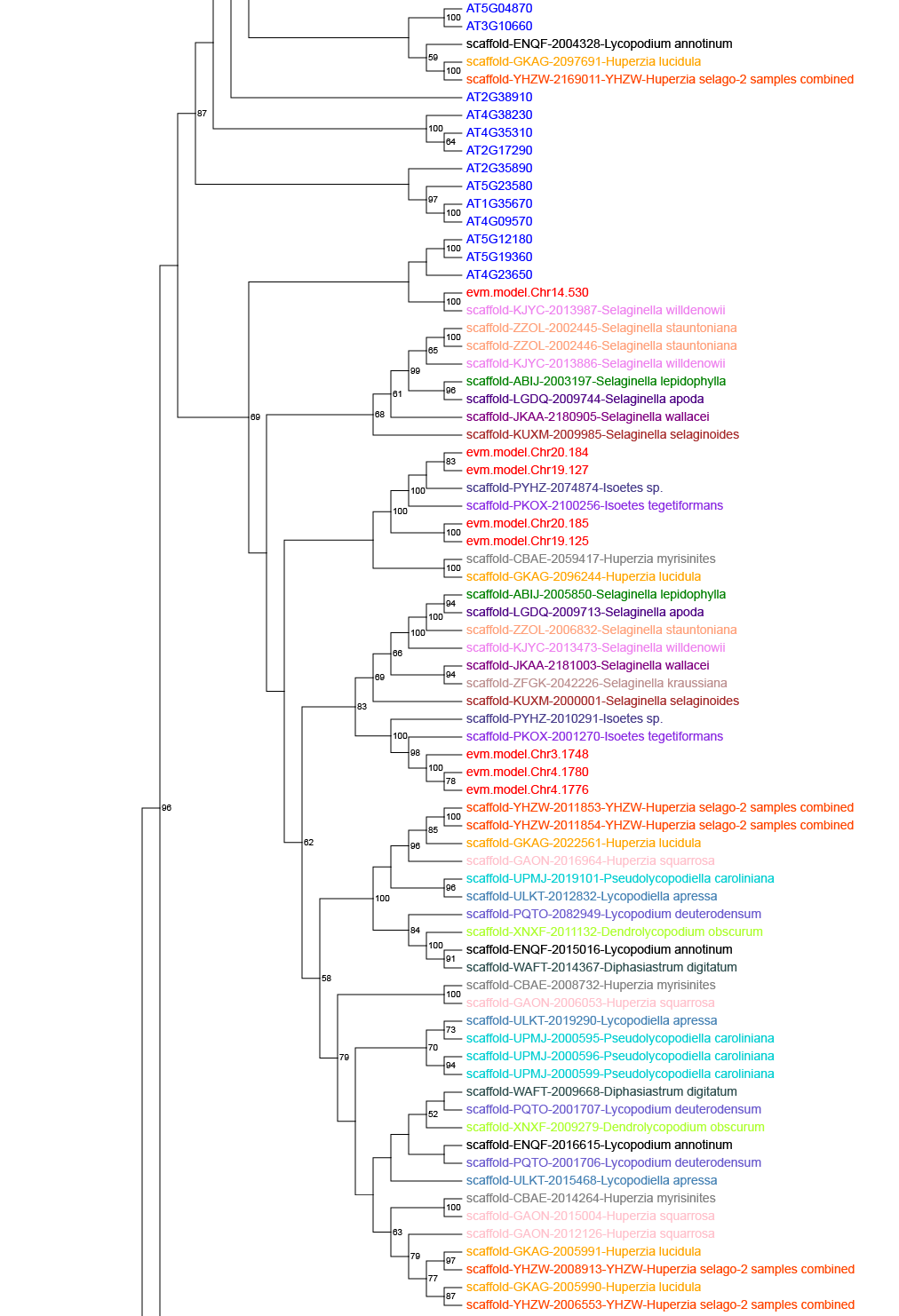

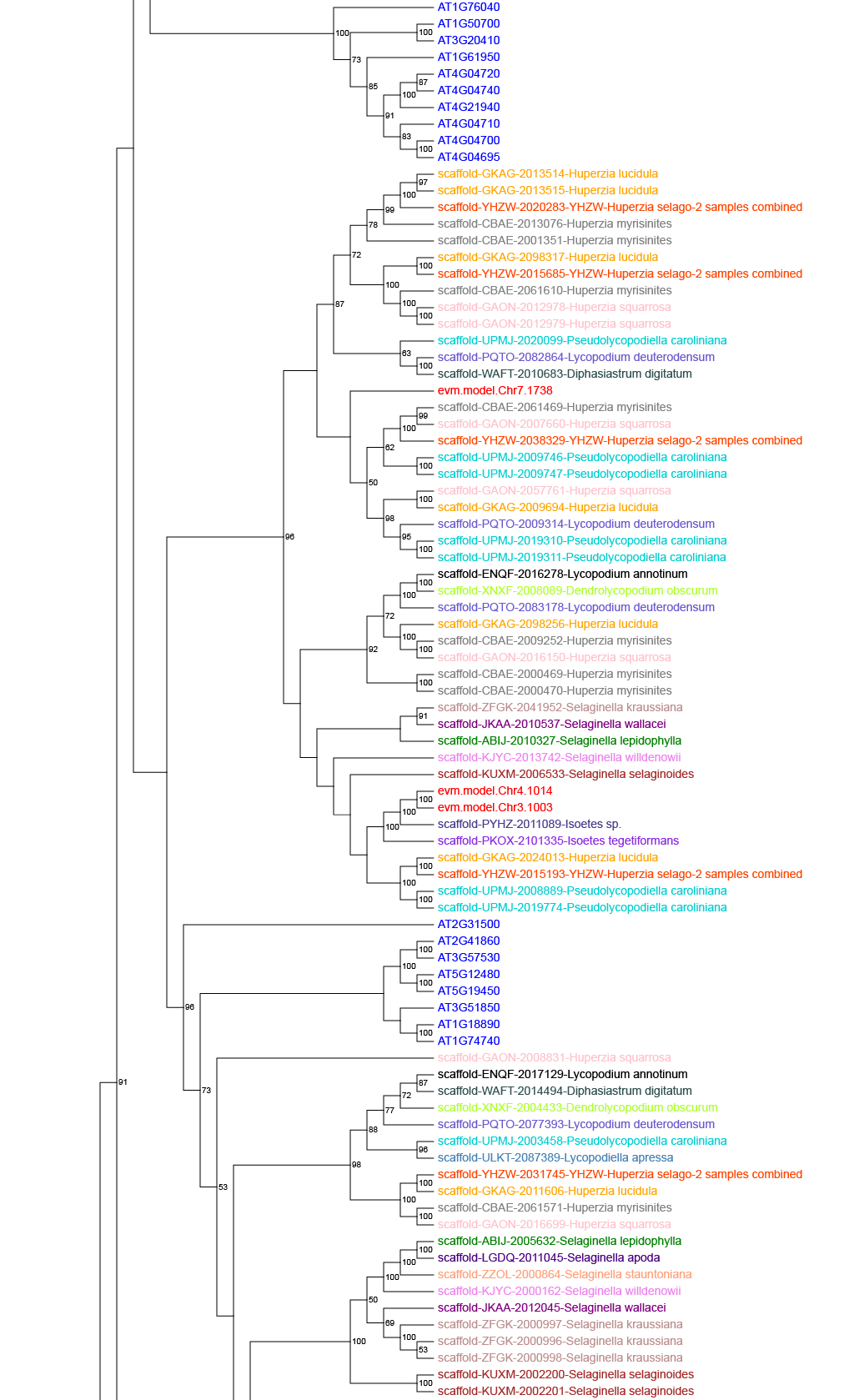


**
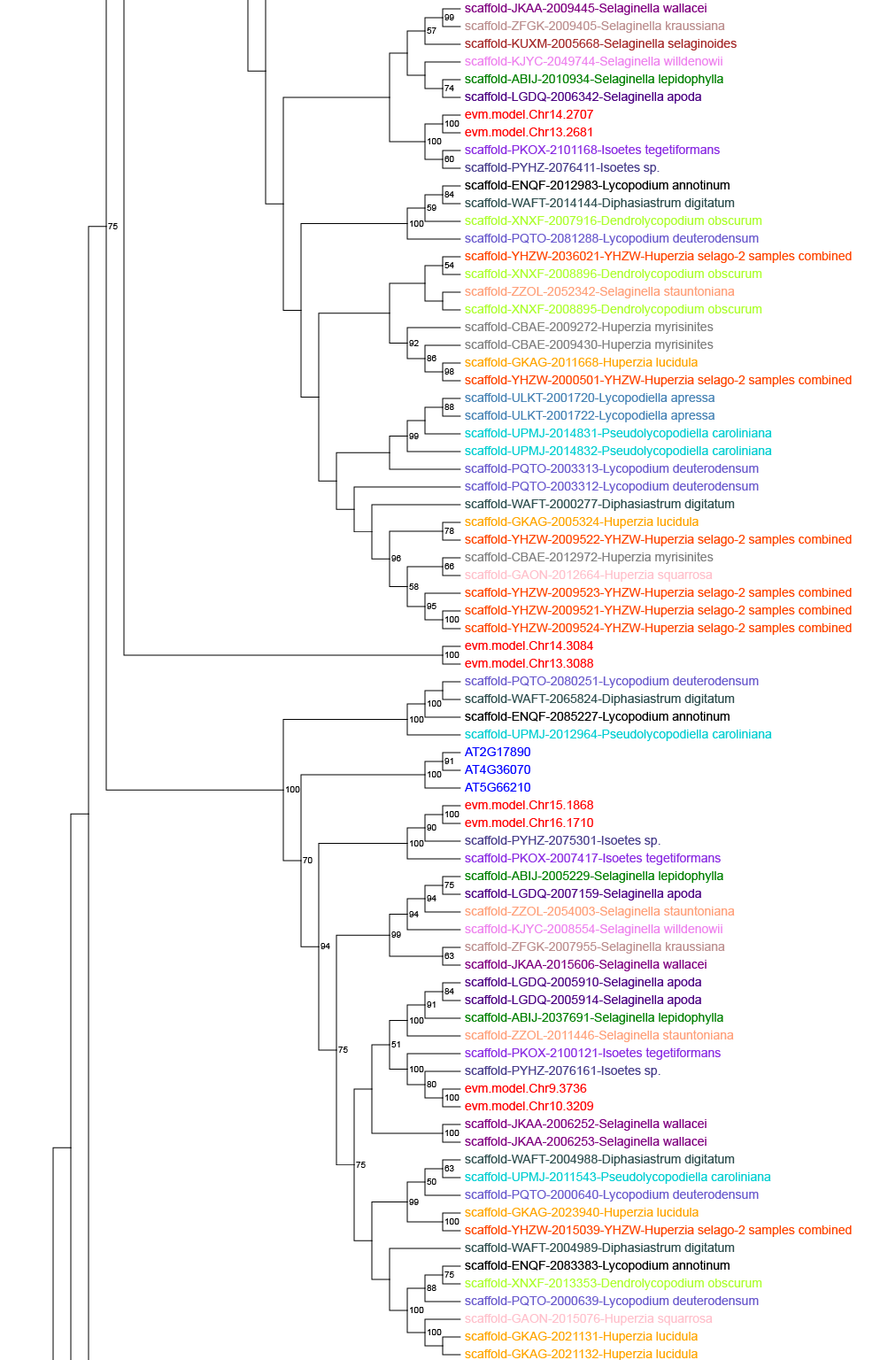

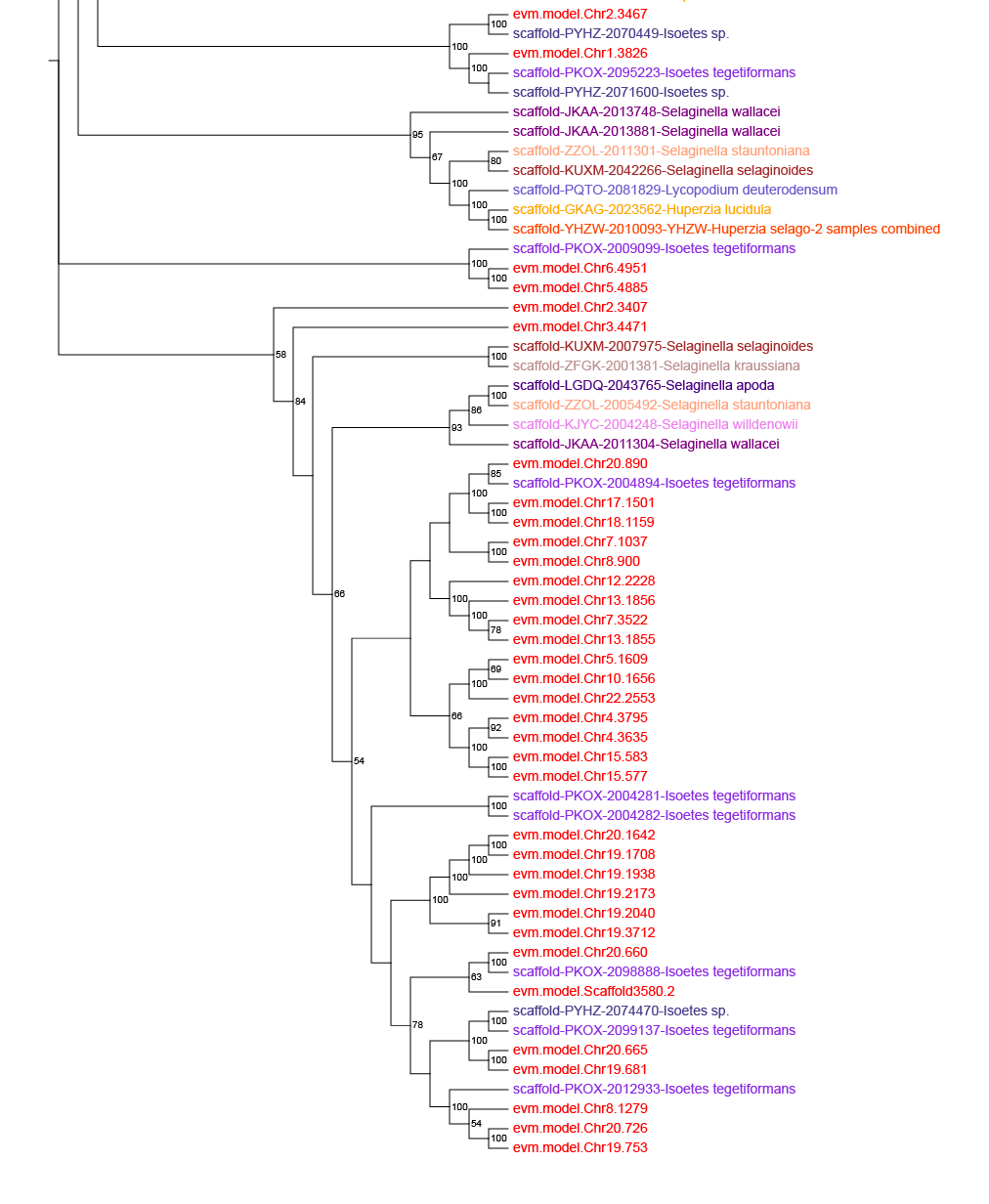
Dataset S65. Phylogenetic relationships of CPK proteins from *I. sinensis* and other lycophyte species.** Numbers on the major branches indicate bootstrap values (> 50%) in 1,000 replicates.

**
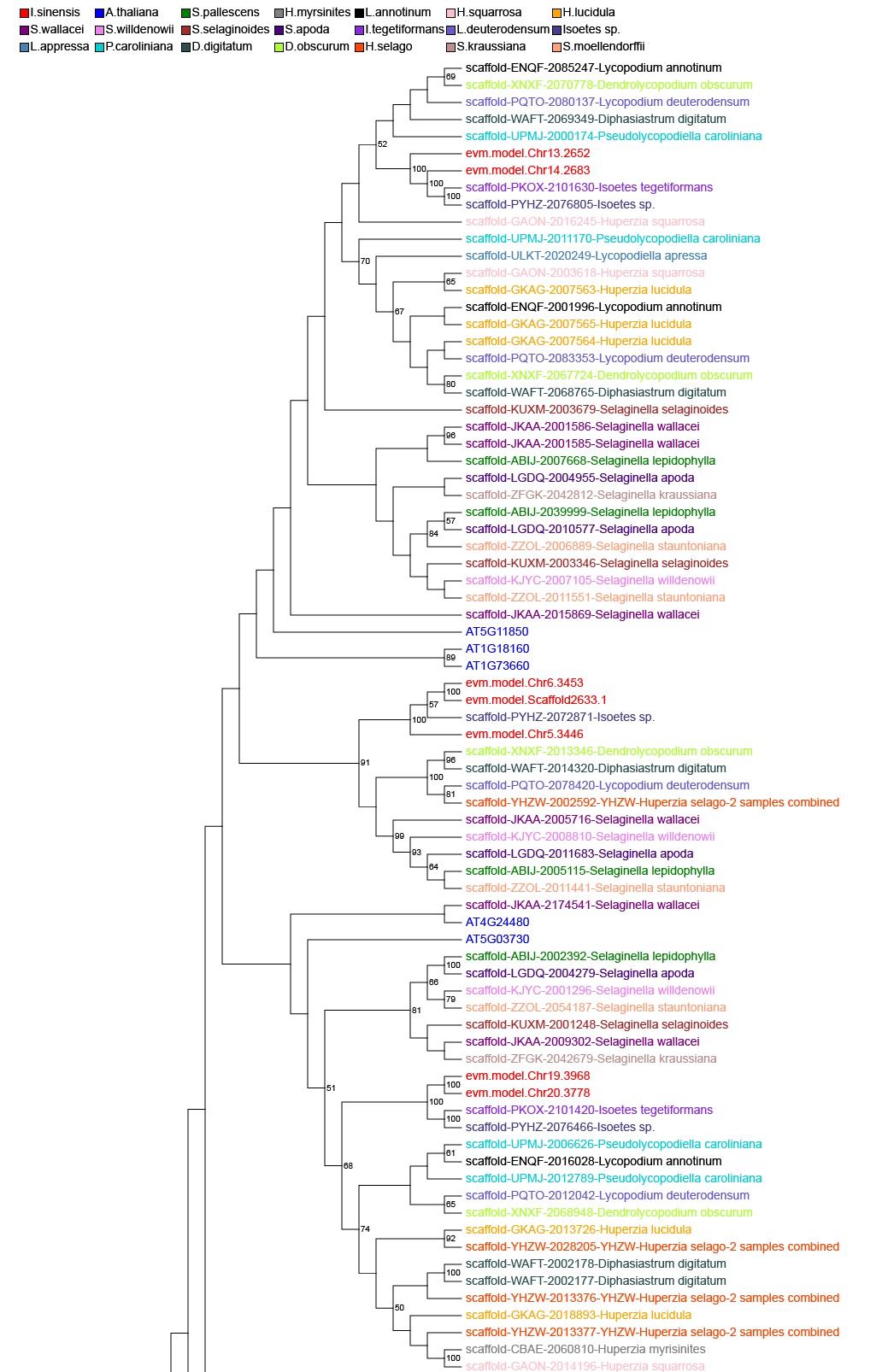

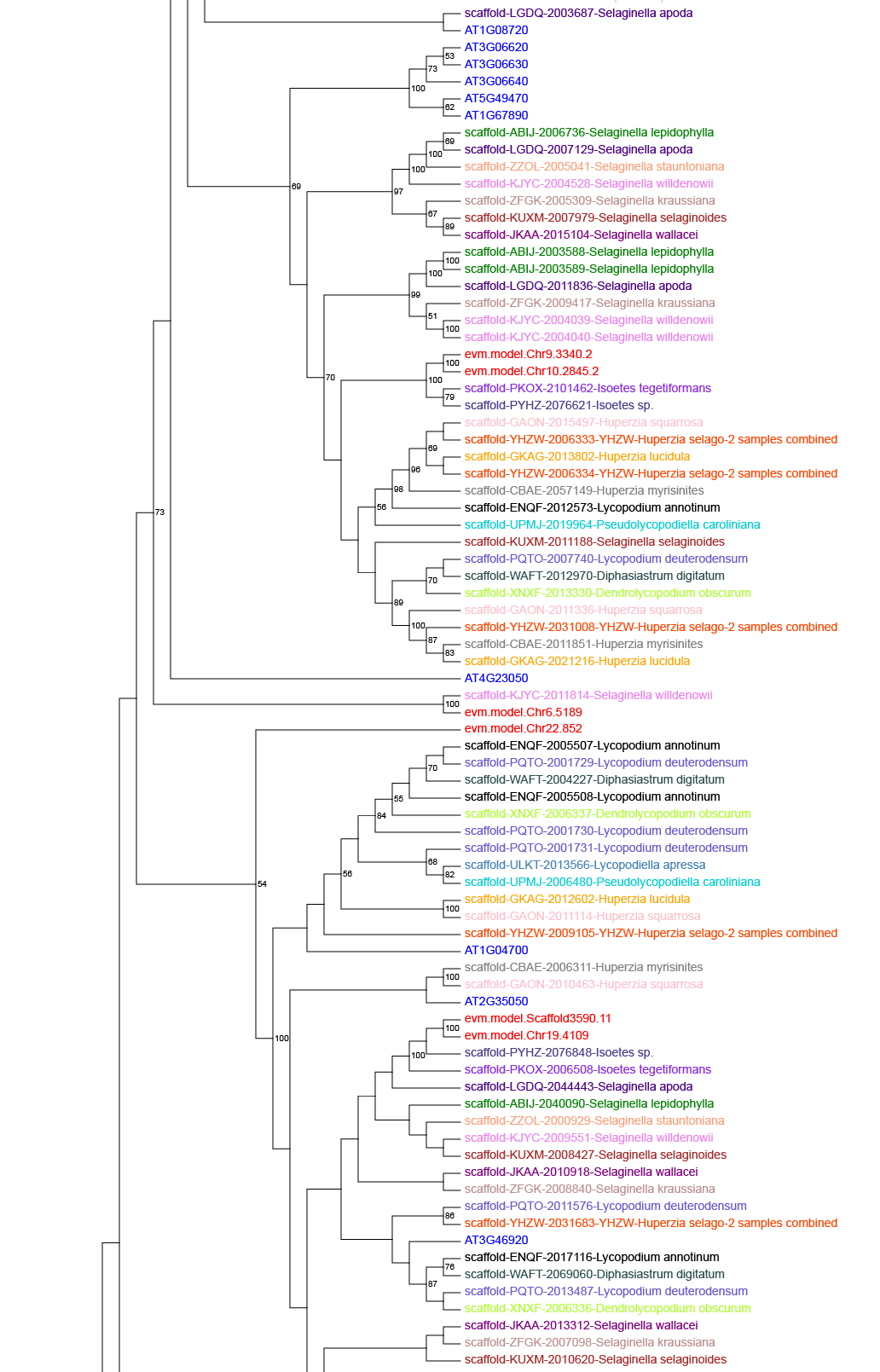

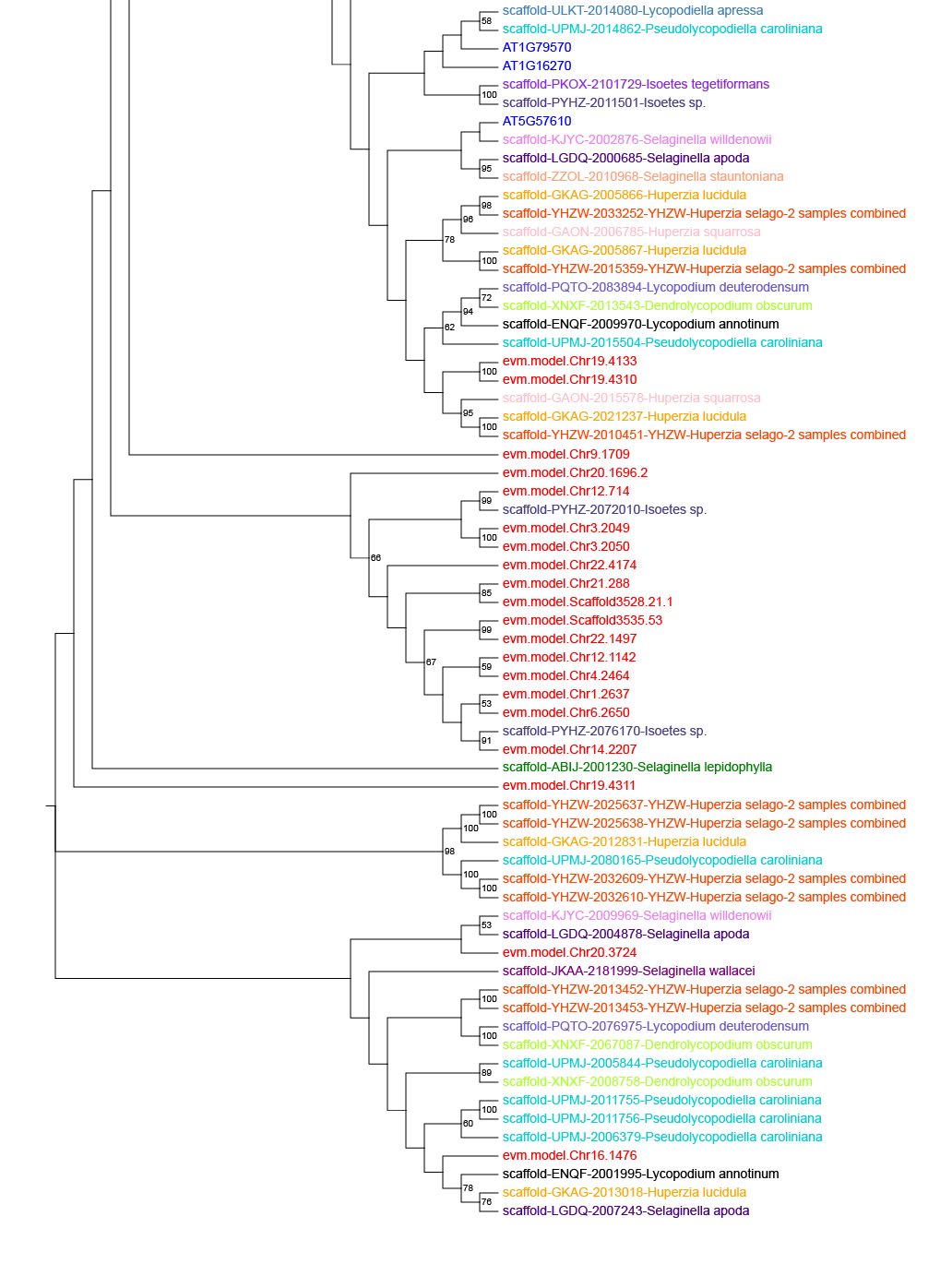
**

**Dataset S66. Phylogenetic relationships of RAF proteins from *I. sinensis* and other lycophyte species.** Numbers on the major branches indicate bootstrap values (> 50%) in 1,000 replicates.


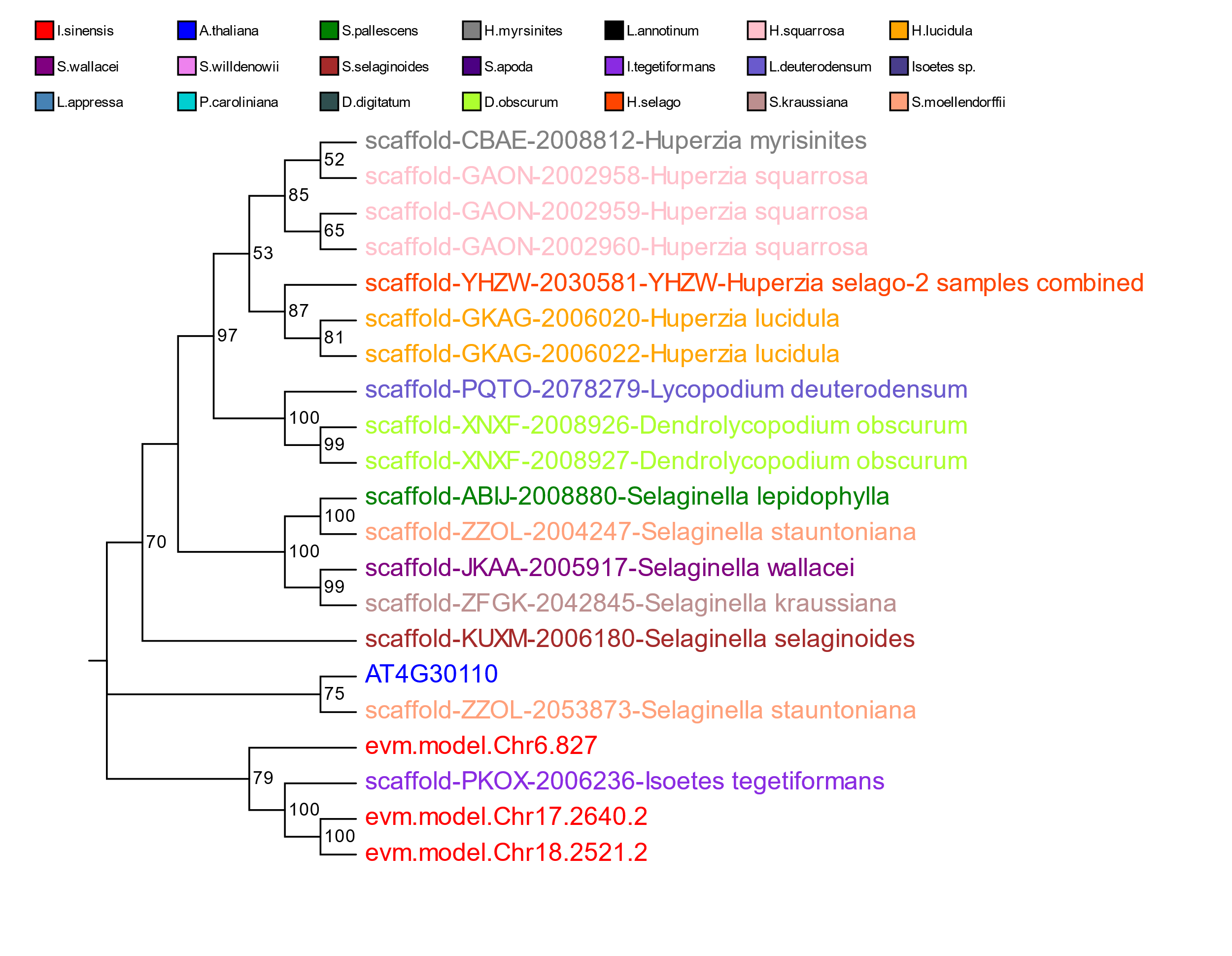


**Dataset S67. Phylogenetic relationships of HMA2 proteins from *I. sinensis* and other lycophyte species.** Numbers on the major branches indicate bootstrap values (> 50%) in 1,000 replicates.


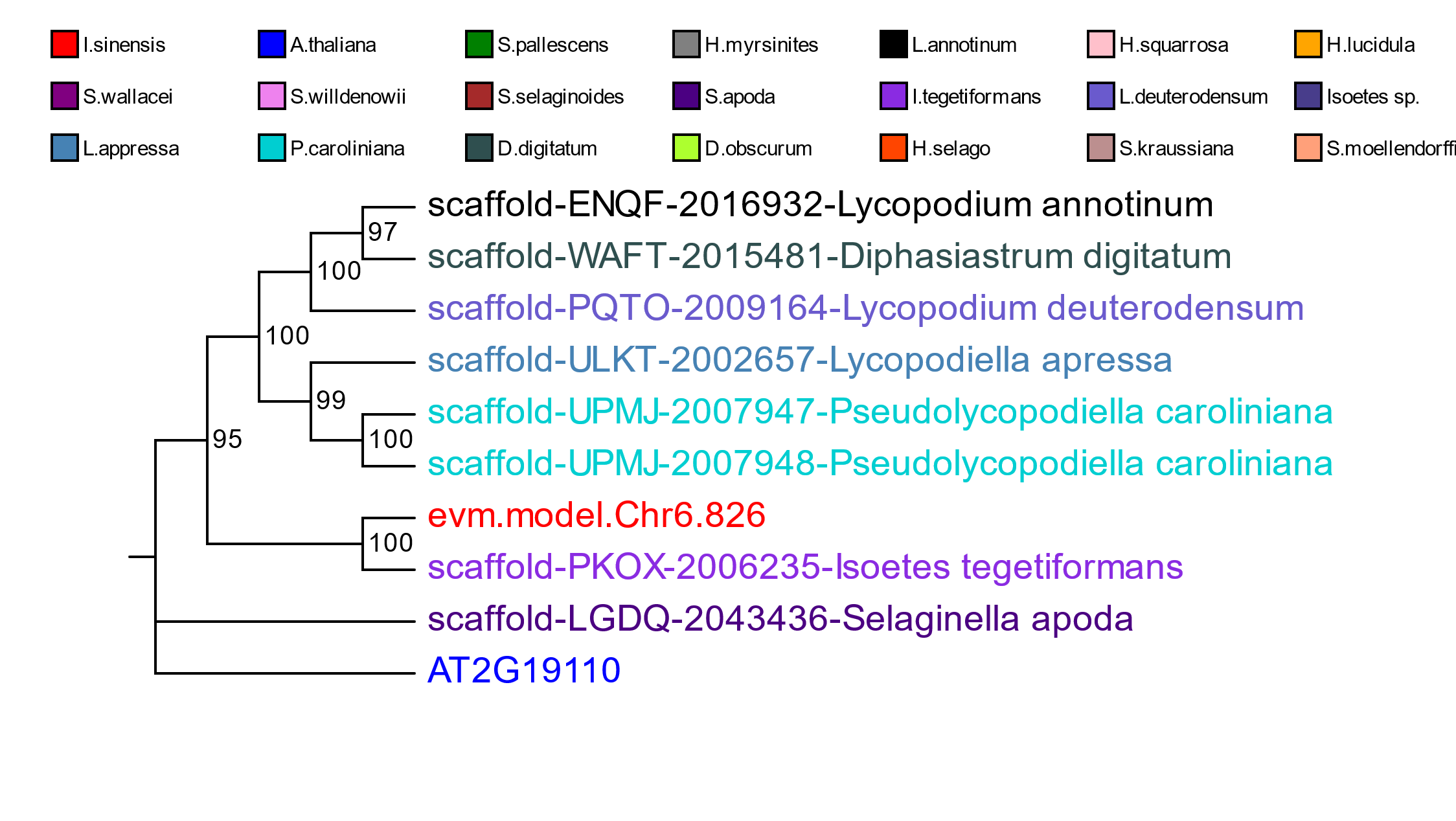


**Dataset S68. Phylogenetic relationships of HMA4 proteins from *I. sinensis* and other lycophyte species.** Numbers on the major branches indicate bootstrap values (> 50%) in 1,000 replicates.
