# Supplementary material for: Chromosome-level reference genome of tetraploid Isoetes sinensis provides insights into evolution and adaption of lycophytes
Source: Gigascience. 2023 Sep 30;12:giad079. doi: 10.1093/gigascience/giad079 (PMC10541799; doi:10.1093/gigascience/giad079)
Supplement: giad079_Supplemental_Files [file giad079_supplemental_files.zip › Supplementary Figure rev 2.docx]

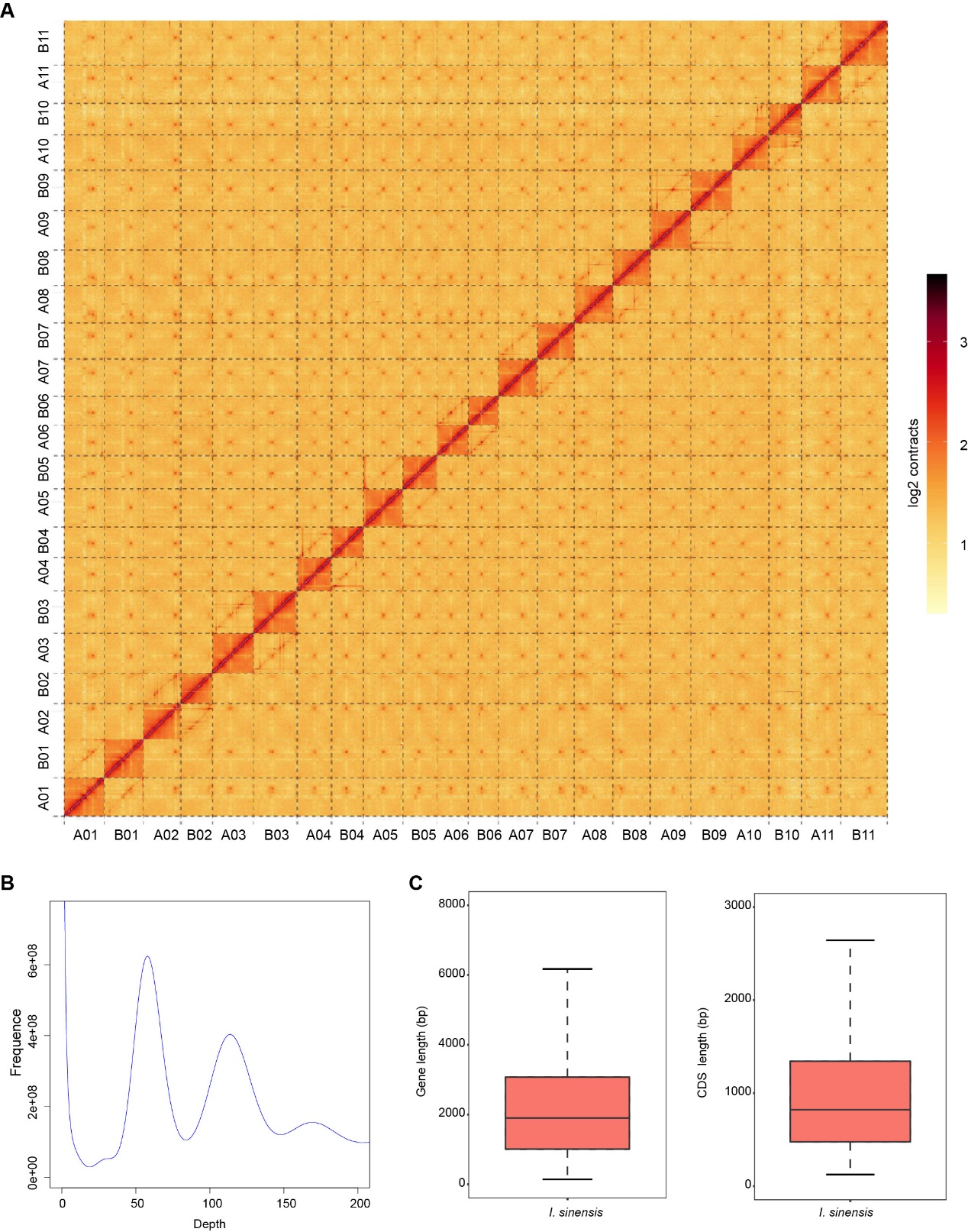


**Supplementary Fig. S1:** *I. sinensis* genome analysis. (A) Interactive distribution of Hi-C links among assembled pseudochromosomes. Normalized Hi-C contact map of *I. sinensis* at a 10 kb resolution. The bar indicates the log10 contact frequency. The heatmap colors range from yellow to red indicated that the frequency of Hi-C interaction links from low to high. (B) Frequency distribution of depth and *K*-mer number. Depth = 57 is the main peak value. (C) Boxplot showing gene (left) and CDS (right) length distribution of *I. sinensis.* Boxes indicate the 1st quartile, median and 3rd quartile with whiskers extending up to 1.5 times the interquartile distance.

**
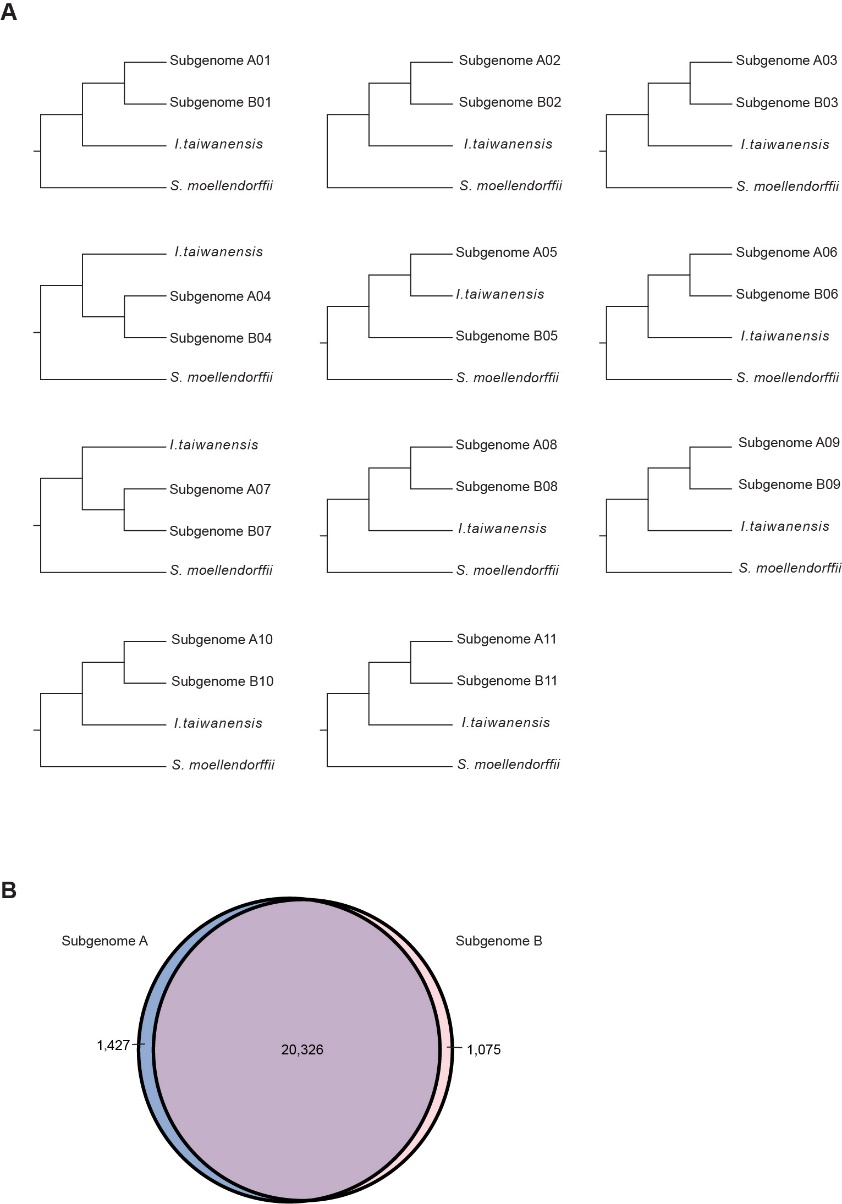
**

**Supplementary Fig. S2:** High similarity between two subgenomes of *I. sinensis*. (A) Phylogenetic analysis showed that the chromosome between two subgenomes of *I. sinensis* was closer than that between subgenome of *I. sinensis* and *I. taiwanensis*, except that chromosome A05 was closer to *I. taiwanensis* than chromosome B05, suggesting that *I. sinensis* was not directly derived from the hybridization of *I. yunguiensis* and *I. taiwanensis*. (B) Shared and unique genes in two subgenomes of *I. sinensis*. The gene number was comparable between the two subgenomes. 93.4% genes of subgenome A were homoeologs of 95.0% genes of subgenome B.

**
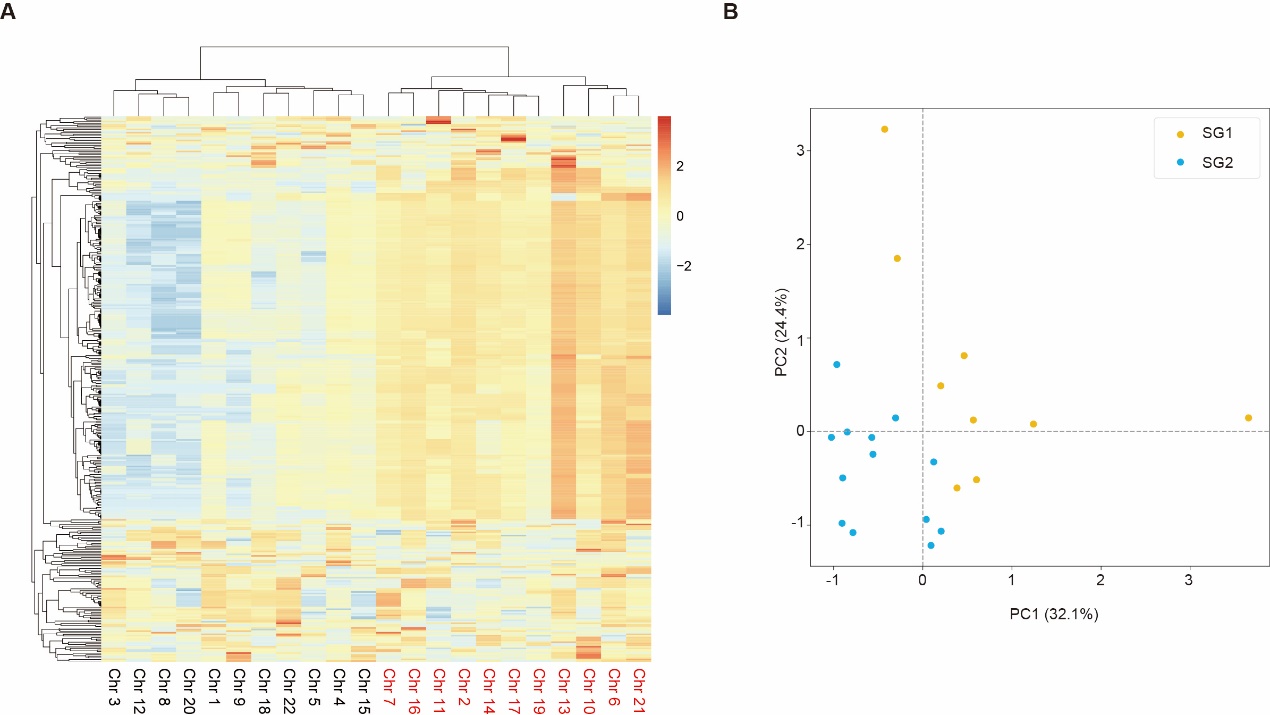
**

**Supplementary Fig. S3:** *K*-mer and Subphaser analysis were not able to separate the subgenomes of *I. sinensis*. (A) Clustering of counts of 13-mers of 22 chromosomes in *I. sinensis.* (B) Principal component analysis (PCA) of differential *K*-mers. Dots indicate chromosomes. SG1: subgenome 1; SG2: subgenome 2.


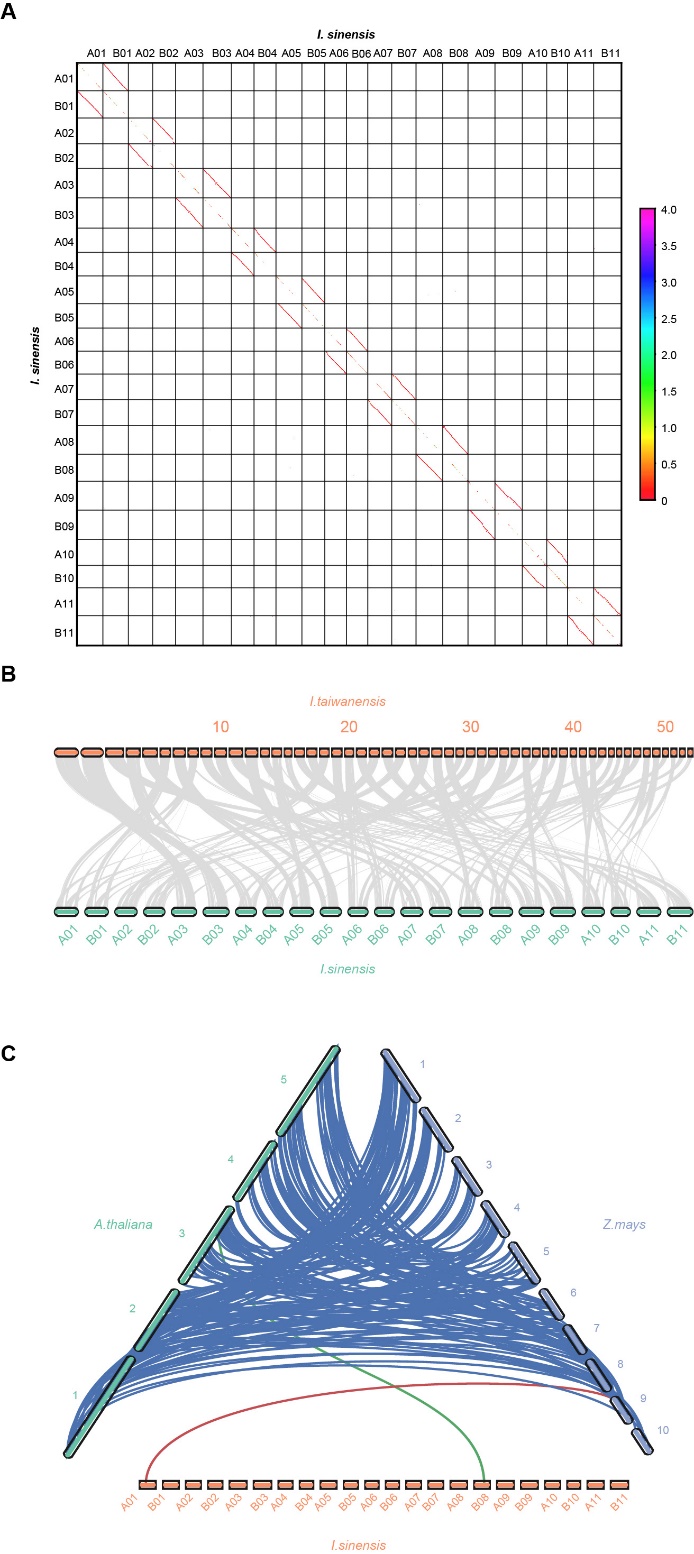


**Supplementary Fig. S4:** Collinearity analysis of *I. sinensis* genome. (A) Collinearity analysis among chromosomes of *I. sinensis.* The collinearity between allelic chromosome pairs (i.e., A01 and B01) was high, while the one between other regions was weak. The bar indicates *Ks* value. The genes used for collinearity analysis were extracted from WGDI, which only considers pairs of homologous genes in analyses, and retains homologous gene pairs related to polyploidization. *Ks* value of gene pairs on synteny blocks was also extracted from WGDI. (B) Collinearity analysis between *I. sinensis* and *I. taiwanensis.* (C) Collinearity analysis between *I. sinensis, Arabidopsis* and maize. Blocks of synteny of at least four gene pairs between the genomes are connected by linked lines (for B and C). The synteny block (4:4) between *I. sinensis* and maize included several duplicated homologs of *I. sinensis*.


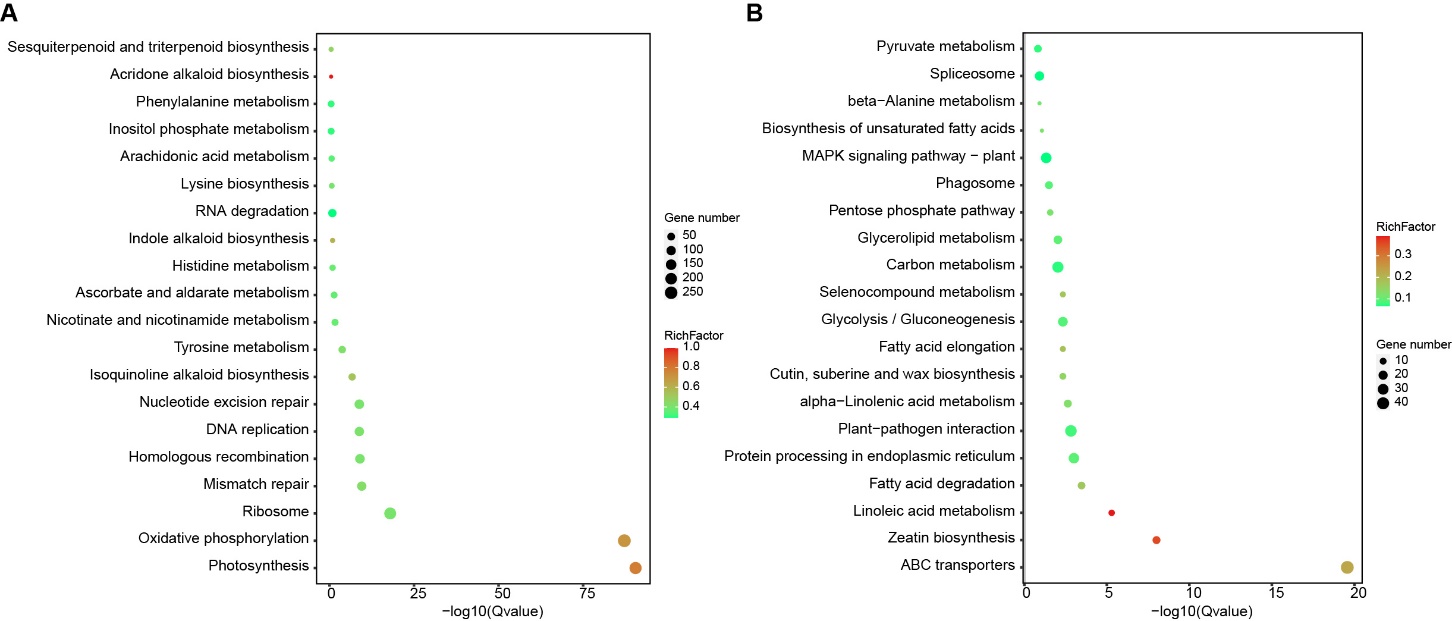


**Supplementary Fig. S5:** KEGG analysis of expansion and contraction gene families during *I. sinensis* evolution*.* (A) KEGG enrichment analysis showing that expanded gene families were mostly enriched in energy metabolism functions such as photosynthesis and oxidative phosphorylation. (B) KEGG enrichment analysis showing that contracted gene families were mostly enriched in lipid metabolism functions such as linoleic acid metabolism and fatty acid degradation.


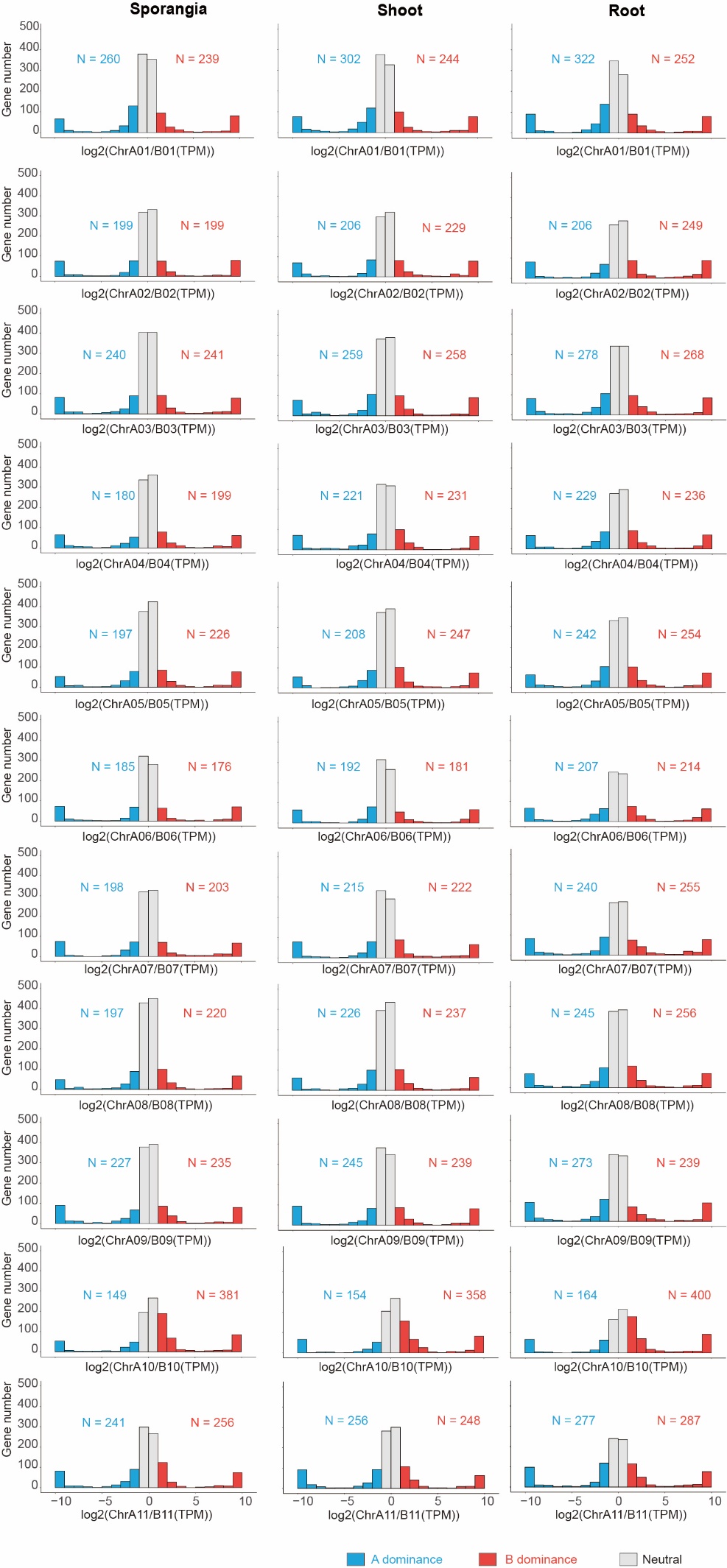


**Supplementary Fig. S6:** Histograms of expression of 1:1 homoeologous genes between pairs of chromosomes among *I. sinensis* sporangia, shoot and root tissues. N values indicate the number of dominant genes in chromosomes A01-A11 and chromosomes B01-B11, respectively. DEG pairs with fold change > 2 were defined as dominant gene pairs. The dominant genes were defined as the genes with higher expression in dominant gene pairs.


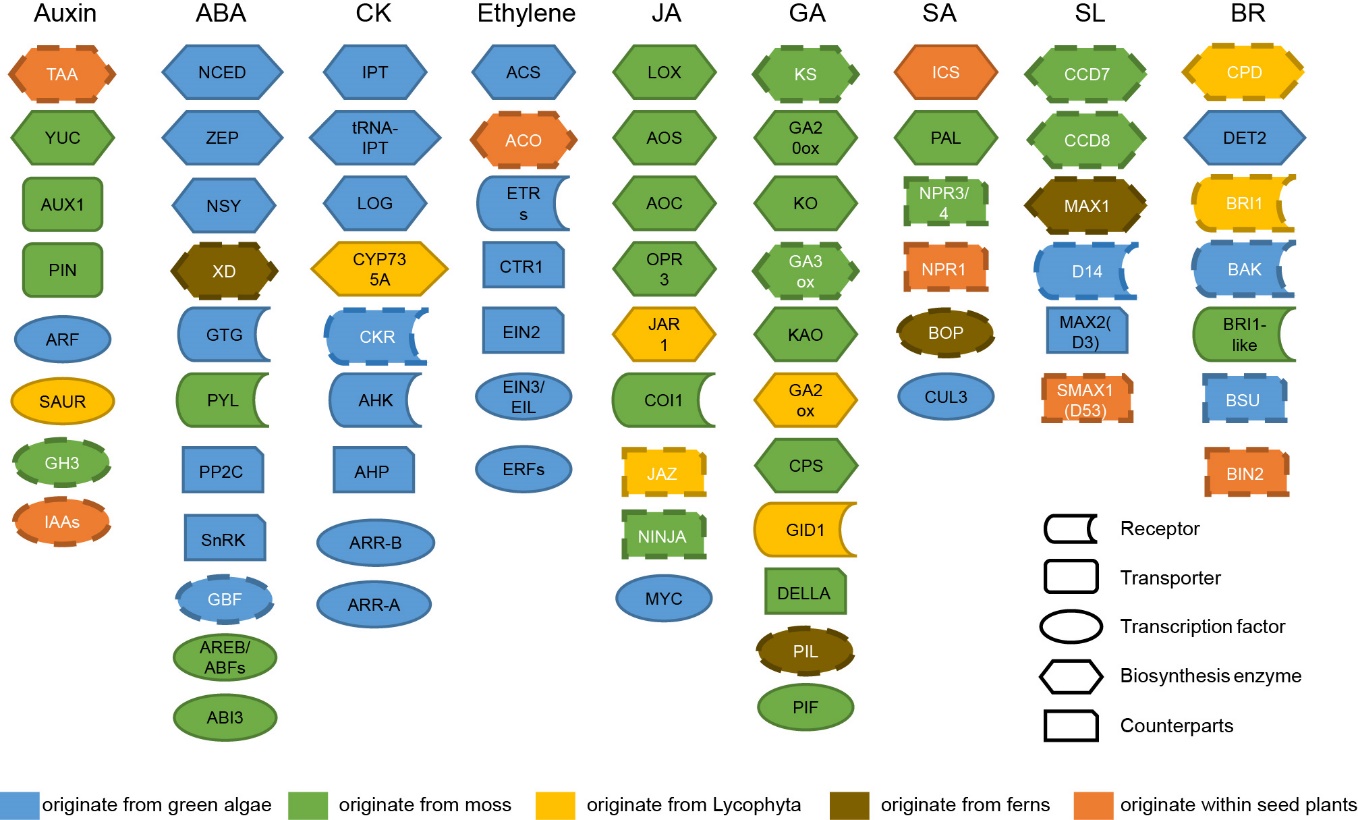


**Supplementary Fig. S7**: Phytohormone biosynthesis and signaling pathways in *I. sinensis*. Diagram showing the evolution of key genes involved in the nine major phytohormone pathways and those that are either present or undetected in the genome *I. sinensis*. Dashed boxes indicate that the gene was not present in *I. sinensis*.


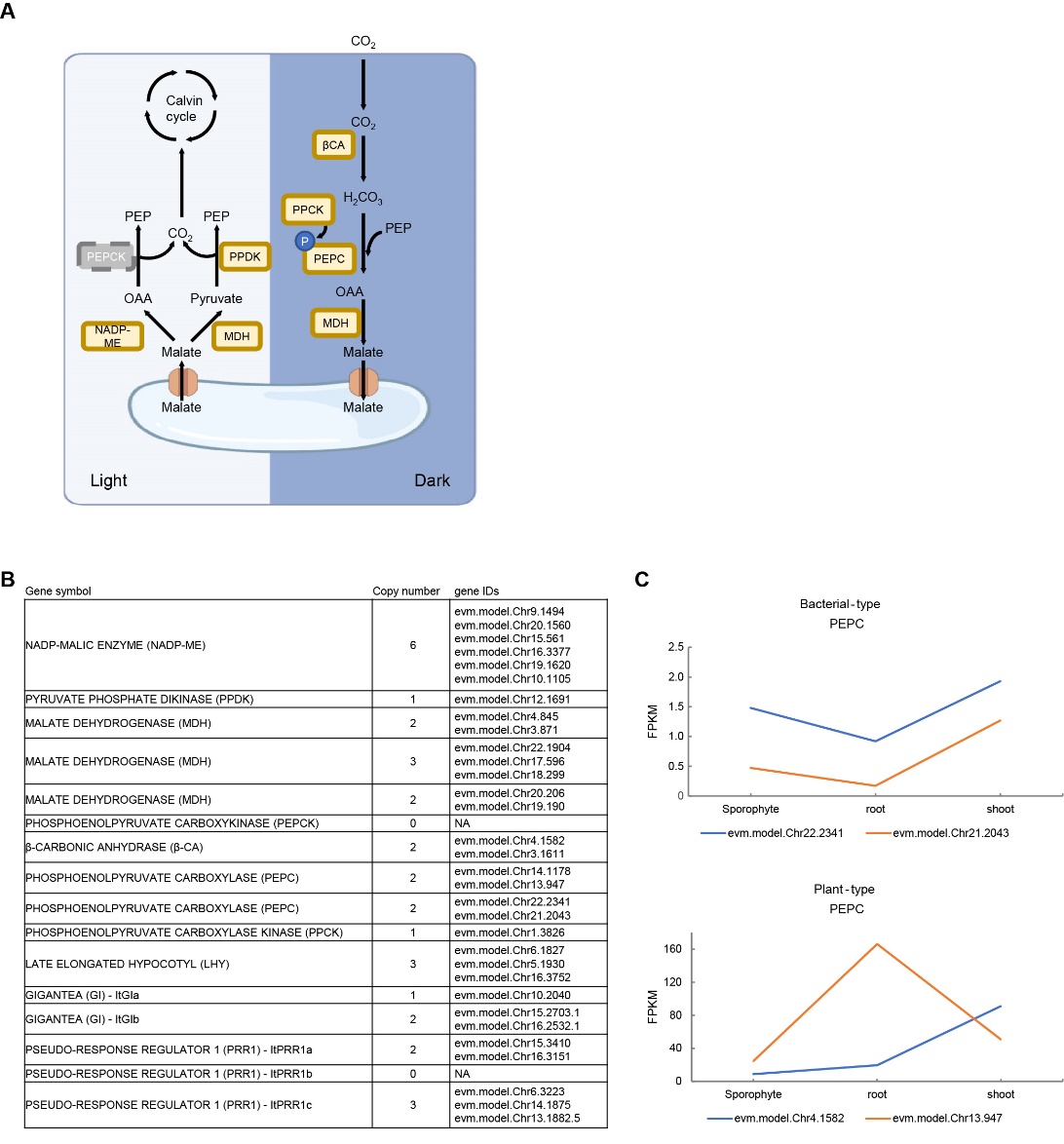


**Supplementary Fig. S8:** CAM related genes in *I. sinensis*. (A) Diagram showing the genes involved in CAM pathway in *I. sinensis.* Coloured boxes indicate the presence of genes in the CAM pathway, gray boxes with dotted lines indicate the absence of the gene. (B) *I. sinensis* lacked of PHOSPHOENOLPYRUVATE CARBOXYKINASE (PEPCK) and PSEUDO-RESPONSE REGULATOR 1 (PRR1) - ItPRR1b compared with *I. taiwanensis*, suggesting differences in mechanisms of CAM within aquatic plants. (C) *I. sinensis* had low-level expression of the bacterial-type *PEPC* and high-level expression of the plant-type *PEPC* in roots, shoots and sporangia, in contrast to the higher expression of bacterial-type *PEPC* than plant-type *PEPC* during development in *I. taiwanensis.*


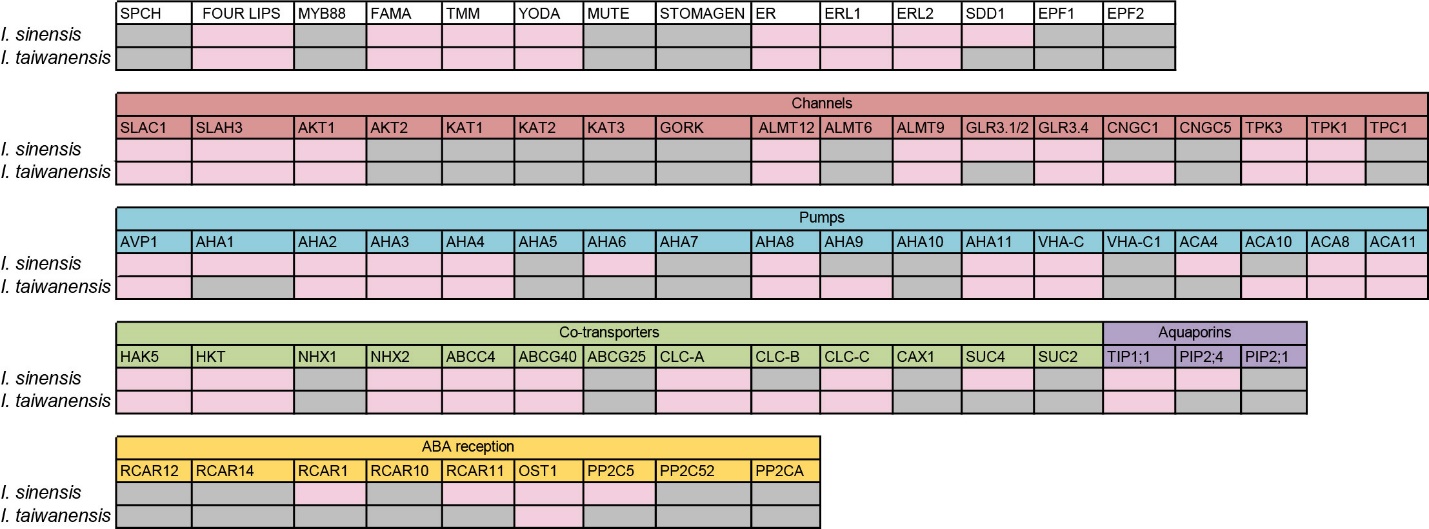


**Supplementary Fig. S9:** Stomatal regulation related genes in *I. sinensis* and *I. taiwanensis*. Heatmap showing the present and undetected of stomatal regulation related genes in *I. sinensis* and *I. taiwanensis*. Some key genes for stomata development, such as *SPEECHLESS* (*SPCH*), *MYB88*, and *MUTE*, are not present both in the genomes of either *I. sinensis* or *I. taiwanensis*, suggesting specialized stomatal regulation in *Isoetes*. Pink represents the presence of the gene and gray represents the absence of the gene.


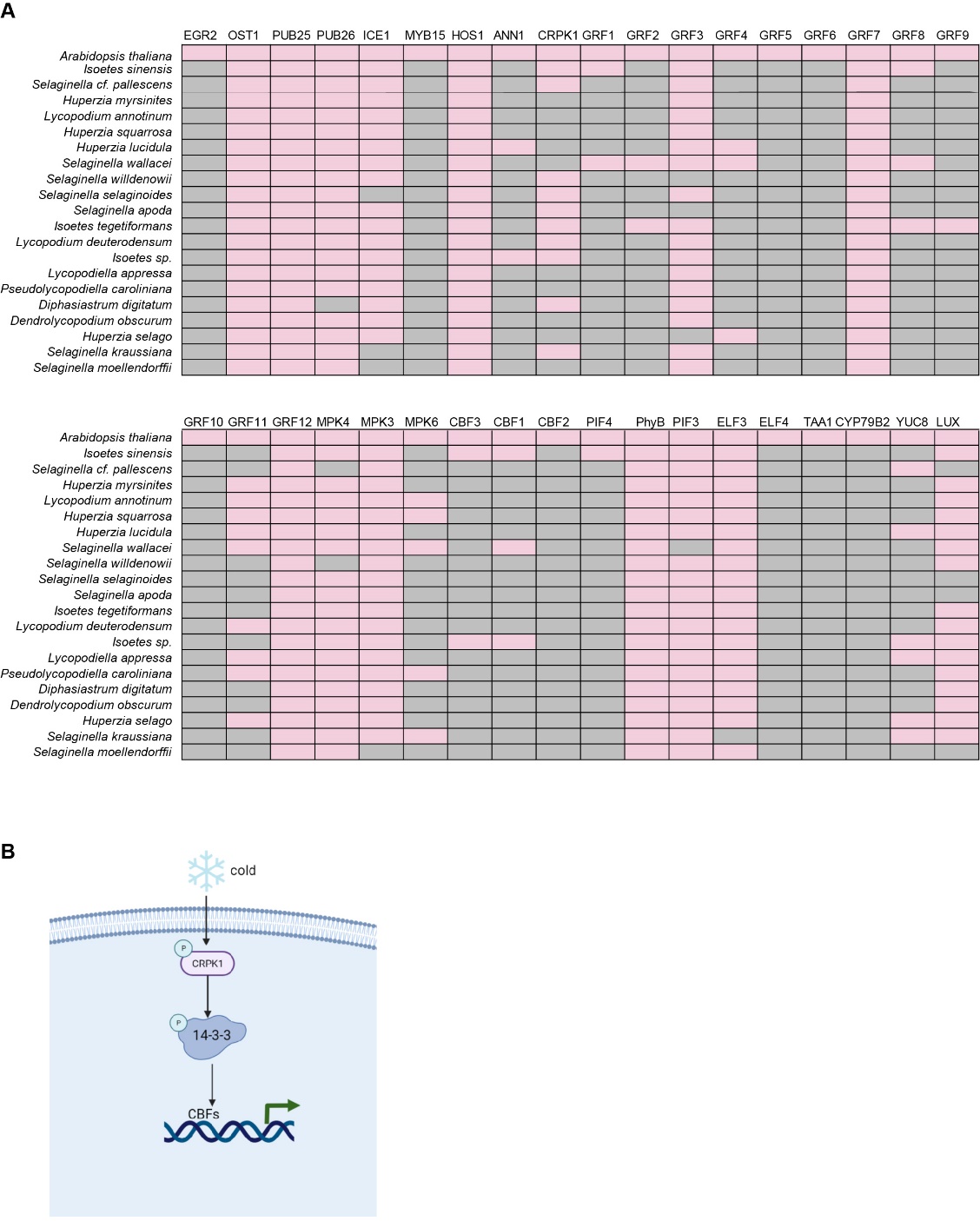


**Supplementary Fig. S10:** Temperature stress related genes in *I. sinensis* and other lycophytes. A, Heatmap showing the presence/absence of temperature stress related genes in 22 lycophytes. Pink represents the presence of the gene and gray represents the absence of the gene. For the genome unavailable species, genes were inferred form RNA-seq data. B, A cold sensing and signaling pathway was shared by lycophytes and flowering plants. Cytoplasmic receptor-like kinase CRPK1 phosphorylates 14-3-3 proteins and promotes their entry into the nucleus, where they interact with CBF and weaken its stability under cold stress.

**
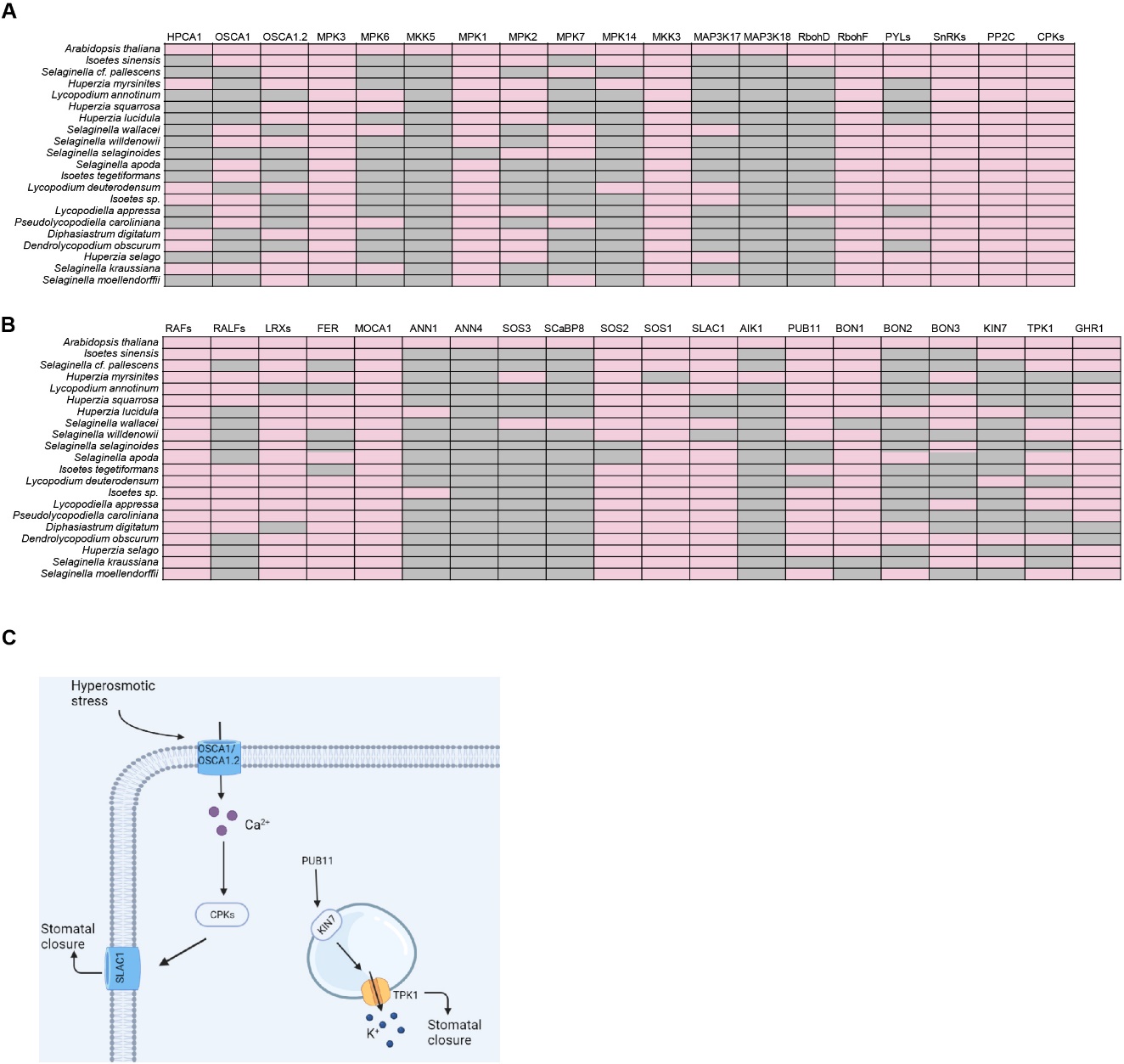
**

**Supplementary Fig. S11:** Salinity and drought stress related genes in *I. sinensis* and other lycophytes. (A and B) Heatmap showing the presence and absence of salinity and (A) drought (B) stress related genes in *I. sinensis* and other lycophytes. Pink represents the presence of the gene and gray represents the absence of the gene. For the genome unavailable species, genes were inferred form RNA-seq data. C, Salinity and drought sensing and signaling pathway shared by lycophytes and other plants. Under drought stress, KINASE 7 (KIN7) is negatively regulated by PLANT U‐BOX 11 (PUB11) for its degradation. KIN7 kinase regulates vacuolar TPK1 K^+^ channel, participates in stomatal closure process, thus playing an important role in drought stress response.

**
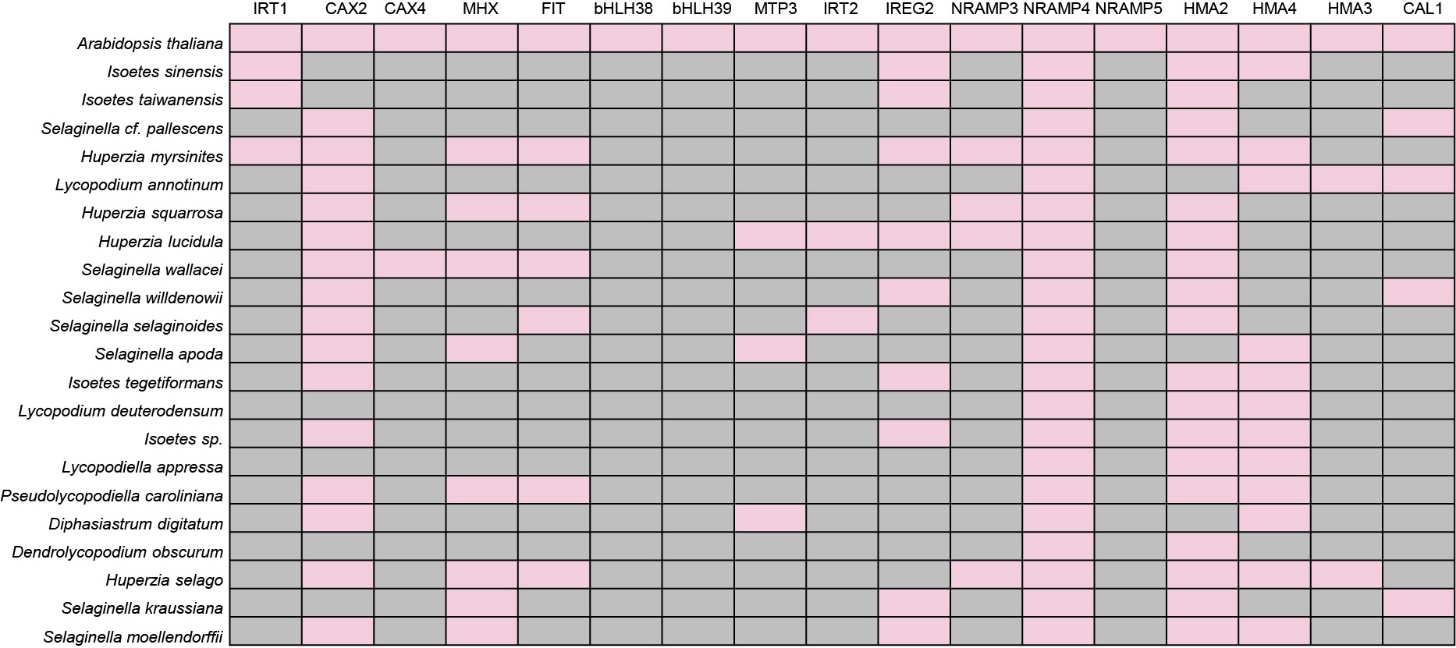
**

**Supplementary Fig. S12:** Cadmium stress related genes in *I. sinensis* and other lycophyte species. Heatmap showing the present and undetected of cadmium stress related genes in *I. sinensis* and other lycophytes. Pink represents the presence of the gene and gray represents the absence of the gene. For the genome unavailable species, genes were inferred form RNA-seq data.
