# Supplementary material for: Chromosome-level reference genome of tetraploid Isoetes sinensis provides insights into evolution and adaption of lycophytes
Source: Gigascience. 2023 Sep 30;12:giad079. doi: 10.1093/gigascience/giad079 (PMC10541799; doi:10.1093/gigascience/giad079)
Supplement: giad079_Response_to_Reviewer_Comments_Original_Submission [file giad079_response_to_reviewer_comments_original_submission.pdf]

# Author's Response To Reviewer Comments

Manuscript No: GIGA-D-23-00116
Manuscript Title: Chromosome-level reference genome of tetraploid Isoetes sinensis provides insights into evolution and adaption of lycophytes

Response to Reviewers

We would like to thank the Editor and two reviewers for the time committed in reviewing this manuscript, and for all the detailed suggestions on improving this manuscript. We have substantially revised the manuscript to address the reviewers' concerns and criticisms as follows. All changes were highlighted in the revised manuscript.

Reviewer #1:
In this study, Cui et al. sequenced, assembled and annotated the genome of tetraploid Isoetes sinensis and analyzed the its evolution and adaption from polyploidization and presence-and-absence of TFs, and genes involved in phytohormone, CAM pathway and environmental stresses (cold, drought, salinity, and cadmium). Generally, the high-quality assembly of polyploid Isoetes will deepen our understanding to plant evolution and provided important genomic resources.
I have some concerns as following:

Major issues:
>Reviewer:
The authors determined the subgenomes of tetraploid I. sinensis based on the length of chromosome pairs (page 5). I am not convinced about the phasing accuracy, although we usually observe the size difference between or among subgenomes in polyploid genomes. In the supplemental Table S3, some chromosomes from A are longer than B chromosomes while the other are shorter. So I do not understand how to determine subgenome by chromosome lengths. Like many other genome papers, e.g. hexaploid Echinochloa (Wu et al., 2022, Nat. Commun.), hexaploid chrysanthemum (Song et al., 2023, Nat. Commun.), subgenome specific K-mers or transposons/LTRs should be investigated to validate the phasing accuracy. In the paper of Artemisia argyi genome (Miao et al., 2022, PBJ), the authors tried to phase the subgenomes using a K-mer approach but failed thus they determined the subgenomes according to chromosome lengths but they did not investigate the subgenome dominance which requires high accuracy of subgenome phasing. Also about 10% of sequences were not anchored on pseudo chromosomes (Page 19), which makes the phasing reliability doubtful. The author should at least supplement a K-mer or LTR analysis to confirm the accuracy of subgenome phasing. Related tools or scripts are available, like SubPhaser (https://github.com/zhangrengang/SubPhaser) (Jia et al., 2022, New Phytol.).
>Authors:
We thank the reviewer for the insight comments and suggestions. We have checked the previous uploaded Table S3 and realized that one was not the final version. However, we used the correct data for all the relevant analysis, thus the results were not affected. We apologize for this mistake and submitted the correct Table S3. As suggested by the reviewer, we have performed K-mer and Subphaser analysis to I. sinensis genome. Clustering of counts of 13-mers identified two groups of chromosomes. However, pairs of chromosomes, such as Chr 3 and Chr 4, were found in same groups (New Supplementary Fig. S3). In addition, Subphaser analysis identified 9 chromosomes in subgenome 1 and 13 chromosomes in subgenome 2. These results suggested that these analyses were not able to separate the two subgenomes of I. sinensis. Therefore, we used the similar approach to Artemisia argyi genome (Miao et al., 2022, PBJ) and artificially determined the subgenomes according to chromosome lengths. We agree with the reviewer that the investigation of subgenome dominance requires the accuracy of subgenome phasing. In the revised manuscript, we removed the subgenome level homoeologs expression bias comparison, and focused on the homoeologs expression between pairs of chromosomes (New Supplementary Fig. S6). Because the majority of our dominance analyses were not based on subgenome phasing, our main findings were not affected by this change.

>Reviewer:

The author quantified the expression bias of homoeologs genes in subgenomes of I. sinensis and I. taiwanensis. It is not appropriate to combine the genomes of diploid I. taiwanensis and tetraploid I. sinensis together, because they are from two different species and the dominance in the pseudo-hexaploid means nothing. The subgenome expression bias has been investigated in many species, such as hexaploid wheat, hexaploid Echinochloa, hexaploid chrysanthemum, and tetraploid Brassica juncea. To investigate the effects of polyploidization on gene expression, the comparison between subgenomes in I. sinensis would be enough to quantify the expression bias.
>Authors:
We agree with the reviewer that the comparison between subgenomes in I. sinensis would be enough to quantify the homoeologs expression bias. As suggested by the reviewer and mentioned above, we have removed I. taiwanensis in the analysis and focused on the homoeologs expression bias between pairs of chromosomes of I. sinensis by using a similar approach that reported in Brassica juncea. On average, 5,206 gene pairs showed homoeolog expression dominance. Notably, the number of dominant genes were comparable between 11 pairs of chromosomes. The exception was found in Chr10, where more than twice dominant genes in ChrB10 than that in ChrA10 (New Supplementary Fig. S6). These results suggest that polyploidization might have affected the relative expression of homoeologs and likely equally affected the two subgenomes except Chr10. We have included the new results in the revised manuscript.

>Reviewer:
In the method part, the author assembled the genome using NGS short reads by SOAPdenovo but this step was absent in Fig 1B. The NGS-based contigs were used to scaffold the contigs generated from hifiasm? The insertion size of Illumina sequencing was 350bp so I doubt the reliability of the contig accuracy. Please describe the assembly workflow more clearly.
>Authors:
Figure 1B is the diagram that depicted workflow for assembly of the I. sinensis genome. The initial contig assembly was based on PacBio long reads using Hifiasm program. The primary contigs were polished by aligning PacBio SMRT reads using the NextPolish software with the default parameters. The consensus sequences for scaffolds were further polished based on Illumina paired-end reads using Pilon. The Hi-C sequencing data were further used for the chromosome-level assembly.
One clarification is that the Illumina NGS-based contigs and scaffold mentioned by the reviewer were used for the survey to evaluate the genomic complexity and genome size, which was before the de novo genome assembly. We have revised the method part to make the workflow description more clearly.

>Reviewer:
The presence and absence of key TFs and genes underlying phytohormone, CAM, stress responses was investigated a lot in this study. But the methodology of such gene identification was not found in Method part. I guess a BLAST-like approach was adopted. The authors should make this clear and the cutoff values (e.g. e value, identity) should be provided, because different cutoffs can lead to different conclusions. Also I wonder where key gene information of these pathways were from, a database or a literature review. Please make it clear.
>Authors:
A BLASTP search (p value < 1e-5) was performed using well-studied proteins (mostly from A. thaliana) as queries to identify the homolog genes in I. sinensis. Following the deletion of redundant sequences, candidates were examined for the typical domain(s) of respective gene families using SMART tool (http://smart.emblheidelberg.de/), and the sequence(s) without the typical domain(s) were filtered out. Multiple alignments of candidate proteins were performed using Muscle with default parameters. The alignments were then manually inspected using MEGA 7. Further analysis only included unambiguously aligned positions. A neighbor-joining (NJ) tree was constructed using MEGA 7 software based on the alignment of candidate proteins. To determine the statistical reliability, bootstrap analysis was conducted for 1000 replicates. We have references and/or IDs (in the phylogenetic trees) for these key genes that used as queries. As suggested by the reviewer, we have included more details in the manuscript.

Minor issues:
>Reviewer:
Page 2: "revealed of genomic features and polyploid of lycophytes" is odd.
>Authors:
We agree with the reviewer and have revised it to “Comparison of genomes between I. sinensis and the I.

taiwanensis revealed of conserved and different genomic features between diploid and polyploid lycophytes".

>Reviewer:
Page 3: The genome of Lycopodium clavatum is also available. See
https://www.biorxiv.org/content/10.1101/2022.12.06.519249v1.full.pdf
>Authors:
Thank the reviewer for providing this information, we have read and added this reference in the revised introduction part.

>Reviewer:
Page 4: A supplemental K-mer distribution plot in genome survey of size and heterozygosity is necessary.
>Authors:
As suggested by the reviewer, we have added K-mer distribution plot in genome survey of size and heterozygosity in Supplementary Fig. 1B.

>Reviewer:
Page 5: Supplemental Fig S3a, "A/B05" rather than "A/B07"
>Authors:
Thank the reviewer for pointing this mistake. We have corrected it in the revised manuscript.

>Reviewer:
Page 6: "only two synteny block between I. sinensis and A. thaliana and Z. mays", how large the two blocks are and what genes are involved. The definition to synteny block should be stated in method.
>Authors:
Two synteny blocks were found between I. sinensis and A. thaliana and between I. sinensis and Z. mays. Blocks of synteny were defined as at least four gene pairs between the genomes, which were stated in the figure legends. The following genes were identified in the synteny blocks:
I. sinensis and A. thaliana
I. sinensis ID A. thaliana ID
evm.model.Chr16.2974 AT3G49740
evm.model.Chr16.2998.1 AT3G49850
evm.model.Chr16.3006 AT3G49725
evm.model.Chr16.3007 AT3G49725
evm.model.Chr16.3046 AT3G49830

I. sinensis and Z. mays
I. sinensis ID Z. mays ID
evm.model.Chr1.1789 Zm00001d046136_T001
evm.model.Chr1.1794 Zm00001d046136_T001
evm.model.Chr1.1797 Zm00001d046136_T001
evm.model.Chr1.1826 Zm00001d046127_T001

>Reviewer:
Page 8: Please add reference to support "2.86% is fewer than other land plants but more than in green algae".
>Authors:
As suggested by the reviewer, we have added the citation for this point.

>Reviewer:
Page 9: No enough evidence to say "number of TF encoding genes increased along with organismal complexity". "We found" not "were found"
>Authors:
As suggested by the reviewer, we have toned down the description as "number of TF encoding genes increased likely along with organismal complexity". In addition, we have corrected "were found" to "we found".

>Reviewer:
Page 14: "The absence of these homologs suggests a diversified or incomplete pathway for ...": it is not appropriate to state "incomplete", the absence just represented the difference or diversification between lycophytes and model plant Arabidopsis.
>Authors:
Thanks for pointing this improper word. We have rephrased relevant description in the revised manuscript.

>Reviewer:
Page 17: Which tissue was selected to sequence, leaf or root? Please make clear. Sentence "A total of 176.46 Gb paired-end reads were obtained for genome survey" was repeated with a statement in page 18 "we used 176.46 Gb Illumina short reads for preliminary evaluation of the genome size, heterozygosity...". Such statement redundancy is observed in many places, please have a careful check and improve the expression to make it brief but clear.
>Authors:
For I. sinensis genome sequencing, the genomic DNA was isolated from shoot sample. As suggested by the reviewer, we have carefully checked and modified the repeated parts in the revised manuscript.

>Reviewer:
Page 19: It would be helpful to supplement LAI (Ou et al., 2018, NAR) to evaluate the completeness.
>Authors:
As suggested by the reviewer, we have added the LTR Assembly Index (LAI) value, which is 9.71 that obtained from LTR_retriever (v2.9.0). It should be noted that in the 103 genomes (Ou et al., 2018), 9.71 is not high compare to many diploid genomes, but among the top in the polyploid genomes. We have included the result in the revised manuscript.

>Reviewer:
Page 23: Sentence “Gene families were clustered using OrthoMCL software with default parameters” is repeated. In the phylogenetic analysis, the authors aligned sequences from difference species and built phylogeny trees. I wonder whether alignment was trimmed before phylogeny construction, considering the large divergence among plant species.
>Authors：
We apologize for the repeat, and have rephrased the relevant description. Prior to phylogenetic analysis, Gblocks software (v.0.91b) (-b5 = h) was used to remove gap regions of the multiple sequence alignments. We have included this information in the revised method part.

Reviewer #2:
The authors have reported a high-quality genome of Isoetes sinensis, which represents an important lineage. They have also revealed the polyploidy history and whole-genome duplications (WGDs) of Ilycophytes. The presence and absence of key genes have provided insights into the environmental adaptation of Ilycophytes. This genomic resource is significant and will attract researchers focused on plant evolution, phylogeny, adaptation, and related areas. I have a few questions and suggestions regarding the manuscript.

Major comments:
>Reviewer:
1. Throughout the entire manuscript, the authors did not perform any analyses related to conservation. Therefore, I suggest that they either delete the related description of conservation in the abstract or conduct some relevant analyses (such as PSMC, genetic diversity, or others).
>Authors:
Thanks to the reviewer’s suggestion. PSMC (Pairwise Sequentially Markovian Coalescent) and genetic diversity analyses requires the sequencing of a number of Isoetes sinensis individuals with different genetic background. However, Isoetes sinensis is an endangered species and the different genetic material was not available. Therefore, as suggested by the reviewer, we have deleted the relevant description about conservation in the abstract in the revised manuscript.

>Reviewer:
2. As a data note article, the quality of the genome assembly should be thoroughly evaluated. However,

this aspect was poorly analyzed in this work. For example, metrics such as QV score (using Merqury), LAI (using LTR_retriever), read mapping rate, and coverage should be added. Additionally, tools like purge_dups could help identify uncollapsed duplications, and this aspect also requires evaluation.
>Authors:
As suggested by the reviewer, we have added the parameters related to the quality of genome assembly in the revised manuscript. For example, the read mapping rate of the Illumina sequencing was 98.58% and the coverage was 99.95%. The LTR Assembly Index (LAI) value, is 9.71 that obtained from LTR_retriever (v2.9.0). The QV score generated from Merqury is 46.1448, and the corresponding error rate is 2.4295e-05.
In fact, previously we have performed purge_dups analysis, 207.32M(9.73%) of the genome sequences were identified as uncollapsed duplications. One clarification is that purge_dups is suitable for haploid or diploid genomes (Guan et al., 2020). However, Isoetes sinensis genome is tetraploid. Notably, uncollapsed duplications were not evaluated in recent published high quality polyploid genomes, such as T2T allotetraploid horseradish genome (Shen et al., 2023). Therefore, we didn’t include the purge_dups analysis in our manuscript.

Minor comments:
>Reviewer:
3. Figure S2 is important as it shows the phylogenetic position and helps readers understand its significance. Hence, I suggest moving this figure into the main text as a separate figure or combining it with others.
>Authors:
As suggested by the reviewer, we have moved the previous Figure S2 to the main Figure 3.

>Reviewer:
4. The different colors in Figure 4 should be explained within the figure itself. It may be more suitable to move this figure to the supplementary section since it only reports gene presence and absence.
>Authors:
As suggested by the reviewer, we have added the colors information in the Figure itself, and moved this figure to Supplementary figure 7.

Relevant references
Guan, D., McCarthy, S.A., Wood, J., et al (2020). Identifying and removing haplotypic duplication in primary genome assemblies. Bioinformatics 36, 2896-2898.
Ou, S., Chen, J., and Jiang, N. (2018). Assessing genome assembly quality using the LTR Assembly Index (LAI). Nucleic Acids Res 46, e126.
Shen, F., Xu, S., Shen, Q., et al. (2023). The allotetraploid horseradish genome provides insights into subgenome diversification and formation of critical traits. Nat Commun 14, 4102.
