# Supplementary material for: Chromosome-level reference genome of tetraploid Isoetes sinensis provides insights into evolution and adaption of lycophytes
Source: Gigascience. 2023 Sep 30;12:giad079. doi: 10.1093/gigascience/giad079 (PMC10541799; doi:10.1093/gigascience/giad079)
Supplement: giad079_Response_to_Reviewer_Comments_Revision_1 [file giad079_response_to_reviewer_comments_revision_1.pdf]

# Author's Response To Reviewer Comments

Manuscript No: GIGA-D-23-00116R1
Manuscript Title: Chromosome-level reference genome of tetraploid Isoetes sinensis provides insights into evolution and adaption of lycophytes

Response to Reviewers

We would like to thank the Editor and two reviewers for the time committed in reviewing this revised manuscript, and for all the detailed suggestions on improving this manuscript. We have further revised the manuscript to address the reviewers' concerns as follows. All changes were highlighted in the revised manuscript.

Reviewer #1:
The authors have addressed most of my questions.
Minor concerns remain:
>Reviewer:
1. Page 6 Synteny between seed free and seed plant genomes: I do not think these two blocks could be considered as synteny blocks between I. sinensis and A. thaliana and and Z. mays (FigS4C). The genes in A. thaliana are completely out of order and three out of the four genes (evm.model.Chr1.1789, 1794, 1797) in I. sinensis are tandemly duplicated homologs to Zm00001d046136_T001 from maize.
>Authors:
We agree with the reviewer and revised the relevant description in the manuscript.

>Reviewer:
2.Table 1: Gap ratio means nothing, and should be removed. "Average" should be added to Gene/CDS/Exon/Intron length or number.
>Authors:
As suggested by the reviewer, we have removed gap ratio and added "average" to Gene/CDS/Exon/Intron length in Table 1.

>Reviewer:
3.Fig1B: Why the texts in the first box are not center aligned as the rest boxed? "Genome polishing", "chromosome scaffolding by HiC".
>Authors:
As suggested by the reviewer, we have revised the format and text in Fig. 1B.

>Reviewer:
4.Fig1C: the legend of circos plot should be 'a/b/c/d/e' rather than "A/B/...". Track a has no unit (Mb). Track b and e show repeat density histogram and number of ncRNA, but I did not see the scale bars.
>Authors:
As suggested by the reviewer, we have changed the "A/B/C/D/E" to "a/b/c/d/e" in the circos plot. We have also added the unit and scale information in the Figure legend of Fig. 1C.

>Reviewer:
5.Fig2: Copia and Gypsy should be italic.
>Authors:
As suggested by the reviewer, we have changed the format of Copia and Gypsy to italic in Fig.2.

>Reviewer:
6.Data availability: None of accession numbers the author provided are accessible now and genome annotation should be also deposited. Before accepting this paper, these data should be public and available. Personally I hope the genome assembly, and annotation can be also submitted to NGDC.
>Authors:
As suggested by the reviewer, we have released all the deposited data, which are available for public. We

have also submitted the genome assembly and annotation to NGDC. However, we didn’t include NGDC accessions in the manuscript to avoid redundance.

Reviewer #2:
The authors have answered all my questions and I agree to accept the article.
>Authors:
We thank the reviewer’s positive comment.
