# Supplementary material for: Chromosome-level reference genome of tetraploid Isoetes sinensis provides insights into evolution and adaption of lycophytes
Source: Gigascience. 2023 Sep 30;12:giad079. doi: 10.1093/gigascience/giad079 (PMC10541799; doi:10.1093/gigascience/giad079)
Supplement: giad079_Reviewer_1_Report_Original_Submission — Dongya Wu -- 7/3/2023 Reviewed [file giad079_reviewer_1_report_original_submission.pdf]

Reviewer Report

Title: Chromosome-level reference genome of tetraploid Isoetes sinensis provides insights into evolution and adaption of lycophytes

Version: Original Submission **Date:** 7/3/2023

Reviewer name: Dongya Wu

Reviewer Comments to Author:

In this study, Cui et al. sequenced, assembled and annotated the genome of tetraploid Isoetes sinensis and analyzed the its evolution and adaption from polyploidization and presence-and-absence of TFs, and genes involved in phytohormone, CAM pathway and environmental stresses (cold, drought, salinity, and cadmium). Generally, the high-quality assembly of polyploid Isoetes will deepen our understanding to plant evolution and provided important genomic resources.I have some concerns as following:Major issues:The authors determined the subgenomes of tetraploid I. sinensis based on the length of chromosome pairs (page 5). I am not convinced about the phasing accuracy , although we usually observe the size difference between or among subgenomes in polyploid genomes. In the supplemental Table S3, some chromosomes from A are longer than B chromosomes while the other are shorter. So I do not understand how to determine subgenome by chromosome lengths. Like many other genome papers, e.g. hexaploid Echinochloa (Wu et al., 2022, Nat. Commun.), hexaploid chrysanthemum (Song et al., 2023, Nat. Commun.), subgenome specific K-mers or transposons/LTRs should be investigated to validate the phasing accuracy. In the paper of Artemisia argyi genome (Miao et al., 2022, PBJ), the authors tried to phase the subgenomes using a K-mer approach but failed thus they determined the subgenomes according to chromosome lengths but they did not investigate the subgenome dominance which requires high accuracy of subgenome phasing. Also about 10% of sequences were not anchored on pseudo chromosomes (Page 19), which makes the phasing reliability doubtful. The author should at least supplement a K-mer or LTR analysis to confirm the accuracy of subgenome phasing. Related tools or scripts are available, like SubPhaser (https://github.com/zhangrengang/SubPhaser) (Jia et al., 2022, New Phytol.).The author quantified the expression bias of homoeologs genes in subgenomes of I. sinensis and I. taiwanensis. It is not appropriate to combine the genomes of diploid I. taiwanensis and tetraploid I. sinensis together, because they are from two different species and the dominance in the pseudo-hexaploid means nothing. The subgenome expression bias has been investigated in many species, such as hexaploid wheat, hexaploid Echinochloa, hexaploid chrysanthemum, and tetraploid Brassica juncea. To investigate the effects of polyploidization on gene expression, the comparison between subgenomes in I. sinensis would be enough to quantify the expression bias.In the method part, the author assembled the genome using NGS short reads by SOAPdenovo but this step was absent in Fig 1B. The NGS-based contigs were used to scaffold the contigs generated from hifiasm ? The insertion size of Illumina sequencing was 350bp so I doubt the reliability of the contig accuracy. Please describe the assembly workflow more clearly.The presence and absence of key TFs and genes underlying phytohormone, CAM, stress responses was investigated a lot in this study. But the methodology of such gene identification was not found in Method part. I guess a BLAST-like approach was adopted. The

authors should make this clear and the cutoff values (e.g. e value, identity) should be provided, because different cutoffs can lead to different conclusions. Also I wonder where key gene information of these pathways were from, a database or a literature review. Please make it clear.Minor issues:Page 2: "revealed of genomic features and polyploid of lycophytes" is odd.Page 3: The genome of Lycopodium clavatum is also available. See https://www.biorxiv.org/content/10.1101/2022.12.06.519249v1.full.pdfPage 4: A supplemental K-mer distribution plot in genome survey of size and heterozygosity is necessary.Page 5: Supplemental Fig S3a, "A/B05" rather than "A/B07"Page 6: "only two synteny block between I. sinensis and A. thaliana and Z. mays", how large the two blocks are and what genes are involved. The definition to synteny block should be stated in method.Page 8: Please add reference to support "2.86% is fewer than n other land plants but more than in green algae".Page 9: No enough evidence to say "number of TF encoding genes increased along with organismal complexity". "We found" not "were found"Page 14: "The absence of these homologs suggests a diversified or incomplete pathway for ...": it is not appropriate to state "incomplete", the absence just represented the difference or diversification between lycophytes and model plant Arabidopsis.Page 17: Which tissue was selected to sequence, leaf or root ? Please make clear. Sentence "A total of 176.46 Gb paired-end reads were obtained for genome survey" was repeated with a statement in page 18 "we used 176.46 Gb Illumina short reads for preliminary evaluation of the genome size, heterozygosity...". Such statement redundancy is observed in many places, please have a careful check and improve the expression to make it brief but clear.Page 19: It would be helpful to supplement LAI (Ou et al., 2018, NAR) to evaluate the completeness.Page 23: Sentence "Gene families were clustered using OrthoMCL software with default parameters" is repeated. In the phylogenetic analysis, the authors aligned sequences from difference species and built phylogeny trees. I wonder whether alignment was trimmed before phylogeny construction, considering the large divergence among plant species.

Level of Interest

Please indicate how interesting you found the manuscript: Choose an item.

Quality of Written English

Please indicate the quality of language in the manuscript: Choose an item.

Declaration of Competing Interests

Please complete a declaration of competing interests, considering the following questions:

- Have you in the past five years received reimbursements, fees, funding, or salary from an organisation that may in any way gain or lose financially from the publication of this manuscript, either now or in the future?
- Do you hold any stocks or shares in an organisation that may in any way gain or lose financially from the publication of this manuscript, either now or in the future?

- Do you hold or are you currently applying for any patents relating to the content of the manuscript?
- Have you received reimbursements, fees, funding, or salary from an organization that holds or has applied for patents relating to the content of the manuscript?
- Do you have any other financial competing interests?
- Do you have any non-financial competing interests in relation to this paper?

If you can answer no to all of the above, write 'I declare that I have no competing interests' below. If your reply is yes to any, please give details below.

I declare that I have no competing interests

I agree to the open peer review policy of the journal. I understand that my name will be included on my report to the authors and, if the manuscript is accepted for publication, my named report including any attachments I upload will be posted on the website along with the authors' responses. I agree for my report to be made available under an Open Access Creative Commons CC-BY license (http://creativecommons.org/licenses/by/4.0/). I understand that any comments which I do not wish to be included in my named report can be included as confidential comments to the editors, which will not be published.

Choose an item.

To further support our reviewers, we have joined with Publons, where you can gain additional credit to further highlight your hard work (see: https://publons.com/journal/530/gigascience). On publication of this paper, your review will be automatically added to Publons, you can then choose whether or not to claim your Publons credit. I understand this statement.

Yes Choose an item.
