# Supplementary material for: Chromosome-level reference genome of tetraploid Isoetes sinensis provides insights into evolution and adaption of lycophytes
Source: Gigascience. 2023 Sep 30;12:giad079. doi: 10.1093/gigascience/giad079 (PMC10541799; doi:10.1093/gigascience/giad079)
Supplement: giad079_Reviewer_2_Report_Original_Submission — Yongzhi Yang, Ph.D. -- 7/5/2023 Reviewed [file giad079_reviewer_2_report_original_submission.pdf]

Reviewer Report

Title: Chromosome-level reference genome of tetraploid Isoetes sinensis provides insights into evolution and adaption of lycophytes

Version: Original Submission **Date:** 7/5/2023

Reviewer name: Yongzhi Yang, Ph.D.

Reviewer Comments to Author:

The authors have reported a high-quality genome of Isoetes sinensis, which represents an important lineage. They have also revealed the polyploidy history and whole-genome duplications (WGDs) of Ilycophytes. The presence and absence of key genes have provided insights into the environmental adaptation of Ilycophytes. This genomic resource is significant and will attract researchers focused on plant evolution, phylogeny, adaptation, and related areas. I have a few questions and suggestions regarding the manuscript.Major comments:1. Throughout the entire manuscript, the authors did not perform any analyses related to conservation. Therefore, I suggest that they either delete the related description of conservation in the abstract or conduct some relevant analyses (such as PSMC, genetic diversity, or others).2. As a data note article, the quality of the genome assembly should be thoroughly evaluated. However, this aspect was poorly analyzed in this work. For example, metrics such as QV score (using Merqury), LAI (using LTR_retriever), read mapping rate, and coverage should be added. Additionally, tools like purge_dups could help identify uncollapsed duplications, and this aspect also requires evaluation.Minor comments:3. Figure S2 is important as it shows the phylogenetic position and helps readers understand its significance. Hence, I suggest moving this figure into the main text as a separate figure or combining it with others.4. The different colors in Figure 4 should be explained within the figure itself. It may be more suitable to move this figure to the supplementary section since it only reports gene presence and absence.

Level of Interest

Please indicate how interesting you found the manuscript: Choose an item.

Quality of Written English

Please indicate the quality of language in the manuscript: Choose an item.

Declaration of Competing Interests

Please complete a declaration of competing interests, considering the following questions:

- Have you in the past five years received reimbursements, fees, funding, or salary from an organisation that may in any way gain or lose financially from the publication of this manuscript, either now or in the future?

- Do you hold any stocks or shares in an organisation that may in any way gain or lose financially from the publication of this manuscript, either now or in the future?
- Do you hold or are you currently applying for any patents relating to the content of the manuscript?
- Have you received reimbursements, fees, funding, or salary from an organization that holds or has applied for patents relating to the content of the manuscript?
- Do you have any other financial competing interests?
- Do you have any non-financial competing interests in relation to this paper?

If you can answer no to all of the above, write 'I declare that I have no competing interests' below. If your reply is yes to any, please give details below.

No. I declare that I have no competing interests.

I agree to the open peer review policy of the journal. I understand that my name will be included on my report to the authors and, if the manuscript is accepted for publication, my named report including any attachments I upload will be posted on the website along with the authors' responses. I agree for my report to be made available under an Open Access Creative Commons CC-BY license (http://creativecommons.org/licenses/by/4.0/). I understand that any comments which I do not wish to be included in my named report can be included as confidential comments to the editors, which will not be published.

Choose an item.

To further support our reviewers, we have joined with Publons, where you can gain additional credit to further highlight your hard work (see: https://publons.com/journal/530/gigascience). On publication of this paper, your review will be automatically added to Publons, you can then choose whether or not to claim your Publons credit. I understand this statement.

Yes Choose an item.
